# Supplementary material for: The implementation, use and sustainability of a clinical decision support system for medication optimisation in primary care: A qualitative evaluation
Source: PLoS One. 2021 May 3;16(5):e0250946. doi: 10.1371/journal.pone.0250946 (PMC8092789; doi:10.1371/journal.pone.0250946)
Supplement: S2 Appendix — (DOCX) [file pone.0250946.s002.docx]

**Main themes, secondary themes and codes**

**Main Themes**

**What is the CDS?**

**Why implement it?**

**The purpose and reasoning for its implementation**

**Across all themes: - Reflexion and monitoring.**

**Does the implementation work?**

**Does CDS work?**

**PINCER v CDS**

**How do people know that it works?**

**What evidence to they gather and how?**

**Using CDS as a feedback system**

**How to implement the CDS**

**The processes involved in change**

**Building, maintaining CDS profile – priorities and expectations**

**Flow of information between different stakeholders and stakeholder groups**

**The work required to implement the CDS**

**The contexts in which the CDS is implemented**

**Outer contexts - The varied contexts in which prescribing takes place in primary care**

**practice, patient and prescriber characteristics**

**Practice, patient and prescriber characteristics**

**Inner contexts - What influences what prescribers do - Balancing expertise, knowledge and information in decision-making processes**

**Work, practices and workflow – fitting the CDS in, the impact of CDS on work**

**Learning, Education and Change**

**Main themes, secondary themes and codes**

1. **What is Optimise? Why implement it? The purpose and reasoning for its implementation**
   - 1. **The reason for CDS- ‘It’s a great safety net’ – Implementing and using the CDS because of its role/potential role in medication safety**
     2. **The purpose of the CDS : to enhance safety or to improve cost effectiveness?**
     3. **Value**
2. **How to implement the CDS-The processes involved in change**
   - 1. **Getting used to the system - the implementation journey**
     2. **Implementation - Direction of travel**
     3. **Implementation drivers**
3. **The work required to implement the CDS**
   1. **Building, maintaining the CDS profile – priorities and expectations**
      1. **Management, maintenance and customisation of Optimise**
      2. **Profile management-Processes and approaches**
      3. **Tailoring messages in profile management and set-up to avoid excessive alerts**
   2. **Flow of information between different stakeholders and stakeholder groups**
      1. **Collaboration and engagement between CCG and general practices**
      2. **Communication, collaboration and engagement - MMT, CCG and Software developer**
4. **The contexts in which the CDS is implemented**
   1. **Outer contexts - The varied contexts in which prescribing takes place in primary care including practice, patient and prescriber characteristics**
      1. **Patient characteristics**
      2. **Patients – impact upon, engagement with and adaptation for**
      3. **Prescriber characteristics**
      4. **Prescriber interaction with patient**
      5. **Prescribing contexts in general practice**
   2. **Inner contexts - What influences what prescribers do - Balancing expertise, knowledge and information in decision-making processes**
      1. **Expertise – the CDS as a knowledge base**
      2. **Broader decision-making process**
      3. **Expertise and prescriber knowledge**
      4. **Prescriber decisions and decision making**
      5. **Relevance and reliability of alerts**
5. **Work, practices and workflow – fitting the CDS in, the impact of the CDS on work**
   - 1. **Fitting within or alongside other interventions and technologies**
     2. **Efficiency, Functionality, Ease of use, User interface**
     3. **The CDS and the context of General Practice work - spaces, people, roles**
     4. **Receiving too many alerts in practice**
     5. **Technology - development, problems, limitations, solutions; relationship of technology to the engagement of users**
     6. **Time**
6. **Learning, Education and Change**
   - 1. **Learning and Behaviour change**
     2. **Learning Feedback and Education**
7. **Reflection and Monitoring**
   - 1. **Using the CDS as a feedback system**
     2. **Does the CDS work?**
     3. **PINCER v the CDS**
