# Supplementary material for: The implementation, use and sustainability of a clinical decision support system for medication optimisation in primary care: A qualitative evaluation
Source: PLoS One. 2021 May 3;16(5):e0250946. doi: 10.1371/journal.pone.0250946 (PMC8092789; doi:10.1371/journal.pone.0250946)
Supplement: S1 Data — (PDF) [file pone.0250946.s003.pdf]

ALL EXTRACTS FROM CODING OF TRANSCRIPTS

Theme 1: What is (the CDS)? Why implement it? - The value, purpose and reasoning for its implementation.

Sub-theme 1.i: The reason for (the CDS) - 'It's a great safety net' – Implementing and using (the CDS) because of its role/potential role in medication safety

Software developer staff

Software Developer staff 1

R: One of the best descriptions of (the CDS) it's like having a medicines management pharmacist or technician sitting by your side, looking at your choice of medicine, going actually for this patient, you might not want to choose that; or what you're picking there isn't on our formulary; or hang on a minute, that's red; or whoa, look at their, you know, renal function. GPs have to be…they have to be a master at everything but they can't remember everything. Guidelines update on a regular basis. I think if you asked most GPs are they on top of all the new NICE tags, the new NICE clinical guidance, the new MHRA warnings. You know, if I think about, say, the (place) area, so I know that the (place) formulary has just been recently updated in December for the new COPD guidance. Now, would I expect every single GP to have…it would be nice if they all sit down and read those guidance and go, right, I exactly know which inhaler I'm meant to be using, what FEV the patient's meant to have before I initiate a steroid, when am I meant to use…we can't expect them to remember all of that, and that is when…[…] Yeah, our software can pull all bits of information and at that point of prescribing, steer them in the right path to say actually, this is the type of inhaler, or, you know, if it's an asthma patient, a gentle reminder that yes, there may be a steroid on their prescription and a long active beta 2 agonist, but this asthmatic actually hasn't requested that steroid for 12 hours, but are always having their Salmeterol, that's, you know. It's more from the safety and best practice.

CCG staff

CCG Pharmacy Technician 2

R: Yeah, I think I could probably in the future go out there and maybe promote it a bit more but then why bother when our acceptance rate is above average and we've got so much more other things to be doing. And like I said I don't think it's the ultimate solution for helping with prescribing but it complements and it, I think it definitely in terms of safety messages where, if we didn't have this system, we would be constantly sending out updates, you know, so many times of week of things that they need to look out for. So you know, GPs don't always have time to log onto their e-mails and read everything or open up, you know, journals and things and read them. So having it there at the point where it would be relevant to that patient is, I think is very, very useful. And I think that in itself would save money because potentially some of the safety stuff could potentially stop someone from going into hospital or they could be directed to something they should be having instead of the old thing they had before, that is no longer recommended. So, and that's hard to quantify in terms of cost but it will have a cost attached to it.

CCG Pharmacy Technician 2

So, I think that's a big thing, and a massive impact on safety, as well. I tend to feel this is more focused on that, which, actually, I feel more reassured, because it's hard to keep up and every day alerts come out, safety alerts. So, I think safety-wise, if it proves that it is working, by whatever it means that it does, whatever outcomes we get in the end, I think that's more important than the switch from this to that. Because we do that anyway, as a team, and it's not the whole picture, it's not what the NHS is all about. We do have to tighten our belts completely, but it doesn't fit every... It's a grey area and not every switch will suit everybody, but guidance, have you seen national guidance and safety alerts? This is why. They're, kind of, this is to keep you safe […] So, I think, for me, the most important thing would be the safety and the quality side, for sure, yeah.

CCG Pharmacist 2

I'm hopeful the safety perspective and antibiotics and things like that, I'd hope that it would be making things better for them because the safety net is there. I feel a lot of it maybe… I don't know without knowing what it looks like in terms of alerts and things like that but I feel a little bit more confident that there are those safety messages there and not relying solely on the clinical systems where sometimes there isn't an alert. It's just something that's in that system on one page where there's a whole load of other information on there where things could get missed.

CCG Pharmacist 2

But, I think clinicians generally, feel a little bit more assured, the prescribing group that I go to, that's where I report, in terms of medication incidents that we have locally, or if somethings happened, or a drug safety update comes up, or a patient safety alert comes nationally, they're like, have we got a message for that, or is there a way of trying to put as many blocks into the Swiss cheese, as possible, to try and prevent that happening? And he CDS seems to be one of the slices with a block in it, sort of thing, in addition to the clinical systems.

CCG Pharmacist 3

R: The point of (the CDS) is it's to do with safety and quality, yes, it is cost, but I was actually doing a course when I started this job. So I used a formulary as my innovation project as such. And when I looked in detail at how we use the formulary and whatnot, I was able to identify gaps and problems. So along with doing that project, I felt what did we need to try and fill those gaps? And some of the things that I identified was first and foremost, the knowledge that we're expecting the prescribers to hold in their heads. It's just not feasible anymore. We're changing things constantly because new information is coming out, new decisions are being made. And they need something to help them, because they've got a lot of responsibility and they've got a person sitting in front of them, they're supposed to know everything about the patient, the best medication for them, but then also we're nipping away at their heels about cost. So we needed something there and it's supposed to be patient-centred care, so it's about looking at what is best for the patient.

GP Staff

GP Nurse 1

I: So having the alerts then how much do you find that helpful?

R:	It is helpful because sometimes if you're in a hurry you tend to cut corners so it's a back-up, and if you haven't checked something, again, it's a back-up or if you didn't know something it's a back-up, because you don't know what you don't know sometimes so that's a back-up for you again. So it's good to have that, that's like that extra person saying, don't forget that, and you go, oh yeah, I can't do that.

GP Nurse 2
R:	One of the things that I do find really useful is that it comes up a lot about the allergies. It's very big on that so it's a great safety net.

GP Pharmacist 2
R:	Yes. I suppose it's another way of having that swiss cheese effect, isn't it? […] I mean, you're still going to have a degree of human error, I mean, you know, there is, there's still going to be harm. But, all of these things though, open you to litigation, I suppose. Not litigation, you know, there's a degree of responsibility, if you know that you have a tool and, you know, and there's potentially a person sat there, who could have been looked at, who hasn't been looked at, and is subject to potential harm. You've got responsibility, haven't you, as a clinician, because that information is there for you to access.

GP3
Because that's the whole point. If I was perfect I wouldn't need anything. So no, these reminders that are both educational and just summing up perhaps we're all human, we make prescribing errors. It just reminds you. It keeps you straight.

GP10
I:	I mean, we're coming up to, well we're way over time actually, you've given me lots more time than I actually promised you, but we'll round up now, but one of the final things then, to finish up with, is, in what way does it improve care? Or, does it improve care?

R1:	It certainly has the capacity to. And it's like all software tools, it depends on the programmer doesn't it? So, I think it's very likely to improve care when it's triggered. So, assuming the rules that are in it, it's such a complex thing isn't it, care, that any model of care, including the one that drives how it's programmed, is going to be imperfect, and that's why I think you still need the PINCER stuff to check that what you think is a good idea, actually turns out to be one. But I think, there's direct ways it improves care, by, if you make the assumption that NICE guidelines are correct, which they probably are the best, I have quite a lot of faith in NICE, it's as robust a process as we can get to get the best population evidence to the individuals we're caring for. So, if you accept that, and then if you accept that it's programmed with NICE guidance to highlight deviations from it, then more patients are likely to end up on evidence based protocols of care, and fewer patients off them, and more patients, when they are off the evidence based protocols, that that's done with thought, rather than just by default.

GP14
R:	Yeah, so, I think from my perspective it's probably two-fold. It's to alert you really, if there are any major safety issues with the drug that you are essentially trying to prescribe, so, the fact it, sort of, links in to medical records and their current medication history as well. It will pick up if you try and prescribe some Penicillin to somebody who's Penicillin allergic and maybe it hadn't just occurred to you. So, that's the one side of things which is really important. But then also being the prescriber I'm really keen for cost effective prescribing, so, there's the alternatives that sometimes flash up, have you considered prescribing this instead of this. It gives you that option to rethink actually and think, well, actually, that drug's basically the same but cheaper and not going to do any harm, so, that's a perfectly reasonable suggestion, I'll do that. I still learn stuff off it, and I always encourage my registrars who are training in the practice as well to, you know, for cost effective prescribing as well.

<u>Sub theme 1.ii: The purpose of (the CDS): to enhance safety or to improve cost effectiveness?</u>

<u>Software developer staff</u>

Software developer staff 1
With my old CCG hat on, because I used to be a CCG pharmacist in the (name of place), area and in (name of place), cost, always cost. And it's the one thing that we try not to focus on, but we can't not because every financial year, the financial teams will come along and say, right, so medicines management, we want you to save £1.5 million this year. And sort of it's the one place that finance know that probably we can try and get savings, other than cutting services. Because that's all CCGs do to save money is cut this service and cut that and make few redundancies. And for the medicines management team, it's right, what can we do cheaper. It's getting harder and harder now because there are no what we call big wins anymore, you can't switch all to one brand and expect to save £750,000. So by adding…we can send pharmacists and technicians out to do that switch work but even better, why can't we switch someone at the point of prescribing when they first started, so the technicians could be doing other things like care home work where there are huge savings to be made, you know, direct…it allows medicines management team to direct the work to what I consider to be more important than going in and doing switch work.

Software developer staff 2
I:	Yes, I think so. Basically, yes, they are different. One of the things actually within those messages as well, we're talking about messages, what might be the last bit we talk about, is again…and I know this is something which we've talked about before. But when I go round, talk to CCGs, talk to people in GP practices there is this ....is it about cost, is it about safety? And obviously many people turn to me and say well, I want it to be about safety, safety's the important thing to me, but yes, it's also about cost. Is it actually about cost versus safety or are they sort of mixed together? Or are you seeing more that it's being used for cost now?
R:	I think it's not a cost versus safety, but I think customers… So assuming that a large proportion of our customers have used another system before they used (the CDS). They were attracted to (the CDS) because we have deeper information and we can be more specific to the patient and probably have a greater focus on best practice and safety
I:	Yeah.
R:	However, they run with that and then it's like…but at the same time still being driven by an economic consideration. They've got quick plans, quick targets, and they have to fulfil those. And it just strikes me just from conversations, the more successful they are, the bigger target they get the next year [voices overlap 0:33:41].
I:	Yeah.

R: So yes, savings are still very important. I think what…and you've got a sense of that from the information (name) gave about the economic evaluation, looking at [inaudible 0:33:59] PROTECT study will enlighten on some of this. But actually, the simple cost savings that a few years ago are diminishing there's still housekeeping that could be done and you could get some cost savings, but making sure you're on top of those opportunities. But really the greatest saving economically that we all as professionals prescribe to is if you're prescribing the right things to the right patients they'll get the better outcomes, they'll not develop [inaudible 0:34:29] all that. Everybody kind of gets that, but nobody is kind of selling that in their CCG.

Software developer staff 3
But, as well as that, there was always the piece that prescribing is more than just about safety. Safety is a massive element of the work, but around the same time as this was all happening, I think (name of University) were doing their report around meds optimisation, and the whole concept of meds optimisation was appearing, and that was an extension from safety into best practice prescribing and also cost definitely became a clear driver, especially in primary care. And so, I guess, the guys within the teams, always knew there was a better way of doing what we're currently doing or were currently doing in terms of our core CDS. So, the idea of creating a solution, which was able to support clinicians to prescribe in a more appropriate way, by giving them actual guidance that would affect their decision making, evolved from there.

Software developer staff 3
There are also other drivers in healthcare and costing, one of them, it may amaze you to know that we weren't always going to put a cost swap element to it, to the solution, so initially the solution was around safety and best practice guidance, but to enter the market, we needed to, there was an incumbent there, which I'm sure you know who that is, who was offering a cost efficiency, cost swap solution, we had to look to deliver that, because that was a fundamental requirement [voices overlap 00:14:41].

CCG staff

CCG Pharmacy Technician 2
R: I would say, and I don't know the exact ratios, most of it would be quality and safety. Pre- (the CDS), we'd have a work stream that the technicians used, of things that we would go and spot out, when we're in the surgeries, to do with cost, alongside our other duties. But those things are on the work stream, we've already looked into: it's bio-equivalent, it's available in our local pharmacies, and it's having a good uptake. So we've checked that out first, so these are these things, were added on to (the CDS), and I wouldn't say that was thousands of lines. So, I would say, generally, it's quality and safety. I would say that would be the figure ratio. But then there are also messages that maybe have prevented a drug being started, because it should have gone into the hospital. Now, that could be a quality thing, because it needs to remain under the specialist, because they need to... Because, potentially, if a GP prescribed it, I mean, I know they're allowed to prescribe what they wish, but they're taking that responsibility on, of this, what a specialist would keep a closer eye on. So, that could, potentially, prevent someone having like an adverse reaction and ending up back in hospital, but it's also the cost side of it.

CCG Pharmacy Technician 2
I: Yeah, and I mean if, from your point of view those benefits are, does it improve safety or can it improve safety?

R: Safety, I mean I think the switching from one thing to another to save money, it's okay for some things but it's not the answer for everything and actually can cause more problems. It can cause supply issues and…I try to be very, very careful what we recommend switching something to something else that's the same sort of, it's the same drug but more cost effective, but I wouldn't say, and we have had some successes from that but we do it very carefully and we have, we try not to have it as…I mean some CCGs it's just absolutely everything. You know like they try and find everything like a change for something else. But you have to think also about who's getting it at the end, the patient at the end. You could be sending these changes out to someone who maybe has a dosette tray, which is like a weekly filled tray by the pharmacy, they rely on what the tablets look like and if they change from tablet to capsule or the tablet changes colour, they could be changed to a different manufacturer, you know, you have to think of that as well. So that does have a knock on effect to safety. So I think you have to just be careful and mindful how you do switches. So yes, and I think that's why it's important to keep on top of what's in there, in your (the CDS) profile and keep reviewing it.

CCG Pharmacist 1
We'd asked for some modifications and refinements (to the previous system), and it became clear after a while that they weren't up for actually doing anything. So the message was always well, you're just you, lots of people like it as it is, but we wanted ultimately something that did more than just make financial recommendations. We wanted something that had a lot more functionality in terms of NICE recommendations, safety messages and HRA guidance, et cetera. It became clear that (a previous system) people just didn't intend to make that change. They saw theirs as a sort of self-contained unique system which has its own potential attributes and they were just basically sticking with it.

CCG Pharmacist 3
R: Yes, but the thing is is that we're professionals first and foremost, but often we get waylaid with the day-to-day jobs and the priorities. So for instance, our focus at the moment is on cost-savings. But it's almost like, how can you balance that professional head of patient safety with your cost-saving, so yes, we've all got it. It's important to us but it moves in priority depending on what is happening currently. But that's what we are, we're professionals, we're here to take care of patients, to ensure that they get the best, but we have got limited resources. So we've got to use it wisely.

CCG Pharmacist 3
I: What's going to happen in two years' time?

R: Yes, I think the amount of cost-saving we're going to be getting out of it is going to go down and down so I think there probably will be more focus on the safety, best practice messages.

CCG Pharmacist 3

R: Absolutely. I mean it's very much embedded, it is actually a very specific [inaudible 0:10:56] [line 0:10:57] which gets reported on every month for the CCG, so we class it as like a significant aspect of the work which we actually do, and one of the ways in which we not only implement the safety aspect but also monitor saving as well.
I: So from a CCG point of view is that safety – because I mean we talked about safety and cost last time – is that safety still the priority, the big main thing? You talked about it as a big selling point last time.
R: From the CCG point of view, because of the financial situation we're in the cost is the priority.

CCG Pharmacist 5
I: Yes, or the whole package, the programme. Is it there to be a safety net?

R: And a cost-saving tool. I think if there wasn't a cost-saving attached to it, the CCG wouldn't fund it. I think if it just sold itself on safety, then I don't think the CCGs would invest in it. I know that sounds really bad but the way that the finances are at the moment, they can't afford to be investing in things that aren't going to show a return on investment. So, it's just fortunate that it can show a return on investment so that then we can invest in the safety side of it. […] So as a pharmacist, I think it's there for safety but practicality wise and funding wise, the CCG would say, it's there for cost-saving.

CCG Pharmacist 5
R: So, I think they can see the value of the safety messages. We've certainly not had any GPs, at least not in (name of place), turn around and say, we're not using this because it's too annoying. I think even they will accept that the safety side of it is worth putting up with the annoyance of the cost-saving messages.

CCG Pharmacist 5
R: Oh, absolutely. And I mean it's not unusual, so it's what happens within CCGs or within the NHS; so next year we are going to have a more uphill task getting approval for funds to come to you to have it. And it gets more difficult each year. […] At the moment, when you are facing a deficit and you've been told that you have to save a specific amount of money, and you also undergoing the restructure, I think it's a credit that we've actually managed to get it approved.

CCG Pharmacist 5
I: Right. So, there wouldn't be any future planning over the profile, would there? Or would there be...?
R: There might be discussion about it, but that would be more, sort of, an operational issue about what sort of messages we wanted to have on and off. I think the CCG are obviously more interested in cost savings, so we need to make sure that the messages we have switched on are delivering cost savings to allow the CCG to keep funding it.

CCG Pharmacist 5
I: I mean, what is that balance, is that balance still, is (the CDS) there for medication safety or is it there to save money?
R: I think it's there because it saves money, but the safety aspect is brilliant. And if it didn't save money, the CCGs wouldn't fund it at the moment. I think that they are that strapped for cash, if something doesn't save money they can't justify it, which is obviously, very, very short-sighted, but that's the situation they're in. I think if you'd asked me ten years ago, I think they would have funded it on safety grounds alone.
I: Just purely as a medication safety?
R: Yeah, absolutely. So, the thing that we need to do is try and put a cost saving on safety measures and that's the bit that we're trying to work on at the moment, how do you, sort of, say, okay yes, we've saved a life or we've saved a hospital admission, how much is that worth to the CCG? And if we can put a value on that, then we're more likely to get the safety aspect of it funded. The CCG obviously care about patients.
I: Yes, [voices overlap 00:07:56].
R: It's just the position that we're in at the moment, it's ridiculous, it's like we've got to save millions and the only way we can do it is by making short term savings, at the moment, and...

CCG Pharmacist 5
R: Yeah, and that's what we want to do as clinicians, we want to do the safety stuff, but if we have to prioritise the cost saving in order to be able to keep the safety stuff on the radar, then we will. So...

CCG Pharmacist 5
I: Yes, because in some respects, the sustainability of (the CDS) as a system then, is dependent upon that, because it won't get funded. But, does that also then impact upon, you know, the messages you put into that profile? Is it that you're then, sort of, thinking, oh, we need more cost saving measures, or is it...?
R: I honestly don't think it does. I think our priority as pharmacists working on it, I think the fact it's there, the cost saving messages, they're either relevant or there not, we will have them on or off, depending on whether or not they're going to save any money. So, that bit just happens and that's what the CCG likes, they're going to get their cost saving from that. But I think when we're working on it, we probably don't think, oh, I must put that message on because it's got this costs saving and I want to do this safety one, we'll turn it on if it's relevant. So, I think, from a strategy point of view, the cost saving is very important and we need to make sure it's delivering. But from a day to day, oh, here's a new message, do I turn it on or not? I don't think that really impacts a huge amount on decision making.

CCG Pharmacist 5
I: Yes, I mean, I can see that. But, you said that you feel that the safety ones, the cost saving ones are not getting a look. Because one of the things that I was thinking was that, from what we've been finding is that, you know, where this, you know, you're getting constant bombardment of cost saving, and often GPs will say, oh, it's just to save a penny. And...
R: That's when we try and limit the number of cost saving messages that it gets, so we try not to turn on the really low value ones. And yeah, I think possibly, the most valuable ones we perhaps wouldn't just do as a simple message, we'd put an explanation with it, so that you could put in the titles something like, massive cost saving all over it], I don't know. Yeah, but most of them are fairly average sorts of savings and they'll just accept it. So...

I: And the problem is that, you know, even if it is a 20p savings, you have to think, it's not just you doing it, is it? It's across a CCG, across the country.
R: Exactly.
I: Once you've done all of that though, if it's a very, very commonly prescribed drug, it's going to add up, isn't it?
R: Yeah. I think one of the problems with the cost saving messages is often that cost saving is done per dose, so it doesn't look that huge. Whereas, if they gave the cost saving per month or per year, I think that would have a bigger impact .
I: Oh yeah. So, if it's 10p per day, but they're taking it four times a day...
R: It would be like, oh that 10p...
I: ...and they're taking it four times a day and they're taking it all day.... for the next year, it starts getting to be a lot more.
R: Yeah, it's that...so I think that might be useful if they could, yeah, change the way they've...they might have done it already, but..

CCG Pharmacist 6
I: What sort of things drove things forward then in those early days of getting the system up and running, as it were?

R1: So when we had someone come and do a demo, I think there were two aspects for it for me, and one was the best practice messages and one was the costing-saving messages. Now it was very much sold on the best practice and safety, which was good, because it wasn't just focusing on the money, but it still had an aspect of the cost-saving element to it. So I think, for us, that was a big driver, and also the fact that a local CCG had it, and I think (name of place) had it as well, did they?

CCG Pharmacist 7 and CCG Pharmacy Technician 2
R1: So I think it's a mixture of cost efficiencies and quality/safety interventions.
I: In what sort of ways?
R1: So it's particularly where you've got high volume so you're needing to put a lot of sort of reminders in, and it's a useful way of doing that, you know, sort of via an automated manner, rather than trying to have a person in the practice going through all of that all of the time. So, you know, every time the prescription sort of presents itself there's a reminder.
R2: And all the safety alerts are updated automatically by (the CDS). So in terms of that that's kind of covered; that was one of the things we were a bit worried about but…
R1: Yeah, things come on and off so it's…so if there's…something was a cost efficiency and then there's a major change in price and it's not then it'll come off, or the MHRA stuff goes on automatically, doesn't it? So it's quite sort of good, in that it's more self-sustaining, it's not relying on us to do everything, you know.

CCG Pharmacist 7 and CCG Pharmacy Technician 2
R2: I think we have more safety messages on there and things that flag up, rather than it all being about cost, more than Script Switch was.
R1: Yeah, I think you're probably right; probably the balance is more towards sort of safety alerts, you know, safety or MHRA updates, that type of thing, probably.
R2: Safety…when things go wrong, safety has a cost anyway, doesn't it?
I: Well, yeah, absolutely, yeah precisely. So…
R1: I think as well maybe if you were in an area where you had a lot of, say, branded generics, you'd probably have a lot of cost stuff on, whereas we have some but we don't have a massive amount, so… Because if you're in costing it's going to be a very simple like, that to that without too much thinking. You can't say, oh well, that to that, and then do all this and do that and do that and do that. It's got to be, you know, like a straightforward, that is what you've chosen, that's…this is the choice.
I: This is cheaper, go for this one.
R2: Yeah.
R1: As I say we don't really do…we do some branded generics but not a massive amount, so probably that influences it as well. You know, you might find it different in a different area.
R2: And I think if you have too many switch to this, switch to that, switch to this, and oh no we're changing to that now…
R1: Yeah.
R2: …it gets very confusing and that can be unsafe as well. So what you're trying to achieve you could actually end up making something unsafe…
I: Because?
R2: …which is going to have a cost. You could have duplications on the screen for example, you know, someone forgot to take something off or… do you know, things like that can happen, which is…if you've got more of a human touch… If you had a technician looking at the screen it's a lot, I find, safer in a lot of ways. I think it has got its place but I don't think cost savings are the be all and end all. I think it's the systems that cause the problems that they work with, because I think everything can be over-ridden, any risk can be over-ridden all the time. It's not watertight and that's the problem.

<u>GP Staff</u>

GP Nurse 2
I: And in terms of, you know, we talked there about how it's used within your work, what do you think the system's there for? I mean the way I've phrased this question for people is, what do you think the point of having (the CDS) is?

R: So it would have to be safety and cost, yes.

I: Which of those is more important?

R: To me, safety, to the NHS, both. No, it's got to be both, hasn't it? It's got to be both, actually, yes. Not being cynical, it has to be both, doesn't it, because if you've got a clinician that messes up with the prescribing then the implication is for the patient and for the Trust, so yes, it's got to be both.

GP Nurse 3
I: What's the most important bit of those two, costs and safety?

R: Safety. […] I'm not particularly bothered about how much it costs. I'm not bothered about the cost. The safety is most important for everybody concerned.

GP Pharmacist 2
I think it's focussed more on cost. Because, definitely in re-authorisation, it seems to be cost that gets triggered.

GP Pharmacist 3
R: The purpose of (the CDS) – I think there are a couple of factors involved, number one is for I would have to say safety, prescribing safety number one. Number two, it's very close, is financial. Cost effective but yet clinically safe prescribing.
I: Yes.
R: But I would have to say out of the two, financial.

GP Pharmacist 3
I: That in itself is quite interesting. Just to focus a bit and building back and some of the things you say, you talk about alert fatigue and we talked a bit about safety and cost.
R: Yeah.
I: Do you see any safety alerts coming through (the CDS) at all? Can you remember any of them coming through?
R: Not recently.
I: So most of the time it's coming through as cost –savings?
R: Yeah. I can't think of any safety ones. I would have expected one for mirabegron that I did recently. And this MHRA alert for severe hypertension or hypertensive crisis.
I: Because some of the MHRA alerts should be embedded within it, I think.
R: I never saw it. And it's consultant led prescribing, not prescribing I do myself.
I: Right.
R: But no message popped up, and because I've run an audit previously on mirabegron and hypertension, I added it as a safety point to the prescription itself.

GP Pharmacist 3
I: Yeah. But you haven't, so yeah. But you are not seeing those safety indicators coming through or safety alerts coming through Optimise?
R: Valproate I've seen, that's one that comes to mind, I've not seen any others, not that I can recall.
I: That's interesting in itself and it's also interesting, do you think there should be more?
R: Yes. There should be. This practice where I work now the prescribing is very safe.
I: Yeah.
R: We both know that the average for an error in prescribing is probably everyone in eight or one in six. It shouldn't be that sort of figure.
I: Yeah.
R: And blind signing prescriptions at the end of the day isn't great because I've picked up a couple of errors every week, that would have gone through mind signed but I've picked up the prescription pile and actually looked at each one.
I: Yeah.
R: Much to the humour of the GPs. But actually, it saves them half an hour or an hour each week, through prescribing errors.
I: Yeah. So, this would be on reauthorisations?
R: Yeah. But we do need more safety messages in some sort of polite way through (the CDS).

GP3
R: And that's my concern with it. Certain things I'll just flick through in (the CDS) because it's not worth the effort or this potential argument or time that I'd have to spend with a patient to actually change things, and I make a judgement about that. But when it's a safety one it's really good, because you learn things. I pick up things and I think I didn't know that, and I think I'm fairly competent at interactions. And it'll flow past them. It'll be ones that you've perhaps not got time to just look at the 28 repeat medications as thoroughly as possible, and it will always pick up…one this week, the amlodipine and simvastatin that I might potentially missed if I hadn't just had a little reminder. So I like the safety reminders. They help me. The cost reminders don't.

GP3
R: Yeah. Much that it would be good if we can minimise pop ups. But I think for the safety ones I don't mind that at all. That's fine. I never object to a good safety pop up. Even the nonsteroidals, that's still a good one, even though it should be engrained in us by now. Occasionally you get the weird one that comes up and you think that's really interesting, I didn't know that.

GP3
Because that's the whole point. If I was perfect I wouldn't need anything. So no, these reminders that are both educational and just summing up perhaps we're all human, we make prescribing errors. It just reminds you. It keeps you straight. And occasionally I've said to patients I'm going to do this, and I'll go well, maybe that's not such a good idea. So yeah, some of them, particularly the cardiac arrhythmia ones, because there's so many newer stuff that's on there that affects QT and all these other things that even five years ago wouldn't have been on [voices overlap 0:31:58].

GP3
I: Yes. You talked a little bit earlier about this. Do you find the alerts are relevant and useful to you?

R: The safety ones, I think they're always very good. It goes back to me saying sometimes the cost saving ones are…the switchy ones I'm just not that bothered about, and I would have dialled them out anyway. I've seen them once, that's it. I don't want to see them every time I do this. Medicines management will kill me when I say that, but they don't have to do my job.

GP3
R: But I'll be honest and say the pharmacists in the past, the medicines management pharmacists, they're so QIPP and cost savings obsessed. Not even cost savings, I'd say demonstrable, identifiable cost saving obsessed, that they sometimes are counterproductive, and they're not as safety led as we are. Among other things we have regular significant events meetings about either drug errors or drug problems or drug interactions, and myself and Doctor [name 0:08:48] used to be the prescriber on JPAG many years ago, and we'd almost have them on a daily basis. That's what we do. We were just pharmacology nerds.

GP3
I: Yeah. I didn't do it, yeah. A question I've asked quite a lot of people is what do you think (the CDS) is there for? What do you think the point of (the CDS) is?

R: I think it should be safety. But unfortunately I think it's tagged on to generating quantifiable savings, and I don't like that, because I think just because they're quantifiable doesn't mean they're savings […] As a clinician I just think we've got a big pot and just because we can measure it doesn't mean it's not coming out of somewhere else.

GP3
I: So the cost reminders are more niggling than…??

R: Again, they are. I know the policy, if I decide that I'm not going to follow the guidelines, and they are guidelines, so be it, that's my responsibility. As a GP it's still fundamentally way under budget. I've shown that I'm a competent prescriber. So I do get niggled by it a bit, and there's certain ones that I understand why they say that they're not cost effective and things like that, but I'm sometimes not prescribing them for being cost effective, I'm prescribing them for overall health budget. So rather than refer someone or ask someone to come back I'm doing no harm is the way I'm looking at it, and I might be prescribing something that cost £2.16 rather than £2.05, but I'll live with that.

GP4
R: I don't think cost is irrelevant. The reason I get frustrated about cost is because it changes so much. And there's a real short-termism about budgets. And this idea that right, this year, we're going to change your patients from this drug to this drug because there's a safety – not a safety thing – but there's a cost issue. But then two years down the line that will have changed, and that drug has come off patent or there's now a new formulary kid on the block that's the new statin or the new inhaler or whatever. And that I find frustrating because you feel you're putting in effort and we all work in a system where we have to look for cost savings and be aware that we're spending taxpayers' money and that we can't just ignore that, but on the other hand it doesn't always feel like there's a consistent long-term strategy that we're moving people in an organised way towards the more cost-effective prescribing strategy and that it will stay like that. You know it's going to be changing in the next year. The inhaler you prescribed this year is no longer the cheapest, so… So that frustrates me about the cost aspect, but I don't mind it in principle. I don't think we should be immune from that. I think GPs should prescribe cost-effectively if they can. And the safety thing, yeah, I can't really argue with that.

GP9
R: I still see it more as cost-saving.
I: Right.
R: I think the safety thing comes up on a different screen prior to that.
I: Right, which system are you on, (GP CLINICAL SYSTEM)?
R: (GP clinical system).
I: (GP clinical system), right, okay.
R: So the safety bit comes up when you actually prescribe it, not the (the CDS) bit so I use (the CDS) as the cost-saving bit.
I: Right, so you're not seeing safety messages in Optimise.
R: I think there are a few, aren't there, sometimes with statins and clarithromycins so, yes, but…
I: Yes, but by and large you're seeing…
R: They've come before anyway.

GP10
R1: So, I think, (removed potentially identifiable material) but the cost side is probably more important on a day to day basis, than the safety side. But it's nice to know that, we sort of have a mix now, with the (GP CLINICAL SYSTEM) flags up every possible little safety problem, but if (GP CLINICAL SYSTEM) and (the CDS) agree, then you probably really ought to look at that.

GP10
I: And we talked earlier about the balance between cost and safety. And how, you know, you said most of the alerts you get are cost. Do you think the balance should be more on safety do you think, or on cost, or what's the best use of something like this sort of decision support system?

R1: It depends who your customer is, I suppose […] The real customer of (the CDS) is the CCG, at the moment, isn't it? […] So they want it for cost, so I guess if they're going to buy it for cost and have the safety stuff for nothing, that's great. I suspect that if the NHS had limitless prescribing budgets, (the CDS) wouldn't exist. So, I think it's there because of the cost, but the real positive affect of it is probably the safety, so you can hijack, you know…

GP11

R: Yes, I think more on the… for me I think for me there's a distinction between safety ones and cost-effective ones and I think it's useful to know the cost-effective ones and knowing when something is ill advisable from an NHS point of view. But I think this is something that the different members of the GP principals would vary on. And I think even out of the cost effectiveness ones a couple of the partnership would follow those relatively absolutely and then I think a couple of others, probably myself included, would be more inclined to say - if it's clinically effective then, fine, and have a lower threshold to override.

GP14
R: Yeah, so, I think from my perspective it's probably two-fold. It's to alert you really, if there are any major safety issues with the drug that you are essentially trying to prescribe, so, the fact it, sort of, links in to medical records and their current medication history as well.
It will pick up if you try and prescribe some Penicillin to somebody who's Penicillin allergic and maybe it hadn't just occurred to you. So, that's the one side of things which is really important. But then also being the prescriber I'm really keen for cost effective prescribing, so, there's the alternatives that sometimes flash up, have you considered prescribing this instead of this.
It gives you that option to rethink actually and think, well, actually, that drug's basically the same but cheaper and not going to do any harm, so, that's a perfectly reasonable suggestion, I'll do that. I still learn stuff off it, and I always encourage my registrars who are training in the practice as well to, you know, for cost effective prescribing as well.

GP14
I: Yeah. I'll come back to that. You said something there that was interesting. Is that a general practice wide view, do you think or is that, do you have a specific take as prescribing (lead) ?
R: No, I think it's a…as a practice, I think we are all fairly similarly minded doctors here, particularly the partners, but the salary and the registrars as well. We all try our best for good quality, safe and cost effective prescribing. We are quite big prescribers in the local area, we have a lot of patients with a lot of medical problems, complex ones. […] we are always fairly above budget when it comes to prescribing budgets and stuff. Which is what's sometimes frustrating because I think we are quite on it compared to some other practices. I think it's just very challenging for a set of patients to [voices overlap 05:02].

GP14
I: Yeah. We'll get on to safety and cost as well because you talked there about the, you know, you get the two different messages. You get, change this to another cheaper brand. Which do you think is the more valuable, which is the more useful out of those two things in terms of the way the system is used?
R: I get it. Depends on where you, where you lie with things from your own perspective, but for me personally it's got to be patient safety. Because if that can stop you prescribing something that would do the patient harm, that otherwise wouldn't have flashed up to you, that's got to be worth its weight in gold from my perspective, because there's a lot of patients who become ill, die, admitted to hospital because of prescribing mistakes or oversights or whatever. So, that's got to be the number one from my point of

<u>Sub-theme 1c:Value</u>
Software developer staff 1
With my old CCG hat on, because I used to be a CCG pharmacist in the (name of place), area and in (name of place), cost, always cost. And it's the one thing that we try not to focus on, but we can't not because every financial year, the financial teams will come along and say, right, so medicines management, we want you to save 1.5 million this year. And sort of it's the one place that finance know that probably we can try and get savings, other than cutting services. Because that's all CCGs do to save money is cut this service and cut that and make few redundancies. And for the medicines management team, it's right, what can we do cheaper. It's getting harder and harder now because there are no what we call big wins anymore, you can't switch all to one brand and expect to save £750,000. So, by adding…we can send pharmacists and technicians out to do that switch work but even better, why can't we switch someone at the point of prescribing when they first started, so the technicians could be doing other things like care home work where there are huge savings to be made, you know, direct…it allows medicines management team to direct the work to what I consider to be more important than going in and doing switch work. And our software can do that, it frees up that workforce to go out and review elderly patients in care homes or help GPs review asthmatics or COPDs or diabetics. And to do more quality work rather than…because, I mean, gosh, there are even companies out there that just do switch work, like medicines management solutions or something, it's all they do.

Software developer staff 1
We will see our customers we say about four times a year. We do have some customers, one north west of here, that want to be seen twice a year, don't want to see us four times a year, it's like there's no need, we're fine, is it getting its money? Yeah, you know, that is fine by them. We have other customers that would love to see us far more regularly or want more touchpoints if they're having any issues or whatever.

Software developer staff 1
R: Yeah, so there'll be…it's different, I think if it's a tag they'll definitely do, guidance they might tend to localise a little bit as well. But I don't think these days…the trouble is, I'm worried that people are just swinging more back to cost than best practice, which is really…not really disappointing, it's just a bit disappointing. And now…and everyone focuses differently, so all I hear now at QRMs is, I don't hear about things about safety indicators, drugs of low clinical value, that is all I'm hearing, you know, the new NHS England…

I: And is that coming from CCG or from GPs, who…?

R: That's coming from powers that be that fed down to CCG, so NHS England and all of that. Obviously, I mean, the biggest player in all this as well now is (a previous system), or (a previous system), whatever you want to call it. I think really because it's so cheap to sign up, I think nearly every NHS organisation's now signed up to those guys. And they will take…they will create messages, and that's all…it does have some quality about it, but it's more about cost effective alternatives.

Software developer staff 2
But I also used to work for a competitor company, which you're probably aware of, called xxxxxxx who had brought to market a solution that did very simple swap messages, if you like or provided pieces of information to prescribers. I guess they sort of developed a market really, because

people didn't have software until they'd purchased this product, and then a number of CCGs took that on. Initially that worked extremely well. At that time, eight, ten years ago, it was very simple to make significant savings to a prescribing budget where largely GPs had complete free reign really over what was prescribed. And then we saw PCTs, I guess, in those days, develop formularies and start to control GP prescribing. For me, (the CDS) took that to another level in that it could interrogate the patient record further rather than just depend on what was prescribed, it could look at other parameters within the patient record, making it more intelligent as such, and address a lot of the concerns, I guess, that GPs had from using the previous solution from the other company, xxxxxx in that it reduced alert fatigue, it was more targeted and directed. And I believe that when (the CDS) was first introduced, whether this is just a company myth, was that it was developed on best practice and safety, and it was actually the market that told them that they needed cost saving element as well.

Software developer staff 2

R: That's a very good question, and the reason I had to postpone this interview is because I was up in a practice doing (an audit and feedback product) this week, and we've done our first activation. I think the answer to that question is we know it's a question, but we don't actually have the answer. I have always held the view, and I used to work at another company that had a similar product [inaudible 0:13:53], and I had the same view there, that really the most impact that a system that pops up an alert, is actually going to be at the initial prescribing of something. Whether that be acute or starting a drug that's going to be on repeat and long term. Because at that stage the patient hasn't had it, it's easy to change your route slightly, you haven't to communicate anything particularly to the patient because you were only starting it then. So I suspect our acceptance and the majority of our effectiveness is at that stage. (the CDS) also fires the repeat re-authorisation on medication review. However, we learned from practices that the repeat prescribing process is not as textbook as we'd imagined, and that patients are often not present at reauthorisation. So therefore, if something pops up and you want to change it there's actually quite a lot of work around that in terms of if I just change it they might come back and query it [voices overlap 0:15:10]…

<u>**CCG staff**</u>

CCG Pharmacist 2

I think, predominantly, obviously on everyone's mind in the CCGs is the finance aspect isn't it, so the cost saving, that's what comes across, first and foremost I guess, is like what's the most cost effective option. Behind all of that is the evidence base and everything as well, that came across, it's based on NICE, it's based on Public Health England's information, or anything to do with National Guidance, and what they recommend, any alerts that come up would recommend anything to do with those, but obviously I think, I can't remember how they said it was going to fit in with our formulary, but I think there is a way of turning things on and off?

CCG Pharmacist 2

Obviously, GP practices are going to be over the moon about saving money where they can as well, for their own businesses sake and how well they perform from the CCG perspective.

CCG Pharmacist 2

The reports we have seen, tend to be more like finance related, like, in this past quarter, we've saved so much, or, this practice has done really well in this, and (the CDS) has been shown to help that happen.

R: And to a degree and then also the fact that the practices didn't have to pay for it. If they'd had to pay for it, it might have been a different kettle of fish. And they were also very concerned about what would happen after the first year and what would happen after the second year. Would they be asked to pay for it in the future? And I wasn't able to say categorically no or categorically yes. And I was very honest with them. I said, I do not know. At this particular point in time, we can pay for it, but going forward, I cannot guarantee that. So I think they actually appreciated that honesty.

CCG Pharmacist 3

R: And to a degree and then also the fact that the practices didn't have to pay for it. If they'd had to pay for it, it might have been a different kettle of fish. And they were also very concerned about what would happen after the first year and what would happen after the second year. Would they be asked to pay for it in the future? And I wasn't able to say categorically no or categorically yes. And I was very honest with them. I said, I do not know. At this particular point in time, we can pay for it, but going forward, I cannot guarantee that. So I think they actually appreciated that honesty.

CCG Pharmacist 3

R With the second (GP CLINICAL SYSTEM) practice, which is within my patch, they don't feel they need it.

I: Oh, right. Interesting. Yeah.

R: They don't feel they need it, they feel that they're very good prescribers, and as a result of not having (the CDS)for several months it hasn't caused any detriment to the practice, so therefore they do not want to have it. So they haven't got it activated. The other practice, which is the System One practice that has deactivated it, we are working with them at the moment, they're back on doing a trial; again they feel that there are too many messages coming up.

CCG Pharmacist 3

R: Absolutely. I mean it's very much embedded, it is actually a very specific [inaudible 0:10:56] [line 0:10:57] which gets reported on every month for the CCG, so we class it as like a significant aspect of the work which we actually do, and one of the ways in which we not only implement the safety aspect but also monitor saving as well.

I: So from a CCG point of view is that safety – because I mean we talked about safety and cost last time – is that safety still the priority, the big main thing? You talked about it as a big selling point last time.

R: From the CCG point of view, because of the financial situation we're in the cost is the priority.

CCG Pharmacist 3

R: Oh, absolutely. And I mean it's not unusual, so it's what happens within CCGs or within the NHS; so next year we are going to have a more uphill task getting approval for funds to come to you to have it. And it gets more difficult each year.

I: Yeah.

R: At the moment, when you are facing a deficit and you've been told that you have to save a specific amount of money, and you also undergoing the restructure, I think it's a credit that we've actually managed to get it approved.

CCG Pharmacist 3
R: Why do we continue to use (the CDS) within the CCG? One of the reasons I think is we have definitely won the argument around safety and efficacy; so I think within my department that is well understood. I think because the practices value it.
I: Yeah.
R: And I think we've been able to put forward a compelling case of the usefulness of it, even with all the financial constraints that we have.

CCG Pharmacist 3
I: Right. That's interesting, isn't it, because I've heard that before, where some of the messaging is being altered, as it were, by yourselves at the CCGs, rather than at FDB.
R: Yeah.
I: So a sort of decentralisation of it, isn't it, which is quite interesting.
R: [Voices overlap 0:28:14] very enthusiastic about, personally I'm not enthusiastic about it.
I: Right. Why?
R: Because I feel one of the reasons that we went for (the CDS)was for that level of peer support and safety; and I feel that things done across a wider network with more people involved there is less room for error.
I: Right. Okay. So because it goes wider, that safety aspect is being further taken away, it's being more…
R: Well, I think it's also being the responsibility is being shared, so like for instance, whereas we would make…two or three of us would get together and say we want this message on for a reason, because we'd have to send it to (the CDS) to author, they would do a double check against national guidance, they would also look at the messages that were already on the clinical system, and because they have specific criteria for authorising messages I feel that's effectively a third check; and there are some messages that they were not authored for a particular reason, it does make us think again. And there are some cases where we insist that, yes, we do want it on our profile and they will do it just for us, but I just feel that it's a bit of a safety net, and I don't think when you're coming to people's health you can always be too safe.
I: No. No, precisely. Precisely.
R: And having other expertise involved, and it can only be of a benefit; and I worry about that if we are doing all these things ourselves. Because if we're doing all these things ourselves how much of it could we do ourselves? Do we need to have Optimise? Okay, I mean I know that the technical aspect, and that's why I'm a little concerned, it's kind of like watch this space carefully.

CCG Pharmacist 5
R: So as a pharmacist, I think it's there for safety but practicality wise and funding wise, the CCG would say, it's there for cost-saving.

I: Yes, I mean I know someone had previously said to me, yes, as a pharmacist, with my pharmacist hat on, it's safety but I'm sure there are accountants who think it's much more important, a cost-saving.

R: Yes, [voices overlapping 08:34] money.

I: Do you think that's a perception shared widely or is that a perception that is…?

R: From speaking to GPs and the pharmacists that deal with the GPs, I think the perception on GPs is that it's cost-saving, which is a shame.

I: Why do you think that is?

R: I think that because that's probably the majority of the messages that they see is cost-saving and they're probably the ones that annoy them the most and so they're the ones that they remember.

CCG Pharmacist 5
I: I mean, what is that balance, is that balance still, is (the CDS) there for medication safety or is it there to save money?
R: I think it's there because it saves money, but the safety aspect is brilliant. And if it didn't save money, the CCGs wouldn't fund it at the moment. I think that they are that strapped for cash, if something doesn't save money they can't justify it, which is obviously, very, very short-sighted, but that's the situation they're in. I think if you'd asked me ten years ago, I think they would have funded it on safety grounds alone.
I: Just purely as a medication safety?
R: Yeah, absolutely. So, the thing that we need to do is try and put a cost saving on safety measures and that's the bit that we're trying to work on at the moment, how do you, sort of, say, okay yes, we've saved a life or we've saved a hospital admission, how much is that worth to the CCG? And if we can put a value on that, then we're more likely to get the safety aspect of it funded. The CCG obviously care about patients.
I: Yes, [voices overlap 00:07:56].
R: It's just the position that we're in at the moment, it's ridiculous, it's like we've got to save millions and the only way we can do it is by making short term savings, at the moment, and...

CCG Pharmacist 5
I: I think, probably last time, I'm going to round up on this, but I think we've...I'm like, throwing some questions out there really, it's just, sort of, it's a follow-up, and I think we probably touched on it in our last interview, I think the value of having something like (the CDS) going forward, what is that value? What's it going to do for patients and...?
R: For me, it's the ability to get a message out to all prescribers, even if they don't engage with the CCG, they are going to see that message. So, I think, yeah, cost saving, great, if they accept those, brilliant. But, for the safety ones, that message will definitely get seen at some point by all the prescribers if that patient needs it. And I think that's something we can't do, if somebody doesn't want to engage with us, they won't. Whereas at least we're giving them the chance to [voices overlap 00:35:46].
I: So, they've at least got the information there.

R: Yeah. So, that's from the safety point of view. From the cost saving and QIPP work, it saves us an immense amount of time because the GPs are doing it themselves and it's quick, and they can do it while the patient's there, they can have a quick chat to the patient and it's sorted. Whereas, if we have to go in, do an audit, change it, it's quite time consuming, so it's...

CCG Pharmacist 5

I: Thinking, sort of, of other things, you've got these structural changes with, you know, you're now becoming...across six CCGs you're becoming one CCG. Are there any other, sort of, structural changes, like PCNs coming on board which you think are going to make an impact upon it at all?

R: I'm not sure it will actually...so the PCN staff coming on board, I think, will benefit enormously from Optimise, and I don't think we've really sorted out our working relationships with them yet, to get messages out to them and give them updates on what to change on the formulary and all that yet. So, (the CDS) is a really fantastic way of making sure that they are actually getting those messages. Other than PCNs I can't really think of any other structural changes that are going to have a big impact, really. But, it doesn't matter how many people are using the system, it doesn't change the amount of work we've got to do on it. That's basically...

I: No, it's just, I was thinking more...

R: ...the beauty of it.

CCG Pharmacist 6

I: Thinking of pharmacists, does having (the CDS) mean […] does it mean you need more pharmacist input into practice, or less, or does it change the way that the pharmacists are used in practice? […]

R1: …what you'll find is the pharmacists are actually doing what the pharmacists should be doing, and not answering queries that they don't need to be answering. So an ideal situation, if (the CDS) wasn't there, an ideal situation from a formulary drugs point of view, would be they'd go and look at the APC website, but those queries will come into the pharmacist. But now (the CDS) is there, those queries don't come, which frees up the pharmacists' time to do something more clinical or something more helpful to either the CCG or the practice. So it's not that you can do away with the pharmacist, it's just actually you're getting more value for money from the pharmacist, because you're not...

<u>GP Staff</u>

GP Pharmacist 3

I: Is that a sort of widely held view, do you think across your colleagues?

R: Yeah, they don't see it as a piece of bolt on software that's just been added on they do see it as a legitimate piece of software to use with their prescribing.

I: Yeah.

R: And it's come from a good source.

GP1

R: It's quite a long time ago, I think it was through the medicines optimisation. We had a previous software, I can't remember what else it was called any more; it was another CDS.

I: Yeah, another sort of CDS system.

R: The guys had looked at (the CDS); I think it was cheaper, and that they felt it was giving us what…I think there'd been a problem with the other system, that they weren't updating it brilliantly; they'd put the price up and then they were wanting a commitment that they weren't willing to make, so they looked at (the CDS) system and decided it was pretty much the same. I think, I can't remember now, there was one very positive thing in its favour as well, which I can't remember what that was now.

GP3

R: But I'll be honest and say the pharmacists in the past, the medicines management pharmacists, they're so QIPP and cost savings obsessed. Not even cost savings, I'd say demonstrable, identifiable cost saving obsessed, that they sometimes are counterproductive, and they're not as safety led as we are. Among other things we have regular significant events meetings about either drug errors or drug problems or drug interactions, and myself and Doctor [name 0:08:48] used to be the prescriber on JPAG many years ago, and we'd almost have them on a daily basis. That's what we do. We were just pharmacology nerds.

GP3

R: Again, they are. I know the policy, if I decide that I'm not going to follow the guidelines, and they are guidelines, so be it, that's my responsibility. As a GP it's still fundamentally way under budget. I've shown that I'm a competent prescriber. So I do get niggled by it a bit, and there's certain ones that I understand why they say that they're not cost effective and things like that, but I'm sometimes not prescribing them for being cost effective, I'm prescribing them for overall health budget. So rather than refer someone or ask someone to come back I'm doing no harm is the way I'm looking at it, and I might be prescribing something that cost £2.16 rather than £2.05, but I'll live with that.

GP3

I: So can you tell me how, firstly, when (the CDS) came here and how it was introduced into the practice?

R: (The CDS) came from the CCG led, the medicines management led, to try and I think originally, not from a safety point of view, I think it's from a cost savings and QIPP point of view. And we did resist it a little bit at the start because life's complicated enough sometimes, and then it was almost mandated on us. So we didn't resist then when they said that.

GP10

R2: I think…I suppose final thing is that when it initially came, it did appear to be…I'm quite Luddite, so yeah, just bear with me on this thing. But equally I do embrace change. It did appear quite clunky and quite intrusive. It's become significantly less, so whatever the reasons, I suppose it's very hard to say, and got to the point where it strikes quite a nice balance in my opinion between giving me enough information regarding

safety and patient education and cost-effectiveness. Again, it's just getting on with the work that I've got to do, i.e. I've got a set amount of work in that day, so I don't want to be spending just my time [voices overlap 36:18].

GP12
So my understanding was that it came through the CCG, that the CCG decided to, and I guess I was at the meetings so I remember it being discussed and the potential of whether they buy it or not and whether it was going to be worthwhile. I think mostly the discussions I remember were more about the finances and how much money could be saved with that, and I think originally what I recall was that I think that CCG pharmacists were slightly reluctant to bring it in because of it being perhaps that some systems which had worked like this (elsewhere) had been an irritation (elsewhere), there had been negative feedback on those and the GPs hadn't liked them and things, so there was discussion as to whether this was, it would be worthwhile and would it really make the savings that people originally thought it would make or it was supposed to make. So I got the impression that we were a bit late, the CCG was a bit late or reluctant to bring it in, but then with the pressures of the finances and having to make savings, the decision was okay, well we'll have to go for something.

GP13
(the CDS), I think, does that less often, maybe, I think in the last month, I think (the CDS) might have annoyed me once. And (GP CLINICAL SYSTEM) might have annoyed me, or in fact, I might have annoyed (GP CLINICAL SYSTEM) reports, much more than that. So, (the CDS) has a better hit rate.

I: Because it's not interrupting things as much?

R1: Yes, but it also seems to be better targeted. The thing that annoyed me recently, was when it was obviously a CCG driven thing, 'cause it was a budgetary one, you should prescribe this brand, and you'll save 1p. And you just think, why are we…? But I don't think that's an (the CDS) problem, I think that's the people at the CCG who are populating it. I have no idea how it works really. I assume there's someone at the CCG typing all these things into it, and telling it what to do.

GP13
I: And we talked earlier about the balance between cost and safety. And how, you know, you said most of the alerts you get are cost. Do you think the balance should be more on safety do you think, or on cost, or what's the best use of something like this sort of decision support system?

R1: It depends on who your customer is, I suppose. […] The real customer of (the CDS) is the CCG, at the moment, isn't it? […] So they want it for cost, so I guess if they're going to buy it for cost and have the safety stuff for nothing, that's great. I suspect that if the NHS had limitless prescribing budgets, (the CDS) wouldn't exist. So, I think it's there because of the cost, but the real positive affect of it is probably the safety, so you can hijack, you know…

GP13
R1: So, it's probably about 50/50 'cause I'm the one, well actually I'm not anymore, but when I was the one that had to go to the meetings and justify our prescribing budget, it was nice to know that we were managing, we were getting the right brand of pill, and we were getting…that was nice to know that, because I could say, we're over-budget, but not because we're being rubbish, we're over-budget because the budget isn't big enough, look we're doing everything (the CDS) tells us to do, what more can we do? So, it's quite supportive from that point of view.

I: Actually that's quite interesting, 'cause that provides you with ammunition, as it were, or data, on your prescribing, yeah, we're doing everything we're told to do, we've made all the switches.

R1: I think I said in a meeting, what more can we do? If we're doing all that, what more can we do? And it sort of reminded the CCG that we could be at a point where we were actually reducing the quality of care to hit the budgets, rather than…[…] Yeah, rather than just improving cost effectiveness.

GP14
I: Yes, precisely. Does it…in which case when you're…in fact, I can…this issue around multiple morbidity and polypharmacies and which has been raised by other prescribers and, I've had two different messages. One which is said, well, actually that's where (the CDS)is quite useful. Others have said, (the CDS) is useful for the one of acute and actually when you've got a polypharmacy patient, you've got… it's far too much to deal with and there's too many alerts happening and, to be honest, it's just confusing the picture. So, which of those do you think is…?

R: I mean, the first one. I think you're better getting the alerts and be aware of it and make your own decision to ignore or cancel it, because these are just suggestions, they're not…you don't have to follow through with it. But from my personal point of view, particular from a safety aspect, I would rather know even if it's flashing up loads and ignore them all, if I'm going to take that responsibility and carry on with prescribing whatever that job might be. I would personally rather know but I can see there's two different angles from it in a sense isn't there?

<u>**Theme 2: How to implement (the CDS) : The processes involved in change**</u>

<u>**Sub-Theme 2i: Getting used to the system - the implementation journey.**</u>

<u>**Software developer staff**</u>
Software developer staff 1
…they may decide to pilot. It doesn't matter what size organisation, we tend to only pilot in four practices. And we tried to say get practices with prescribing leads, vocal GPs, someone that will either have a positive…something to say about it. There's nothing worse than piloting in a practice that is just so very quiet. Was it good? Much like, you know, thinking about what you were saying before, yeah, it's alright, brilliant. Do you like it? Yeah, it's alright. How do you think it compared to everything else? About the same. You don't want that, you want someone to have some feedback, whether it be positive or negative.

Software developer staff 2
….they are all different implementations, although there is probably a lot of overlap, but we are not very good at the moment at understanding the commonality. I think that might be something to look at in the future. In terms of implementation, with a customer that's up and running it's not so much of a challenge, we've got the tools in place to allow them to have that flexibility of introducing like the formulary tool this year to give them more control. In latter years it's been designed, I think perhaps it's surprised them initially how much divergence would be required. Yeah, so that's not too much of a problem. In terms of bringing on a new customer, which obviously that's slowed down and we don't have quite so many of those, but if they've come from the existing system, getting them up to performance on (the CDS) does take quite a bit of work on their side and ours because it's matching what we've got in the national sector and what their priorities are, et cetera. But it's by no means impossible. Have I answered the question?

<u>CCG Staff</u>
CCG Pharmacist 3
R: Let's go with what could have gone better. I think what happens is that we have a prescribing group meeting every month and due to the workload, it was getting it on the agenda and getting the time – dedicated time – because there was no point having a five-minute presentation. And then also, as what always happens, other priorities come along. ut also at the time I was running two big things because I wanted to put this in to aid with the formulary, but I was rearranging the way that we did the formulary as well at the time. So that was a big thing and that involved our internal staff members and thinking back to the fact that not so long before we had had two organisations that had come together that worked very differently. So that was quite challenging, so I was having to manage that at the same time as trying to introduce (the CDS).

CCG Pharmacist 3
R: Tenacity. Ensuring that I had got all the relevant stakeholders engaged. And then also being able to identify in the finance, but it did involve quite a lot of meetings, questions, reports, and I know we weren't one of the first to come on but the (neighbouring CCG) had already gone on, so it was worth… So it was waiting to see what they had come up with, because that was nearer home.

CCG Pharmacist 3
R: Making sure that practices felt involved, and it wasn't something that was being done to them. hat they had a choice but that was [down there 17:53]. But I think what I have realised in this organisation, it's about establishing who holds the power. And I established quite early on that in this area it is the practice managers. So I went to them. We did the clinical bit with the prescribing leads, but then I went to the practice managers. Because they were key to getting their practices on. And because I involved them, I told them what we were doing and it was in a different focus from cost-saving, it was…the focus was on patient safety. And that was what I kept pushing.

CCG Pharmacist 3
And to a degree and then also the fact that the practices didn't have to pay for it. If they'd had to pay for it, it might have been a different kettle of fish. And they were also very concerned about what would happen after the first year and what would happen after the second year. Would they be asked to pay for it in the future? And I wasn't able to say categorically no or categorically yes. And I was very honest with them. I said, I do not know. At this particular point in time, we can pay for it, but going forward, I cannot guarantee that. So I think they actually appreciated that honesty.

CCG Pharmacist 3
R The main thing that has changed over the year, there was a problem with (GP CLINICAL SYSTEM) in particular.
I: Oh, right.
R: There were problems with…I can't remember the origins of the problems, but there was a fault with the software due to the change I think TPP and (GP CLINICAL SYSTEM) had made, so it did go down for some time, I think it was about the better part of a day. TPP went back online pretty quickly, but (GP CLINICAL SYSTEM) didn't; and we had to wait for an update of (GP CLINICAL SYSTEM) which took them about four months to implement, because even though it was an upgrade that was needed on (GP CLINICAL SYSTEM), which needed to implement (the CDS) software, they don't do updates for just this CDS.
I: Oh, right.
R: There's a regular update process for the whole clinical system, so they would have several things in there. And as they update they tend to roll it out, but they don't do everybody at the same time, they tend to roll it out to a small group of practices and then roll it out to more practices, so it's a phased approach. But as a result of problems that happened with other updates they didn't roll it out across all practices until in totality four months. As a result we had a couple of practices that switched off.

CCG Pharmacist 3
I mean last time you talked about people felt that things were slowing…it slowed the clinical system down; is that something which people still talk about then?
R: No, it's not. I think sometimes it's just getting used to certain things. There was that initial thing that, oh, anything else is going to slow it down; but no, that's not a complaint we've had recently. We have done an evaluation and a survey, and we working to try and improve things as much as we can from our end.

<u>GP staff</u>
GP Pharmacist 3
I: It was implemented very well; I certainly find it runs very nicely in the background, it's not as obtrusive as I've known it to be, so I think it's more honed in this area.
I: Right yes.
R: As far as I understand with the software itself, it runs different protocols and I think they've tuned the protocols a lot more finely here, rather than blanket recommendations upon every prescription you are trying to generate. Which is very nice. It works very well from what I find here.

GP1

I'm not sure somebody came in and showed us how, basically it was fairly similar to the previous system and it pops when you're prescribing with information, so it didn't really take a lot of getting used to, we were fairly au fait with what was going to be happening anyway.

GP4

R: Yeah, okay. Well, we are still using it. I suppose it's just become more embedded, so I am a little bit less aware of it, I suppose, it's become a normal part of the clinical system that I use. And I'm definitely aware of it still but I don't think, the comments I would have now haven't probably evolved a great deal, I don't think. I don't think there has been anything particularly new that I've discovered about it that I didn't know when we spoke last time.

GP6

R: I think it might have been (name) or somebody from the CCG might have come to one our neighbourhood meetings and told us it was happening. But I don't remember any kind of training and they said, it would be pretty much like it used to... There may genuinely have been a five minute... I'm just trying to rack my head because it actually was well over a year ago now I think we went over to Optimise. And I think somebody from medicines management may have briefly said, it will be rolled out, it's pretty self-explanatory, when this pops up it's pretty much like, and that was it. But then it's not a complicated...why would you. But we were just encouraged to actually go along. So if it came up with an option, to take the options.

GP12

R: My decision making, I didn't think we decided anything as a practice. We haven't discussed (the CDS) as a practice. We haven't got that far. We're still, you know, in the last month I think we still worked out how to handle prescriptions. And there's even some bit of actually prescribing that I still don't think I've got right. I don't understand why it doesn't print and things like this because (GP clinical system) prescribing is so complicated. We're still trying to negotiate that.
And I just feel that we haven't got the brain space to then take on another alert. (GP clinical system), you spend a lot more time just even to issue the prescription looking at the computer screen. What I noticed in (GP CLINICAL SYSTEM) is I spent a lot more time looking at the patient and would just do a couple of clicks and issue their prescription. Whereas now I find I'm looking at the screen for so much longer trying to get through it.

GP12

I: Last time we talked, one of the things that you focused upon was that from your perception (the CDS)was very much a cost saving package, because (GP CLINICAL SYSTEM) was sending a lot of the safety alerts, now that you're on (GP clinical system), is that the same sort of thing you see or you're just not seeing them because of all this?
R: Do you know, to be honest with you, I've absolutely no idea what I'm seeing.
I: Right. And is this getting used to the system or do you think this is a going to be...?
R: A long-term thing?
I: Yeah.
R: Interesting. I think what it seems to be long-term in (GP clinical system) is the number of clicks that you have to click on to issue a prescription. You don't just click on the drug at (GP CLINICAL SYSTEM), you just kind of clicked on the drug, and I think, you know, and it came out in the printer just like that. Whereas actually in (GP clinical system) you click on the drug, you then click to issue it, you then click to print it and it says this, that and the other and do you want this, that and the other and you click that ….. just so many more clicks on particular things of your screen. Now then you have to add (the CDS) on to that and you just end up thinking that's just too many clicks.

GP12

I: So do colleagues basically are feeling...
R: Do they feel the same, yeah, I think everybody feels it. I think that there also, I mean, you can in (GP clinical system) you can switch off the drug alerts. Now that we've found the place where user preferences can actually switch off loads of alerts, we're all sitting here, you know, is the decision which we are making in the practice is which alerts in (GP clinical system) are we going to switch off, which is safe to switch off and which aren't, we don't know, and it just, you know. I think one of the colleagues has switched off all of them because she actually thinks, you know. And it maybe that what we need to do is switch off all the (GP clinical system) ones and just use it if he CDS is going to give us better alerts but we haven't worked that… Nobody has advised us, nobody said, nobody from the CCG has said this is what you need to do if you want safe prescribing. It just sounds ridiculous. It sounds ridiculous that at the last partners meeting I'm saying, (name) [19:55] can you work out which alerts we should and shouldn't be using. If you're the national person for safety, and yet I don't even know in our practice what we, you know, what's safe anymore. And nobody seems to, you know, nobody said to us I don't know what other practices are doing, it's crazy.

GP14

I: When you first saw it then, what sort of, you know, what went well with those first initial uses of it?
R: So, I've always been pretty open to getting nice suggestions about how you can do things, A; either more safely or more cost effectively or whatever it might be, again we're going back to the fact that they've been around for a long time.
So, I think from my personal perspective having been a GP for a long time, I'm also the prescribing lead for the practice, so, I'm all very much for good quality prescribing and safe prescribing. So, for me it was a fairly smooth transition it was fairly self-intuitive as to what you did or didn't do in terms of accepting suggestions or not. For me it was pretty easy despite the lack of knowledge about when it had changed over or why.
I: So, it's interesting they were no conversations, CCG or anything from, just imposed in volume.
R: No, just, these things happen, that's not out of the ordinary, could I say. So, yeah things just happen. It's the same with new hospital services or new forms that we're meant to be using for referrals or end of life care. They often will appear before you have any training or knowledge of it whatsoever. Then if you're lucky maybe there's a launch event six months after you've already started using it. So, yeah, it didn't seem particularly well organised when this appeared but again, as I say sometimes there are extenuating circumstances, so, maybe I was away on a week where somebody came and talked about it, but yeah don't know.

<u>Sub-theme 2ii: Implementation - Direction of travel</u>

<u>Software developer staff</u>

Software developer staff 1
I: That sort of brings us onto what we can have as the final question. What do you think builds confidence in the system?

R: Being listened to. So, if a GP has fed back and something's been done about it, then that builds, because they just feel they're not being listened to. So, I know, you know, I think some of my customers, if I have feedback a particular message, they might contact me going, can we make this a bit more specific because it's triggering a bit too much, can we add in this logic. We certainly can, shall we do that. Brilliant, let's do that. Or shall we just turn it off. And again, even if it's more generic, so even if an MM team has not done that work, we will do it for them at QRM and we'll go, right, in the last three months you've had 12,500 messages hit, 5,000 of those are just to this one message, which is accepted one per cent of the time. That means we do a number of things. One, we turn that message off; they're seeing it, they're not doing anything about it. Two, do you need some education to your prescribers of why the message is there and why it should be listened to. Or three, do we change the logic so it appears less frequently, and what can we do about it. Because then, all of a sudden, that message stops appearing, the GPs think, oh, thank God that message isn't appearing anymore.

Software developer staff 1
But you'll get more engagement and there'll be better acceptability if the end user feels they've been listened to and gets that engagement. Which is why sometimes I think maybe doing a feedback or a survey with them would be useful, because then if they did something, we listened and we acted on it, they're like goodness gracious, I've actually been listened to. And GPs, yeah, they like that. But again, feeding back more. I really try and encourage MM teams to give each practice (the CDS) data, you know, these are your savings you've made, these are the best practice…you know, don't use it as a stick to hit them with, look at these 20 black drugs you've prescribed, what are you doing. That would just get their goat up. I know, I've been there many times.

Software developer staff 1
R: And actually, we thought we can't just do a standard pilot anymore, we need to engage more, and the people we need to engage more is with the GPs. Now whenever I've piloted, I will always visit the practice. Hello, my name's (name), I'm account manager and we are piloting some software. This is what it looks like, this is a demo, I'll either do a live demo or video. And answer any questions, this is when it will start, it will run for four weeks, it's very easy to turn on, if you've got any software, just turn it off. And answer any questions. It seems that when I was not here for the year, because the team were so run ragged, that wasn't necessarily done, but they've still obviously got the pilot. But we're now saying, if you're piloting, that is a must, you have to go to the practice and you have to…sometimes only a practice manager will turn up but that's no good, you need to get at least one of the GPs involved in that discussion as well, about the feedback.

Software developer staff 1
Because the thing with GPs is that they will listen peer to peer, they don't really like being directed down to. So if we can have someone that likes the software and is happy to go with it and be our sponsor within there, that often helps because then they can have at least a decent discussion with one of the GPs that piloted that didn't like it, because then they can say, why didn't you like it, because it popped up with any other…I mean, I've been in a meeting like that where, you know, oh, I ignored it because of this. And the prescriber [inaudible 0:43:53] why did you ignore that message, that is clinically unsafe for you to ignore that message. It's like, oh gosh, it's all got a little bit heated. But it was true, the prescriber [inaudible 0:44:02] was like, but why wouldn't you listen to that advice, you're sitting here telling me that a piece of advice came up and you ignored it. You know, he said that worries me a little bit as, you know, being your peer, sort of. And so it was, like…the other [inaudible 0:44:15] was just like, well, it's stuff and nonsense, and the prescribing lead said well, I don't believe, you know, it is.

<u>CCG Staff</u>
CCG Pharmacist 3
So they knew, the prescribing leads knew, so by the time I started introducing it, people were aware of what was happening, because often one of the problems that we have here, and I'm sure it happens in other organisations, is communication. And people complaining about not being told, that they don't know, so they were fully, fully aware. And that was the key to making sure that we had a successful implementation.

CCG Pharmacist 3
R: Well, it is active in the majority of practices, so we are still using it very much as we used it before with regular updates; so we action all the updates that come through from (the CDS), and we also add our own messages on. There are a few more facilities that FTB have implemented whereby we can actually do a few more things ourselves; and there are some new reports which they've put on.

CCG Pharmacist 6
Yeah, so initially, when we piloted it, we did a whole questionnaire on feedback. Yes, so a feedback form for the GPs to actually let us know how they felt. So I think it was setting the expectations from the outset was really important, so it was like, actually, do you know what? This tool is here as a pilot, it's not going to solve all your problems or issues that you have, it's going to be a bit...like there's going to be, you know, a few issues that you'll find, but bear with us because we'll work on them, and that's where your feedback is really important. Because otherwise, I think the GPs tend to switch things off because they think, I don't like this and I can't change it, and I don't own it.

CCG Pharmacist 7 and CCG Pharmacy Technician 2
I: And in terms of getting it into the…getting the practices on board to start with, how did that sort of go?
R1: I think it went fairly well because the thing is, they were used to having it because it's not like we went from nothing to that.
I: Yes, it was just changing across.
R1: We'd had Script Switch and we moved it over. So I don't think…it wasn't an alien concept to them, so therefore they were fairly kind of happy to have it. Nobody said, oh no, you're not putting that, and I'm not having that, you know. …But they were used to it and they had it a few years, you know, for sort of a messaging system.
R2: Didn't two surgeries highlight it as well – was it (names of practices) first?
R1: Yeah, we did a little bit of a pilot first of all, for them to trial it and that was sort of positive. So there was nothing that was so bad that…so they were all over it.

<u>GP Staff</u>

GP Pharmacist 1

R: Yes, we had information from our CCG pharmacist to tell us that it was coming and, you know, what the purpose of it was and what things it covered. I communicate with her on a regular basis so we knew it was coming but we didn't have to do anything physically to put it on there.

GP Pharmacist 1

R: It came from CCG directive, by medicines management.

I: Right. So, were there any decisions in practices whether to have it or what?

R: In this practice, I don't think there was any problem in having (the CDS) over the top. There have been discussions within the GPs themselves that they are having a bit of, what they call click fatigue, when they are trying to prescribe something and (GP clinical system) is throwing up its adverse drug reaction warnings, and then you have (the CDS) trying to switch the drug over to something else. […] That's the issue. Otherwise, it was accepted as part of the software to have to run and as part of the prescribing quality.

GP3

(the CDS) came from the CCG led, the medicines management led, to try and I think originally, not from a safety point of view, I think it's from a cost savings and QIPP point of view. And we did resist it a little bit at the start because life's complicated enough sometimes, and then it was almost mandated on us. So we didn't resist then when they said that.

GP4

Well, through (name of CCG Pharmacist). So Medicines Management, so she notified us that it was going to start, gave us a little bit of brief outline about it, gave us some email information about it to have a read of, just asked if we had any objections at practice to taking part, but I don't think there was an option anyway. The CCG had made that decision anyway. So yeah, she gave us a date when it was going to be started and off we went.

GP13

So, okay, I can't remember how long ago it was, but it must have been something like between two and three years, 18 months to three years, something like that. And it was, as far as I know, it was something the CCG, I don't know if they strictly required us to do, but there was a strong expectation that we would, we would give it a go and try it. We accepted it on the basis that it could improve patient safety, and it would help us achieve our prescribing budgets, which is…I think we're always aware of the dual purpose of it, but it felt like a prescribing budget thing when it was given to us, rather than a patient safety thing.

GP14

I: So, there we go, that's working nicely. So, can we start by talking about how (the CDS) was introduced to you at the practice here.

R: So, from my perspective, I don't know how long we've had it now altogether, but it just snuck on and appeared if I'm honest, so, there wasn't any grand trumpeting, you know, obviously, sometimes there can be reasons for that if you've been on holiday when something got introduced or something but it just started flashing up. We had previous incantations of it, of prescribing aids before it changed, I can't remember how long ago now, you probably know better. And, so, it just appeared and was there and working and just cracked on with it really.

<u>Sub-Theme 2iii: Implementation drivers</u>

<u>Software developer staff</u>

Software developer staff 1

R: Yeah. But sometimes they'll…and then the CCG will feed back to us as to feedback. But I do remember the old account managers used to do…would recommend doing a GP survey. And I think that's something we might think about reintroducing. It's encouraging medicines management teams to send out an (the CDS) survey, what do they like, what don't they like, what would they like to see. Because then that helps with our development of [inaudible 0:46:17]. Because all we tend to get is from the MM teams about using the tool itself, so using the portal and the messages. Yet we do seem to forget a little bit the people that are using it on the other end every single day, and so what would they prefer to see. Some MM teams do that, some MM teams will use the data from he CDS at their review meetings with the GPs and use that as a feedback mechanism. Some MM teams go, I don't care if they don't like it, they're still having it anyway, type of mentality. But it's like…I know, it doesn't go down well. So it's probably something I think we could look at. I might mention it on the call tomorrow as to whether we start reintroducing almost a yearly GP feedback survey to come back to us as well as the MM team.

Software developer staff 2

R: Probably this past year the main things have been around the formulary tool the CCGs a bit more control around that. And then, something that we call the multi profile tool, which is really just acknowledging that we know that CCGs are coming to work together, either merging or coming as an SDP type scenario. And yet they've started off with individual (the CDS) accounts and profiles we call them. So how do they suddenly become one and how do they manage that, and actually is it that simple? The multiprofile tool is there to either help the align or to help them manage having more than one profile and knowing that they've got to have some differences. Because these big organisations are still having localities, they have more than one acute trust [voices overlap 0:39:18].

Software developer staff 3

R: I think the biggest problem, well one of the biggest problems, is the maturity of the CCG itself. […] So, some of the best, again, I don't get out that much to talk to everyone these days, but the best CCGs are the ones who've got a clear meds management, meds optimisation plan. […] What are they trying to achieve? They've got a clear cost target, and they've also got a very clear outcome, or outcome for different areas of focus or work. […] 'Cause in some ways, (the CDS) is a change tool, in a lot of ways. It allows you to understand where you are now, and where you want to get to, and by putting in these groups of messages, you can impact change, and we give you the ability to measure that change, or to track that change. So, if you come to this tool, just thinking about it, as, I want to save as much money as possible, and put in all my cost messages, apply them, I mean that's what you're getting out of the tool, and we can give you that, but what we're finding it, the ones who come to us and go, actually, we know that cost savings are low hanging fruit, we've been doing it for years, we just need you to continue that, but we want to focus on

these areas. And I think the market are moving, I think costings are moving. But, it takes quite strong leadership and direction for that message to get from your heads-of down to your pharmacist and your technicians, who are working in the teams or in practices. And so, it's very clear when you go and meet somebody at MMTs, which side of the fence they lie on, or have they got a clear feeling for what their priorities are?

CCG Staff

CCG Pharmacist 3

Making sure that practices felt involved, and it wasn't something that was being done to them, that they had a choice but that was [down there 17:53]. But I think what I have realised in this organisation, it's about establishing who holds the power. And I established quite early on that in this area it is the practice managers. So I went to them. We did the clinical bit with the prescribing leads, but then I went to the practice managers. Because they were key to getting their practices on. And because I involved them, I told them what we were doing and it was in a different focus from cost-saving, it was…the focus was on patient safety. And that was what I kept pushing. Yes, there are cost-savings but the focus is patient safety, so I think it was because I ensured that I got the practice managers and there was a meeting which I attend with primary care where you have practice managers that represent each area and making sure they had the information so that they could feed it back to their colleagues.

CCG Pharmacist 3

R: It was the main driver, we had a near miss with the previous system that we used with the formulary, there was a near miss. And I was given the task of looking at the root cause analysis and what could be done to prevent such a thing happening. So no patient was harmed, but it was quite a serious near miss. So this was my solution.

CCG Pharmacist 3

R: We're changing things constantly because new information is coming out, new decisions are being made. And they need something to help them, because they've got a lot of responsibility and they've got a person sitting in front of them, they're supposed to know everything about the patient, the best medication for them, but then also we're nipping away at their heels about cost. So we needed something there and it's supposed to be patient-centred care, so it's about looking at what is best for the patient. So it was putting the patient in the middle of it and thinking putting everything aside, okay, I know there's money, there's this, but what is safest for the patient? So I looked at it from a patient-centred approach. And that was how I managed to take it forward to a degree.

CCG Pharmacist 3

The main thing that has changed over the year, there was a problem with (GP CLINICAL SYSTEM) in particular.

I: Oh, right.

R: There were problems with…I can't remember the origins of the problems, but there was a fault with the software due to the change I think TPP and (GP CLINICAL SYSTEM) had made, so it did go down for some time, I think it was about the better part of a day. TPP went back online pretty quickly, but (GP CLINICAL SYSTEM) didn't; and we had to wait for an update of (GP CLINICAL SYSTEM) which took them about four months to implement, because even though it was an upgrade that was needed on (GP CLINICAL SYSTEM), which needed to implement (the CDS) software, they don't do updates for just Optimise. […] There's a regular update process for the whole clinical system, so they would have several things in there. And as they update they tend to roll it out, but they don't do everybody at the same time, they tend to roll it out to a small group of practices and then roll it out to more practices, so it's a phased approach. But as a result of problems that happened with other updates they didn't roll it out across all practices until in totality four months. As a result we had a couple of practices that switched off.

CCG Pharmacist 5

I: Thinking, sort of, of other things, you've got these structural changes with, you know, you're now becoming...across six CCGs you're becoming one CCG. Are there any other, sort of, structural changes, like PCNs coming on board which you think are going to make an impact upon it at all?

R: I'm not sure it will actually...so the PCN staff coming on board, I think, will benefit enormously from Optimise, and I don't think we've really sorted out our working relationships with them yet, to get messages out to them and give them updates on what to change on the formulary and all that yet. So, (the CDS)is a really fantastic way of making sure that they are actually getting those messages. Other than PCNs I can't really think of any other structural changes that are going to have a big impact, really.

But, it doesn't matter how many people are using the system, it doesn't change the amount of work we've got to do on it. That's basically...

I: No, it's just, I was thinking more...

R: ...the beauty of it.

CCG Pharmacist 6

Yeah, so initially, when we piloted it, we did a whole questionnaire on feedback. Yes, so a feedback form for the GPs to actually let us know how they felt. So I think it was setting the expectations from the outset was really important, so it was like, actually, do you know what? This tool is here as a pilot, it's not going to solve all your problems or issues that you have, it's going to be a bit...like there's going to be, you know, a few issues that you'll find, but bear with us because we'll work on them, and that's where your feedback is really important. Because otherwise, I think the GPs tend to switch things off because they think, I don't like this and I can't change it, and I don't own it.

CCG Pharmacist 7 and CCG Pharmacy Technician 2

I: So, well either of you, it doesn't really matter, I mean we're talking about…perhaps if we go back to the very beginning of when (the CDS)was introduced in this area – what sort of things happened, what drove it forward or how did you go about bringing it into the…in?

R1: We had Script Switch before. There'd been issues with Script Switch, so we were looking at some…whether it was time to change to a new system, and there were only two systems that were available. So we did a paper that went to…I can't remember where it went… It went…followed an audit trail through the CCG committees, where we had a sort of pros and cons for each system. And then there was a…so there was an organisational decision to go with (the CDS), so that was it really. So that was…it's not that we hadn't used the things before, we had, we'd used Script Switch before.

R2: It was going wrong, wasn't it? It was saying that…

R1: We had issues with it a number of times. So that's how we kind of came to move on to Optimise.

Theme 3: The work required to implement (the CDS).

Sub themes 3a:Building, maintaining (the CDS) profile - priorities and expectations.

Sub-Theme 3a i: Management, maintenance and customisation of (the CDS).

Software developer staff
Software developer staff 2
I: Well, GPs almost by nature, they like something brief, to the point, concise. It was something I think that came out of the user group, I think someone was saying about that, that the change in the wording was going to help.
R: Yeah. But I guess in terms of nationally have acceptance rates gone up? They've not. They've kind of levelled. But there's a big driver for that in that this year a lot of CCGs have enabled self-care messages, the messages that say you shouldn't be prescribing [inaudible 0:26:34], you shouldn't be prescribing pain killers, they should be going to the pharmacy and buying it. Now, we produced a suite of messages in response to NHS England, and we had a lot of customer pressure to do that. We were always concerned that these were going to fire indiscriminately, even though we've brought in as much logic as we can think of to try and rule out patients for whom that wouldn't be appropriate. So, CCGs who have enabled those, the message there as a whole only gets accepted around ten per cent of the time. There is variation in that. So that is kind of dragging down their overall acceptance rate. CCGs that don't have these enabled, generally have a higher overall acceptance rate. Again, as account managers, we'll often highlight that, and these can account for 15, 25 per cent of the messages being seen. It's huge. It's like 30 messages are counting for up to a third of the hit count. So we're always trying to make CCGs aware of the decisions that they're making and how it's influencing… We get a whole spectrum. We get the well, this is really important, we're being beaten at the head by NHS England, we've got to do it, to well, we take a more pragmatic view and we're cycling the messages and… Because we know there's an alert fatigue. But then that creates, oh well does that mean if the message doesn't fire, it's okay to prescribe it [inaudible 0:28:15] complex.

Software developer staff 2
R: Not yet. We've got an eye on PCNs. I think we'll need to look at those more closely. I think from an Analyse perspective PCNs has come to the forefront, but from an (the CDS) perspective certainly the next 12 months will still be a CCG sale I think. We're looking at reporting at the moment, there's work underway on that to make reporting much more visual and more… We've always had good data but we've not always had the best tool to be able to drag it out. So the bases aren't changing, but hopefully they'll be able to see a bit more or maybe I'll be able to group a PCN and give a PCN level report, rather than a CCG or practice, but that kind of middle tiering and give people more insight to what's happening. More granular rather than people just focusing on the high level. So I think that that will help, and that will maybe drive more conversation. Whether people want to manage (the CDS) at a PCN level, again the multiple profile tool could allow that. My personal feeling, I don't think it needs to be that granular, but [voices overlap 0:40:51].

CCG Staff
CCG Pharmacist 1
We have asked that they translate all the information from [area prescribing group] onto the system, and we thought we had that but it turns out we didn't. So there is still a mismatch. I think because it's a national system and we're working on a regional level they often are unaware of regional variations between maybe what happens in [name of place] around [name of University] et cetera, and maybe what happens in in [name of place] from the in [name of place] new drugs group or where there are regional decision making bodies or groups, and somehow they seem very focused on national stuff, we don't know what they will do with the RMOCs, but sometimes that gets seen as more of a driver. The reality is that few people would be able to cite what NICE say about drug X, but they're quite au fait with talking at what [area prescribing group] says simply because they understand the process here and we have a GP on the patch who's part of that decision making process and so they understand the input and the links and the confidence they have around the system, whereas few people would actually venture into the NICE website and look what NICE says about it because it's too big and they don't feel connected with that process, and yet (the CDS)seem very focused on the national recommendations, and if I have a comment say well, that's okay, but at least give people the opportunity to see what the local decision making groups say.

CCG Pharmacist 2
R: And we also have members of our team who specialise in different areas, so, another one of our technicians specialises in respiratory, so I will have an update with her every few months. We'll go through the whole of the respiratory section and, just make sure you're happy with everything. Is there anything new that needs to go on? And then I'll request it. We have an antibiotic specialist pharmacist, and the same with her, we go through the antibiotics, has anything changed, et cetera? There are things, because we've had it 12 months, there are still things I need to develop, and so things like wound care, oral nutrition, incontinence, more continence products, stoma, those things adding by the specialist nurse in, to sit with me, and from (name of place). And I need to know from them what the triggers are and what message you want to come up on the screen.

CCG Pharmacist 2
So basically, I'm due one (a review with the software developers) next month and they'll look at our profile and maybe we'll go through things that seem to be working, which they can, you can run reports yourself but they'll do all that for me because I just don't have the time at the moment. And they will show me which ones don't seem to be useful, because no one is really accepting them. Or they'll show us, so bring forward some ideas of what we could maybe change it, how it, and maybe that would make it, the uptake might improve, or whether it's just something that just doesn't, that simply doesn't work so we need to address it in a different way.

CCG Pharmacist 3
R: I think it's an embedded service now […] I have a team of pharmacists that update the profile, review messages that come through, request new messages. I might be…and then I manage that team and I supervise that team. I might be checking appropriateness of messages when we ask for new messages.

CCG Pharmacist 3

I: And one final question then; how much work is required to sustain, to keep that level of sustaining the intervention going, to keep it going?
R: That's difficult to quantify at the moment, because we have been working differently, and we're moving to a different system. […] I think there is still a significant amount of time. I mean I would have to… I mean I think you will be speaking to a couple of the people who actually work on the profile, and they'll be able to give you an idea of some of the time they spend updating the profile. But theoretically the amount of time that they spend updating the profile should have been reduced over the year, and it's been reduced even further, because if you think we spent a certain amount of time updating the profile in (name of place) they spent a significant amount of time updating the profile in (name of place). Now because we worked together we were both able to cut down on the time that we used from both sides; but as this team that I have in place would be updating the first half for the whole of (name of region) that is going to cut the time drastically. I wouldn't say we've cut it in half exactly, initially, but it will cut it drastically. And then also there are other updates on (the CDS)which they've done, which does involve us doing things ourselves. […] And me, I feel that may well increase the amount of time we do use updating the profile; but then again until we work with it for the next year or so we don't know how significant that will be. So they've given us more functionality, which is fine, but then the onus is on us to do more ourselves.

CCG Pharmacist 4
So we have a rota between us, so every two weeks we are on rota, and that will involve looking at what update(s) (the CDS)have posted in that two week period. So every two weeks they release an updated series of messages so we look at those, switch them on if they make sense and they're uncontentious, discuss them amongst ourselves and make a decision. If we're not sure we refer it up to (name) or (name). If we're really not sure we'll just switch it off if it doesn't make sense, or not activate it.

CCG Pharmacist 5
R: So, I've set up a monthly rota so we've got a team of pharmacists that work on (the CDS) so we've got representation from each of the four (name of place), CCGs. We meet every couple of months. We did meet more regularly when we were merging but it's becoming more routine now so we don't need to meet as often. So as part of the rota, one pharmacist will take on reviewing new (the CDS) messages for one month […] Whilst they're doing that, they will also answer any queries that come in. So, we've got a centralised log for all of the CCG pharmacists to write if they've got a problem or if they spot a message that's not right. So that log gets looked at and any problems are sorted out or brought to the next meeting if they're too complicated to sort out straightaway. We also review the bulletin that (the CDS) send out so that we can see if there's any changes to messages that we need to action or if, say, a cost-saving on a message has changed drastically and it's no longer worth it for the GPs to see that message, we can turn it off. If we've got queries on messages, we have a system which is the same as (name of place) where you write help in the comments box next to the message so you can then find all your help messages and go through them to see if you can tick any of those off and solve any problems.

CCG Pharmacist 5
R: And now we're in talks with (name of place) to bring their profile into ours as well, so we'll have six CCGs all on one profile, if we can make that work, so…
I: Right.
R: And I think it's sounding promising, so...because we share one formulary that makes it a lot easier.
I: So, you already to that across those...?
R: [Voices overlap 00:01:18] those six CCGs already share one joint formulary, which means that there's no real reason why we couldn't have one profile. It's just little commissioning differences between the two that makes things slightly more difficult. But I think we'll be able to overcome those.

CCG Pharmacist 5
R: It just means there are fewer people that need to be involved in maintaining the profile, so when we have two profiles running it's not quite double the staff because we have people involved from every CCG, so we've got more people working on it in (name of place) than (name of place) But they've got the same amount of work to do, so they find it very, very time consuming with the two of them doing it for the whole of (name of place). So, they'll notice a huge difference when they move over to us. So, we have a rota for managing the profiles, so every month one person is responsible for checking all the new messages, enabling them, checking our query log and seeing if anything needs updating or changing. So, when we bring (name of place)on board, they'll only have to do that twice a year, so, once every six months or so, which will be a huge improvement for them because they're doing it all the time at the moment.
I: So, it's really a resource.
R: It's a resource, it's not cost saving, (the CDS) have said they won't give us a reduction in price if we bring (name of place) on board, whereas we did get a reduction when we joined the four outer (name of place) ones. They said that, you know, we're at rock bottom, so...yeah, so it is just staff time that's going to make a difference, and consistency across the whole of (name of place), so the GPs will notice that they'll get the same messages wherever they work in the whole of (name of county). Whereas, at the moment, they could get different messages in (name of place) than in (name of place)

CCG Pharmacist 5
I: So, that's feedback with GPs. What about the other end feedback, and the partner work as it were, with FDB?
R: That seems to be quite good. So, the user group was really useful, that was nice to meet all of their staff. But we've got a really good relationship with our account manager, we know that we can...because she generally replies within a few hours to emails. Yeah, we know we can pick up the phone if we need to. So far, I think that seems to be going pretty well, and the content team, so, if we put in a request for a new message the content team are quite responsive. They did go through a phase of being quite slow, but they seem to have sorted that out now.
I: I heard that they were allowing you to write some of the current messages [voices overlap 00:27:16].
R: Yeah, so we can change the formulary messages now, but only the generic ones. So, we have a grey category for drugs that are non-formulary. So that, for an example, we can change the trigger list on that, so the drugs that are in the grey list, we can add those in and take them off ourselves now. But if we had a specific message for a particular grey drug that was more complicated, then FDB still author those ones for us. But that's saved us a lot of time, and probably them as well.
I: And have they in...I think they were changing some of the way they were wri...the phrasing of some of the messages. Has that changed at all?

R: So, we can edit, I think we can edit those formulary messages so we can change the wording in those messages, but it is very small, the group of messages that we can author ourselves. Yeah, and we can add links onto...oh, yes, so we can add our own links onto, but we can also, there's now a new box where you can write CCG notes, so we can, sort of, add an extra bit onto their messages as well, which is really helpful.
I: Again, to help for that...?
R: Yeah.
I: ...relevance and...?
R: Yeah, and I think on some of the national messages, you can change which drugs it gives as an alternative. Which is getting...that's much better, so it will only offer formulary options then, as choices for them, yeah. To save them accidentally picking a non-formulary option and then getting another message that says, oh, you can't have that one.

CCG Pharmacist 6
Yeah, but we still need to keep an eye on what we turn on/turn off, we need to keep an eye on the reporting part of it. So (name) our analyst, looks at a lot of the backend reports that come through and... I'll let you talk about what you do in a minute, but from the frontend part of it, from our profile point of view, we have a working group that we've set up. So we have a representative from each CCG that sits on the working group, so we have a strategic meeting every three months, which I chair, and then every other month the working group gets together and looks at the messages and looks at what needs to be turned on, what needs to be turned off, and there's a rotation of pharmacists. So, like, one month the pharmacist would be responsible for making sure that all messages that need to be turned on are turned on, and all the other extra bits that need to be done, like, I don't know, inform GPs of changes, maybe. But if there are any queries that they have, we also have a queries log, which they then…if they're not sure about anything, they will then put on the queries log and bring that to one of the meetings to discuss as a group. So that's how we maintain the profile really. So the workload is shared across the CCGs.

CCG Pharmacist 7 and CCG Pharmacy Technician 2
I: And then carrying onto that then how much do you have to do with…I mean, we've talked about how they…
R1: It depends but we try and do maybe a session in a fortnight, something like that, so maybe an hour or two.
R2: Yeah.
R1: And we did a lot more at the start because obviously we started off with, I think it was (name of CCG).
R2: I think so, we used their profile.
R1: ...profile, we used that as a sort of basis. So they'd had it before us so we used that as a basis and then we kind of added to it.
R2: But we didn't have everything on, did we? We just selected what we wanted and we just went very slow, small and slow.
R1: And we just added things on. So we did a lot more – we met…probably we were going, like, once a week at the start and just, like, bit by bit built up the profile.

<u>GP Staff</u>
GP6
But in reality I'm not giving it anyway because I wouldn't give that drug. But do you see what I mean, if a locum is doing that or something, there's nothing useful there to say, and actually please don't prescribe Trimethoprim because it's not even third line advice in this neighbourhood and that's because it hasn't been customised for our local patch.

<u>Sub-theme: 3a ii Profile management - Processes and approaches</u>

<u>Software developer staff</u>
Software developer staff 1
And I'm trying to think. So this one organisation that we need to sort out has turned on about 1,600 messages. And that's bigger than I think most of our customers have got. Most customers tend to sit around 1,200 messages. We still have some customers that have about 400 messages, and they just don't…and they're very, very careful about how they use it. And some have all best practice, very little cost. We have ones that are all about the cost and throw in some best practice. Every organisation is very different to how they use the tool.

Software developer staff 1
So I think from the other end, so for the CCG, the purchase of, they've seen a huge development from how to enable to messages, how to run reports, and now giving them the flexibility to go in and alter messages themselves without having to go through our content team. And even in the last 18 months, that development has really rolled on. Where I was before, it was crazy because that was our own piece of software, so really we could have done any development requests. And I was with that company for six years and even after six years, we hadn't delivered the one thing that people wanted, which is the ability to have coloured text, for instance. So it's been really good for me to come to a company that we try and listen and development. Our sticking block is not our end, it's our partners' end.

Software developer staff 1
So, because of issues around staffing and just on-boarding so many new customers, it's all…you know, capacity is just…so actually, a new role that we've had agreed this morning that we'll also be doing, is triaging some of our customers' content on behalf of the content team. Because we're all pharmacists, (names), we are all pharmacists, so we are able to give that clinical…but it's more to check, if they have a request come in that's so ambiguous, you know, what do they want out of this message, just to cut down on time. So, at the moment, we are not fire-fighting, we are managing expectations at the moment. So, we have a whole host of unhappy customers at the moment. So that's the only thing, as much as…it's a brilliant tool and when it's…it's just (name of place), have got issues around staff, and that's affected our customers. And the trouble is, as account managers, we get all off. And we've been shouting about this since middle of last year when it first started to rumble, and only now are things being done. So that's a bit frustrating.

Software developer staff 1
But I think we've grown so quickly, and we didn't plan for it, you know. And we should have got a lot more people ready and trained for when that work came in. Which is why…and I think they're hoping that giving our customers the ability to alter national messages themselves or take that off, but our selling point is we'll do the work for you, so why would they want to do the work. If they've got to do the work, they might as well save 10p

a patient and go back to the other piece of software. And I think that's my only worry. I mean, they'd all agree that they prefer our software, but speed and turnaround of content at the moment is a big issue.

Software developer staff 1
So, we'll do all of that and then we sort of go to, you know, normal account management. So, each account manager, we have around 20 to 30 customers each in a sort of geographical area. We're trying to work around what the STPs are going to look like, but who knows because they're taking so long to form. Whether they'll actually come to fruition, who knows. We will see our customers we say about four times a year. We do have some customers, one north west of here, that want to be seen twice a year, don't want to see us four times a year, it's like there's no need, we're fine, is it getting its money? Yeah, you know, that is fine by them. We have other customers that would love to see us far more regularly or want more touchpoints if they're having any issues or whatever. So, we are the point of contact for those customers, we're the [paying 0:28:52] points, you know, they can come to us with content queries, with training requirements, if a new GP practice has come on board, if they need help with the portal, if they want help with content. So, with my customer yesterday, I thought it was going to be quite a difficult meeting. It ended up being fine, but I'm having to do some report work for her, because that way if I do the work, I know she'll do the work on the profile, and if work's done on the profile, the GPs will be engaged.

Software developer staff 2
R: So, in essence, they do everything with me as an advisory role. Maybe advise is a strong word. Maybe a training role or we give them the tools as to how to do things, how to physically switch them on and off. So, there's kind of a concept that we're bubbling up now of content strategy and getting people to think a bit more bigger picture, what am I actually trying to achieve, is this the best way to do it, is (the CDS) the vehicle to make that change. Sometimes historically people have thought let's put it on our CDS system and let it happen, yet we know that certain things don't work particularly well. So, an example might be inhaler switches, which asthma, COPD is often managed by practice nurses in reality, and so a GP might re-authorise a prescription, get a message saying that's not really the inhaler they should be on anymore, our first choice is something else. But that's quite a difficult thing to just click a button and say accept because the patient may not be there, it might be different, actually it's managed by the nurse and they're not really familiar with the inhaler devices anyway. So just putting it on (the CDS) isn't going to necessarily drive through that change.

R: So, as account managers, we aim to visit once a quarter at least to meet our CCG contacts and see how things are going really. So we'll cover a broad range of topics. Hopefully they will actually come to the table with a list. Some do. And we can go through those. That might be around profile management, reporting, any particular issues with any individual practices, although they become less, hopefully, over time, the value that they're getting from the system, innovation about how to use it, and also an opportunity for us to tell them about forthcoming changes, developments, et cetera.

I: And how do those meetings pan out as it were?

R: They're usually pretty good really. I mean, all the customers are different, which is what makes the role interesting I guess. But yeah, as you start to see people more frequently and you've seen them over a year or so and they've got to know you a bit better it can be really quite informative, and when they begin to share things with us about what they're trying to do with it that's really enlightening because we can then maybe share oh, I think I know a CCG that's done something like that and begin to share best practice or different ways of working.

Software developer staff 2
I: Yeah. So what sort of changes have been going on?
R: So I think from a functionality perspective, in terms of what the GP sees popping up on (GP CLINICAL SYSTEM) or (GP clinical system), there's probably been no change really. But I think in the background, our content team has probably matured quite a bit in terms of their experience and things. So the experience that our CCG customers have had in terms of the turnaround of content and the quality of content that we're creating at both local and national, I think has probably improved. So whilst things look and feel the same on the screen, hopefully GPs are seeing more…we're keeping up that relevance and making things timely and similar stuff.

Software developer staff 3
And they turn everything, and that's fine. And them you'll get your reporting usage, and then they can tweak or adjust as required. You'll get other customers who'll go, well we were using the other system, and all we want to replicate is the message from the other system, and put that in place, 'cause that's all we care about. So, what you'll get there is customers who are very cost focused, but don't really think about best practice, or safety. And then there's customers who are really clued in, and say, actually we just want to turn on three - four hundred algorithms, and it's because it aligns to x. y and z in our plan, and we want to track and monitor, and see how successful we are. And really we want to get everyone on to that last course, so they're putting some sort of consideration into why they're switching something on. […] So, there's this concept of profile management, and profile optimisation so we're actively working much more closely with our AMs, to make sure they are, one, that they understand, well, they're making sure customers understand which messages deliver what type of value, and also with that, hence the grouping bit becomes important. So, if you have to understand, your focus is on this I see, from your strategic priority, or you've got an incentive plan, so these are the messages you should be grouping together, and then we'll show you how those are being adopted.

<u>CCG Staff</u>
CCG Pharmacist 1
And we have to just try and put it all in an email, and it caused a few issues last year. We have a clinical needs policy whereby a lot of the things which are available OTC basically don't prescribe this, tell the patient to go and get it from the minor ailment scheme. Now, the complexities of trying to get them to understand that and translate it into recommendations on Optimise, I just don't know how much time we spent on that one. It was weeks. Because we couldn't talk to them. All we could do is try and put it in an email. Then they came back with are you saying this, and we said not quite, we sent it back. So we spent hours and hours via emails trying to get them to understand what it was that we wanted to say. Because basically you could give paracetamol for chronic osteoarthritis, which is okay, but we're not about giving paracetamol because somebody's got cold and flu like symptoms. We want them to go and buy that. But that concept was a problem. Two forms of paracetamol, almost, one for long term condition, fine, one for an acute presentation, not fine, go buy it or go and access it. But that, because they base it

around the drug and the clinical condition, all of which caused them immense problems. So, we don't want people prescribed hay fever for seasonal allergy, but if you happen to have urticaria it's fine. But again, trying to get them to understand that distinction was very, very difficult.

CCG Pharmacist 2
R: Well, they gave us a choice. They said we could go with a profile that everything's on, but not enabled, so we would go through and switch on what we wanted. That was one choice. Or we could, more or less, have that same profile with it all enabled straightaway. So, we decided to go step by step and just enable what we wanted, because of the history we had with pop-up fatigue and we didn't want to overdo it. Plus, even the time capacity, just to go through together what we wanted enabling, initially, which took time, which we didn't have, so we went through very slowly, step by step. And it worked really well because our acceptance rate is one of the highest, I think. Yes, so it worked really well.

CCG Pharmacist 2
I: And why, what do you think's, what do you think is the reason behind that?

R: I just think we put real thought into what we enable, what we think would be useful and we go at a slow pace rather than sprinting to get everything on, that actually would just irritate because it's over, you get pop ups, you don't just get (the CDS)pop ups in your surgery, you get all sorts that pop up. So, I think we're just very, we just think things through, what would be useful. And I think that's probably the reason why it's accepted better, because they are more likely to accept things because it's more, you know, it's more useful than just something you can't… If you enabled everything on the profile well it's, I don't think that's a really good way of managing it because you need to check in detail what, if it actually is useful for your population and if it sort of complements the kind of work that we're aiming to do.

I: Yes, right. In what ways, in what ways complements that work?

R: So, if we're, like with these, say a specific project or something. For example the gluten free, we've had new national guidance that's come out very recently so we're able to use the message that's on (the CDS) that identifies the products that are no longer allowed on the NHS, and from that we can attach our own patients' letters and leaflets to explain in more detail. So when they come up, the practice can print them off and send them to that patient. So for that, I think for things like that, that would be an example of how it complements what we do.

CCG Pharmacist 2
At the same time I think some of the feedback that we've had as well is… I don't know if that was still rumbling on when you had interviewed myself, things like the national…there's like national quality premiums for antibiotics and things. One of them is reducing the use of trimethoprim in patients who are over 70 and all that sort of thing. So that's still in the pipeline a bit because we had put forward, this is what we'd like, this is the message and this is when it should trigger and all that sort of thing. I think (the CDS) are alongside this unbeknown to us, I think, were doing a national one related I think, sort of, nitrofurantoin and trimethoprim prescribing. What they came up with was actually a little bit different to what the guidelines suggested, what we were trying to do locally. So I think they said something…and it was all to do with dipstick testing as well so it all got a bit more complicated than we'd originally anticipated, sort of thing. So I think that's still a work in progress. I'm still waiting for this message that we proposed be produced and then maybe be overridden by the national one [voices overlapping 04:53].

I: This was the antibiotics that are basically not effective, is that right?

R: So, yes, trimethoprim, it's to do with the resistance level so the high resistance level but also, yes, because the whole thing about E. coli bacteraemia in older adults is more prevalent. That's one of the other main reasons that they're trying to reduce its use in over 70s, in older patients. […] Yes, so I don't think that one ever… I'm still waiting on those ones to come through because alongside that, (the CDS) had done this national one but then in the process of all that there's been… I think part of it's to do with how national guidance has also been changing alongside all of this. Locally we've done a lot of work with like care homes and patients who are over 65 years of age and encouraging them not to dipstick, urine dipstick tests to diagnose like UTIs and things. When (the CDS)'s message came back it still was saying, anyone under 69 years of age should have a dipstick test. We were like, well, that contradicts what we're trying to do. I think we just…you know, just liaising with them about making sure that it matches the message we're trying to get across locally, sort of thing. It sounds like they're going to be changing that one according to national guidance which does say that 65 years or above you don't dipstick test urine [inaudible 06:18].

CCG Pharmacist 2
I: So you can add in extra stuff to the profile without going through (the CDS)to…?

R: Yes, because if it's already on then there's certain ones that you can edit. You can't edit all of them but if they're, I think if they're national messages there's an option where they have a CCG text box in it and you can, we have permission to add our own message to it, say if there's something that we need to say that's more specific locally, or any additional information, so that can be added in. Yeah, so that's pretty good.

CCG Pharmacist 2
There might be things that come up locally. They might be mentioned at the hospital, that things that are coming through that shouldn't be, that aren't approved, and, oh right, we can maybe ask for that to be added onto (the CDS). Or things at meetings; I've had two meetings today where I've got half a dozen things that could potentially go onto (the CDS) to help our GPs. So yeah, things like that. Meetings help a lot, and emails from staff, things that you notice when you're in surgeries. There might be a pattern of things that are going on, that, actually, that's not recommended. Where's that coming from? Let's have conversations. And then once that's sorted, let's put it on (the CDS). It's that kind of thing, yeah.

CCG Pharmacist 3
R: Well, with the profile, one of my selling points to getting it approved was that the (other part of the county) already had it, and we had joint decisions, so what they had should be 100 per cent applicable to us. So that meant there was not minimum but less effort needed from our part in setting it up. And also what it was is that they had set it up but they hadn't checked it. So we now checked it, found omissions and whatnot, because it happens, and we were able to put that in for ourselves and for them. So I can't tell you how many alerts and whatnot we have but I

think we spent less…well, I'm not sure we spent less time than any other CCG because we already had a profile to work off of. But that was one of my selling points.

CCG Pharmacist 3
So probably actually taking off messages that are really not useful, and we've seen that from our records because it's like a 100 per cent rejection rate, or they don't get offered very often.
I: Yes. Right. So either ones that fire and are universally rejected? Or ones that never actually fire?
R: Yeah.

CCG Pharmacist 3
R: That works very well, because I have a little team that work on it; I have a team of pharmacists, and they regularly update everything, all the messages that come through, all the requests for messages, all the traffic light changes; so I have a very effective team that actually do that between them. And then we have quarterly meetings with (software developers) ; so that works well. I'm not sure how it's going to work going forward because it's going to slightly different; but the last thing which has been done is we've worked towards, as I said earlier on, merging the profiles, that was always on the cards from the time we had taken (the CDS) on, and I think we have a go-live date for next month actually.

CCG Pharmacist 3
R: Yes. Over the last, well since we implemented it, and especially over the last year we have been working to ensure that messages put on or deactivated are the same across the patch. So my teams worked with looking at the differences, so they've looked at the differences, dealt with all the differences, and so now it will just be one profile and it will be that one of the CCGs will switch over to the other profile; but it will be the same, it's just that the one they're switching over to will be more up to date because all the latest messages have been activated then as a result of the system which I have in place.

CCG Pharmacist 3
I: Right. That's interesting, isn't it, because I've heard that before, where some of the messaging is being altered, as it were, by yourselves at the CCGs, rather than at (software developers).
R: Yeah.
I: So a sort of decentralisation of it, isn't it, which is quite interesting.
R: [Voices overlap 0:28:14] very enthusiastic about, personally I'm not enthusiastic about it.
I: Right. Why?
R: Because I feel one of the reasons that we went for (the CDS) was for that level of peer support and safety; and I feel that things done across a wider network with more people involved there is less room for error.
I: Right. Okay. So because it goes wider, that safety aspect is being further taken away, it's being more…
R: Well, I think it's also being the responsibility is being shared, so like for instance, whereas we would make…two or three of us would get together and say we want this message on for a reason, because we'd have to send it to (the CDS) to author, they would do a double check against national guidance, they would also look at the messages that were already on the clinical system, and because they have specific criteria for authorising messages I feel that's effectively a third check; and there are some messages that they were not authored for a particular reason, it does make us think again. And there are some cases where we insist that, yes, we do want it on our profile and they will do it just for us, but I just feel that it's a bit of a safety net, and I don't think when you're coming to people's health you can always be too safe.
I: No. No, precisely. Precisely.
R: And having other expertise involved, and it can only be of a benefit; and I worry about that if we are doing all these things ourselves. Because if we're doing all these things ourselves how much of it could we do ourselves? Do we need to have Optimise? Okay, I mean I know that the technical aspect, and that's why I'm a little concerned, it's kind of like watch this space carefully.

CCG Pharmacist 4
R: I was very concerned about the fact that we're maintaining a formulary, there's a huge amount of resource goes into that, there's a lot of resource that goes into (the CDS), and actually I have this big thing about duplication and simplification at work and actually really don't like doing things twice. So I suppose my role is to try and make sure that we use both to their potential and appropriately really. But with such a big team everybody's got different opinions on what should be used for what, and actually a recent example would be the MHRA alert. Well, it's not that recent, but about patients should be involved in which brand they should have for anti-epileptic drugs, the class one and class two, and there's messages already on the embedded system, so TPP have got messages in, (GP CLINICAL SYSTEM) has got messages in. The (software developers) are quite strict on the fact that actually they don't author messages if it's in the host system they can't write new messages, which is right. And ultimately again it's in one place, you don't want it in another. So it's sort of maintaining the continuity as best we can really.

CCG Pharmacist 4
This is one of the fights we have with the formulary. So with the formulary we have to wait for that decision to go through the area prescribing committee, then we have to wait for it to be ratified, then we have to wait for the minutes to go online, and only then can we amend the formulary. So that process can be anything up to six weeks. So there's always that period of time when something's actually on formulary but nobody knows about it. So very timely example is rosuvastatin, cholesterol drug that's been off patent for six months. Bit cheaper now. I'm also doing some work with (name of CCG area) diabetic service in looking at improving cholesterol in patients, and one of the things I want to do is use rosuvastatin now it's off patent. On our formulary it's still saying consultant use only. It went onto the formulary on 24 April but that process hasn't gone through. So I've authored a paper to get it reclassified and it's already been done because the process is… So you've got that process and then you've got the (the CDS) authoring on top of it which could be up to another six weeks. So you've got three month lag where the formulary doesn't necessarily meet Optimise, so that's something that has caused a little bit of friction.

CCG Pharmacist 5
I think the amount of work you've got to put into it is quite immense so although you're paying for (the CDS) to do a lot of the organisation for you, you've still got to do a lot at your end […] There is a team of us spending half a day every couple of months chewing over data or whatever and then one person every month who has a portion of their time so cost is part of it. I think other drawbacks, I think sometimes it feels like we could do more ourselves. It would be nice to be able to edit a message quickly. It's often not timely so if there's a formulary change or a change of

guideline and we want to change something quickly, it's often not possible to do. You end up turning the message off and waiting for the new message to arrive so there's a period of time where you haven't got any message for that particular drug […] It's getting slightly better. On the national messages you can now put a CCG note so you can personalise some of the national messages which is really helpful but I think there's more they could do to be able to allow us to do a little bit more ourselves and quickly. In the time it takes us to write a request to them to change something, we could have just changed it ourselves and it would save them a lot of time and might reduce the lead time for new messages.

CCG Pharmacist 5
I think without it, it wouldn't work because your messages would be out of date or incorrect or there'd be too many turned on or not enough turned on. You couldn't do it without a team of people working on it, I don't think. I think it would be months, probably, before the GPs got so fed up with it, they'd just turn it off. So essential.

CCG Pharmacist 5
For our team, personally, we're going to work a bit harder on making sure the messages they do get are relevant to them and useful so we're going to be working harder on looking at the rejection reasons and tailoring the messages they get. We were also thinking about turning messages on and off so they don't get fed up with them so doing it on a rolling programme so that they align with the work that we're doing. For example, the self-care work so looking at over-the-counter products that patients should be using, we're doing that in waves. So, we started with dry skin, hay fever. Some of it's seasonal anyway so we can turn the messages on and off seasonally or when we're doing the work to remind them. So yes, just trying to use it a bit more savvily, if that's a word.

CCG Pharmacist 5
R: Yes, so, as I said, there were four profiles when I came here so there were three county profiles and one city profile. The city profile's the one that's been running the longest so they'd had theirs for, I think about four or five years or something like that so theirs was very, very well established. The way that their staffing worked means that they didn't have as many pharmacists per practices as the county CCGs so they relied very heavily on their (the CDS)messages to do quite a lot of their switch work, cost-saving work and provide messages for the GPs that perhaps the county CCGs might have delivered face-to-face. So the way they used it was slightly different so the county CCGs came on board after city. They took city's profile and then adapted it as they saw fit.[…] So, we then had three separate county profiles that were all slightly different. Then when we tried to merge them, we started trying to look at all four and tried to look at which messages were turned on for which profile and see if we could come to an agreement between ourselves about which ones we turn off, turn on and see if we could harmonise them all. We started doing that and it took forever so, in the end, after a lot of discussion, we decided we were probably all singing from the same hymn sheet and that what city had done was quite sensible, really. So we adopted their profile. There were still a few changes that we had to make but they were basically around which preferred brands people were using so practical considerations rather than anything…

CCG Pharmacist 5
R: Yes, I do think I haven't come across a message that I've thought, that's wrong. I think they are very, very careful to make sure that the clinical content of their messages is right but I think the timeliness of them possibly isn't quite as good as it could be. It's getting better, I think, as…From the times it takes from us requesting a message to actually get the message or something changing nationally and for them to change their own messages, I think it doesn't work. It's not quick.

CCG Pharmacist 5
I: Yeah. Which is, as you say, it's that consistency. One of the things that we're thinking about as we're doing the follow ups and as we're thinking, you know, looking at it, with the interviews now, is how (the CDS) is sustained, how it keeps going.
R: Yeah.
I: And that seems to be something that might be part of that, but I mean, what are the things that you, as a CCG, you put in place to keep it going?
R: Well, I suppose…operationally, or that, sort of, how do we make the CCG keep paying for it, is that what you meant?
I: No, operationally.
R: Operationally, by keeping it up to date and relevant, the GPs will get what they need out of it and so they won't get fed up with it. So, it's very important that we keep on top of the profile to make the content good and up to date. And I think the systems we've got in place help with that, so we have the operational group, which does that monthly rota. I am responsible for the APC part of it and keeping the formulary messages up to date. And so, that just ticks along, we seem to have that working quite well. We meet every two months just to sort out any problems that we've had. And then we've got the strategy group, where we involve the data analysts and the senior pharmacist to, sort of, sort out future planning. And so, that's where we talk about merging with (name of place) and any contract changes, that sort of thing.

CCG Pharmacist 5
I: Yes, because in some respects, the sustainability of (the CDS) as a system then, is dependent upon that, because it won't get funded. But, does that also then impact upon, you know, the messages you put into that profile? Is it that you're then, sort of, thinking, oh, we need more cost saving measures, or is it…?
R: I honestly don't think it does. I think our priority as pharmacists working on it, I think the fact it's there, the cost saving messages, they're either relevant or there not, we will have them on or off, depending on whether or not they're going to save any money. So, that bit just happens and that's what the CCG likes, they're going to get their cost saving from that. But I think when we're working on it, we probably don't think, oh, I must put that message on because it's got this costs saving and I want to do this safety one, we'll turn it on if it's relevant. So, I think, from a strategy point of view, the cost saving is very important and we need to make sure it's delivering. But from a day to day, oh, here's a new message, do I turn it on or not? I don't think that really impacts a huge amount on decision making.

CCG Pharmacist 6
R1: In the profile maintenance of it, because we had to author our own messages before, whereas with this one, a lot of them came authored already, based on national guidance, so the actual maintenance of the profile was a lot easier. And I think that was the biggest driver for us, was that actually, yes, it's a great tool, but how much work would we need to put into it to maintain the profile? But because a lot of that work was done, and also, from a variation point of view, because the messages were from national guidance, you can feel reassured that actually

everyone's getting the same message, as opposed to having locally authored messages, which might not be in sync with what other people are doing.

CCG Pharmacist 6
R1: …what we need to be looking at is what's firing and what's not being accepted and what's being rejected, and what percentage is being rejected and whether that message is still appropriate or not appropriate? Do we need to turn it off? So that's all the backend work that we still need to do. Because there's no point having a message on if 90 per cent of your users are [...] ignoring it…

CCG Pharmacist 7 and CCG Pharmacy Technician 2
R1: And we just added things on. So we did a lot more – we met…probably we were going, like, once a week at the start and just, like, bit by bit built up the profile.
I: So you built it from a small and a low down rather than…
R1: Relatively small, yeah.
R2: Yeah, rather than switching everything on, it can be…
R1: So we didn't…we went through the MHRA stuff, so switched on things we wanted to switch on and then we started adding our own things. So I mean, it's not a small amount of work at first but once you get…you've done a lot of the basic stuff then it's…you know.
R2: It's just maintaining then, yeah.
R1: Yeah, it's then maintaining it and putting changes in.

CCG Pharmacist 7 and CCG Pharmacy Technician 2
I: Which is interesting because, you know, that profile management, when we've found, you know, as we've been talking to people, one of the things I've sort of looked at is actually, you know, that is quite a sort of tricky area of it, but you've said that's actually been easier?
R2: Oh yes, definitely because you can see straightaway…really quickly the messages that aren't being accepted.
R1: And also, because it might be for each individual…say there's one medicine, and there might be obviously different areas of how they go and do the messaging in different ways, so you can kind of pick the best one, can't you?
R2: Yeah.
R1: So you kind of…the one that you feel covers it best. So you're seeing, like, everyone's attempts at it really, because we're on the system and then you pick the ones that best…that you feel best suits what you…the message you want to get across, so you're kind of learning from others' experiences as well. I think it depends – if you'd not done anything before, you'd probably think it was a lot of work, but if you've done something before that was a lot more work then you don't think it's so bad. So it depends what you're used to really, where you're coming from.

CCG Pharmacist 7 and CCG Pharmacy Technician 2
R1: Like potentially a bit more bespoke, if you need it to be.
R2: Yeah, the national messages that everybody sees, you can put a little…there's a text box that you can put a little bit extra of your own…like your own links or your own, you know, if it's [voices overlap 08:54].
R1: Yeah, so that you might want have a link to, like, a local forum or something like that and that we'll feel we'll include with it so we can put that in, you know, with a couple of sentences.

CCG Pharmacist 7 and CCG Pharmacy Technician 2
I: And how, you know, following that… I mean, I suppose to continue with some of that profile management stuff then, how much help do you get from FDB with that, or are they…?
R2: In terms of?
I: In terms of if you want to make any changes, if you want to put a new message on or…
R2: Yeah, they are there all the time so if you needed something changing there's already some…there's always somebody online that you can send messages to – you can ring them. It depends at what point of their cycle when they release the update, so if I asked for something new this week but they've just released one last week, I'd have to wait for a couple more weeks before they're ready to release the next one and then it'll be live. But even then I would have to enable it, it wouldn't just automatically go online.
R1: Because we've got to read through and sense check it and think that's the message we want to put across.
R2: Yeah, I think they're ace support, I do yeah.
R1: There's that thing as well where the…you know, they sent the…you know, when they used to send things through on the spreadsheets and then they changed the…
R1: Yeah, that's all gone now.
R1: Yeah, so it's better now.
R1: It's all automatic…well, it's all in like a saved…like a profile within the profile if you want to request content changes. So, before we had to fill out an Excel sheet, attach it to an email and it took a long time and then I found myself chasing lots of emails, whereas this you can see, it's like a list of which ones you've sent and at what point you're up to, whether it's been agreed or if it's been completed. And then I know I can go back and I'm like, right I know that's completed on this date, I can enable it if we're happy with it. So it's all on one page then rather than me having to attach documents and a…it's a lot more straightforward.

CCG Pharmacist 7 and CCG Pharmacy Technician 2
I: So they keep that formulary, they list those messages.
R1: Yeah, because they link up to our GMMMG so there's somebody there who will update it. But you can do things like, I don't know, take drug… trigger drugs out; if you don't want certain things to fire you have that…you have a different kind of accessibility to it, so you can manipulate the formulary messages. I think that would be a project that we'd have to talk about, and certainly more city-wide, whether people want to join in and some…one person look after it, but I don't know what benefit that would be if they're updating it anyway at the moment.
R1: It's like one of the area Y things that we'll have to be working on pretty soon is around the messages that link up the NHS over-the-counter things.
R1: Yeah, healthcare.
R1: There'll be some key messages that will link to certain medicines, which are obviously for treating the conditions that are in the NHS England over-the-counter thing, and we're trying to…or the aim is really to have like the same message across the whole of(name of place) , so

everyone who uses (the CDS) and (a previous system) will have to do their own thing. But for ours that…we have like a shared set of messages, so we agree those for…so for, I don't know, head lice or whatever the thing that's the key message, you know, to go with the [inaudible 17:03] product. So it'll be a sort of joint statement on patients who are kind of in exempted categories and then messages about self-care or self-limited conditions, that type of thing. So I think we're still going to have that meeting yeah but hopefully I think that's something that's going to be organised soon. I think HMR were taking the lead on trying to organise that although we said we'd be involved as well.

Sub-theme: 3a iii. Tailoring messages in profile management and set-up to avoid excessive alerts
<u>Software developer staff</u>
Software developer staff 1
Reduce the volume of messages down, the acceptance rate goes through the roof. They don't like seeing pop-ups. So think about what pop-ups…this is the content strategy work that we're doing with (name), to try and really focus on what do you want to focus on, don't turn everything on because GPs will get disengaged. What's the interest of the GPs, what do they want to focus on. I mean, I know we have what we want to as MM [medicines management] teams, but they're out doing that work in practices. How can (the CDS)do all the work that they can't do, how can we support, and what are the GPs looking at, what is a particular issue.

Software developer staff 1
I: And I was at a meeting recently where I was talking about our evaluation, and someone, I think it was a GP, said "too many alerts". And that's…is that something you hear or is that…?

R: It's a difficult one because there's not only our alerts, there are the inbuilt CDS. So (GP CLINICAL SYSTEM) have obviously a whole lot of alerts as well, and we will then…so there'll probably be a CDS alert, then we'll appear and they'll just be like, bloody hell, it's another chuffing box. Obviously, if it's in (GP CLINICAL SYSTEM), our message will not be the same as what their message is, at least that's, I think…again, if they knew that, it's just like, oh, you know, so they don't ignore it. One of…you can always…sometimes counter argue that if they'd prescribed the correct thing, then they wouldn't see an alert, which really gets their goat up.

Software developer staff 1
Because we always recommend customers, especially if they've not had any decision support before, to go very small. This organisation has turned everything on and not even looked at what they've turned on, they've got duplicates. Oh, it's just a nightmare. So we're having to go and help and sort those guys out. But you just think, wow, that GP has never seen anything before, he's now having multiple pop-ups of sometimes saying almost saying the same thing, and their quit count is huge. You don't want that for a new customer…

Software developer staff 2
R: Well, I think there's a two pronged responsibility if you like on that. So there's the customer responsibility, and I mean CCG and the profile manager in terms of looking at the content and making sure it's relevant for their local domain, and the account manager's very much support with that in terms of quarterly reviews and trying to highlight where messages might be over alerting, why that might be, specific practices, the message generally, things like that. And then I think we have a responsibility to obviously produce new content and new guidance if released or if things are updated.
And there's quite a big review process that's continually ongoing, a triage process if you like, of key documents, NICE, NHRI, that sort of thing that we review. And we have to review them because we've got existing messages, and that might result in no change because the guidance hasn't changed dramatically, it's just been updated. Or we may identify messages that need minor updates, or we might identify messages that need retiring and new ones in their place. So that's a big undertaking that the content team do, and I think we've become smarter at that and more timely. We've also responded nationally to the product shortages that are affecting a lot of patients, pharmacists, GPs, when drugs are not available. We try to produce content to support where we know it's national and it's a very difficult picture because it's not always national.

Software developer staff 2
R: Yeah. So there's little things that [inaudible 0:24:03] the little things add up to this. So one of the things we try to encourage our teams to actually actively seek feedback from their end users. Often it's kind of like, well, if they don't say anything then it must be okay, rather than actually proactively prodding and seeing if there are things that they can improve on. So challenging them really about whether they've got mechanisms in place, maybe doing an ad hoc survey, although they tend to be self-selecting in that you just get a bit of a Marmite effect, you'll either get the people that love it or hate it or in between that respond. Or making sure that if they've got teams based in practices, if they hear things on the ground that they know how to feed it back, and also kind of close that feedback loop so if they do get any feedback and they make a change actually feed that back. So (name of CCG) are quite good at that, they try and actively promote that they might have turned off messages that were highly alerting, and it gives the practice a feeling that it's not just something that's being pushed at them and they're not interested in their experience of it. So that's one thing. Tinkering with messages, so turning off ones that are highly alerting or deciding to do some education around them or asking us whether there's something we can do to make the message more specific. Internally we're also looking at that. We've got a mini project going on about the wording of messages, can we make them more succinct. We realised that less is sometimes more.

<u>CCG Staff</u>
CCG Pharmacy Technician 1
R2: So in the end we were faced with a decision where some GPs were getting very close to deactivating the whole system, and I think one or two actually did and said I can't cope with this as it is. So (R1) and I did a piece of work and we stripped out many of the simple recommendations. We just basically deactivated them. Because we had to, because there was danger of throwing the baby out with the bathwater and losing the whole functionality. So anything that would effectively save 10p a week or whatever we just stopped all those. At the sacrifice of some potential savings for keeping it working, because there was just too many things built into it. One of the ongoing criticisms is that they still try and put too much into the system, and so we have to go systematically through all the recommendations every month and decide whether this is justifiable or not.

CCG Pharmacist 2
I just think we put real thought into what we enable, what we think would be useful and we go at a slow pace rather than sprinting to get everything on, that actually would just irritate because it's over, you get pop ups, you don't just get (the CDS)pop ups in your surgery, you get all

sorts that pop up. So I think we're just very, we just think things through, what would be useful. And I think that's probably the reason why it's accepted better, because they are more likely to accept things because it's more, you know, it's more useful than just something you can't… If you enabled everything on the profile well it's, I don't think that's a really good way of managing it because you need to check in detail what, if it actually is useful for your population and if it sort of complements the kind of work that we're aiming to do.

CCG Pharmacist 2
Plus, we had more guarantees that things were triggered to more of an individual status, whereas the original software we had, the messages that we put on, whether be it cost, quality, whatever, it would flag all the time, so it wouldn't be individualised to a person. And we found a lot of GPs and clinicians would get pop-up fatigue; they'd get so fed up of it all.

CCG Pharmacist 2
R: …but there was a bit of a mention of, oh what about the alerts that are already coming? Basically I think the way it was sort of sold was, it won't duplicate, the aim of it is not to duplicate stuff and have alert overload and work fatigue, sort of thing.

CCG Pharmacist 3
And one of the key things for (the CDS)is for the prescribers not having message fatigue. Because if they get message fatigue, they're going to switch off. So they accept or dismiss but they're not looking at it or reading it. So we're trying very hard not to have message fatigue. So the (the CDS) Editorial Board are very clear about that, so if we send a message and they feel there is already an adequate message on the system, they won't activate it and they will tell us why.

CCG Pharmacist 4
My view is we're paying 300 K for (the CDS), use it to its maximum potential really. What prescribers don't want is a formulary message then (the CDS)message, then perhaps a protocol trigger if you're thinking about the phenytoin valproate issue recently. They just want one message, and they don't want this message overload, message fatigue type things.

CCG Pharmacist 5
R: Most people had decided on the same safety messages and most of the discrepancies were around which formulary messages you turned on and which brands that we were using. Views about message overload were different in different CCGs. So, because [place] wanted more of their messages to be seen by the GPs because they didn't have as much face-to-face contact, they tended to turn more on […] so they had all of their formulary messages turned on so all of the drugs that you couldn't prescribe, say, were grey, they'd get a message for all of those. Any drugs that had a shared care protocol, they'd get a message. So, everything, whereas other CCGs had complaints from their GPs saying, we're getting too many messages, we don't want to know if it's got a shared care protocol because we already know it's got a shared care protocol […] So, we had to have a little bit of discussion and we'd come to a compromise about which ones we turn on…

CCG Pharmacist 5
I: And you talked about making the alerts timely and relevant for the GPs which is a, yeah, I mean, that's something that we've picked up on as being important. Some GPs, not necessarily within this area, but certainly some GPs around, you know, they've just, sort of, moaned, as GPs do, moaned about alerts. Too many, far too many, loads of stuff all over the place, we're getting too many. And one of the things I've...it's...and there's not just alerts coming from Optimise, there's alerts coming from [inaudible 00:10:31] as well.
I: I know, they come from everywhere, yeah.
I: So, how do you make it that the one, you know, that the important safety alert, how prescribers...you know, the PINCER alert that's embedded in Optimise, how do you make it that that GP sees that alert as being timely and relevant?
R: I don't think you can change the look of the message to make that one stand out against all others, so the feedback we've had from GPs is, if they haven't heard about it from elsewhere, they generally ignore the message. So, I think the message to our team is (the CDS)alone won't deliver an action on that safety message. But, if we back it up with an alert to GPs saying, look, sodium valproate, you need to counsel your patients who might become pregnant, you need to stop it, you need to do this, that. They then have that knowledge and (the CDS) is a reminder. And that's where, I think, (the CDS) works best, where the GPs already know the information, the message pops up and they go, oh, thank goodness that popped up because that stopped me from doing this.

CCG Pharmacist 5
I: Yes, I mean, I can see that. But, you said that you feel that the safety ones, the cost saving ones are not getting a look. Because one of the things that I was thinking was that, from what we've been finding is that, you know, where this, you know, you're getting constant bombardment of cost saving, and often GPs will say, oh, it's just to save a penny. And...
R: That's when we try and limit the number of cost saving messages that it gets, so we try not to turn on the really low value ones. And yeah, I think possibly, the most valuable ones we perhaps wouldn't just do as a simple message, we'd put an explanation with it, so that you could put in the titles something like, massive cost saving all over it], I don't know. Yeah, but most of them are fairly average sorts of savings and they'll just accept it. So...

CCG Pharmacist 5
R: Yeah, so that would be good. And we also look at messages that have a high hit count and pulse them if...so, in fact, probably the only example of that is the self care messages. So, there's a whole raft of messages where you tell patients they really ought to be buying their own medicines for that condition rather than using NHS resources. And some of those are extremely high hitting, like the emollients, so for dry skin, the creams for that, that's huge message numbers. So, what we'll do is we'll turn that on for a few months, and then we'll turn it off again to give them a break and turn something else on instead. So, those big high hitting messages for self care, we cycle through them, and so they'll only have two or the high hitters on at any time. And we'll do it seasonally, so we'll have they hay fever one on it a couple of months when hay fever season comes back. And yeah, so that helps as well.
I: That's an interesting way of doing it, because that's avoiding that fatigue.
R: We try and avoid the fatigue, yeah.

CCG Pharmacist 5

I: I mean, in both those, sort of, bits of the, you know, it's not just feedback is it, the relationships you have with the GP, it's the the relationship you have with the software developers. What are the things within those relationships that are really like, key to keeping it going and, you know, avoiding GPs just switching everything off or avoiding the CCG saying, no we don't want something?
R: I think accuracy and quality, and understanding of what we're actually trying to achieve. So, yeah, if we ask for a message, we need to know that they absolutely understand their side of it and technically, this is going to work. But also, clinically, checking that what we're asking is actually clinically relevant, and being able to have that professional discussion with them and know that they do actually understand what we're trying to achieve and what national guidelines are out there really. So, I think yeah, quality of staff and their knowledge is really important. And yeah, the understanding of practice pharmacy and general GP practices and how they work and how little time they actually have.

CCG Pharmacist 5
I: I don't know whether it was yourselves or whether it was somewhere else, people were saying that, sort of, usu..Yes, are the CCG…either yourselves or some other CCGs were talking about the using (the CDS) for specific things. And I think, and maybe that's also, I've had that come down from (software developers) as well, have you done that, or have you thought of using it in that way?
R: Yeah. So, the example that a new type of message, I don't think we really twigged it was a new type of message, but splenectomies, so people who've have their spleens removed need vaccines to prevent infections in the future. And so, we've done a big piece of work in(name of place), where we've audited to see if practices do give those vaccinations. And it showed that, actually, they weren't always giving what they should. So, we wanted a message to check if somebody's coded as having a splenectomy, we wanted the message that (the CDS) check which vaccines they'd had and give a warning that you've got a splenectomy patient, they're missing this one, this one. So, they've done that for us, which is really helpful. So, it's backing up the work we've already done, we've already trained the GPs, they know that that's what they should be doing and hopefully, this is a reminder to actually, oh, yeah, I need to do that, I need to book that patient in. And it's clever enough to also pick up patients who declined it. So, you won't get a message if the patient has been offered the vaccine but they've said, no. So, we've given the GPs a raft of codes to use to say, no, you're not having that one.

CCG Pharmacist 7 and CCG Pharmacy Technician 2
R1: I've not heard that. I think because it's…because of the mechanism for triggering the message it's a lot more targeted than Script Switch was. So Script Switch we used to get that because, like, if you put one on for an antibiotic it'd hit every single issue of antibiotic, whereas with this it might be…you know, you can target it by age, you can target it by condition. So the trigger points…so you're actually seeing…the practice is probably seeing less. It's not that sort of blunder bus approach, if you know what I mean, it's a bit more targeted. So I think that probably does help a lot, because we did with Script Switch, have people moaning. In fact the last one switched off, didn't they, one of the practices?
R2: Yeah.
R1: Whereas that's not been the case with this, so...

CCG Pharmacist 7 and CCG Pharmacy Technician 2
R1: Yeah, absolutely, yeah it makes it more… I think it puts people off if they see a lot of…you know, a number of paragraphs, they're not going to go through all of that. It's got to be...
R2: You need it punchy.
R1: Yeah, definitely.
I: And by and large the ones that are accepted, do you think they are like that?
R1: Yeah.
R2: I'd say most of our messages are punchy. I don't think there are ones that go on and on and on. We used to, didn't we?
R1: No, we do deliberately try and pick, you know, shorter more to the point versions.
R2: Yeah. I think in our previous one, to make sure we had all the information there where we were coming from, because we were hand typing it, you kind of put more into it.

<u>GP Staff</u>
GP Pharmacist 2
R: So, you have, like obviously, (GP CLINICAL SYSTEM) has got flags anyway, you know, safety things, they have categories, you know, high severity, medium severity. So, you've got (GP CLINICAL SYSTEM) software telling you what to do, and then (the CDS) also, so there is a lot of sensory input, I suppose, which clinicians don't like, even though they're there to help them or make them safer[…]

I: What do you think the effect of all those alerts then, is going to have upon their use of the system, or people's use of the system?

R: You might ignore the more important ones obviously. So, you go through that, click, click, click. And you don't read it, which obviously, if it's a safety thing, that's really important.

<u>Sub theme 3b. Flow of information between different stakeholders and stakeholder groups</u>

<u>GP Staff</u>
GP Pharmacist 3
I: Yeah. When you talk about that, you trust that you've got those people honing it, as you say, what sort of communication is happening between you and the medicine management team, do they have conversations about (the CDS) to you?
R: No. I will be honest, no. Not in the context of what are we going to do with (the CDS). We get, I get on famously with our local medicine management pharmacist, he's a great guy and he will give us fortnightly, monthly updates of new changes or drugs, not related directly to (the CDS) but we know what's coming through, whether it's a brand switch or things are out of stock. But not directly from (the CDS).
I: Yeah.
R: And we wouldn't have a bulletin to say this is what's happening to the software next month.

<u>Sub-theme: 3b i Collaboration and engagement between CCG and general practices</u>

<u>Software developer staff</u>

Software developer staff 1
And GPs, they can be very vocal but it just…yeah, and have a lot of useful things to say. And it does seem crazy that we know some of the functionality issues that they have, like too many clicks, but if they felt listened to, then yes, perhaps they might be more engaged to vote on a System One button to, you know, change certain functionalities within…but at the moment, if they don't feel they're being listened to, they won't engage. So engagement is so important really.

Software developer staff 1
But you'll get more engagement and there'll be better acceptability if the end user feels they've been listened to and gets that engagement. Which is why sometimes I think maybe doing a feedback or a survey with them would be useful, because then if they did something, we listened and we acted on it, they're like goodness gracious, I've actually been listened to. And GPs, yeah, they like that. But again, feeding back more. I really try and encourage MM teams to give each practice (the CDS) data, you know, these are your savings you've made, these are the best practice…you know, don't use it as a stick to hit them with, look at these 20 black drugs you've prescribed, what are you doing. That would just get their goat up. I know, I've been there many times.

Software developer staff 2
R: Yeah. So there's little things that [inaudible 0:24:03] the little things add up to this. So one of the things we try to encourage our teams to actually actively seek feedback from their end users. Often it's kind of like, well, if they don't say anything then it must be okay, rather than actually proactively prodding and seeing if there are things that they can improve on. So challenging them really about whether they've got mechanisms in place, maybe doing an ad hoc survey, although they tend to be self-selecting in that you just get a bit of a Marmite effect, you'll either get the people that love it or hate it or in between that respond. Or making sure that if they've got teams based in practices, if they hear things on the ground that they know how to feed it back, and also kind of close that feedback loop so if they do get any feedback and they make a change actually feed that back. So (name of CCG) are quite good at that, they try and actively promote that they might have turned off messages that were highly alerting, and it gives the practice a feeling that it's not just something that's being pushed at them and they're not interested in their experience of it. So that's one thing. Tinkering with messages, so turning off ones that are highly alerting or deciding to do some education around them or asking us whether there's something we can do to make the message more specific. Internally we're also looking at that. We've got a mini project going on about the wording of messages, can we make them more succinct. We realised that less is sometimes more.

<u>**CCG Staff**</u>
CCG Pharmacist 3
I: In terms of communication between yourselves and the practices about (the CDS)and about the various reports, alerts and stuff; I mean last time you talked about how important that communication was for the implementation of (the CDS) between yourselves and the practices; is that still the case and is that still ongoing and…?
R: That's, that's okay. We did a survey. We don't send out reports on a regular basis because we didn't feel there was a need, and the practices didn't want them; so we do report intermittently or if there was a specific request.
I: Right. Yeah. So would you say that the amount of communication you're having with practices is not quite as great as it was at the start, or has it evolved or changed since when you were in that first information stage?
R: It's [voices overlap 0:16:45] [as such 0:16:45] it's very minimal, but that is because we don't feel there is a need for it and there hasn't been a request for it as such.
I: Right. So it's coming from them as well. Yeah.
R: Yeah. There's not the request for it.

CCG Pharmacist 3
I: Yeah. I mean how useful did you find that feedback from that survey?
R: It was useful, because it's allowed us to actually kind of monitor where we are and how people generally think about it; and it also enables us to actually look at the positive comments and look at how we could expand on those positive comments and how we can highlight that to maybe other practices that didn't feedback quite such positive comments. [deleted – inaudible].So we had that for new registrars, NMPs in particular, we had that across the board; but that might have been from a practice whereby one of the GPs said it's cumbersome and it slows the system down.

CCG Pharmacist 5
I think the GPs want somebody to talk to about it. You couldn't just put it into a practice with no support at all. They'd be like, well, if I'm getting a message and I don't understand it, I need somebody who's a representative to say, I don't get that, is there something I'm missing or is that message wrong? So, I think they need a personal touch and especially right at the beginning when it gets turned on. I think they need to know that they've got back-up because if it all goes belly up and they're getting messages left, right and centre, they need somebody there to support them. Otherwise, I think they'd just turn it off.

CCG Pharmacist 5
R: Yeah. I think they could probably improve GP involvement. At the moment nothing's really changed, it's the same as it was. So, they give their feedback either by, well normally, just by speaking to their practice pharmacist, so there's a CCG pharmacist attached to every practice, and the GPs don't hold back if they've got a problem with Optimise, they do tell them, so that gets fed back to us. So, we've got a query log that all of our pharmacists can input into, so if they get a comment from their practice, they'll whack it on the query log so that we can review it. And then, GPs have the option of the rejection. So, if they're going to reject the message they always get the option to write a message to us and explain why, and we keep an eye on the rejection messages every month and just see if anything, if there's any common themes or any particular messages that are annoying people. But yeah, I think, like you say, it's there's so many messages popping up, I don' think they always twig it's Optimise. So, yeah. And I don't...

CCG Pharmacist 5
I: But, yeah, so you're getting that feedback. I mean, is that only through the, you know, they talk to the pharmacist, the pharmacist comes back and talks to you, there's no, sort of...?
R: No, not at the moment, there isn't really a...

I: A user forum or something like that.
R: No. I don't really know how to do that. But it's definitely something that would be useful. It would be quite handy if there was something actually in the system that they could feed into.

CCG Pharmacist 5
I: But, I mean, is that a lot of feedback you're getting from there, or is it just occasionally they're writing it.
R; I think if they feel very strongly about something, that's the feedback we're getting. But, to be honest, I think their biggest bugbear is when it goes wrong. So, when they get connection errors or they get messages saying that (the CDS) hasn't worked because of x, y, z. We seem to be getting more of those at the moment and I don't know if it's connection issues or if it's something with (the CDS) that's causing more problems. So, yeah. But that seems to be their biggest bugbear.
I: If it's just not working, it's [voices overlap 00:24:52].
R: Generally, most GPs don't complain that they get too many messages. And I think many realise that if they're getting a lot of messages perhaps they need to question whether or not they're coding their patients properly. You know, if they're actually, perhaps not up to date with what they know. But...

CCG Pharmacist 7 and CCG Pharmacy Technician 2
I: I was thinking about the…you know, we were talking there about your relationships with FDB. What about then with the practices, you know, what sort of…? You say, you know, the alert rate's been really really good here. What sort of feedback or any feedback have you been having from practices? How do the practices like it as a system, if you like?
R2: I wouldn't know, to be honest, I've not done any work recently on that.
I: But you've never heard anything bad, have you?
R1: No, we don't [voices overlap 18:49].
R2: No, this is what I go off because we've not heard anything bad. If I'd heard something bad, then obviously I'd need to do some work but… They also have…when they reject things, they have an option to comment about why they're rejecting, so those messages can be quite useful to look at. But yeah, I don't know how useful any feedback would be, to go there and find out what they think about Optimise.
R1: I mean, by now most of them expect some sort of message to come through the messaging system because it's like coming up for nine years that they've had it now, and certainly when we were looking to change it from Script Switch to (the CDS)or considering whether we were going to change or not… You know, because one of the options obviously we had was, well, we don't have any system, but no-one didn't want any system; they all said they wanted messages. So that was…there was a sort of consensus. I think, based on the time they've had some sort of messaging system, they all kind of wanted it, and they all expected it. They don't kind of think, why am I getting this message? They just expect it.

GP Staff
GP Pharmacist 1 and GP10
I: And we talked something about relationship with CCG and a very good relationship with the CCG pharmacist here. What other…how do those relationships help with prescribing in general and with Optimise? What's the…?
R1: Well, I think it's because it's always I suppose high on the agenda in people's awareness. Because I'm here all the time, every week and then I catch up with(name of CCG pharmacist) regularly. So, I'm…
R2: And we can contact (name of CCG pharmacist) at any point if (name of R1) not around…
R1: …on the system.
R2: …so we have a good, close working relationships.
R1: Yeah. And (name of CCG pharmacist) will – if something comes up that's important or urgent – she will message us all. So it's always in our consciousness I suppose, the issues that are…
R2: …around.
I: And when you feedback, would it be through (name of CCG pharmacist)? It wouldn't be beyond that [voices overlap 24:27]…?
R1: No, so (name of CCG pharmacist) our CCG medicines management pharmacist, so we do still…
I: And then she would feedback to…
R1: She would feedback…

GP Pharmacist 3
I: Yeah. When you talk about that, you trust that you've got those people honing it, as you say, what sort of communication is happening between you and the medicine management team, do they have conversations about (the CDS) to you?
R: No. I will be honest, no. Not in the context of what are we going to do with (the CDS). We get, I get on famously with our local medicine management pharmacist, he's a great guy and he will give us fortnightly, monthly updates of new changes or drugs, not related directly to (the CDS) but we know what's coming through, whether it's a brand switch or things are out of stock. But not directly from (the CDS).
I: Yeah.
R: And we wouldn't have a bulletin to say this is what's happening to the software next month.

GP Pharmacist 3
I: Yes. That's interesting because that relationship, because you are seeing him regularly, you have that relationship with the MMT, I think it's quite interesting in terms of how that's maybe helped or doesn't help with using the [voices overlap 08:27].
R: Yeah. There has been, I've seen situations where there's been relationships of attrition between medicines management and practice pharmacists.
I: Yes.
R: That the practice pharmacist really should be following the medicines management role, and really, they are part of the practice. It's practice work.
I: Yes.
R: We are there to do the clinical work, not the drug switching work or the budget saving work.
I: Yeah.
R: But here, the medicine management team fully respect what I do. Which makes me want to help them with, you know…

GP Pharmacist 3

R: Fairly, yeah. That's a fair assessment. They are coming in from a financial assessment, also with PINCER, safety always. In some cases, they don't consider the patient. Particularly not in this area, they are very very good. In other areas where I've seen blanket switches being made by medicine management, it's been a lot of kerfuffle going on.

I: Yeah.

R: And I've had to, as a practice pharmacist, then pick up on that and bring the patients in, have a chit chat, and tell them why the changes have been made, are you happy with it, would you like to be changed back.

I: Yeah.

R: We see it differently. In this area, we complement each other better because we talk, and there is no attrition, from my professional relationship with medicines management.

I: So, a lot of that is based around that relationship.

R: Yeah. I think that relationship is most important.

I: Yeah.

R: Especially when medicine management understand what clinical and practice pharmacists are in practices for.

GP4

I: Yeah. I mean has there been anything come down, changes from Medicines Management at all, have they had any further contact with you about it or do they keep regularly in touch with you?

R: No. I'm not aware of anything. It may be that I've missed the odd email or something but I can't, I'm not aware of any direct contact. We do now have a pharmacist role where the clinical pharmacist that we appointed at the beginning of this year, that we employ ourselves rather than a CCG funded role, and so she, I did talk to her briefly about it in anticipation of this and I know that she's been aware of being able to access things that we've, you know, the log essentially, so the things that we have overridden or the list of things that we have actually changed as a result of (the CDS) messages. So, she must have had some level of communication with the CCG at that level to be aware that she can interrogate our data in that way. [Voices overlap 02:05]

GP9

I: Yes, so they're coming as well. So also the other thing I was thinking around, I don't know whether we talked about it last time, I think we probably did, what sort of feedback do you have with the CCG around Optimise, around prescribing?

R: We don't have any feedback from around prescribing. We get regular feedback in terms of what they want us to do or what we're not prescribing or what we shouldn't be prescribing in terms of our budget and safety but nothing with Optimise.

I: Yes, and have you got a practice-based pharmacist or a CCG pharmacist?

R: CCG pharmacist.

I: CCG pharmacist but not a practice-based pharmacist.

R: Not a practice-based.

I: So the CCG pharmacist, do you feed back to them or do you feed back directly to other people at the CCG?

R: In terms of the prescriptions, anything to do with prescribing, we can just contact her.

GP12

…so (name) who is the CCG pharmacist has emailed round and said how are people finding (the CDS) and you know, that email's gone round to our GPs and we have been able to say that and we've been able to give that feedback to them. I think we work pretty well in communication with our CCG and actually feel that they are pretty responsive to the feedback.

GP12

I: So do colleagues basically are feeling...

R: Do they feel the same, yeah, I think everybody feels it. I think that there also, I mean, you can in (GP clinical system) you can switch off the drug alerts. Now that we've found the place where user preferences can actually switch off loads of alerts, we're all sitting here, you know, is the decision which we are making in the practice is which alerts in (GP clinical system) are we going to switch off, which is safe to switch off and which aren't, we don't know, and it just, you know. I think one of the colleagues has switched off all of them because she actually thinks, you know. And it maybe that what we need to do is switch off all the (GP clinical system) ones and just use (the CDS) if (the CDS) is going to give us better alerts but we haven't worked that... Nobody has advised us, nobody said, nobody from the CCG has said this is what you need to do if you want safe prescribing. It just sounds ridiculous. It sounds ridiculous that at the last partners meeting I'm saying, (name) [19:55] can you work out which alerts we should and shouldn't be using. If you're the national person for safety, and yet I don't even know in our practice what we, you know, what's safe anymore. And nobody seems to, you know, nobody said to us I don't know what other practices are doing, it's crazy.

GP12

R: No one to talk to us about safety alerts.

I: Have you had any sort of, have they talked about why you've moved, about the moving from (GP CLINICAL SYSTEM) to (GP clinical system)? Have they discussed any of the (the CDS) stuff within that or...?

R: They've not discussed any of the (the CDS) stuff within that. I mean, we do have a pharmacist in our practice who is 60 percent, contract is with us. We're 40 percent subcontracted to the CCG, so she's 60 percent in our practice. And she's very good and she's done some fantastic work just in teaching receptionists the prescriptions and then just making the system kind of work as best that it can...

I: Yeah, precisely.

R: ...looking at prescriptions. And, I guess, we haven't, this is maybe the start. Maybe our last partners meeting was the start of us saying we need to look at the alerts now, you know, we need to get this right.

I: Yeah, absolutely.

R: But it needs, you know... Have other practices done this, I don't know, does someone come up with a unique this is what, this is the alerts we should switch and switch on. My guess is it's probably up to the preference of the practice and nobody is going to take responsibility for saying, you know, they depend on [voices overlap 23:16]

GP14

R: So, I've always been pretty open to getting nice suggestions about how you can do things, A; either more safely or more cost effectively or whatever it might be, again we're going back to the fact that they've been around for a long time.
So, I think from my personal perspective having been a GP for a long time, I'm also the prescribing lead for the practice, so, I'm all very much for good quality prescribing and safe prescribing. So, for me it was a fairly smooth transition it was fairly self-intuitive as to what you did or didn't do in terms of accepting suggestions or not. For me it was pretty easy despite the lack of knowledge about when it had changed over or why.
I: So, it's interesting they were no conversations, CCG or anything from, just imposed in volume.
R: No, just, these things happen, that's not out of the ordinary, could I say. So, yeah things just happen. It's the same with new hospital services or new forms that we're meant to be using for referrals or end of life care. They often will appear before you have any training or knowledge of it whatsoever. Then if you're lucky maybe there's a launch event six months after you've already started using it. So, yeah, it didn't seem particularly well organised when this appeared but again, as I say sometimes there are extenuating circumstances, so, maybe I was away on a week where somebody came and talked about it, but yeah don't know.

Sub-theme
3b.ii Communication, collaboration and engagement - MMT, CCG and Software developers

Software developer staff
Software developer staff 1
So we try and get the GPs engaged early on because what we found is, if an organisation just buys it and chucks it out, GPs are like, hang on a minute, you know, yet again you've bought something that's affecting us and we don't have any buy-in. Whereas if we go and we push the fact that it's best practice and it won't pop up a lot, and if it pops up, there's normally a reason because we've looked at READ codes, we've looked at past medicines. So we really want them to, you know…and a lot of…most GPs think actually, yeah, it's really useful, but you'll still get the ones that just hate it regardless and refuse to…but you get that everywhere

Software developer staff 1
So it's about getting the GPs more on board, which is why when we go live, we try and do all the GP events. I've done specific events before just on (the CDS), do a live demo. If the post-pilot practices want a post-pilot meeting not just with the medicines management team, we can do that. If they want some data…because some that are quite engaged, will say, well, I want to know how I've done in the pilot, we have that ability to give them the data to do that. Yeah, we're just redressing that. And actually, going forward it's about more touchpoints with our customers and things. For instance, I'm off for a week and a half now, but it's like I worry about my customers. So I emailed them all today to say I'm not here so don't panic, but I can…I've got people to look after you when I'm not here, you know. But those practices, when we go and see our customers on their what we call the QRM, the quarterly review meeting, CCG…well, GP feedback is on our agenda and we will say, have you had any feedback, what…because we might need to do further engagement, you see.

Software developer staff 2
R: Yeah, I think so. I think probably that user group that you came to, although it didn't materialise, I think at that time the teams were almost suffering from our own success at that point in that we'd acquired a large number of customers and we were running to try and keep up a little bit. So although customers didn't actually express any discontent at that user group that you were at, we were concerned in the background that we weren't perhaps as…you know, our level of service wasn't as good as we wanted it to be at that point. But I think we've kind of matured in the background to accommodate that. And people are using the solution in more innovative ways, and we're beginning to partner with different organisations. Well, I think partner is a bit of a strong word at the moment, but looking to form partnerships with…

Software developer staff 2
R: Probably this past year the main things have been around the formulary tool the CCGs a bit more control around that. And then, something that we call the multi profile tool, which is really just acknowledging that we know that CCGs are coming to work together, either merging or coming as an SDP type scenario. And yet they've started off with individual (the CDS) accounts and profiles we call them. So how do they suddenly become one and how do they manage that, and actually is it that simple? The multiprofile tool is there to either help the align or to help them manage having more than one profile and knowing that they've got to have some differences. Because these big organisations are still having localities, they have more than one acute trust [voices overlap 0:39:18].

CCG Staff
CCG1 Pharmacist 1 and CCG Pharmacy Technician 1
R1: We have a three monthly meeting with the programme…I don't know. What's the title?

R2: He's our regional account manager, and he comes up with somebody who is far more technologically au fait with the system. However, all the software people based south of here, in [name of place] I think they are, and so the opportunity to get all this cohort of people up here, 300 miles away is very difficult. So we give them messages and we highlight some of the issues that we are finding but we often find that we don't get a resolution following that meeting.

R1: The resolution that we desire.

R2: Yeah. We get their version. Or as compromise we often find is what we see rather than okay, we're on that, it'll all happen. But it has highlighted a number of communication problems where we thought things had been resolved and they say actually, no, we haven't picked that one up. We found quite a or faux pas recently where somebody had promised that something would happen automatically and it turns out that that individual is not authorised to give that promise, so we have…

R1: And had left his position.

R2: Yeah. We had been under this misapprehension for the last couple of years at least whereby us thinking that changes made to the website, to [local formulary]would automatically be transferred into Optimise, and that, despite the reassurance, turned out it wasn't happening.

CCG Pharmacist 2
I think it's just a different kind of support, because it's easier to use the system now. It's a lot, it seems a lot more efficient, but also I get quicker responses to queries and things like that if I'm not sure. So I think that's better. I don't know whether they've invested more in staff, I don't know. I'm not sure, but my accounts manager is a lot more hands on with this one. But yeah, I just, I just think it's just, there's more support there really.

CCG Pharmacist 2
I: I mean that sort of collaborative working then with (the CDS) themselves is quite interesting in so much as it…I mean I know there's been, from what other people have said, there have been delays in changing things but that's really interesting as to how that's developing.

R: Yes, sometimes I think we've got our ear to the ground in certain specialist areas knowing what's going to come before they, sort of…because they're going by whatever they think national guidance currently says. We're aware of rumblings, you know, whether it's a national project or local that we're doing in conjunction with that. So we know what's coming and say, actually, that doesn't really match the local message we're trying to get across to our prescribers so it would just contradict that. We decided to switch that one off until they reviewed it.

CCG Pharmacist 4
I: Okay. And in terms of working with [software developers], how does that operate?

R: Well, (name) always very good. So in terms of the account manager I think our relationship with and access to (name) is fine. She does seem to be able to get things moving when needed. I think they've had resourcing issues, so we have had issues in terms of getting messages authored in a timely fashion. This is one of the fights we have with the formulary. So with the formulary we have to wait for that decision to go through the area prescribing committee, then we have to wait for it to be ratified, then we have to wait for the minutes to go online, and only then can we amend the formulary. So that process can be anything up to six weeks. So there's always that period of time when something's actually on formulary but nobody knows about it.[…] So you've got that process and then you've got the (the CDS)authoring on top of it which could be up to another six weeks. So you've got three month lag where the formulary doesn't necessarily meet Optimise, so that's something that has caused a little bit of friction.

CCG Pharmacist 3
R: There were problems with…I can't remember the origins of the problems, but there was a fault with the software due to the change I think TPP and (GP CLINICAL SYSTEM) had made, so it did go down for some time, I think it was about the better part of a day. TPP went back online pretty quickly, but (GP CLINICAL SYSTEM) didn't; and we had to wait for an update of (GP CLINICAL SYSTEM) which took them about four months to implement, because even though it was an upgrade that was needed on (GP CLINICAL SYSTEM), which needed to implement the (the CDS) software, they don't do updates for just Optimise.
I: Oh, right.
R: There's a regular update process for the whole clinical system, so they would have several things in there. And as they update they tend to roll it out, but they don't do everybody at the same time, they tend to roll it out to a small group of practices and then roll it out to more practices, so it's a phased approach.
But as a result of problems that happened with other updates they didn't roll it out across all practices until in totality four months. As a result we had a couple of practices that switched off.
I: Oh, right.
R: So we actually still have two practices that are switched off, two (GP CLINICAL SYSTEM) practices that are switched off. And both of them have not expressed a willingness to reactivate it. And we also have another System One practice that have switched off.
I: Did they give any particular reason for that, or was it just that…?
R: The two (GP CLINICAL SYSTEM) practices. One of them it's because they had an internal IT problem, and in that their system is very slow anyway, so as a result of this update on (GP CLINICAL SYSTEM) they felt it would slow it down a bit further, they were not willing to have anything which might contribute to it, because they'd already had a lot of IT problems; so what they said – because I think it's a lot to do with connections and the cables and whatnot – but they did say that when they have sorted that they would be willing to have (the CDS) on again. So that was kind of acceptable to a degree, because it's not only (the CDS)that is affected, it's affected other things. That's in (name of place). With the second (GP CLINICAL SYSTEM) practice, which is within my patch, they don't feel they need it

CCG Pharmacist 3
I: Well, yeah. Precisely. One of the things we talked about last time was about communication being really, really important. And two aspects of that from your point of view: one the communication with practices; and the other communication with (software developers). If we start with (software developers) because you talked about reports you were getting from them, and the various facilities, extra facilities they've put into the system; how's the relationship with (software developers) worked? How's that information exchange, if you like, been going with them? How is that getting on?
R: Oh, that's fine, they are very accepting to things that we suggest, and also they do get back to us pretty quickly; and our account manager will run a specific report when we need them within a day or two.
I: Yeah. Good. And has there been any issues with any of that communication or information?
R: [Of course 0:13:47] there was a time the system was down, we did actually escalate that and have an extra meeting, and that involved the other people in FDB; but that was resolved and explained. But yes, that was a problem, with the system going down, and we tried to put something in place to ensure that things like that did not happen again; and also about how we communicated to the practices, so that we had [inaudible 0:14:24] like that happened, because so far we've not had a repeat of that.
I: Good. That sounds very positive. And so when that went down there was a day when the messages weren't firing; did the practices themselves, did they sort of say, oh, this isn't working very well? What was their response?
R: They notified us but what also happened was that there was a message sent by their provider, that is TPP and also (GP CLINICAL SYSTEM), that there was a problem on Optimise.
I: Yeah.
R: The clinical system providers switched off Optimise.

CCG Pharmacist 3

R: Yes. I think so. But I mean, as I said, at the moment it may not significant, but I just feel that extra support, that extra clinical input from (the CDS) I think is very useful, and it keeps us very aware of what is happening in the rest of the country.

CCG Pharmacist 5
R: I don't think they engage with the...
I: Engage really with general practice.
R: No, not unless there's a technical issue.
I: Yeah, or unless there's a particular thing that they need to go and do. And perhaps there should be, I don't know.
R: Yeah, I think they'd like to be. We've had a suggestion from our account manager at (software developers) that we invite a GP along to our strategic meetings. And I just can't help thinking that they'd be bored stiff in the meeting and most of it would be totally irrelevant to them. I think, probably, a better way of doing it would be a Survey Monkey or something, so we could get their feedback....

CCG Pharmacist 5
I: So, that's feedback with GPs. What about the other end feedback, and the partner work as it were, with FDB?
R: That seems to be quite good. So, the user group was really useful, that was nice to meet all of their staff. But we've got a really good relationship with our account manager, we know that we can...because she generally replies within a few hours to emails. Yeah, we know we can pick up the phone if we need to. So far, I think that seems to be going pretty well, and the content team, so, if we put in a request for a new message the content team are quite responsive. They did go through a phase of being quite slow, but they seem to have sorted that out now.
I: I heard that they were allowing you to write some of the current messages [voices overlap 00:27:16].
R: Yeah, so we can change the formulary messages now, but only the generic ones. So, we have a grey category for drugs that are non-formulary. So that, for an example, we can change the trigger list on that, so the drugs that are in the grey list, we can add those in and take them off ourselves now. But if we had a specific message for a particular grey drug that was more complicated, then FDB still author those ones for us. But that's saved us a lot of time, and probably them as well.

CCG Pharmacist 5
I: I mean, in both those, sort of, bits of the, you know, it's not just feedback is it, the relationships you have with the GP, it's the the relationship you have with the software developers. What are the things within those relationships that are really like, key to keeping it going and, you know, avoiding GPs just switching everything off or avoiding the CCG saying, no we don't want something?
R: I think accuracy and quality, and understanding of what we're actually trying to achieve. So, yeah, if we ask for a message, we need to know that they absolutely understand their side of it and technically, this is going to work. But also, clinically, checking that what we're asking is actually clinically relevant, and being able to have that professional discussion with them and know that they do actually understand what we're trying to achieve and what national guidelines are out there really. So, I think yeah, quality of staff and their knowledge is really important. And yeah, the understanding of practice pharmacy and general GP practices and how they work and how little time they actually have.

CCG Pharmacist 7 and CCG Pharmacy Technician 2
I: And how, you know, following that… I mean, I suppose to continue with some of that profile management stuff then, how much help do you get from (software developers) with that, or are they…?
R2: In terms of?
I: In terms of if you want to make any changes, if you want to put a new message on or…
R2: Yeah, they are there all the time so if you needed something changing there's already some…there's always somebody online that you can send messages to – you can ring them. It depends at what point of their cycle when they release the update, so if I asked for something new this week but they've just released one last week, I'd have to wait for a couple more weeks before they're ready to release the next one and then it'll be live. But even then I would have to enable it, it wouldn't just automatically go online.
R1: Because we've got to read through and sense check it and think that's the message we want to put across.
R2: Yeah, I think they're ace support, I do yeah.
R1: There's that thing as well where the…you know, they sent the…you know, when they used to send things through on the spreadsheets and then they changed the…
R1: Yeah, that's all gone now.
R1: Yeah, so it's better now.
R1: It's all automatic…well, it's all in like a saved…like a profile within the profile if you want to request content changes. So, before we had to fill out an Excel sheet, attach it to an email and it took a long time and then I found myself chasing lots of emails, whereas this you can see, it's like a list of which ones you've sent and at what point you're up to, whether it's been agreed or if it's been completed. And then I know I can go back and I'm like, right I know that's completed on this date, I can enable it if we're happy with it. So it's all on one page then rather than me having to attach documents and a…it's a lot more straightforward.

CCG Pharmacist 7 and CCG Pharmacy Technician 2
I: What sort of relationships do you have with them as a…you know?
R1: Just our account manager really, (name) she comes in.
R1: She's fairly regular, she comes in.
R2: Yeah, every quarter she comes in and she does a full…she shows us a full summary. Me and (name) usually attend, the other technician, who helps, you know, again with this, and from that we can. And we review what's happened in the last quarter, so any new ideas, anything new coming through, anything new that's coming through from them. So she comes and she gives us a summary of what's happened in the last three months, anything new that's come in…up and coming into the service, any ideas, or if we've got any problems then we can raise them there. But yeah even our quarterly meetings are shorter now than when they originally were because we're all more settled with it now.

<u>GP Staff</u>
GP Pharmacist 1 and GP10
R2: Yeah, striking a balance between enough for a highlight and allowing normal work to proceed, I think is always this tricky one, isn't it?
I: Yes.

R1: It might be useful, I mean, perhaps this would be more relevant in a bigger practice where there's more prescribers to have a report of perhaps…
R2: …what's accepted and why.
R1: The groups of the messages and the percentage of acceptance rate…
R2: And why.
R1: …within the practice…so that…I could see in a bigger practice with a lot of GPs that would be a really useful tool for somebody like me to go in and say, okay, well, look, there's this thing on this.
I: So, you never see your hit rate, your [acceptance really 27:52]?
R1: No. I'm sure that that facility is there…
I: Oh, yeah, I'm sure it is.
R1: …but we certainly don't get that kind of pressure.

GP Pharmacist 3
R: Then it's great. Then as an area using (the CDS), it would be a lot easier, but really, yeah.
I: So, those supply issues are…
R: They are very problematic and it's not tripping up the software but because of the speed of the way stock movement is at the moment with community pharmacy, the software isn't reactive enough. I think because the information isn't being fed back to medicines management and to the software writers in time.
I: Yeah. Around those, supply issues and so on.
R: Yeah. I mean, I'll happily go and see the medicines management pharmacist every Monday, which I do for a general chit chat, and tell him what's happening on my side of primary care, and he'll take a note, 'cause he's good like that, but whether he feeds it on, that I'm not sure.
I: Yeah.
R: Or whether he will take a note of it and then monitor it.

<u>Theme 4:The contexts in which (the CDS) is implemented and used.</u>

<u>Sub theme 4a: Outer Contexts - The varied contexts in which prescribing takes place in primary care.</u>
<u>Sub-theme 4a i: Patient characteristics</u>

<u>Software developer staff</u>
Software developer staff 3
I mean, that's a great point. So, acute prescribing, generally is done quite well, through (the CDS) we think. As in it feels like it's something that makes sense to a GP. Someone has come in with an acute problem, they need this drug for this length of time, and (the CDS) helps me do that […] The problem is with LTCs, are one, patients can have multiple LTCs. When they come into a consultation, it's really unclear, until you talk to the patient, why the reason they're there. And it may be a completely different problem, and actually that's not related to their long term management. So, I guess, we see the role of GPs as changing. So, it's almost like there's a stratification. There's the kind of easy, quick and dirty acute stuff, which they're always going to have in their inbox, like they're always going to have those types of patients. Those patients are starting to also be seen by non-medical prescribers. Then the more long term condition management, but the standard long term, I'm a person with diabetes, whose HbA1C is running at 7 to 8, I'm taking me meds, I do exercise, and I watch my diet. So, I just need an annual check-up, and if I have any issues, I don't necessarily need to go and see a doctor, I could see a pharmacist, or I could see a nurse practitioner. So, for those kind of patients, actually, a point of care tool like (the CDS) isn't very useful, because you're already going to be on the medications you should be on hopefully. So you'll find that we're probably not offering too much then.

<u>GP Staff</u>
GP Nurse 2
I: Yes, and in terms of those patients, then, does that have an impact upon how you might use (the CDS)? I mean do you think it changes if you get more alerts because of that patient group or fewer or what?
R: Probably more because some of the drugs that I generally prescribe probably within diabetes are still relatively new so there's always that caution around…so that might be why some of the guidelines… So, it could be that because they're still quite new drugs on the market, some of them. It could be that.

GP Pharmacist 1
R: So, my approach is probably slightly different because I don't do diagnosis on any acute things. All of the patients I see are chronic disease review, medication review, so I am actually more focussed… […]on actually dealing with those complicated things that the GPs don't have time to deal with when they're seeing the patient acutely because there's something actually wrong with them. You know, they're coming in to see me routinely because it's their annual review and that's what I'm dealing with. So, I can't think of an occasion where I've had lots of different (the CDS) things on one patient. It's more a case of where a thing comes up that you disagree with because of x, y and z else that's going on […] Then perhaps the multi-morbidity or the frailty or the social situation of that patient or whatever it is means that I'm not going to do what it wants me to do for whatever reason […] I've got to think about the patient as a whole, not that just individual thing.

GP Pharmacist 1 and GP10
R2: [Voices overlap 08:39] more appropriate for acute. I don't think it's not appropriate for chronic. My personal feeling is that I don't ever mind pop-ups coming up. Yeah, whether it's acute or chronic. The only slight rider to that is that I'm probably more likely to tick, not suitable for this patient, if it's something that's a chronic condition, that they've had something for a long period of time and I know it's not on anybody's radar and I know that it's not gone against guidelines and so on and then I'm likely to put, not appropriate for this patient. Yeah. So, I personally don't mind it. I don't think [inaudible 09:17]. It's minimal amounts really of interference…
R1: 'Cause you just…
R2: …in a day's work.

GP Pharmacist 1 and GP10

I: Would you see say on a chronic patient with multiple morbidity, where you've got 20-odd drugs or something. Would you see lots of alerts with them and would it be useful around that or…?
R2: Yes. [Voices overlap 11:48]…
I: …perhaps [voices overlap 11:48]…
R2: I think that's why I said, it's…you don't really want to take them off those chronic conditions because it does give you a heads up to remind you to just be checking that it's still appropriate in all the circumstances. That's my view.
R1: Yeah, and particularly from my point of view 'cause I will be tending to do the…
I: Yeah, tend to do the…yeah, precisely.
R1: …medication reviews, where you're actually…the reason they're sitting in front of me is to review their medication. So, then that's useful because it then makes you think, well, is that something that we need to change, is now the time to change it? Is that appropriate? So again, it's a good, just…

GP Pharmacist 1 and GP10
I: Yeah. And from a patient angle, are people presenting with more complicated issues than they were? Is that part of it, that as well?
R2: Not complicated but more issues that are running together. So more co-morbidities. Yeah. So not necessarily more complicated or more unusual presentations. Same presentation, same complications, but with co-morbidities which therefore makes diagnostic certainty and treatment much more difficult.

GP Pharmacist 3
R: Yeah. So, that's the only thing with (the CDS) is the big polypharmacy patients, it will try and throw up some recommendations but sometimes it can get a bit much.

GP1
I mean a more complicated patient is where it's actually in a way more useful […] If I'm giving one drug to somebody who's not on anything there's not many interactions so you're not worried about interactions, you're only worried about the pure drug side effects; but when they're on 15 different products some of interactions are very (inaudible) and very important to know them, so you'd tend to take…well, it's more useful in that situation.

GP2
It helps. It should be...helps but it does at sometimes it really hinders because so many...you know, if we are dealing with a patient who has multiple diseases who has 20, nobody should be, but we have patients who are on 20 medications and when you're doing the review it pops like all the time and I'm thinking okay, is there any way to switch if off by the time I finish this.

GP3
There's often as I'm going through a surgery and when I first did the other surgery I had over 300 patients on over 25 medications, and that is impossible for (the CDS)to help me. I've got to be really quite selective about what I do. Because if I've got 20 alerts up when I'm doing a medication review I cannot deal with 20. But I've learned to be a bit more selective about it, and I'll go for the low hanging fruit, and I'll perhaps do a little bit in the notes to say discuss such and such next time. Hopefully we get maybe less and less with time.

GP3
R: The differences is at (name of practice) we don't see it very often. We don't see the cost saving ones. We don't see the interactions. We don't see great safety warnings. And that's perhaps because of the work we've done on prescribing here, and also on our population. Our population are happy to do over-the-counter, for lots of things, and we work very hard I think on the prescribing here. We see it perhaps…I would say a third of what I see at the opposite extreme, which is a city centre, highly deprived, highly unorganised practice in some ways where we see the safety alerts all the time. It's almost universally it's on 90 per cent of the time, which is quite striking. The branch from here is in between the two. It has more cost savings coming up on it. It doesn't have any more safety warnings than here, but it has more cost savings. I think that's just a reflection of the population as well. […] Yeah. And also the mobility of the patients. The patients here tend to be very stable. Been here a while, been educated and have got a tight repeat prescribing routine. Whereas in the chaotic city centre with a population churn it is just not the same at all. So the (the CDS) works very differently across the three sites.

GP4
I: Yeah. And thinking of patients as well, are you thinking about, you know, to what extent are you judging what's appropriate for the patient to what the system is telling you?
R: I suppose that's where the overrides come in; sometimes I will just make a decision that I'm going to ignore it based on how I think the patient might react to that. You know, some patients take things on board very readily and they are with it and they understand how the cost substitutions are important, and you can explain that very readily and they are open to that idea, and also you know that they've got the capacity not to get confused about their medications, you know, things come in a slightly different box or it's got a different name or it's a completely different drug altogether but does the same job, then you might have confidence that they are going to deal with that. Whereas another patient you might think, well this is just going to freak them out; even if it's spelt slightly differently, they are not going to cope with that.

GP4
I: So, those sorts of considerations then are they important ones in terms of balancing that with the messages that are coming through.
R: Yeah, definitely. I mean I don't know, I think sometimes we as GPs perhaps just hide behind that a little bit, because sometimes it's easier just to have the status quo isn't it, and you can say, well I didn't tackle that with the patient either because I'd run out of time or [voices overlap 14:50] so I think sometimes the pharmacist brings that, and it's been interesting seeing how our pharmacist works now that she's seeing patients and doing her own medication reviews. She's much more thorough and she will challenge people a lot more and say, look you've been on this thing, I'm not sure you necessarily need that or you may be need something slightly different, and she will go through it in a more methodical fashion. I think we do probably in our consultations, sort of, go through things a little bit more quickly and make those decisions in our own heads rather than necessarily sharing it, but just make an assumption that this patient is going to give us some grief or be unhappy or be confused if we make a change. So, sometimes I think there is a bit inertia involved.

GP5

R1: Yeah, it is, that if you've got a patient with 20 different drugs, and back to the thing about, they've just lost their job, they're about to become homeless, they've got epilepsy, depression, anxiety, diabetes that's out of control, they've got 15 appointments, but they've missed the last ten, and they've got a safeguarding issue with their child, which is, I'm not going to say typical of my patient population, but it's not a million miles away from where we are. Then, talking about one particular tablet, is just not on their radar. There are much, much bigger issues for them. And yet, because investment has gone into Optimise, this is what's being put to us [...] It needs a fundamental re-think, I think, of what it is that we are doing as general practitioners within the system. It might have been okay 15, 20 years ago, when PINCER was around, but in today's world,I want something that will help me, advise me about this complex patient, and what's going on. And that, essentially is the difficulty. The other way of looking at it is, frailty. If you've got someone with high frailty, be definition, they'll be on 20 tablets, and five different co-morbidities, and living alone, all the rest of it. Well then, if someone was trying to improve frailty, they're probably not going to have (the CDS) as being one of the key things to improve. Whereas (the CDS) might be helpful for a 25 year old, who's just starting, and has got one particular condition, and they just need that one tablet [...] So, again, you're sort of starting risk stratifying, you're thinking, where does (the CDS) help? And it's with one patient, one condition. When you've got a highly frail person where, you know, you're actually asking the question, why are they even on all of these, do they need 20 tablets? It's a different tool isn't it?

GP6

Yes and that's the problem, you see. So if that popped up it might actually, I mean, obviously I'm fantastic clinician and I would never describe an anti-inflammatory with that but, you know, it might not have occurred to me that person was over 65 and things, so that would be useful in a one off. I suppose the problem is that you're actually 76 and you've got six different co-morbidities and that's when it just becomes overwhelming because obviously there are lots of issues that are potentially already going on with their other medications, so it all pops up at once.

GP14I: Yeah. I'll come back to that. You said something there that was interesting. Is that a general practice wide view, do you think or is that, do you have a specific take os prescribing (lead) ?

R: No, I think it's a…as a practice, I think we are all fairly similarly minded doctors here, particularly the partners, but the salary and the registrars as well. We all try our best for good quality, safe and cost effective prescribing. We are quite big prescribers in the local area, we have a lot of patients with a lot of medical problems, complex ones.

I: Yes.

R: So, we are always fairly above budget when it comes to prescribing budgets and stuff. Which is what's sometimes frustrating because I think we are quite on it compared to some other practices. I think it's just very challenging for a set of patients to [voices overlap 05:02].

GP14I: Yeah, let's just talk about that a little bit because I know that's quite interesting. Before, or, they don't want to come on to something else which is, sort of, rapidly writing things down, jumping around here a bit. But you talked there about complex patients, what is your, how would you describe the demographic of your patient?

R: Very low social economic, lots of people not in work, lots of people with lots of chronic diseases. For instance, things like, COPD, diabetes, smoking related cancers, heart disease, strokes, that, so, we have a lot a patients on multiple medications because they've got multiple medial problems. Because the area around here has always been traditionally and historically very socially deprived. We also have a lot of substance misusers, we do have a special substance misuse clinic every week. We look after the homeless services in (name of city) as well, so, we often have patients with that. We have a lot of mental health problems as well, so, a lot of depression, anxiety, chronic mental health disease. Again, patients are often on lots of medications. Next door to the local community mental health team as well.

I: Yes, right.

R: So, we have, really it's great for working as a doctor. Lots of variety and lots of disease, which is what we want to be treating and seeing patients. But prescribing wise that gives a lot of challenges.

GP14

R: So, prescribing wise, if patients are on long lists of medication already, you know, sometimes our (the CDS) will flash up saying, there are over 40 current medications to check against, we can't do it, which is never a good sign. So, we have lots of patients who are on already long lists of medications. Perhaps they've already tried various other medications as well and other challenges if they're under secondary care or other clinics elsewhere. Often we get letters that ask us to prescribe things that are perhaps beyond our expertise and might be a little bit more expensive. For example, just because they've got complex medical needs, we have lots of kids with intractable epilepsies and PKU and lots of, lots of odd prescribing just because that's our patient demographic, we just seem to have lots of complex patients.

<u>Sub-theme 4a ii: Patients - impact upon, engagement with and adaptation for, impacts upon care for individuals and the population</u>

<u>CCG Staff</u>

CCG Pharmacist 2

R: Yeah, it's happening, yeah. I don't know, I've heard the odd thing from colleagues, I don't know whether you guys are going to be talking to patients at all, but you do get the odd rumblings of patients, sometimes get a bit fed up with, oh I had this last time, and I've got this, and I've got this, and now I've got this. You know, it's like different switches all the time, and the products change, and they wanted to keep to that brand, because it worked better, and it tasted better, or whatever it is, sort of thing. So there is that, being mindful of the patient at the end of the day, and making sure that it's not all become so cost orientated. And it's being mindful of, I mean we were having a discussion yesterday at our regional meeting, about, sometimes, safety and quality interventions aren't going to save you money. If you think about adding a PPI, you're adding something on.

CCG Pharmacist 3

So we needed something there and it's supposed to be patient-centred care, so it's about looking at what is best for the patient. So it was putting the patient in the middle of it and thinking putting everything aside, okay, I know there's money, there's this, but what is safest for the patient? So I looked at it from a patient-centred approach. And that was how I managed to take it forward to a degree

You're not taking something away, but at the end of the day, it's all about looking at the bigger picture of safety for that patient.

CCG Pharmacist 5

I mean there are some drugs that we consider to be unsafe that we make non-prescribable so that message would pop up and say, you can't prescribe this because of x, y and z. That would save a patient from getting a drug that either didn't work or was so expensive that we would be wasting resources on something that could be spent elsewhere to benefit other patients.

GP Staff

GP Nurse 2

Yes, I think I'd like to think I do involve them. Yes, very much so, because with diabetic drugs there's so many at the moment. It used to be two tablets and then go onto insulin and now it's just, the market is huge. It's a long-term condition so you can imagine the amount of drugs that they want…So yes, I do discuss with them and obviously, you know, for some people, they just won't want to put on weight, fair enough. So yes, I'm quite happy to prescribe what's right for the patient.

GP Nurse 2

One of the things you said last time is that with some of those switches patients are not necessarily happy to accept them or not happy with some of those changes. Are you still finding that sort of thing?

R: Yes, particularly like with the contraception. I think some patients get very fixated on the ones they're on. It's difficult sometimes to get them onto an alternative one because they're saying, oh, no, I've had that one before, it doesn't suit me. I suppose the only thing now is I'm finding that we're having quite a shortage of certain medications anyway. So that's where the choice is actually taken away from them because some of the more probably expensive ones or bizarre ones that they've been having just don't exist anymore or it's difficult to get hold of so they don't really have that choice.

I: Right, I mean you generally would involve the patient in that, would you, in that decision making?

R: Oh, absolutely, yes. Some of the guidelines as well that are on it probably need updating anyway. There's a particular injection that I prescribe for diabetes which is a GLP one and every time I try and prescribe it, (the CDS) will say, there's a cheaper, you know, a more cost-effective version [voices overlap 06:25] but it doesn't work as well and, actually, guidelines are probably going to be updated soon anyway so I just overrule it.

GP Nurse 2

Do you engage with the patient in terms of saying, oh, no, they're telling you here to self-care?

R: Yes, I just say, you know, it won't let me and also, I always say it's just a lot cheaper. I mean obviously if they're not getting free medications it's a no brainer for them [voices overlapping 16:00].

I: No, precisely.

R: Most patients I find, I may just be lucky, have taken it quite well. You know, the antihistamine ones and buying certain drugs, I say, oh, it's much cheaper to get it over the counter but most of them accept it, I think, quite well.

GP Pharmacist 3

I: Yes. I'm really, that's really interesting, that's a really, sort of, that balancing act as it were, is happening in your...

R: It is, yeah. But if I've got a polypharmacy patient who is on 15 drugs a day and I'm trying to add in another drug at two or three times a day dosing, I will pay heed to the software, but if it's recommending three or four times a day dosing to someone who is already on…then I'll take the decision to swap it over.

I: Yeah. So, do you think the software takes into account the medication burden of that patient?

R: No. It just gives that recommendation. You are right; you are absolutely very good in pointing this out, the, considering the medication burden is a clinician and patient as well, role. Because they are the ones that are going to tell you straight away if they are fed up or not. But we always must bear that in mind.

GP2

For example, if somebody who has been a long time on the same medication […] and you don't want to change either the format or the tablets because the patient is elderly, and they got used to the shape of the tablets and the colour, the difference would be few pennies. However, it would be much more damage if the patient mixed up medications just because they are confused.

GP4

That's a decision I make with a patient and I'm on this occasion not going to take notice of that because I think the indication outweighs the risk that you've highlighted, so […] So technically what (the CDS) is telling me is correct, but I'm also judging that against what I think the patient might be willing to take…

GP9

I: Yes, I'm just looking through these questions here. I think, yes, I suppose actually that's something which I've talked to other people about. When you look at some of these systems like (the CDS) and think about what they're doing in terms of either cost-savings or safety messages or whatever, what do you think is the value to patients of things like that?

R: Well, I suppose they're not aware of the value because they don't care about cost. They just want the drug that they want so, yes, you'll get some people that want Lactulose as opposed to Laxido. It doesn't matter to them about cost. They don't understand what that means to the greater NHS or the greater services so I don't think it has any bearing on patients.

GP9

I: Does it have any negative bearing upon patients, the switches?

R: Only if they want a particular brand like Lactulose because they think it doesn't suit them but actually you tell them the other stuff is just the same, only in that respect.

I: When you're talking about those switches, I mean do you ever get patients not just saying I don't want it but, you know…?

R: If it's a global switch then we don't get involved, we just say it's the CCG choice so, you know, you've got no choice in that.

GP13

R1: Not consultations, because I think the way I use it, I don't often use it in consultations, because I suppose that's one of the problems of it, I don't know if you want me to speak about the problems, but the time it crops up in workflows most, is when you're signing today's 60 repeat prescriptions, so the patient's not in front of you, but there's a medication review overdue, everything's been done, the patient's had their blood pressure, had everything done, so you don't want to get the patient back, so you can update the review date, and then (the CDS)flashes up and tells you that something minor could need changing, and you sort of let it go.

GP13
R1: We had an elderly lady in a residential home, who was on 40mgs citalopram for her anxiety, and I think a medical student, or our pharmacist did an audit, or something came from the MHRA, I can't remember, anyhow it flagged up, we ended up doing a search. And it transpired that I had a knowledge gap, and I wasn't the only one, that 20mgs was the maximum dose you should be on, if you're over a certain age of citalopram. Now, as far as I know, (the CDS)had never told me that, and (GP CLINICAL SYSTEM) had definitely never told me that, because I went in and tried it. And she'd been having that for some time, without any, and I'd been signing her repeat prescription. So, I guess my behaviour had been, there is no warning, it must be okay.

GP13
R1: Yeah, I mean I do, very rarely would I try and hide anything from patients, 'cause I think when you're honest with them, I think trust goes up, rather than down, as an automatic thing, and I think there's also, I think people need to understand the NHS can't pay for everything, and so it's quite good to have, this isn't just me being an arsy doctor, this is the NHS saying, this is the most cost effective solution, or whatever, and sometimes maybe using terms like Big Brother, or Big Brother's watching us, this sort of thing, I think it helps, I've always had this idea that if a patient comes in looking for a trench war, then the best strategy is to jump into the trench and start shooting in the same direction as them, rather than build your own trench and start shooting back. So it's that sort of, it's us against the NHS sometimes, we'll get your care right despite this, don't worry, but we've got to do this […] Which might be a bit unfair, but I think educating, it's certainly helped me educate the population, that there are external cost pressures, that you can't just have everything you want, and that's not me saying that, that's someone else, look.

GP13
And it's like all software tools, it depends on the programmer doesn't it? So, I think it's very likely to improve care when it's triggered. So, assuming the rules that are in it, it's such a complex thing isn't it, care, that any model of care, including the one that drives how it's programmed, is going to be imperfect, and that's why I think you still need the PINCER stuff to check that what you think is a good idea, actually turns out to be one. But I think, there's direct ways it improves care, by, if you make the assumption that NICE guidelines are correct, which they probably are the best, I have quite a lot of faith in NICE, it's as robust a process as we can get to get the best population evidence to the individuals we're caring for. So, if you accept that, and then if you accept that it's programmed with NICE guidance to highlight deviations from it, then more patients are likely to end up on evidence based protocols of care, and fewer patients off them, and more patients, when they are off the evidence based protocols, that that's done with thought, rather than just by default […] And it's also likely that it's improving care by reducing financial waste, in cost effectiveness terms. So, by prescribing the cheaper inhaler, we've got…

I: More money to spend elsewhere?

R1: Less deficit, rather than more money, but less deficits. […] So, it's likely to improve care in both of those ways, but I don't think I would ever be really, being a cynical old doctor now, after however many years. I think I would always need the PINCER stuff to try and make sure that it's not decreasing care in ways that are less obvious, and so I'm not planning to stop doing audits.

<u>Sub-Theme 4a iii Prescriber characteristics</u>

<u>Software developer staff</u>
Software developer staff 3
R: I guess the other piece is thinking about who the prescribers are, or the different types of prescribers, or people within the practice who need different types of information. Right now, (the CDS)focuses very much on the GP prescriber, who has a certain requirement for certain types of messages, certain types of information. But actually, if you take something like drug monitoring for instance, when someone prescribes something, at point of care, telling them that they need to do a three month TFT or LFT, or whatever check, it's not necessarily the best place to tell them, 'cause in their head it's three months away, do you want me to do this now? Well if you do want them to do it now, you need to make the workflow much more easy for them to do that. So, can they get one click, where they populate their order entry screen, and it's done? And right now, the technology or the ability to do that, isn't there. But another way of skinning the cat is, can you present that information to a different person? So, if you got more pharmacists working in practice, or if you got pharmacists text from a support perspective, can you start surfacing those requirements around drug monitoring, or TDM, to a different person who's more likely to action it? So, I think that's where we're trying to push the needle a little, with (the CDS), and probably with the audit tool that we're now building, it's really trying to understand some of those use cases, within the way a practice works, and how the information, there's a wealth of information that needs to be served up, but actually how do we start focusing it to the right person, and [inaudible 00:41:54] in the workflow that matters to them?

<u>CCG Staff</u>
CCG Pharmacist 2
R: Yeah, so some surgeries, and perhaps probably most surgeries, will say to the reception staff, if that pops up, you're not to accept it, because a lot of it, it needs a clinician to accept it, so they might just put, like, a full stop on the rejection thing or... They don't actually indicate that they're not clinicians, and I might ask them to start typing that in, if they're not a clinician, because then I can see how many people are not clinicians and see in in that information, or even just a code to put in, so I can pick it up.

I: It's an interesting thing, because I've heard this before. That you might get someone, when the repeats are being re-authorised or something like that, and it might just be a question of administrative staff, obviously because they've got to go into the record, in doing that. And, as you say, it might be useful to have that, so you know who's…

R: Yeah, a lot of them will take screenshots and print it off and give it to the GP for them to look at later, but then that rejection box has gone and that data's come back.

I: Yes, quite. So you don't get that reason for rejection?

R: Yeah, so we don't always, and I'm sure there will be surgeries that the non-clinical staff will just ignore it and not put a reason, because there's a lot of blanks when they...

CCG Pharmacist 3
There were a couple of practices that we had problems with but they have not switched it off, and theirs was about who generates the prescription. It should only be a prescriber who generates the prescription. Some practices have a system in place whereby they have an admin person generating prescriptions.

I: Would that be on repeats or just…?

R: Not repeats because it only fires for acute and new. So admin people should only be doing repeats, generating prescriptions for repeats. They shouldn't be generating prescriptions for an acute drug, and they shouldn't be generating a prescription for a new drug. Okay? Apparently in some practices they do and that's the way they work. Whatever safety they have in place I don't know but as I said, we cannot condone that behaviour, so we cannot sanction it to say, oh, we know that you're doing it and because of that we're going to try and alter (the CDS) in some way or the other. It's not supposed to be done. It's your system, it's your loss because we activated it only for prescribers. We didn't activate it for admin people.

CCG Pharmacist 3
It's the different systems they use and I know it goes on, so it's not unique. And they might have prescribing clerks…

I: Right, I'm with you, yes.

R: …generating prescriptions, but they are not prescribers.

I: Yeah, precisely.

R: So they should not generate a prescription but what's happening is in some cases, they generate the prescription and they give it to the doctor to sign. And they should only be doing that for repeats. They shouldn't be doing it for new scripts. So let's say for instance somebody's had something previously and it was on as acute, just once. Somebody phones up and says, oh, they want that same item again. Now, the whole point of putting it for acute is that at that time, they could not make a decision as to whether it needed to be continued. So therefore they need to see the prescriber again to see if it needs to be continued. If it needs to be continued, then they can put it onto repeat. It's not up to the prescribing clerk to make that decision and there's always the likelihood that they can generate the prescription and the doctor just signs it without checking. But then the prescriber is 100 per cent liable. So it is a problem, and it's not unique to us. I know it goes on. But if we've put a safety mechanism in place to help them, and they decide to bypass it…

I: And it doesn't generate…(the CDS) doesn't fire when you go to do a repeat prescription…

R: No. it doesn't. Which is fine. Which is what we would want because if it fired… Because what happens is that you put on a new prescription, you have all the relevant messages pop up. So that's fine. You put on a new repeat, you have the messages pop up. But let's say for instance you've put on something for six months and for instance you've got a medication that needs to have a blood test every six months…

I: That would then [inaudible 31:12]…

R: …okay? It's not going to fire every month because you don't need it. You don't need the blood test for six months.

I: Yes, quite.

R: And as a rule, it is non-medical staff that would generate an issue, a reissue or a fulfilment of a repeat. If it came up then, it would be very annoying because first and foremost the non-medical staff would not be able to make a decision……as to whether they should accept it or not accept it. But the risk with accepting it means that they could accept something which could be unsafe. The risk with not accepting it is that the prescriber now doesn't see a relevant message. But if you've given somebody something for six months, unless somebody comes back and says they've had a severe reaction or it's contraindicated, you've taken that decision at that time that it's safe for this person to have it for six months. So you do not need to be presented with the same message month after month after month.

I: Yes, precisely.

R: And that would not have got engagement because that would have slowed down their systems immensely. And they

CCG Pharmacist 5
I: Yes, I mean, I can see that. But, you said that you feel that the safety ones, the cost saving ones are not getting a look. Because one of the things that I was thinking was that, from what we've been finding is that, you know, where this, you know, you're getting constant bombardment of cost saving, and often GPs will say, oh, it's just to save a penny. And...
R: That's when we try and limit the number of cost saving messages that it gets, so we try not to turn on the really low value ones. And yeah, I think possibly, the most valuable ones we perhaps wouldn't just do as a simple message, we'd put an explanation with it, so that you could

put in the titles something like, massive cost saving all over it], I don't know. Yeah, but most of them are fairly average sorts of savings and they'll just accept it. So...

I: And the problem is that, you know, even if it is a 20p savings, you have to think, it's not just you doing it, is it? It's across a CCG, across the country.

R: Exactly.

I: Once you've done all of that though, if it's a very, very commonly prescribed drug, it's going to add up, isn't it?

R: Yeah. I think one of the problems with the cost saving messages is often that cost saving is done per dose, so it doesn't look that huge. Whereas, if they gave the cost saving per month or per year, I think that would have a bigger impact .

I: Oh yeah. So, if it's 10p per day, but they're taking it four times a day...

R: It would be like, oh that 10p...

I: ...and they're taking it four times a day and they're taking it all day.... for the next year, it starts getting to be a lot more.

R: Yeah, it's that...so I think that might be useful if they could, yeah, change the way they've...they might have done it already, but..

CCG Pharmacist 5

R: Generally, most GPs don't complain that they get too many messages. And I think many realise that if they're getting a lot of messages perhaps they need to question whether or not they're coding their patients properly. You know, if they're actually, perhaps not up to date with what they know. But...

I: Because, if the coding is not good...

R: Yeah, if the coding isn't good they'll get more messages than they should do because all of the messages are tailored, so they're very specific messages, most of them. So, there's a logic behind them, they'll only pop up for patients with certain conditions, and if you haven't coded that condition then, or have coded the condition incorrectly, you're going to get...

I: Or if you've coded it more broadly.

R: Yeah. Or, if you're diagnosing something and you prescribe before you add the read code to the record, you're not going to get the tailored messages. So, you might get extra ones saying, well you can't prescribe that because this patient hasn't got that condition. But it's just they did it in the wrong order, so teaching [voices overlap 00:25:58].

I: Yeah, so you should rather diagnose before, diagnose the symptoms then...

R: Yeah, code it first and then prescribe.

<u>GP Staff</u>

GP Nurse 1

because I'm a nurse and I think we just get a bit more scared of making a decision.

GP Nurse 1

Anything else that I'm not doing on a regular basis I will speak with the GP, so I won't initiate the...I might initiate blood pressure management or I might initiate diuretics in heart failure but anything a bit more than that I would speak with the GP. Even when it comes to anaemia I might do the assessment and request the tests to get to a diagnosis but then I'll say to the GP, this is the result, they probably need iron replacements but because they need to go on repeat can you do it?

GP Nurse 1

R: No, so just don't use them, full stop. But I think it's the way that...I mean I only know how I'm trained, I did my nurse prescribing 11 years ago but I think the way nurses think because we see it as an extended role, it's not part of this. I think there's that element of it and we're always worried, we're always covering our back all the time. A doctor said to me once that it's the mind set of how we're trained, nurses are trained to question, have I done something wrong, and the doctors don't think like that. So it's how we think, scared to do something wrong and if we're scared we go and speak to somebody. So that might have something to do with it so we always...I'm sure the GPs do too, we're always studying and keeping up to date with stuff, doing non-medical. I get non-medical prescribing updates at least every other month at the acute hospital just to keep up to date with what's current practice, what we should be doing, antibiotics and stuff, what we should be avoiding and things like that.

GP Nurse 2

R: So I mean initially, although I wasn't prescribing, sometimes I would generate prescriptions but certainly once I'd started prescribing then you would see the alerts and the different options. That's when I realised it, really, and I find it quite helpful, yes. I, as a new prescriber, definitely find it helpful because it gives you the options and more and the cost and different… Then I don't want to get confused with like…because our CCG also put flash-ups on about certain drugs and obviously there's the formulary as well. So we've got that many things we're looking at and I know some can actually be turned off, can't they, on the prescriber?

GP Nurse 2

I: When I spoke to you on a very hot day, I think, in was it last summer, and you talked because you were a fairly new prescriber, about being a bit sometimes precautious when you were prescribing, has that changed over the last year or so?

R: I think I've become less cautious but certainly still aware… Particularly if it's something new that I'm prescribing, I'll be more cautious. So ones that I do on a day-to-day basis, I'm absolutely fine with. If it's something new I'd be more cautious always, yes.

I: (the CDS) as a system, then, do you think it's beneficial for people like yourself who have come newly to prescribing perhaps more than it is to people…?

R: Definitely, yes. Yes, definitely, and for people probably that have been prescribing a long time as well because, for example, I've got a colleague of mine who was at a practice where, although she did the long-term conditions, she didn't prescribe. The doctor there was prescribing all sorts of wonderful things that actually weren't on formulary and was away and above and expensive but you can switch off all the alerts so I suppose it's only as good as how it's set up, isn't it?

I: Well, yes, precisely.

R: If they're unclicking all the buttons and just doing what they want then, in theory, they can, can't they?

GP Pharmacist 1 and GP10

I: Is that because holding all that stuff in your head is…it's e…
R2: Impossible.
R1: It's impossible…
R2: Impossible.
R1: …and becoming increasingly more impossible in my opinion. Maybe that's just 'cause I'm getting old.
R2: It's impossible.
R1: But yeah…
R2: It's truly impossible.
R1: …because it's endless and also changing constantly and I think there's so many more variables now, so we now have much more issues now than we did this time last year with stocks shortages and having to prescribe alternatives because of things were out of stock and then maybe prescribing things that we wouldn't normally prescribe, for example when the Ranitidine was out of stock, we're starting to prescribe Cimetidine which we've not prescribed for years. So yeah, and just the amount of information, advice, guidance out there is just mind-boggling.

GP Pharmacist 1 and GP10
I: You think it's the right thing to do. One of the things that also has been said to me, perhaps I can use – see what you think to that – is that some people have said, oh, very useful for people who are less experienced. This is probably from someone who was very experienced, saying, but for me, and I've been prescribing 30-odd years, it's not actually useful for me, no, it's useful for someone who's just come out of medical school. But do you think…
R1: I would disagree with that, quite strongly.
R2: I would, 100 per cent. I would definitely really strongly disagree with that.
R1: I imagine [voices overlap 16:23] but might be a degree of…
R2: I mean, I think…certainly getting on a bit in years and fairly experienced in prescribing and I still value – even if I'm overriding occasionally – I still value that input. Yeah, very much so. Yeah.
I: It would be just [inaudible 16:46], wouldn't it?
R2: Unless I knew that I was perfect [inaudible 16:50]…
R1: Well, that sort of attitude is the unconscious incompetence, isn't it?
R2: I would have thought so [voices overlap 16:56]…
R1: I think I'm great, and I know everything, and I actually don't realise that I don't.
I: Yeah, I'm not going to say…
R2: It's always…it's nice to have the prompts, it's nice even if it's not appropriate in that circumstance…

GP Pharmacist 1 and GP10
R1: Although having said that it is useful for new prescribers or people who need it.
R2: Oh, yes.
R1: So I would say for…yeah…and pharmacists new to the role and that sort of thing and it's a really useful prompt because again, if you're new to this then…so I'm thinking of the pharmacists that are new to the role in general practice, there is so much to take onboard and it's a new role and it's a new setting and all of that stuff. So, any of this sort of thing, PINCER, (the CDS) is really useful for them because it just might be stuff they just don't know. And then that prompts them then to go and look it up or ask or speak to somebody about it, so they then know why that's coming up.

GP Pharmacist 3
I: There's too many alerts.
R: Correct, yeah.
I: Is that something you've found?
R: Yeah. Especially when I'm trying to do a polypharmacy medication review, I've got ten minutes to go through 15 items.
I: Yeah.
R: Yeah. I can probably do some of the major switches but then I really do need to sit in front of the patient for 20 minutes, which is what is recommended for a level three medication review in practice, to go through each one and make sure they are happy to go for it.
I: Yeah.
R: So, yeah, sometimes we just can't action them all. Or I think of what our clinicians would do is just ignore it all.
I: Yeah.
R: Because if the patient is stable on what they've had for the past 12 months, why change it and add extra complication to your consultations, when they are coming two weeks later to say, I've ended up with this new tablet, what's it for.

GP Pharmacist 3
I: So, in that respect the next question here is, has the potential benefits of the system be realised, have the potential benefits been…no?
R: I can't say fully, no. I'd have to be honest; I'd have to say no. 'Cause it's also down to the user at the end of the day.
I: Right, yeah.
R: The software is trying it's hardest, but if the clinician, the user isn't following it through, then no, it isn't [voices overlap 31:00].
I: So, again it's back to, there's the information, it's what your people are doing with it.
R: Yeah. Maybe it's down to the actual healthcare professional looking at the software. There may be a shift change; you may see more success when there is a pharmacist using it.
I: Yes.
R: So, you may see better outcomes when practice pharmacists are using (the CDS) rather than GPs.

GP Pharmacist 3
I: Does (the CDS) also need pharmacist input?
R: Orientation…yes it does. I think you would achieve better long-term outcomes if the software is more tailored for a pharmacy edge.
I: Yeah. And if then…

R: Where the pharmacist can look at the information provided and think, I need to audit that, I need to sort that, I need to (the CDS) this. So, yes, it does require work from software engineers but also the clinician sat on the seat. And maybe you just need a different clinician looking at your software.
I: Yeah.
R: Yes, my day is time pressured as I have ten minute consultations, just like the GPs, and also 20 minute consultations for long term, just like the nurses, but I also have more time in the day to do analytical work like this as well. And software is more optimised to help me do that, yeah; it would make my life a lot more easier.

GP2

R: It depends from which... Experienced GP, they know what they're prescribing and how they want to prescribe and the reaction of the medications and you're dealing with a certain number of tablets which you are familiar with. So, your prescribing habits changes very little. You know, it does change but it's not as much. You know, your first line, second line, third line...you have your boxes in your head. However, with the younger generation, with inexperienced GP...you know, younger doctors, I think it's quite useful.

I: Yes. So, it seems as though there is an experienced part to what you usually say, you're…

R: Yes, but for example if somebody's on amlodipine you will automatically change the simvastatin to 20 milligrams or change the dose. It shouldn't normally cross...across mind of my junior colleagues because it's not anymore...it was pertinent maybe five, six, seven years ago but it's not anymore in our eyes. For us it's like second nature. Oh, interesting, that might be the problem...change. And similar things to, you know...

GP 11

R: Yes. This is probably anecdotal, I'd say, but I think it has a bigger effect on training doctors, I would argue. And then, within GP partnership... This is very anecdotal, but I think there's probably a variety as to how you respond more to the cost effectiveness ones. Obviously, safety is safety when it comes to it.

I: So, you're saying there basically that the less experience, the more you perhaps rely upon it? Is that sort of fair, or the more you use it?

R: Yes, and I think that the stronger it would be that a prompt would be an absolute to not doing it. And, okay, I'll not do this until I've discussed this with a senior because I think for the rest of us, we would have different thresholds as to what to think of it depending on what exactly it was.

GP13

R1: Yeah, generally…is it just general practice, I think GPs tend to feel that we're better than anybody else, 'cause we manage without immediate test results and things, but general practice is about managing risk, it's all about managing risk. It's often, I'm no gambler, but I'm interested in the mathematics of poker and probability although, I don't play and I don't win when I do, but it's a very similar process, it's, you know, looking at ranges of possible outcomes, and what are the likelihood of them, and how can I shift those probabilities to be in favour? It's not about, do this, and this will happen. And some of it is, there's an adage, I don't know how true it is, that a good protocol will manage 80 percent of the patients that go through it, and the other 20 percent need a doctor. So, the pragmatism comes from the doctors […] And, I think, we had a meeting yesterday about skill mix and what have you, and that's probably one of the issues in general practice, is that we all try and do everything, whereas in fact, what we're trained to do as doctors, is to do the difficult stuff, being pragmatic, the balancing two, three, four, competing risks, and working out what to do despite them, and how to manage them, and how to safety net them and all that sort of stuff, and (the CDS)definitely helps from that, because anything that helps remind you of what the risks are, helps you manage them better. And so, what might have been, 15 years ago, a prescribing error, you could do exactly the same thing, but at least you're doing it as a consciously managed risk now, rather than as a prescribing error.

GP14

I: Yes indeed. And, so, if you were…would you use the system, because both of those examples answer around acute prescribing. Would you use it for any review of patients, so, if you were going say on re-authorisations of medicines?
R: Yeah, so, no I don't. I think when we're doing medication reviews and you can see the list of whatever's on that patients repeat, you go through each one generally with the patient, ask them what they're taking it for, check that it's still indicated.
Because you can see the medications on the list, any potential interactions, you see already. And I don't think I would know how to do it because you're not automatically going to get a pop up when you re-authorise the medications, so, you don't get any alert if…
I: If it's just a repeat?
R: …If it's just a repeat, yeah. So, I don't use it particularly for a repeat or medication reviews.

GP14

I: Right. So, you are, by in large, using it around that acute prescribing?
R: Yeah, although if you're putting in a new repeat prescription on, so, say you seeing somebody with heart failure or something, you want to start them on an ACE inhibitor or whatever, and you prescribe it and you're putting it on repeat, it will still pop up if they have a contra-indication to that particular drug that you're trying to put on repeat, if that makes sense.
I: Yes.
R: Whereas if you're just re-authorising medications that you are…that the patient is already on and perhaps has been on for quite some time, they…
I: …They know they can't.
R: It's not just acutes it's the initiation of a new drug, is probably a better term.

GP14

I: No. But in general, then, so, you will use your…what's driving that clinical judge…what's driving that decision making, if you like?
R: The drive is doing what you think is best for the patient and what's going to make them better whatever the situation is. If they're poorly, or we are treating their chronic disease well or whatever. So, obviously, that relies on your own experience, skills, confidence, that sort of

thing. I think probably as maybe one of the relatively older GPs, having lots of, having a reasonable amount of experience and confidence in what you're doing, probably means that you cancel it off more often than a younger one. Don't know. It probably varies.
I: Yeah. That's interesting.
R: Again, probably part of your own personality and your own experience.

GP14I: Some people don't realise that. You'd be surprised. Has it, you know, at the beginning you were talking about you came in one morning and there it was. How has it fitted in to your work, has it…?
R: In what sense, fitted in?
I: As in…has it disrupted or changed or made any difference to the way you work?
R: Disrupted, no not in a negative way particularly, as I was saying before, having had quite a lot of experience of other things popping up and even within System One there's often little pop ups that come up. It's part of daily working in an IT system, it's fairly normal for me. Computerised records have been around a long time now.
I: Yeah.
R: So, just part of the normal daily working routine for me.
I: Yeah. So, it's not…there's no massive thing, it is used as you say isn't it?
R: Not for me. I can imagine [voices overlap 25:05-25:06] other people, personalities might find it irritating. But it depends on how you work and what your own thoughts and attitudes are towards it.

<u>Sub-theme</u>
<u>4a iv. Prescriber interaction with patient</u>

<u>Software developer staff</u>
Software developer staff 2
R: I suppose the other thought, I guess we're seeing GP practices merge quite a lot, and these super practices, and I guess the days of a GP knowing their list size and knowing their patients is going. They won't have that knowledge of the patient sitting in front of them. So in a way, (the CDS)and any other [inaudible 0:44:39] perhaps becomes a bit more important, to prod them to think of things that they might not be aware of about individual patients.

<u>CCG Staff</u>
CCG Pharmacist 5
R: I think sometimes it's, I haven't got the patient in front of me. I physically can't do anything about that so I need the patient there to talk to them. They might not have time to deal with it then and there. Some of them, they may well have accepted it in their head but don't want to do it now so they'll reject it or close it but do it anyway. It also fires for test patients so some of this could be people training or looking things up. It might be our team doing switches and because we're recording it elsewhere…

I: [Inaudible 31:05]. So, part of that is…yes.

R: …we'll reject the message because it will come up for us as well.
<u>GP Staff</u>
GP Nurse 3
I: I know that sometimes these things come in at the wrong time. […] During the consultation. And that can affect things. Have you found that at all?

R: You see, what I would do is I would do the whole consultation first, and the prescribing would be the last thing I did.

I: Yes, so because the prescribing is coming at the end, and the decisions to prescribe particularly…

R: Was done at the end, yes, so everything else that has gone before…

I: Yes. So, if it was coming earlier…

R: Yes. That would make the impact then if you did have to change it.

I: Yes, because you had to change your mind halfway through. Yes, so if it was coming earlier, would that be…

R: That would make the impact then, if you did have to change it.

I: Yes, yeah, because you had to change your mind, halfway through.

R: Yes. I mean I'm not saying that hasn't happened because it has, and I just explained why I've changed my mind, and I tell them the truth […] I can't prescribe you this because there was an issue with your kidney function last time, so I'm changing it to this one.

I: Yes. Yes. And patients are fine about that? […] I find that they're happy, so long as you tell them the truth […] They don't mind […] It's when you start dancing round it and trying to…a bit, shall we say, not so truthful, that there will be a problem, so I just tell them the whole truth and that's…

GP Pharmacist 1
R: Yes, so again, slightly differently, if I'm doing a medication review and it pops up with a formulary thing then I would discuss it with the patient and I would say, oh, the computer is telling me that you're on x but actually y is the same medication but more cost-effective. Really, we should be trying to prescribe that and then have that discussion with the patient. I suppose that's a formulary option thing.

R2: I think I have that same discussion if they specifically are asking for a drug and that's what pops up, I do. Then that's a different matter but, yes.

I: Not on a safety one, it's about…yes.

R2: No.

I: Not a safety one, yes.

R2: No, on the safety aspect, yes, I'll follow the guidance if it's appropriate and I won't say anything until I've seen what that is and then relay that information.

GP Pharmacist 3

R: …. in my perspective as a pharmacist I do see, my consultation is structured differently to a nurse and a GP.

I: Yes.

R: I have sat in with GPs and I've sat in with nurses and I've also had registrars and STs sitting with me and watch me work, and they've said to me, you structure your consultations differently, you converse with the patients in a different way. My weighting on having a successful consultation with good outcomes weighs on the patient being happy with the clinical reason they've been given.

I: Yeah.

R: Hence, the weighting is on the patient.

I: Is that something to do with the types of patients you are seeing? Because by large you are seeing people with long term conditions I presume.

R: Yeah. Correct. However, some patients like pain management, have to be told differently. So, then the weighting will tend to rest more towards me and the software.

I: Yeah.

R: If I'm trying to prescribe, let's say, Pregabalin, but the software says Gabapentin is clinically equivalent and safer and more cost effective, that will weigh more than what the patient is after but they will still get the same outcome, because they never knew whether they were going to get Pregabalin or Gabapentin.

I: Yeah.

R: But then that's a decision that me and the software have made together.

GP3

R: Another thing is, and I'm very careful about this, if there's been historical bad prescribing, to change that you've got to say to someone that wasn't right, and that's actually quite a difficult thing because you undermine either your own confidence or the confidence they have in the medical system. So it's a really thin line there sometimes to say what the bloody hell's that? I think my plan seems to be working not too bad. Again, it's one of those things that I'm not quite sure where we'll be in three months' time. It's like the Forth rail bridge, I'm painting it and going back to the start again.

GP3

R: I don't think the patients notice, no.

I: The patients know anything.

R: Because I normally know when it's going to come up and I normally know I'm moving onto the next bit before. So I've worked out my plan before. Certainly here.

I: What I was thinking was has there ever been a time when you've said to a patient oh, well, I think what we'll do is we'll give you X for the next five months and then see how that works, or three months and see how that works, and you've told the patient…

R: Yes, is the answer. What you say is yes.

I: …and then it pops up and you go oh, hang about, I can't give you that.

R: Even me. Yes. Because that's the whole point. If I was perfect I wouldn't need anything. So no, these reminders that are both educational and just summing up perhaps we're all human, we make prescribing errors. It just reminds you. It keeps you straight. And occasionally I've said to patients I'm going to do this, and I'll go well, maybe that's not such a good idea. So yeah, some of them, particularly the cardiac arrhythmia ones, because there's so many newer stuff that's on there that affects QT and all these other things that even five years ago wouldn't have been on [voices overlap 0:31:58].

GP3

I: Right, okay, yeah. How about patients themselves? Presumably within the consult…does it lengthen the consult or make it more difficult or longer?

R: It potentially would. Again, that's what I'm saying, it depends how I react to… In some ways I'll let it deliberately lengthen the consultation if I'm at the other place and I just need its help to go through things to be safe. So it will take a bit longer. I don't mind that if it's thinking time and I'm incorporating it. Sometimes the bit that it doesn't do that I have to add to it is does this patient need this medication in the first place. And it doesn't do that for you yet. So I have to do that bit. It might suggest reducing dose or change…but it rarely says is this indicated, which is the bit I've got to do when I do a medication review […] So I need to fit in with that. And if I get enough red star warnings I've got to think is this worth the risk and how do I impart that to the patient that this has got some risk, what level of risk it is.

GP4

R: Yeah, but that's not to say that I'm right. It's an odd…it's just there's so many things you're juggling at the same time about… So technically what (the CDS) is telling me is correct, but I'm also judging that against what I think the patient might be willing to take…

I: Yeah, precisely.

R: …how long I might be going to use that thing for so I might for example choose to override a concern because I've decided that I'm only going to use it for two weeks or something, whereas the problem that it's highlighted might apply to a longer term use.

GP4

R: That's a decision I make with a patient and I'm on this occasion not going to take notice of that because I think the indication outweighs the risk that you've highlighted, so… And I think if that was happening a lot, I think if that was constantly jarring and it was telling me to do things that I was finding irritating, then I would say so but I don't really get the impression it is hugely and when it does I close it down.

I: So when you feel that your expertise takes over, as it were, oh no, I'm doing this, yeah? 'Cause that's really…that's…I find that really an interesting area, that whole …it's not a decision system, it's a decision support system.

R: Yeah. I think that's right.

I: The expertise of prescribers has still got to be there.

R: Yeah, but that's not to say that I'm right. It's an odd…it's just there's so many things you're juggling at the same time about… So technically what (the CDS)is telling me is correct, but I'm also judging that against what I think the patient might be willing to take…

I: Yeah, precisely.

R: …how long I might be going to use that thing for so I might for example choose to override a concern because I've decided that I'm only going to use it for two weeks or something, whereas the problem that it's highlighted might apply to a longer term use.

GP4

I: Any other positives about it in terms of the way you use it then, or any negatives?

R: I think the negatives are always just another thing that…distracting you from the patient, I suppose it depends…the whole transaction really between patients and doctors has changed throughout the last 23 years and it's just another thing that's distracting your attention. So you get…it's a newish thing for us, so you're ending up trying to prescribe something, you're trying to prescribe something smoothly and efficiently and quickly and in a way that doesn't detract from what you're talking to the patient about and then the thing pops up and you're, oh, right, sorry, oh, hang on, and you're usually…

I: Do you find that…how does that impinge on that relationship, I mean, is the patient aware that you're making those changes, or…?

R: No, not always, no, 'cause I wouldn't always vocalise that, so they might just find themselves looking at my ear for a few moments. And I'd guess they'd probably surmise that I'm…that my concentration has moved really from them directly but I guess that happens a lot if we're looking at results or we're looking at a letter from the hospital, so I suppose patients aren't particularly surprised by that really.

GP4

R: Well, I suppose…and also, it's like a confidence thing, isn't it, in that if you say, right, I'm going to prescribe you this and then you start umming and ahing about it…but actually, I do see that as a positive really. Once you get beneath that slight clunkiness that it causes, it's actually…we shouldn't be prescribing things whilst we're half chatting to patients and being distracted. We should be focused on that task. I mean, general practice you're often mentally juggling quite a lot of balls so it's quite easy to drop one. You dropping a prescribing ball is potentially very serious. So yeah, I think it's just that I…I find it relevant and I don't resent it. I think of why. I've got on with it really.

GP4

R: I think it's…I'm trying to get to the nub of why, what would make it feel intrusive versus any other sort of reminder or pop up, and I think for me it's two things, it's quite often it's useful and I can see the point of it, and also it's easy to overrule it if I don't want to…and, for example, the QOF pop up that might be saying to you, you know with patient, not that it's irrelevant to what you are doing because obviously it comes up when you are trying to prescribe something. So, you have got the patient in front of you, you need to prescribe them something, you have decided that's what you want to do, and this system makes me feel that it's helping me to do that in a cost effective way, and in a way that's safe. Whereas a QOF pop up, for example, can be just completely left field, it can be it's just noticed that this patient hasn't been asked about smoking or they need a BP doing, but actually they've come because they are distressed because they've lost their job or something. So, they can be very intrusive and not relevant. It's sort of a non sequitur really, whereas the (the CDS) comes up when you need it really, it's not something that just bounces up in your face when you're doing another unrelated task really

GP4

R: I think where it's easier is with acute prescribing, you know, if you are starting something, I suppose if you come across something that, you know, somebody has always done because they've been on repeat, you might be [noisy background 16:11] think about a change, it's more difficult. With an antibiotic, I suppose, where something comes up, I suppose that Nitrofurantoin is a good example, the patient doesn't necessarily know what you are intending to prescribe them at the point the (the CDS)thing comes up. Sometimes I say, well you know we need to sort you out with something for that type of comment, and then you end up starting to prescribe something (the CDS)pops up, it gives you an alternative and then at that point you'll say, right this is what I'm going to give you and this is how I'd like you to take it, type of thing. So, what I tend to find is that very rarely have I gone into a detailed description about what I am going to prescribe somebody and then I've suddenly realised

that that's not appropriate because of (the CDS) and I've had to sort of backtrack and say, no actually I've changed my mind I'm going to give you something else. So, in that sense, maybe that's just to do with the way in which I consult, but I suppose if you'd decided to give somebody a long explanation about what you to prescribe and then (the CDS) told you something different then that's going to be more intrusive, but that hasn't tended to happen because, I suppose a lot of us will start to explain what we're going to prescribe as we're prescribing it really.

GP7 and GP8
R2: You can say, I'm not allowed to do this.

R3: You can say, it's not me. You know this isn't, you know a few years ago we were saying we shouldn't be prescribing this because you can get it over the counter or…

I: That's interesting, that's really interesting.

R2: It does make it easier to pass the blame on to that, yeah.

I: To be able to pass the responsibility on to whoever…

R2: Somebody who isn't there.

I: So it's not like the "computer says no", but it is…

R2: Well it sort of is. You can say that…

R3: This is the local guidance and this is what they suggest. It's what we're doing locally…

R2: We're not allowed to do that.

R3: …/nationally, whether it's NHS England or CCG, and patients accept that.

GP7 and GP8
R2: Because they think we're just being mean.

I: Yeah.

R3: Patients have often said to me, you're not giving me that because it's coming out of your budget. The implication being, because it's coming out of your budget, it's coming out of your pocket. They don't understand the complexity. I mean we have a notional budget and we're trying to save the CCG money, but…

R2: It's not our take home money.

R3: …they seem to think that it's your money. And now that we have this box I just show them this box. It's wonderful. I'd love to give you this stuff, if it were down to me, I'd give it to you. You can have bucket loads of the stuff, but I'm not allowed to. Oh fantastic, no arguments; it takes the argument out of the consultation.

GP7 and GP8
R2: But that's a lot easier when it's an initial script because it's a lot more difficult to say that when they've been having it for the past ten years and then you say, we no longer want to give this to you. Then it is, because they do know somehow that it can be over-ridden, because they've just been having it haven't they?

R3: They have but we're starting the process or re-educating, whether it's gluten products, whether it's anti-histamines, whether it's, whatever, the message over the last year or two is getting out.

And people are now going to the, they do all the minor illness stuff as well. So people are now going to the ch(GP clinical system)t for their thrush treatment or whatever. So slowly but surely I think we're going to be able to educate our patients to save the NHS a little bit of money or prescribe cost-effectively.

And one or two patients, well one patient said to me I know I was given Atorvastatin this brand by the consultant and I've now been given this one, which has upset me. I appreciate you need to save some money but is there any way round it.

GP 11
I: Yes. So, you wouldn't do it and if you'd shared that information with the patient? It's also interesting as well because talking to other people, people have sort of talked about that if an alert came up on the screen, they wouldn't actually tell the patient that, that was actually happening. Or they wouldn't actually talk about why they were changing... Well, this computer has told me change the medicine. So, you would be prepared to talk through some of that with patients?

R: Yes, I think so. Well, it's thinking of the terminology with it. And again, there seems to be some variety in the categories of this. I think particularly now that I've been here a couple of years and there's been a few practice-wide changes that have been as part of (names of places) wide changes. I think knowing where they've come from and, you know, are they based on safety and or cost effectiveness, I think getting into a bit of patter of we're looking to change Seretide to Fostair and, you know, this is through a mix of effectiveness and de, de, de, what do you think? And then, see where people fall with that.

GP12

I: So in terms of your, you know, it pops up and your able to prescribe, so just talk me through how you would use it or how you would interact with the system.

R: Okay so, you'll prescribe something or other and then (the CDS) will say, how about something else? That's the usual way, how about this rather than that and if you prescribe this rather than that you'll make £2.50 savings a month. Would you like to do this? And I'll put yes or no. Now, if it is an acute first item that I am giving to the patient, then actually I'll often go along with it because the patient doesn't know anything else. The difficulty comes when you're doing repeat medications and it pops up then and you just think, the faff of changing it midway through when a patient's already used to one thing, in order to do that you've got to contact the patient, you've got to explain to them, you've got to, you know what I mean. And that's the sort of thing that our pharmacist would probably do, but actually, we wouldn't do as GPs because we just haven't got the time for it.

I: That's interesting, because you're saying therefore that it's, if the patient doesn't know anything for the acute new drug, fine. But swapping something for a patient, more difficult?

R: Yeah, for us as GPs, we just haven't got the time, there's just too much stuff.

GP12

Now, if it is an acute first item that I am giving to the patient, then actually I'll often go along with it because the patient doesn't know anything else. The difficulty comes when you're doing repeat medications and it pops up then and you just think, the faff of changing it midway through when a patient's already used to one thing, in order to do that you've got to contact the patient, you've got to explain to them, you've got to, you know what I mean. And that's the sort of thing that our pharmacist would probably do, but actually, we wouldn't do as GPs because we just haven't got the time for it.

I: That's interesting, because you're saying therefore that it's, if the patient doesn't know anything for the acute new drug, fine. But swapping something for a patient, more difficult?

R: Yeah, for us as GPs, we just haven't got the time, there's just too much stuff.

I: Because you'd either have to call the patient back in…

R: Yeah, if you were going to do it properly, you've got to at least let the patient know. It's a little bit easier now with texts because we can actually send texts to patients, so actually, which is a wonderful one-way method of communication and the patient has got to call the practice if they are going to object. So that makes that a little bit easier but still, I think it's something that I'd be more likely not to bother to do.

GP12

I: Yeah, that's interesting.

R: Whereas actually in (GP CLINICAL SYSTEM) it was much better presented. Somehow it just looks simpler. It was the tight spacing, the whole issue that it was a smaller box. (the CDS) is written in a bigger type face. You knew what it was. It just seemed to be, I don't know whether there were less buttons but it just seemed that in the (GP clinical system) version of Optimise, it just seems so much, you know, so much more potential for different buttons that you're expected to kind of get right. But actually, you just don't have that time when you've got a patient with you and the patient is talking to you. They're telling you about their next problem while you're trying to prescribe their last drug that they want. And it's just, it's all I can do to remember, override or, you know, just two buttons to get through quickly before, you know, while it's going on.

GP12

R: So you've then got the patient in front of you talking to you at the same time as you're trying to issue a prescription.

I: And this is the fitting it around that whole consult there.

R: That's right. And because (GP clinical system) has so many more clicks. I spend my time actually…you know, quite a few times I have to say to patients, excuse me, I'm terribly sorry, I can't multitask anymore. And even then I'm trying to get through that prescription as fast as I can in order that I can spend the quality time with the patient and therefore I just, you know, I mean the (the CDS) alert is just another, you know, awful (GP clinical system) screen thing to plug my way through to just remember the two boxes that I need to click to get on to the next bit.

GP13

I: Yes, which is what we talked about before, yeah, precisely. And an over reliance upon the alerting. What about patients? I mean, you talked about the fact that most of the time, you are looking at it, it's for re-authorisations and repeats and what not, and you're less looking at it, when you've a patient in front of you. Are there occasions when there's patients in front of you, or what's the impact on patients of the system?

R1: I mean, it doesn't never flash up when patients are in front of you. And when it does, there's definitely been, the last two or three years, patients are becoming much more accepting of some external NHS, which is telling us now what to prescribe. So, three years ago, it was, if I made a prescribing decision on clinical grounds, I wanted to change someone's adalat to some other form of, 'cause nifedipine wasn't used for blood pressure anymore, so I wanted to change it to one of the NICE guidance, the patients would either, I like my Adalat, or you're doing this for cost reasons. They would be definitely, I'm doing this, they would be blaming me for is. Whereas now, there's definitely this sort of, the NHS Big Brother, is not paying for earwax removal anymore, and patients are going, oh alright then. Fewer of them are kicking up a fuss about that. And (the CDS), just a small number of occasions, has definitely been a visible sign to patients, when you can look at it and go, oh look, Big Brother's telling me we can't use that one, we're supposed to use that one. And people are getting used to that now, which I think is a good thing, 'cause it's…

GP13

R1: Yeah, I mean I do, very rarely would I try and hide anything from patients, 'cause I think when you're honest with them, I think trust goes up, rather than down, as an automatic thing, and I think there's also, I think people need to understand the NHS can't pay for everything, and so it's quite good to have, this isn't just me being an arsy doctor, this is the NHS saying, this is the most cost effective solution, or whatever, and sometimes maybe using terms like Big Brother, or Big Brother's watching us, this sort of thing, I think it helps, I've always had this idea that if a patient comes in looking for a trench war, then the best strategy is to jump into the trench and start shooting in the same direction as them, rather than build your own trench and start shooting back. So it's that sort of, it's us against the NHS sometimes, we'll get your care right despite this, don't worry, but we've got to do this [...] Which might be a bit unfair, but I think educating, it's certainly helped me educate the population, that there are external cost pressures, that you can't just have everything you want, and that's not me saying that, that's someone else, look.

GP14
R: Yeah almost the same. So, basically it would flag up, for example if you're trying to prescribe some Naproxen or something and the patient is over 65 and you haven't thought, actually I should giver her PPI, it flashes up and you go, oh yeah, give her PPI. Then it might affect the consultation because you'll then say to the patient, I forgot to tell you or whatever, we need to prescribe something for stomach infection because in these medications can cause stomach irritation, so, I need to give you something to protect that as well, so, you're going to get two items on your prescription and then prescribe whatever PPI you might choose.

Sub-theme 4 a v: Prescribing contexts in general practice

Software developer staff
Software developer staff 1
So I've seen probably more development from a user end rather than from an end user - using the software end. We are limited on that respect because we rely on the clinical system providers to support us developing that side, they have lots of other priorities. So I think no one will really know this this but there's something called SNOMED, which is a new way of READ coding, and that on everyone's priority. So if we say, oh, can we change this in the box, that is not a huge priority for System One, SNOMED and everything is.

Software developer staff 1
There's also housekeeping aspect, because we do have messages that will trigger on products that don't have a READ code associated with that medicine. So a classic one might be something like Aricept. And Aricept is used for people with Alzheimer's and dementia. GPs get paid to have a dementia register, so they have lots of different payments. We actually have a message, if someone's on Aricept but we cannot find a read code for dementia or Alzheimer's that meets the criteria for that product, we'll say, we cannot find a read code. So we're actually helping the GP make money because we're highlighting the fact that they've got someone on a product, the patient obviously has got dementia and Alzheimer's, and they've not READ coded it. So I like to...that's a nice way to promote it to GPs is that it will help them with their housekeeping and their read coding and their lists.

Software developer staff 1
I: What do you think are the biggest strengths there are of the system?

R: The fact that it can link in with the patients' READ codes. It's its strength and it's also its weakness, based on the fact that it works really well for a practice that read codes really, really well.

I: I've just made a note, read coding.

R: Yeah, if you've got a practice that isn't brilliant at read coding, then they won't make the best out of software. This is...

I: Because the system pulls from the individual patient record?

R: Yeah. So for instance, we might have...one of our most hitting messages is around use of junior aspirin, aspirin 75 milligram. And it's obviously used in wide abundance with people for what we call secondary prevention, so they've had something that's happened, a stroke or a heart attack or, you know, they've got Raynaud's or poor circulation. But we have a message for primary prevention, so they've not had anything go wrong with them. Now if you think back to the Daily Mail in the 90s, baby aspirin was a cure-all for everything, you know, cancer and all of that. So there was a huge upload in people wanting this super cheap aspirin that was going to prevent all of these things, and slowly but surely they ended up on repeat prescription, despite the fact that it's super cheap. And now data has shown that a lot of these people are maybe having haemorrhaging or GI bleeds and problems, and because they actually don't have any cardiovascular risk, they're just putting them at risk of something else. So guidance says now, NICE says if you haven't had anything, don't take aspirin. So we have a message that will scoot across all of the records and will see have they had any of the multiple conditions that would indicate aspirin. And if they haven't, we say stop. It's a stop message, don't carry on with aspirin. And it's one of the most readily ignored or rejected. And the only reason it could be is that they have got a reason to be on it, but they've not READ coded it. They may have free typed hypertension, or they've used such a random read code that it's not worked. Going over to SNOMED will hopefully help with all of that, but that...so even though it's the strength of our tool, it's also the weakness. And the biggest example of that is, we have an amazing suite of messages around the best appropriate antibiotic. So we've got the Public Health England antibiotic guidance. So, say, you come in with cellulitis, then if the GP puts cellulitis in and he puts appropriate antibiotic, we can say which one they should have. That's all based on the fact that if they type in "cellulitis" before you've put your antibiotic in. And I will say 9.9 times out of ten, you'll go in, they'll give you an antibiotic, see how you are, come back in seven days if no better. And then will end the consultation, by which point it's all too late, the prescription's gone and we haven't had the ability to trigger our software, because that relies on the ability for that GP to type in the word "cellulitis". Because it happens within the consultation but if they do that after they've put our drug in, our drugs can't pick up on that READ code. And I try and teach GPs that, so just again, I get, I haven't got five minute. It's like, I have no time [inaudible 0:18:03]. You know, and there's a patient perception that they've come in and if they see a GP typing, they're not listening, then patients get annoyed. And you can understand that, it's like, hang on, I'm here, stop looking in your computer.

Software developer staff 2

I: So you think, because it's at the point of prescribing it's more going to be a tool around that acute GP activity rather than…I mean, reauthorisation is the patient's not sitting in front of you so…

R: Might be, might not be, yeah.

I: Yeah. It's either way.

R: Yeah. So my theory is that most of our change comes about at initiation of prescribing. Some might happen at reauthorisation, but a smaller proportion. However, we have a suite of monitoring messages, and they're really key for reauthorisation, because if you're about to renew something for another six or 12 months then monitoring should have occurred. That's the best place for it to be available.

Software developer staff 2

R: We've also responded nationally to the product shortages that are affecting a lot of patients, pharmacists, GPs, when drugs are not available. We try to produce content to support where we know it's national and it's a very difficult picture because it's not always national.

I: Yes, quite.

R: But when there's been something that's come from the Department of Health or something… And we've had to try and really get to these people at Department of Health. Often some of this information that you think is public is sitting behind paywalls or you need privileges to access. So we've gone right to the top, (name) has, and spoken to the Department of Health and shown them what we do, and they're like oh, this is quite innovative isn't it, and quite useful, yeah, of course you can have the information. So we've tried to get that flowing a bit better so that we can respond quite quickly. It's only part of the story for GPs, but hopefully if they see a little alert, they're about to prescribe something that might not be available it can at least prompt a conversation with the patient to say, I'm prescribing you this, you might not actually be able to get it at the first pharmacy, would you try a couple more, and if you can't get it at all speak to the receptionist and I'll have made a note of what else to give you. Rather than they get frustrated when patients come back, they can't get stuff and things, at least if they're forewarned at the time of prescribing they can maybe adjust their plan.

Software developer staff 2

I: Actually, I just made a note and I realised I made a note when we were last talking around this business of acute prescribing versus this reauthorisation. I remember a GP somewhere, I said talk me through when you get that alert sort of thing, and I was talking about a patient walking in and wanting something, perhaps they'd done their knee in or done their back in and they wanted some naproxen but they were over 65, et cetera. And the (the CDS) alert comes up and says make sure you prescribe PPI. The response immediately, was oh actually, no, (the CDS) is much more reauthorisations. And other people have said, oh no, I only use (the CDS) for acute. There is this variation of end user use. Not CCG use, at GP level. I don't know how much you get to see that or know that or is that something that you are aware of? What do you think to that?

R: Yeah, I think there is a wide variation in our practice prescriber and their policies around that and therefore how they will interact with Optimise. I guess we are largely blind to that in an (the CDS) world because account managers [inaudible 0:19:10], we deal with the MM team. And unless they are people who work within the practices as well – some do, some work centrally and do some work out in the field as well, others don't. So we really rely on those third hand, second hand insights from those people and from all the account managers, pharmacists, so we've all got some level of experience of things in the past. I think for me, and I'm quite lucky in that I've been involved with a few practices for Analyse is that I'm getting much closer to the end user for Analyse and for Optimise. So through this process we will probably get closer for the practices a bit more. And already we've got a handful of practices that we're working with and we've got a handful of different processes that they use. They're all a bit different. And even the standard functionality within the clinical system, they all use different things.

Software developer staff 2

R: I suppose the other thought, I guess we're seeing GP practices merge quite a lot, and these super practices, and I guess the days of a GP knowing their list size and knowing their patients is going. They won't have that knowledge of the patient sitting in front of them. So in a way, (the CDS)and any other [inaudible 0:44:39] perhaps becomes a bit more important, to prod them to think of things that they might not be aware of about individual patients.

Software developer staff 3

R: Because now you're able to kind of capture patients where it may be appropriate to consider whether they should be started on a new treatment, which we can't do in Optimise, you can't do, 'cause the trigger is a medication being prescribed, you can't advice that there's a patient who has been diet controlled for their diabetes, but actually their HbA1C has gone up, so we should consider starting them on some sort of oral hypoglycaemic agent. But, with an audit tool you can start doing that. You can start identifying opportunities for new intervention. You can also start thinking about opportunities for escalation of therapy. So, those are definitely areas where we're looking at, and you can start doing mass bulk changes, or you can start understanding who needs what test.

And that kind of management of patients, we think is going to be take up more and more by non-medical staff.

I: By other people than GPs, yeah, indeed.

R: Exactly, and that kind of leaves the GPs, to deal with the more complex patients.

I: Yeah, acute diagnosis.

The reason why, that example you gave with the diabetes, just checking back there, the reason why that wouldn't then necessarily be useful with Optimise, is because the diagnosis isn't already in there, or the change isn't already in there, so you have nothing to support that decision making, is that right, have I got that right?

R: No, I think, well, it's more likely that you do have the information in there, but if you think about what it means to, with the ority of those patients, not much is going to change. And I guess you're right, it's when things do change, are those elements put into the system? […] But even then, unless you're making a prescribing change, or it's a new drug being prescribed, that information isn't going to be taken account of […]

I'm just trying to think of an example. So, patients on an oral hypoglycaemic agent, and actually their HbA1C has been in control, but their renal function has been going off […] At that stage, you may say, right, it's time to add in an ace inhibitor, or any ARB too as part of their long-term management plan. So, we need to stop x, y and z. So, they may have had their renal function tested, and they may have had their albumin-creatinine ratio measured, and those values may have been put into the system, but unless, (the CDS) isn't going to tell you to consider an ARB, unless you're putting it in, you're actually prescribing something for it to go, ah, I'm running this rule, I'm looking at a renal function, and I think, right, we need to do this.

Software developer staff 3
R: I guess the other piece is thinking about who the prescribers are, or the different types of prescribers, or people within the practice who need different types of information. Right now, (the CDS) focuses very much on the GP prescriber, who has a certain requirement for certain types of messages, certain types of information. But actually, if you take something like drug monitoring for instance, when someone prescribes something, at point of care, telling them that they need to do a three month TFT or LFT, or whatever check, it's not necessarily the best place to tell them, 'cause in their head it's three months away, do you want me to do this now? Well if you do want them to do it now, you need to make the workflow much more easy for them to do that. So, can they get one click, where they populate their order entry screen, and it's done? And right now, the technology or the ability to do that, isn't there. But another way of skinning the cat is, can you present that information to a different person? So, if you got more pharmacists working in practice, or if you got pharmacists text from a support perspective, can you start surfacing those requirements around drug monitoring, or TDM, to a different person who's more likely to action it? So, I think that's where we're trying to push the needle a little, with (the CDS), and probably with the audit tool that we're now building, it's really trying to understand some of those use cases, within the way a practice works, and how the information, there's a wealth of information that needs to be served up, but actually how do we start focusing it to the right person, and [inaudible 00:41:54] in the workflow that matters to them?

<u>**CCG Staff**</u>
CCG Pharmacy Technician 1 and CCG Pharmacist 1
R: The main thing was to explain that it would go on everybody's computer, so that included receptionists. So the main thing was that they wasn't to accept or reject these messages because it's clinical information. So that was the main thing to get across.

I: And is that part of the way the software operates or is it…?

R2: Yes. So Script Switch would go on the individual computer, clinicians' computers. (the CDS) goes on everybody's in the practice.

CCG Pharmacist 2
R: Yeah. We're a mixed bag with that, because it's accessible to everybody in the surgery who logs in, so not everyone's a clinician.

I: Yes, as it's within the record.

R: Yeah, so some surgeries, and perhaps probably most surgeries, will say to the reception staff, if that pops up, you're not to accept it, because a lot of it, it needs a clinician to accept it, so they might just put, like, a full stop on the rejection thing or... They don't actually indicate that they're not clinicians, and I might ask them to start typing that in, if they're not a clinician, because then I can see how many people are not clinicians and see in in that information, or even just a code to put in, so I can pick it up.

CCG Pharmacist 2
Exactly, and then they'll only pop up if specific things trigger them from the practice end. So, if, like, someone's read coded with a certain condition, for example, or if someone's already tried... Say, it was a cost-saving, if someone's already tried that, what we're asking, it wouldn't flag again, because they've already had that conversation, they've already done it and we don't want to keep mithering the patient all the time. There might be a valid reason for them not. Say, it was a cream, maybe there's something in that cream that they particularly had a reaction to, for example. So, yeah, I suppose a lot of it, we depend on what is from the practice end, what's read coded, and in terms of conditions. And also the medication side, if it's a trigger against an actual drug, that's a bit easier, because if they've had it before, it won't trigger. So, I think it just depends on what's on their system.

CCG Pharmacist 2
R: Yeah, and I think that's one thing, that when they were sort of selling it to us, the prescribing thing, was, that it is quite an intuitive, intelligent system, that you won't be getting that alert come up for every single patient who's on that drug, it's when they trigger, with, for example, poor renal function, and this matches and this matches and this matches, then it will come up.

I: Yes, precisely. If you don't Read Code that correctly.

R: Yeah, exactly, so it does rely a lot on, and I don't know, I think that was one of the concerns when they first started, like actually people aren't in the habit of doing Read Codes, it depends on the practice, or the prescriber. Some people are probably better than others. But, there is that element of it, that they were a bit concerned that actually, are they going to Read Code it properly, and if they don't, then it's not going to trigger as you'd want it to. So, there is that issue as well.

GP Pharmacist 2
I: So, you're a CCG pharmacist who comes here, or are you based here?

R: So, when I first came here, I was on the pilot scheme, so that's the GPPTP, so this pilot scheme, where they wanted pharmacists to be in the GP practice. So, I was only based at (name of general practices), like physically, but then I would work remotely for the CCG. Does that make sense? […] So, go to the CCG meetings and stuff. So, my split was, yes, it's always been 60/40, yes. And then on that scheme, the money, sort of, came to an end. It was supposed to be a four year scheme, I was only on it for two and then the practice took me on as a practice employee. But, I still have a CCG element to my job, again still for the 60/40. But, again, just remotely, so that, I'm trying to think when it was, it wouldn't have been the summer gone, it would have been the summer before, they were getting us to do all these (the CDS) updates and messages. And turning

things on and turning things off, that sort of thing, where we were all sort of separate CCG bodies. And then, sort of, (CCG partnership) was coming through, so we did it for a bit, but now...and we all had...the way the divvied it up was chapter wise of the BNF, prior to (CCG partnership). Which seems a little bit like you're duplicating the wheel, because if (name of CCG) are following (name of place) APC formulary, and the message is that...then we're doing the same, yes, we're just doing the same work for the Optimise. Yes, so it just seemed like we were doing each other's work. So, it's better in a holistic approach, but now it's all being looked after by (CCG partnership). So, I don't have to necessarily run reports and look at what messages are being ignored.

CCG Pharmacist 3
It's the different systems they use and I know it goes on, so it's not unique. And they might have prescribing clerks…

I: Right, I'm with you, yes.

R: …generating prescriptions, but they are not prescribers.

I: Yeah, precisely.

R: So they should not generate a prescription but what's happening is in some cases, they generate the prescription and they give it to the doctor to sign. And they should only be doing that for repeats. They shouldn't be doing it for new scripts. So let's say for instance somebody's had something previously and it was on as acute, just once. Somebody phones up and says, oh, they want that same item again. Now, the whole point of putting it for acute is that at that time, they could not make a decision as to whether it needed to be continued. So therefore they need to see the prescriber again to see if it needs to be continued. If it needs to be continued, then they can put it onto repeat. It's not up to the prescribing clerk to make that decision and there's always the likelihood that they can generate the prescription and the doctor just signs it without checking. But then the prescriber is 100 per cent liable. So it is a problem, and it's not unique to us. I know it goes on. But if we've put a safety mechanism in place to help them, and they decide to bypass it…

I: And it doesn't generate…(the CDS) doesn't fire when you go to do a repeat prescription…

R: No. it doesn't. Which is fine. Which is what we would want because if it fired… Because what happens is that you put on a new prescription, you have all the relevant messages pop up. So that's fine. You put on a new repeat, you have the messages pop up. But let's say for instance you've put on something for six months and for instance you've got a medication that needs to have a blood test every six months…

I: That would then [inaudible 31:12]…

R: …okay? It's not going to fire every month because you don't need it. You don't need the blood test for six months.

I: Yes, quite.

R: And as a rule, it is non-medical staff that would generate an issue, a reissue or a fulfilment of a repeat. If it came up then, it would be very annoying because first and foremost the non-medical staff would not be able to make a decision……as to whether they should accept it or not accept it. But the risk with accepting it means that they could accept something which could be unsafe. The risk with not accepting it is that the prescriber now doesn't see a relevant message. But if you've given somebody something for six months, unless somebody comes back and says they've had a severe reaction or it's contraindicated, you've taken that decision at that time that it's safe for this person to have it for six months. So you do not need to be presented with the same message month after month after month.

I: Yes, precisely.

R: And that would not have got engagement because that would have slowed down their systems immensely. And they wouldn't have been able to do that. So it had to be acute and new repeats.

I: So is that something which you chose to [voices overlap 32:36] …?

R: No, that is how they set it up and we're completely happy with it, and that is also what happens with ScriptSwitch as well. The problem with ScriptSwitch was when you renewed all the repeats, the messages didn't come up again because you renewed them all together.

I: Right, so when you renew them they come up, but when they're reissued, they don't , yeah, on automatic. Oh, yeah.

R: Yeah. So we were very clear about that and that was what we wanted, acute, new repeats, the message, that's the only time the messages fire.

CCG Pharmacist 5
I: Within that then, there's some working relationship, then, isn't it, because they sort of like maintain this relationship with the practice, how important is that to keep those working relationships in the team here and so on, going?

R: I think for the practice pharmacists, they need that relationship to be able to work with the practice. Without the relationship, the GPs aren't going to make the changes that they want to change. They need to be able to trust that practice pharmacist but because they already trust a practice pharmacist, when this new system came in, they went with it which was great. I think because it was done as a mass project, all their peers were doing it as well, they didn't really question it as much as they could have done. I think they saw it as a way of saving money and I think they liked the fact there was that safety net for them that if they did do something horrific, they'd get a pop up message to say, are you sure?

I: Yes, absolutely. Have the teams here and that, has that changed things at all or have things changed because of Optimise?

R: I think the way that people approach projects has possibly changed a bit in that it's part of a project plan now to think, Optimise. Yes, it's a good way for the practice pharmacist to, sort of, find out if there's any problems with prescribing. They'll often use the data from (the CDS) for their own practice to spot problems that they can perhaps go in and do some education or, yes, do an audit of their own.

I: In terms of practice pharmacists are we talking about CCG pharmacists who go in?

R: I'm talking about CCG pharmacists.

I: Yes, rather than practice pharmacists who are solely employed or based at the practice or NHS England ones or whatever.

R: I don't think… In fact, I'm sure they don't have access to the reports that our practice pharmacists would do from (the CDS)so we're quite keen for our practice pharmacists or the CCG pharmacists to access those reports for their own practice. They present those at the practices' annual review so it's become embedded as part of the, this is how you are doing talk every year. I think the practices like the fact that they can be compared to other practices.

GP Staff

GP Pharmacist 3

R: Yeah. To say that the recommendation of the software is pulling through, I can't do it because it isn't in stock.
I: Yeah.
R: Which is a big thing at the moment, yeah. And they'll say, absolutely fine, ignore the message; carry on with your own prescribing.
I: Right. Yeah, that in itself is interesting.
R: What I don't know, is that if there's a situation like that, is how quick they can adjust (the CDS) for an alternative that is in stock. But then with the way prescribing has been at the moment, something has been out of stock one week and it's back in two weeks later.
I: Yeah.
R: So, I've been completely stumped at the moment with hypertensive patients, I've actually had to switch their drug altogether, either to a sister drug or a different class only to find out that the original drug is back in stock three weeks later when they've come back in for a blood pressure review.
I: Yeah.
R: That sort of timescale, I don't think they can roll out within (the CDS).

GP Pharmacist 3

R: It's, I think with a lot of GPs they will listen to PINCER work because they are aware of it, it's audited and they can see changes in their future practice because of evidence. Whereas it's difficult on the go with (the CDS).
I: Yeah.
R: And trying to think of a reason why the switch is good when you have already explained to the patient why you want to prescribe something and (the CDS) is giving you a different decision, it's tricky.

GP Pharmacist 3

R: Fairly, yeah. That's a fair assessment. They are coming in from a financial assessment, also with PINCER, safety always. In some cases, they don't consider the patient. Particularly not in this area, they are very very good. In other areas where I've seen blanket switches being made by medicine management, it's been a lot of kerfuffle going on.
I: Yeah.
R: And I've had to, as a practice pharmacist, then pick up on that and bring the patients in, have a chit chat, and tell them why the changes have been made, are you happy with it, would you like to be changed back.
I: Yeah.
R: We see it differently. In this area, we complement each other better because we talk, and there is no attrition, from my professional relationship with medicines management.
I: So, a lot of that is based around that relationship.
R: Yeah. I think that relationship is most important.
I: Yeah.
R: Especially when medicine management understand what clinical and practice pharmacists are in practices for.

GP3

The differences is at (name of practice) we don't see it very often. We don't see the cost saving ones. We don't see the interactions. We don't see great safety warnings. And that's perhaps because of the work we've done on prescribing here, and also on our population. Our population are happy to do over-the-counter, for lots of things, and we work very hard I think on the prescribing here. We see it perhaps…I would say a third of what I see at the opposite extreme, which is a city centre, highly deprived, highly unorganised practice in some ways where we see the safety alerts all the time. It's almost universally it's on 90 per cent of the time, which is quite striking. The branch from here is in between the two. It has more cost savings coming up on it. It doesn't have any more safety warnings than here, but it has more cost savings. I think that's just a reflection of the population as well.

GP3

R: Yeah. On the ground it's very different.

I: …of the patients makes such a difference to that.

R: But I think also the organisation of the practices, maybe that's reflected in the patients as well. The more affluent sometimes the more organised.

GP3

I: Yes. And taking the NSAID over 65, should add a PPI, is that an alert that…does it pop up or is it something which you could know about anyway, you would do?

R: It pops up, and this is where I have an argument. What's the difference between 64 and 66? This is the problem about PINCER. It's much better if someone suggests would it be a good idea to have some PPI cover with this? That's a much better way, rather than say these are the people that have found…or over 65 that are nonsteroidal.

I: Yeah.

R: It's not the same. You say it's the same, but it's not. One offers you a bit more of a solution, the other one you have to go back and say what you did wrong.

GP3

I: No, it's more difficult to. And in terms of actually using the system, if you can just describe how from your point of view how you use it, how it's part of your work as it were.

R: When it comes up it will give me a suggestion of what to do. It will give me some information that most of the time I've seen before, so I know the next step. So it's whether I agree with it or I do the "not suitable for this patient" or some other information down that side. In some ways I think my default in over 50 per cent is "not suitable for this patient" at the other place. Here my default would probably be to put the switch screen that says use original, and that's fine, I just click on the convert. There's one that I struggle with, and again it's my age, when you've got a default trade name. I struggle with this. Because in all my training in all my life it's been beaten out of me that I'm never to do this, and then suddenly tells me it's all right.

I: Because you normally default to generics.

R: Yeah, I have a generic default. It's a very strong default. So, for example, the amoxicillin to amoxo, it hurts. It doesn't feel right. And again it's an easy one and it makes no sense, it's no cost, but the worst thing I would ever get was a patient saying I don't want amoxicillin, I want amoxo. So I'm always just a little bit wary about those switches. I do do them. The creams and things tend to be the interesting ones because they tend to be quite seasonal, the changes. The one I can think of recently is the beclomethasone to betnovate, which again just feels a bit odd. But if I've got enough time I click on as many as I think is reasonable. There's often as I'm going through a surgery and when I first did the other surgery I had over 300 patients on over 25 medications, and that is impossible for (the CDS)to help me. I've got to be really quite selective about what I do. Because if I've got 20 alerts up when I'm doing a medication review I cannot deal with 20. But I've learned to be a bit more selective about it, and I'll go for the low hanging fruit, and I'll perhaps do a little bit in the notes to say discuss such and such next time. Hopefully we get maybe less and less with time. I think they probably have already. The first month I was there it was [inaudible 0:18:25].

I: So do you think there is a distinction or a contrast between the tool running as a useful aid to prescribing for polypharmacy, people with multi-morbidity, people with 25 medicines or whatever to people who are coming from just one…?

R: Yeah. The people that would be best dealing with sometimes the polypharmacy are the pharmacists that have got a bit more time rather than ten minutes, because it would be a good reminder to them. Because they're human like me. It's a good safety and cost reminder, but you just need a bit more time for it. The original polypharmacy I said to them if someone's got over 25 medications I need more than ten minutes because I just cannot do it, it's not humanly possible. But I haven't got more than ten minutes so I'm in a catch 22. So perhaps it's thinking about the pharmacist when she uses that on the multiple ones, she takes a bit more time and maybe has a plan for the patient. Because I think probably there's no point changing 12 things at once. It will not succeed. And I am very male about this, I take a single task and if I can get that single task done then I'll move on to the next one. And I think the difference between us as good prescribers here and pretty shocking prescribers elsewhere, that's why (the CDS) works in a very different way. But we are getting a little bit of battle fatigue with it, and the fact that I will go to "not suitable for this patient" a bit quicker than I would have three months ago even.

GP3

R: We just do I think actually. It's interesting, if we come across the geeks like me, Doctor [name 0:34:52] we discuss that amongst ourselves if we come across something that's a bit weird and wonderful, because that's how we are. But in general I can't remember having anyone saying oh I turned it off today or oh, can't say that. The only problem is when we have any IT problems. We've had real…that's slowed our clinical systems down last week to walking pace…

I: Yeah, I heard there was an outage last week.

R: …and that makes everyone go oh, click it off. And the problem is will they click it back on afterwards?

GP4

R: We haven't really talked about it on a practice level actually, which would be a quite interesting thing to do. I mean, I think it's been useful. So I definitely see a benefit from a person…

I: Anecdotally that you mentioned earlier. Yeah. And in terms of patient outcome do you think that's the…as well as perhaps the costs of it?

R: I think it must do, yeah, I think it must do, I mean, it's…actually what would be quite interesting would be to write…I mean, one thing before you came, I was thinking I need to try and – in the day or two before you came – just 'cause I was thinking about it, I thought I need to try and think of or try and notice examples of when it's come up because I think actually I've probably just accommodated to it, it's in…

GP4

R: And we get used to compromising in general practice, so you look at patients and I think sometimes we're very pragmatic, or most GPs are very pragmatic and that's how you get through the day. Whereas sometimes you'll get a pharmacy person coming in and doing medication review and we saw this with the pharmacy – I can't remember what you used to call them – the medication use reviews wasn't it I think. And we'd get a long list from the community pharmacist saying, did you realise this interacts with that and have you check they're using these 'cause this can cause a low sodium and you'd look at this list and think, well, that's just far too much, we couldn't possibly deal with things at that level of detail. It just wouldn't be practical. And so you make compromises about the sort of things that you've decided are worth worrying about and the sort of things that are possible, but at a lower level of concern really. And that tiering of risk is how we function really.

GP5
R1: But, we've had a couple of pharmacists in the practice, that we've employed, and we basically didn't replace them. We found them not to be very helpful at all. And, so, while everyone else has been saying they've been absolutely fantastic, employing pharmacists in a GP surgery, we haven't. But, similarly, when the pharmacists were here, they were complaining about the fact that we weren't using them very much, and they didn't really know what to do. So, it was a two way thing. And when we asked them, well that's really strange, why is that? We are an outstanding GP practice, we've had pretty well maximum QOF points for the last five years, we're fully doctored and fully nursed. That tells us everything you need to know. So, when they turn around and said, well when we come here, all your patients on ace inhibitors, they're already having their Us and Es checked, 'cause you've got a tight system. You're well-led, you're well managed. You can sort of see can't you? Happy patients, happy staff. You've walked into the surgery, Wednesday 12 o'clock, was it complete pandemonium? […] And yet you know that this is a very, very deprived neighbourhood. […] My patients are dying ten years younger than the national average. And yet, there's no queue, there's no one screaming and shouting or anything like that. […] Well-led, well managed, fully doctored, fully nursed. But that's not the same in other practices. And, in some ways, the kinds of things that it's advising us for, would maybe be useful for when you've got someone who's a non-GP who's suddenly got prescribing rights.

GP5
R1: I do, but it's fairly rare. And, it's a bit like the clinical system, that you know, how often do we actually follow through on the safe measures, and how often does it actually change practice? Or, do we just close it down and move on, because we've got 50 other repeat prescriptions to sort out?

I: Yes, right.

R1: And again, it comes back to well-led, well managed. If you've got a small number of prescriptions, if it's quiet, 'cause you've got lots of doctors and nurses around, you've got a lot more time to, oh I think I'll read that, and find out about it.

I: Yeah, right.

R1: But, if you're fire-fighting, and you've got people waiting out of the door, and all the rest of it, then you haven't got the time.

GP5
R1: NHS is in fire-fighting mode. And most of my GP colleagues are trying to get out of GP surgeries, and going off and doing something different. Social prescribing. There's a lot let risk there than there is in prescribing drugs.

I: For what reason, then?

R1: Yeah, absolutely. If I send you to go off to do some dancing and singing, you're not going to complain about that. But, if I prescribe a drug to you that causes you to have renal failure, well that's a complaint. So GPs figured that one out, so let's go off and do social prescribing. That's why you'll hear a lot about social prescribing, and, isn't that wonderful, and it's great, because as long as I refer you for social prescribing, there's no risk, or there's very little risk.

GP5
R1: Websites, information sites. The problem is GMMMG website, is constantly changing, but that's not being passed on. In a previous life I was the Map of Medicine clinical lead for the north west of England. Now that was the map of medicines, that wasn't just medication. 'Cause that's the issue with Optimise, it just doesn't go far enough. It's just purely about prescriptions. When actually, you want it around chronic disease management, and all the other bits and pieces, which is basically what the Map of Medicine would have been. So, although, yes, (the CDS)is good, it won't take too many brain cells to figure out, but why are we just limiting it to medication? Because I'm not a pharmacist, I'm a doctor.

I: Yes, right, okay. So, the limitations then, from your point of view, are that it's just going to change a prescription, it's not going to look at other things?

R1: Yes, because we're talking about a clinical system, aren't we, and a clinical system isn't just there for prescribing? If I was a pharmacist I'd understand that. But again, 'cause I was the Map of Medicine clinical lead, I've seen the bigger side, the bigger picture. And the bigger picture is, actually we're doctors that deal with the whole gamut of medicine, not just prescribing.

GP5
R1: The GMC is not going to be impressed with just looking at medications, they want to look at everything else that you've done as well. And that's why (the CDS) is always going to be just a very small bit, and that's why, it's not surprising the CCG would push that, because it's a prescribing budget issue, but from a GP point of view, if I'm looking at safety, prescribing is just one part of…

GP5
R1: NHS is in fire-fighting mode. And most of my GP colleagues are trying to get out of GP surgeries, and going off and doing something different. Social prescribing. There's a lot let risk there than there is in prescribing drugs.

I: For what reason, then?

R1: Yeah, absolutely. If I send you to go off to do some dancing and singing, you're not going to complain about that. But, if I prescribe a drug to you that causes you to have renal failure, well that's a complaint. So GPs figured that one out, so let's go off and do social prescribing. That's why you'll hear a lot about social prescribing, and, isn't that wonderful, and it's great, because as long as I refer you for social prescribing, there's no risk, or there's very little risk.

I: So, is there a sort of…?

R1: So, (the CDS) needs to direct people to social prescribing. That's how you reduce risk. It sounds crazy but…

I: No, not at all.

R1: …I can tell you now, that's exactly where things are going. You know? And it's a lot less stressful, and patients love coming back and saying, oh doc, I joined…you know, no one ever thanks me for prescribing a blood pressure tablet, but if I send them off for a singing thing, or a dancing thing, they'll come back a month later, saying, you've transformed my life. And that, again, indicates that these are basically social determinants. These are patients who don't need another tablet. What they've got is loneliness. And they need to meet people. Which is why social prescribing is becoming so big in communities like this, because it's not tablets that they need. So, in a way, Optimise, every time you type in an anti-depressant, it should say, have you considered social prescribing? Now, if I was doing safety, from an holistic perspective, that's what I'd be wanting to point to, not GMMMG.

I: Right, yes. So messages need to be…?

R1: In other words, why are you prescribing a tablet? You should be prescribing talking therapies or whatever it might be.

I: Yes. So…

R1: But, that would only come from people who are looking at it holistically, rather than just, I'm here to do a medication thing, and this is to support medical prescribing.

GP12

I: So in terms of your, you know, it pops up and your able to prescribe, so just talk me through how you would use it or how you would interact with the system.

R: Okay so, you'll prescribe something or other and then (the CDS)will say, how about something else? That's the usual way, how about this rather than that and if you prescribe this rather than that you'll make £2.50 savings a month. Would you like to do this? And I'll put yes or no. Now, if it is an acute first item that I am giving to the patient, then actually I'll often go along with it because the patient doesn't know anything else. The difficulty comes when you're doing repeat medications and it pops up then and you just think, the faff of changing it midway through when a patient's already used to one thing, in order to do that you've got to contact the patient, you've got to explain to them, you've got to, you know what I mean. And that's the sort of thing that our pharmacist would probably do, but actually, we wouldn't do as GPs because we just haven't got the time for it.

I: That's interesting, because you're saying therefore that it's, if the patient doesn't know anything for the acute new drug, fine. But swapping something for a patient, more difficult?

R: Yeah, for us as GPs, we just haven't got the time, there's just too much stuff.

GP12

I: So in terms of your, you know, it pops up and your able to prescribe, so just talk me through how you would use it or how you would interact with the system.

R: Okay so, you'll prescribe something or other and then (the CDS) will say, how about something else? That's the usual way, how about this rather than that and if you prescribe this rather than that you'll make £2.50 savings a month. Would you like to do this? And I'll put yes or no. Now, if it is an acute first item that I am giving to the patient, then actually I'll often go along with it because the patient doesn't know anything else. The difficulty comes when you're doing repeat medications and it pops up then and you just think, the faff of changing it midway through when a patient's already used to one thing, in order to do that you've got to contact the patient, you've got to explain to them, you've got to, you know what I mean. And that's the sort of thing that our pharmacist would probably do, but actually, we wouldn't do as GPs because we just haven't got the time for it.

I: That's interesting, because you're saying therefore that it's, if the patient doesn't know anything for the acute new drug, fine. But swapping something for a patient, more difficult?

R: Yeah, for us as GPs, we just haven't got the time, there's just too much stuff.

GP12

I: And it's just a question that that will just take up too much time or is there other reasons you wouldn't want a patient who has already started on something to be changed?

R: I think if I'm honest, it's just purely the time and energy involved and if the patient objects that can be 20 minutes of your time, when you haven't got 20 minutes for that actually, you've got more important things to do. And we do rely on our practice pharmacist, you know, we've got a pharmacist in the practice, so she will do the kind of switches that the CCG wants in the background, so that's going on and she will contact patients directly and she will do that sort of work. So we feel that sort of work is going on, we're doing our bit.

GP12
And we do rely on our practice pharmacist, you know, we've got a pharmacist in the practice, so she will do the kind of switches that the CCG wants in the background, so that's going on and she will contact patients directly and she will do that sort of work. So we feel that sort of work is going on, we're doing our bit.

I: So you can do that at medicine use reviews or when she's doing [inaudible 07:01] relations and stuff?

R: Yes that's right, or she can switch them round and she'll text them all and she'll receive the phone calls and she'll deal with it.

GP12
R: It is, it is, it is. This is during the consultation that we're tending to see (the CDS) because I don't see (the CDS) when I do, it's very rare that I seem to see (the CDS)when we're doing the prescriptions because it seems to work out that what happens is that prescriptions in (GP clinical system) come to you attached to a task. What happened when we started in (GP clinical system), and practices don't realise this because they've got used to, you know, they haven't got the (GP clinical system) off the shelf brand new. When you got (GP clinical system) brand new off the shelf, every prescription comes in a task and it comes in a task to a particular person. So we were getting about 30 tasks and we had to un-attach the prescription to a task and do things to it and sign it and it was just ridiculous paperwork [voices overlap 10:19]
I: So this is on reauthorisations and...?
R: Yeah, and it was just removing it from the task and doing what needed to be done. And then we couldn't work out what were other practices doing because, clearly, they weren't taking two hours to get through their prescriptions or whatever. So then we worked out that what other practices were doing is they use a prescription clerk to remove the task, the prescription from the task, and then put it neatly in a box where all you have to do is go down very quickly, go approve, approve, approve, approve. No (the CDS), no nothing comes up. You just approve this batch load of prescriptions that could be about, I don't know, 40 of everybody's. You hardly have, you don't see them, you hardly see the drugs. It's a bit like the old, here's a load of prescriptions in a basket, sign them all for the day. It feels a bit like that on a computer. So we've worked out that we now do all that. So the only time that you would therefore see an alert is either if you get a task to say meds overdue. And I don't see many (the CDS) on meds overdue partly because I think our repeat meds are probably not too bad in terms of our prescribing, what we prescribe and cost effectiveness and stuff like that are not too bad, so we don't see it then. So the most likely time you're going to see an (the CDS)alert now is with a patient in front of you when they request something funny or you're prescribing something acutely.

GP12
I: So do colleagues basically are feeling...
R: Do they feel the same, yeah, I think everybody feels it. I think that there also, I mean, you can in (GP clinical system) you can switch off the drug alerts. Now that we've found the place where user preferences can actually switch off loads of alerts, we're all sitting here, you know, is the decision which we are making in the practice is which alerts in (GP clinical system) are we going to switch off, which is safe to switch off and which aren't, we don't know, and it just, you know. I think one of the colleagues has switched off all of them because she actually thinks, you know. And it maybe that what we need to do is switch off all the (GP clinical system) ones and just use (the CDS) if (the CDS) is going to give us better alerts but we haven't worked that... Nobody has advised us, nobody said, nobody from the CCG has said this is what you need to do if you want safe prescribing. It just sounds ridiculous. It sounds ridiculous that at the last partners meeting I'm saying, (name) [19:55] can you work out which alerts we should and shouldn't be using. If you're the national person for safety, and yet I don't even know in our practice what we, you know, what's safe anymore. And nobody seems to, you know, nobody said to us I don't know what other practices are doing, it's crazy.

GP13
R1: Not consultations, because I think the way I use it, I don't often use it in consultations, because I suppose that's one of the problems of it, I don't know if you want me to speak about the problems, but the time it crops up in workflows most, is when you're signing today's 60 repeat prescriptions, so the patient's not in front of you, but there's a medication review overdue, everything's been done, the patient's had their blood pressure, had everything done, so you don't want to get the patient back, so you can update the review date, and then (the CDS) flashes up and tells you that something minor could need changing, and you sort of let it go.

GP13
I: Yes. What about acute prescriptions, do you ever see it firing now?
R1: Yes, it fires sometimes on acute prescriptions. I'm trying to think of an example recently, 'cause it doesn't happen that often, and it isn't that intrusive. As I think about it, I wonder if it's actually too non-intrusive, because it might be too easy to dismiss without acting on it. In terms of choosing formulary stuff, so drugs that are on formulary, I have a habit of checking the formulary regularly, because it changes so often, that often…

I: They meet once every two months or something, don't they, or something like that?
R1: Yeah. And so I have a habit of checking the formulary quite regularly anyway. Particularly if it's something I don't prescribe very often, so it's already on formulary, so I guess (the CDS)wouldn't flag up anything other than safety issues at that point, and then (GP CLINICAL SYSTEM) flags up many safety issues anyway.

GP13
R1: Yeah, generally…is it just general practice, I think GPs tend to feel that we're better than anybody else, 'cause we manage without immediate test results and things, but general practice is about managing risk, it's all about managing risk. It's often, I'm no gambler, but I'm interested in the mathematics of poker and probability although, I don't play and I don't win when I do, but it's a very similar process, it's, you know, looking at ranges of possible outcomes, and what are the likelihood of them, and how can I shift those probabilities to be in favour? It's not about, do this, and this will happen. And some of it is, there's an adage, I don't know how true it is, that a good protocol will manage 80 percent of

the patients that go through it, and the other 20 percent need a doctor. So, the pragmatism comes from the doctors […] And, I think, we had a meeting yesterday about skill mix and what have you, and that's probably one of the issues in general practice, is that we all try and do everything, whereas in fact, what we're trained to do as doctors, is to do the difficult stuff, being pragmatic, the balancing two, three, four, competing risks, and working out what to do despite them, and how to manage them, and how to safety net them and all that sort of stuff, and (the CDS) definitely helps from that, because anything that helps remind you of what the risks are, helps you manage them better. And so, what might have been, 15 years ago, a prescribing error, you could do exactly the same thing, but at least you're doing it as a consciously managed risk now, rather than as a prescribing error.

I: Yeah, rather than just simple, oh you shouldn't have done that, it's a, well there's a decision process towards that, and it's around…?

R1: I can give you an example. So, we recently had a woman in a nursing home in AF, who 15 years ago, would never have been anticoagulated, because she sits in a chair in a nursing home, and is hoisted and stuff like that. But, because of decision support, not just Optimise, but various methods of decision support, and [inaudible 00:12:36] she was put on rivaroxaban for her AF, and it was managed appropriately, and then she fell, and she's recently died of an intracerebral haemorrhage. But I think 15 years ago, we would have been thinking, oh blimey, what did we do there, was that the wrong thing to do? Or, if she'd had a stroke and not had it, we would have gone, oh there we go, that's the… Whereas now we can go, actually, that was still the right management, she wasn't falling, she had AF, so whilst she's had a bad outcome, but actually she's been on rivaroxaban five years, and she might have had a stroke three years ago, and been disabled. So yeah, so that's an example of how, 15 years ago, it could have just been not even noticed as poor prescribing.

GP13
R1: Yeah, although that sounds very grand, because most prescriptions I sign, I don't give that level of thought to, 'cause I haven't got the time to do it […] Which is where a lot of the stress comes from in medicine, not just on prescribing, but in anything we do, making a diagnosis, or not making a diagnosis, or ordering a test, or not ordering a test, we don't really have the time to give adequate cognition to those things, and so that's why we'd like the support stuff.

GP13
I: Not quite queuing out of the doors, or whatever. But then, you know, is it important to have this help then, in what ways does this help that workload?

R1: I think it's important, it does help, it definitely helps. It helps because the different directions of responsibility, so our prime responsibility is to the patient who's in front of us now. But, at the same time, we have a responsibility to our whole practice population, and to the country, of managing resources correctly. So, we have three different responsibilities that exist, almost like a holy trinity, they're all there at the same time, there's not one or the other. And so anything that allows you to focus on one, while monitoring your progress on the other, is likely to be helpful, or feels helpful. So, you know, if I have someone in front of me who needs an antidepressant, and the current evidence is that sertraline is better than citalopram but I didn't get to see that, if (the CDS)comes up and says, actually we're now supposed to be giving sertraline, I think, oh that's great, 'cause that's a helpful thing, 'cause it helps me monitor my other obligations at the same time.

GP13
I: Yeah, well I mean, that's actually a question I was almost about to go onto, was sort of what sort of trust and confidence do you have in the system?

R1: Sitting her in the context of an interview, a cautious, positive trust in it. When I've got 68 prescriptions to sign and it's eight o'clock at night, probably absolute trust in it, because I don't…[…] Yeah, because my brain, at that point, isn't ready to start analysing, you know, I'll trust it, and I've paid Defence Union fees. Which, that's another form of risk management isn't it, paying Defence Union fees. But, yeah, so probably anything varying from a fair bit, I don't like a scale, a fair bit of trust, to far too much probably, depending on the other things that are trying to attack my brain at the same time.

GP14
I: Yeah precisely. Let's talk through, about what you, how you use the system. Could you, I mean, this has come back into some of that stuff you were talking about with the patients and how you would respond to those alerts. Can you just talk me through, you're sitting there prescribing and alerts come up? What do you do, what happens?
R: So, if I was prescribing, I don't know for example, say I saw, I don't know, a sick kid or something like that, and I was prescribing some paracetamol suspension. I think, one of the alerts it comes up is that the sugar free suspension is slightly cheaper than the sugared one or something. So, it would come up as I'm prescribing, because you'll often click on the one on top of the list thinking that's the most commonly prescribed one, seems fine, and then it will come on and give you that suggestion. Then you read and take stock of that suggestion, patient still in the room, and go, yeah, okay, and then read and do your prescribing a different way and prescribe the alternative. Or sometimes, you'll go, well actually I'm not going to do that, and you can click ignore, and ignore it and carry on prescribing what you were going to prescribe in the first place. So, it varies according to the circumstance and what's popped up based on that.

Sub Theme 4b: Inner contexts – what influences what prescribes do- Balancing expertise, knowledge and information in decision making processes.

Sub theme 4b i:Expertise – (the CDS) as a knowledge base

Software developer staff
Software developer staff 1
R: But I like the fact that it appears at the very beginning of prescribing, not at the end. So if they've started a product, they don't have to go through putting a dose and a quantity, they'll appear before all of that. I like the fact that we reference everything. We do not provide any messaging, apart from a cost swap, and then you don't need evidence because it's like that's cheaper than that and it's the same product.

I: That is the evidence.

R: That is the evidence, yeah. And often, that's localised and we keep up to date with it. And we have a suite of people in (name of place), to do that, so I think, yeah.

Software developer staff 1
I also say it's the equivalent of having someone from the MM team, because we still have a lot of GPs that will still ring their pharmacist or the head of MM going, can you tell me the formulary status of this and what do I need to do for this. Because if they inputted all the stuff correctly in for that patient then, we would tell them that, it would save them having to pick up the phone. And it's learned behaviours. So yeah, it may have popped up a couple of times, but they will know for next time not to pick the brand that they've normally picked, and what they should do. So hit count will go down over time. And it shouldn't be seen as initiative. For a lot of MM teams, it's just a way to ensure that they can give really good advice out there when they physically can't be.

Software developer staff 1
I: I know I've heard before from clinicians they have a piece of software or…it's either a piece of software or a safety initiative, and it's "yet another thing", it's just another thing we have to do.

R: Yeah.

I: How do you…in terms of implementing it and getting it adopted and getting it into everyday practice, how do you overcome that? We'll deal with that question first and then I'll do the other question.

R: So a lot of this will either be at the engagement sessions or from the pilot. And the way that I probably do tend to sell it is that, you know, there's a whole wealth of information out there, in some ways it is a piece of software but the information within it, there's been a lot of time and effort spent on what content is in there. So before, with any other bit of software that they've had, it's just been authored by the medicines management team, they've put whatever they've wanted to in there and it's just do this, do that, whatever. Whereas we've carefully chosen our wording, we've carefully chosen what we will trigger on, what we might replace to. And the fact is that for the majority of our messages, because they're complex, which means they look READ codes, that when something does appear, it's normally appeared for a very good reason, and not because we want to annoy the GPs, because there's a clinical reason for that message to appear.

Software developer staff 1
R: One of the best descriptions of (the CDS) it's like having a medicines management pharmacist or technician sitting by your side, looking at your choice of medicine, going actually for this patient, you might not want to choose that; or what you're picking there isn't on our formulary; or hang on a minute, that's red; or whoa, look at their, you know, renal function. GPs have to be…they have to be a master at everything but they can't remember everything. Guidelines update on a regular basis. I think if you asked most GPs are they on top of all the new NICE tags, the new NICE clinical guidance, the new MHRA warnings. You know, if I think about, say, the (name of place) area, so I know that the GMMMG formulary has just been recently updated in December for the new COPD guidance. Now, would I expect every single GP to have…it would be nice if they all sit down and read those guidance and go, right, I exactly know which inhaler I'm meant to be using, what FEV the patient's meant to have before I initiate a steroid, when am I meant to use…we can't expect them to remember all of that, and that is when… […] Yeah, our software can pull all bits of information and at that point of prescribing, steer them in the right path to say actually, this is the type of inhaler, or, you know, if it's an asthma patient, a gentle reminder that yes, there may be a steroid on their prescription and a long active beta 2 agonist, but this asthmatic actually hasn't requested that steroid for 12 hours, but are always having their Salmeterol, that's, you know. It's more from the safety and best practice.

Software developer staff 1
R: Yeah, I mean, that's what I always say at a pilot is that this is decision support software, it's a support to make a decision, it's not going you must do. It's always please consider or, you know, are you aware. It's always worded in a way to say, just have a think about, you know. So whether it's metformin and they've got CKD stage four, you know, probably wouldn't recommend this…nationally, NICE say probably don't use this, guys. They can if they want to and if they're happy for the patient, that is their clinical decision to do so. And no one…that's the thing we need to get across, no one's going to hit them with a big stick because they rejected it, it's just they may not have been aware or they weren't aware that the latest U&Es indicated, you know, that their eGFR had changed.

Software developer staff 1
Guidelines update on a regular basis. I think if you asked most GPs are they on top of all the new NICE tags, the new NICE clinical guidance, the new MHRA warnings. You know, if I think about, say, the (name of place) area, so I know that the GMMMG formulary has just been recently updated in December for the new COPD guidance. Now, would I expect every single GP to have…it would be nice if they all sit down and read those guidance and go, right, I exactly know which inhaler I'm meant to be using, what FEV the patient's meant to have before I initiate a steroid, when am I meant to use…we can't expect them to remember all of that,

Software developer staff 2
R: For me the point is that medicine generally is much more complex and GPs are humans who work in a time constraint, pressured fashion, and can't possibly know every nuance of what's appropriate to prescribe at that point. Hopefully they know what sort of drug to prescribe, and (the CDS)helps to direct them to the most appropriate choice from a range for that patient. So it's a nudge tool, if you like. I had a customer describe to me once that it's a bit like a sat nav, it gives you advice and guidance but the prescriber is still driving. So it's there to support, not override. I genuinely think prescribers get benefit from it and do appreciate it so long as it's managed appropriately.

Software developer staff 3
I: Right, brilliant, okay, so can you just from the beginning, describe to me how (the CDS)was introduced, how did things get going with (the CDS)?

R:	Yeah, so the seedlings of (the CDS) probably pre-date me. So, I don't know if you're aware, but FDB or Hearst currently are an amalgamation of two different companies, what was FDB back in the day, and Map of Medicine. And we came together, probably about five years ago, but we were working quite closely before that. But, I guess FDB, for the last 20 odd years, produced data which has provided, what we called, core decision support. So, it does the drug-drug interactions, it does pharmacological equivalents, it does things like dose range check, and suggested dose. Now, that's been the mainstay of the business for a long time, and in a lot of ways we've been thought leaders and forerunner, in creating that data. But, I guess we've always been aware that, how our data is used, is sub-optimal, in the sense that we create all this information, but we are in some ways, arm's length from end users.

<u>CCG Staff</u>

CCG Pharmacist 2

But one thing that's transpired is, I don't know if I'm going off on a tangent here, is that some old, so like, I'm trying to think of an example, it was to do with ACE inhibitors use in pregnancy, or women of childbearing age, obviously that's quite, it's in the forefront of people's minds with the sodium valproate issue that's come out recently, and, 'cause the MHRA drug safety update, was talking about that recently, and it's a well known thing about teratogenicity with the valproate drugs in pregnancy. So, that was done as an (the CDS) message. But then one of the GPs highlighted that there was could have been a near miss, there was a lady of childbearing age, he was having a discussion with her, and she said, oh you're on Ramipril, has anyone told you that, if you do decide to become pregnant, that you need to let us know, because it could potentially have a problem in pregnancy? And the person was like, no, no one's ever told me. And so I think he was like, can we have an (the CDS) message for this? And then, that sort of opened up a bit of a, not a can of worms, but it was almost like, well where do you draw the line? There's loads of drugs that have got potential problems in pregnancy, but then equally it was flagged to me, that there had been a previous, MHRA drug safety update for this, which was prior to 2014, and apparently (the CDS) have only looked at MHRA drug safety updates, from the point that they came on board to the market. I don't know if that's right. Was it about 2014, sort of around? Or was it before?

CCG Pharmacist 2

R:	Good about it, it's there at the fingertips when they're having a consultation. The GPs having a consultation and you've got the information there and then. You've got links to local and national guidance to back you up. You can show it to the patient, no big secret. They can have a look. Look, this is national guidance. We're not meant to do this, and this is why. So, better compliance, maybe, with the patient, a better GP relationship. It's not putting the onus, this is the GP said they can't have this; there's a reason behind it

CCG Pharmacist 2

I:	So do you find that, do you find that GPs, if there's a lot of alerts in the clinical system already, if there's a lot of other alerts they just bundle them all together?

R:	The thing with Optimise, the safety ones, they shouldn't duplicate what's on the (GP CLINICAL SYSTEM) system anyway.

I:	No, no, they shouldn't no.

R:	So they should only fire up if it's not already on the (GP CLINICAL SYSTEM) system. But there was one recently that a GP has flagged, because nothing seems to be firing for [inaudible - 0:17:59] around over 65 year olds who have a certain antidepressant prescribed and you really need to look at the dose because there's risks attached to it. So (GP CLINICAL SYSTEM), and I've looked at this myself because I said I want to go and check this out myself, so I had a look. Warnings do come up but it's not specific to over 65 year old and increased risk of, I think it was bleeding and et cetera. So I raised that with (the CDS)but they said, oh yeah, there are, there is something on there but actually it's not specific enough. So I did a screen shot and sent that back to them for them to look at. So, I suppose, yeah, it's, I don't think it, well it's hard to say. Unless I go and interview loads of GPs it's hard to say.

CCG Pharmacist 3

The point of (the CDS)is it's to do with safety and quality, yes, it is cost, but I was actually doing a course when I started this job. So I used a formulary as my innovation project as such. And when I looked in detail at how we use the formulary and whatnot, I was able to identify gaps and problems. So along with doing that project, I felt what did we need to try and fill those gaps? And some of the things that I identified was first and foremost, the knowledge that we're expecting the prescribers to hold in their heads. It's just not feasible anymore. We're changing things constantly because new information is coming out, new decisions are being made. And they need something to help them, because they've got a lot of responsibility and they've got a person sitting in front of them, they're supposed to know everything about the patient, the best medication for them, but then also we're nipping away at their heels about cost. So we needed something there and it's supposed to be patient-centred care, so it's about looking at what is best for the patient.

CCG Pharmacist 3

R:	Yes, and apparently requests. A lot of people like it. I am still apprehensive about that because one of the reasons we went to (the CDS)was so that there wasn't that individual responsibility on one member of staff. Because the good thing about (the CDS)is that when you send a request for a new message you have a team of people that review it and they review it in line with national guidance.

I:	Yes, precisely.

R:	They also try to standardise the wording. And that is their full-time job. So they become experts at it. My staff that do that, that isn't their full-time job. So they can do the job well and adequately, but I am concerned and I have said this to Optimise, I am concerned about the – what's the word to use – it's not just responsibility – it's…in a way, it's about the protection.

I:	Yes, so that's…

R:	Because the thing is with Optimise, when you switch a message on, it automatically becomes available to every clinical system in the CCG. That is a huge responsibility.

CCG Pharmacist 3
And one of the key things for (the CDS) is for the prescribers not having message fatigue. Because if they get message fatigue, they're going to switch off. So they accept or dismiss but they're not looking at it or reading it. So we're trying very hard not to have message fatigue. So the (the CDS) Editorial Board are very clear about that, so if we send a message and they feel there is already an adequate message on the system, they won't activate it and they will tell us why. Now, if I've got somebody here, it's not their full-time job to do that. Yes, they will look around and see what national guidance is there but I feel there is an extra safety back-up. So I am concerned.

I understand why they've done it, but I am concerned with it and I'm not fully signed into that. So I'm very cautious of what I allow the team to write and alter, for their safety.

CCG Pharmacist 4
I: One thing I think we did talk about and one of the questions I've been asking quite a lot of people is what do you say is the point of Optimise? What do you think it's there for?

R: It's going to sound like quite an altruistic answer really. My argument is that I think the best prescribing is always the most cost effective prescribing. And I think whichever way we get to that goal doesn't really matter. It's only tools that you've got. So I think it's always appropriate to prescribe drugs in this class of patients, and I think it just helps to filter the views for GPs on that where… I think we talked about Pincer and I think Pincer's probably quite a good example, and I think where (the CDS)is good is it's at the point of prescribing for GPs. So if you're going to invoke any behavioural change, I think that's where we always struggle, by mopping work up you don't really make them think, whereas (the CDS)at least does make them think actually.

I: So you think the point of prescribing's more effective at making them think?

R: Definitely. And I don't think you can measure it particularly, other than through a surrogate marker of what the trigger rates are. But I think we probably are affecting some behavioural change. So I know we've done academic detailing before, I know the evidence is there to say academic detailing does… that's where Pincer comes in, doesn't it?

CCG Pharmacist 5
R: Yes, I do think I haven't come across a message that I've thought, that's wrong. I think they are very, very careful to make sure that the clinical content of their messages is right but I think the timeliness of them possibly isn't quite as good as it could be. It's getting better, I think, as…

I: In terms of the change to the messages or in terms of the way the…

R: Yes, so us requesting…

I: …[voices overlapping 32:53].

R: From the times it takes from us requesting a message to actually get the message or something changing nationally and for them to change their own messages, I think it doesn't work. It's not quick.

<u>GP Staff</u>
GP Nurse 2
R: So that was an area that I found, I suppose sometimes there can be too much information because I'm still very mindful of looking at my BNF. That was what the prescribing's all about, very much BNF is your bible and I'm still going like doing the old fashioned way, really.

I: So when you're looking for doses, dosages and things like [voices overlapping 03:48]…

R: I'll go to the BNF.

GP Nurse 2
R: Yes, and trying to…so, for example, this morning I had a patient and they were on sitagliptin and I know that our formulary would suggest alogliptin, so I've switched them over. Then, you know, if that wasn't, then I'd be looking at the linagliptin. So I'm very mindful of looking at what the eGFR is. If I could think of ways of improving then I think it would be quite good if you had more stuff for certain drugs, the monitoring, because that's not always on, is it?

I: Yes, okay. So more around the monitoring of…

R: Well, say for example, someone's on a ramipril drug for their blood pressure, one of the things we need doing yearly is U&Es. When a clinician is doing a medication review, not everybody is as thorough. So some people will keep reissuing it, reauthorizing it, but they've not had U&Es probably done for two or three years. So I think if there was something on that…

GP Nurse 2
R: Definitely, yes. That's why they've happily said that I can do the medication reviews on those because I'm probably quite thorough and I'll look. That's where you like probably pick up things like, well, they're actually on this DPP4 but actually their kidneys aren't working as well so they should be on another one.

I: How much does (the CDS) help with that review?

R: In that respect it doesn't link in terms of safety. So say, for example, one of the DMARD drugs methotrexate, I don't think there's any way you see that that would… So for that you need to have…

I: Yes, the monitoring one should come up because it's a PINCER indicator.

R: The monitoring one comes up, doesn't it? Yes, it does, but I don't know if it specifically links to check...

GP Nurse 2
I: Yes, you talked about the meds management team earlier there, do you have quite regular interaction and contact from meds management?
R: Well, they've got all the formularies on the guidelines so, say, for diabetes, and we have regular updates but the meds management (names of two CCGs) just because it's not on their formulary, I wouldn't take it as I'm not allowed to prescribe it. I'd take it as I'll use my clinical judgement if I think it's best for the patient because sometimes they're a bit behind on the ones that potentially are the best choice for the patient. So, yes, just because it's the cheapest, I wouldn't necessarily prescribe it.

GP Nurse 3
I: Yes, precisely. Yes. And in terms of the system, would you say you had confidence in it or trust in it?

R: Yes, I would.

I: In what sort of ways? What sort of gives you that confidence?

R: I've not found it to be wrong, so yeah.

I: Okay. Yeah. So, you've not seen messages that are…

R: Outrageous, not at all. No.

I: No.

R: They've always been relevant.

GP Pharmacist 2
I: Yes. And so, because you're not yet prescribing, perhaps we can think... do you think from what you know of the system, once you're fully prescribing, how do you think it's going to fit in with your work then?

R: Like, I mean, I'm grateful for the clinical systems, like, I think if it makes you look at something in a bit more detail, and yes, like that's what I was doing today, because we, you know, we had to prescribe a NOAC for a patient, and you know, (GP CLINICAL SYSTEM) flagged up, (the CDS) didn't, actually, no.

I: But if one fires, the other one shouldn't really. Yes, it shouldn't duplicate

R: Right, yes, so (GP CLINICAL SYSTEM) flagged up with, you know, a read code, because, obviously, all of these systems rely on good imputing, don't they. So, if the records don't hold that they've had a re-code of oesophageal varices, for example, then you know, you're going to prescribe that drug, and that extra safety net is not brought to your attention. So, you can't like, look into things and try and assess that, the risk, actual risk to that patient, and make a decision. So, you know, yes, but then maybe I've a little bit of luxury of time this morning, but if you've only got a couple of minutes, then…But that's where you get the difference between an experienced prescriber and someone whose less experience, right?

GP Pharmacist 2
I: Do you trust it?

R: Yes. I do know some clinicians have said, oh, that it's picked that up and it shouldn't have, but actually, when you look at it, it's probably inputting something that needs to look at as a read code, rather than (the CDS) being [voices overlap 23:03].

I: Yes, because you said about the read coding and how important that is, you know, because...

R: That's how it pulls it in.

GP Pharmacist 3
R: It's a clinical need for wanting sustained release metformin for that patient
I: So, is that specific to individual patients or is it something that you just feel that that should be how it works.
R: No, that's actually…
I: …or is that driven by the person sitting in front of you, is what I'm asking?
R: That's driving by the person sitting in front of me because when they've come in and sat down for their diabetes reviews, one of the first question I ask is, are you tolerating your metformin. I won't ask them why I'm asking that question, it's to find out if they are because as far as I am aware I think, only eight out of ten people tolerate normal release metformin.
I: Right.
R: The other two out of ten won't say until you ask them, and they just think they have to gun it through.
I: Yeah.
R: So, I don't mind a warning coming up on (the CDS) 'cause it tends to be quite financial driven. But if there is, a way of switching that off it would be nice.

I: Yeah.
R: Because it's a clinical, need.
I: Yeah.
R: I don't mind the recommendations but sometimes again, you get click fatigue.

GP3
I: To finish up with, do you have confidence in the systems to do…?

R: Yeah. Because it always seems to be it references back to probably the right people, and it goes back to the fact how often I disagree with this rule. That is pretty rare. And I can't think of too many systems that I would disagree less with.

I: Yeah.

R: It's well referenced, and that gives you some confidence I suppose. And there's not even one out and out wrong that I could say is wrong. I could disagree with it a bit and there could be a debate about it, but it would be a debate.

GP4
R: So you're actually at that point when you're prescribing, so it's not popping up at a point in the consultation when it's removing your focus from something else you're supposed to be doing, it complements the activity.

I: Yeah, that's really interesting, yeah.

R: I don't find that a problem.

I: So you don't find it coming in at the wrong time?

R: No, so it's only generated at the point when you try to prescribe something. It's not popping up in the middle of you taking the history about something else.

GP5
And by providing us an easy way of being able to follow through the links, if we weren't sure about, why should we change it, so I remember when I first started, the ability to be able to go and see what GMMMG had agreed for instance, was really nice, because then you could see the governance behind it. 'Cause otherwise you might think, well it's some manager in the CCG that's seen a drug rep, and suddenly decided to change things, and go, why should that be the case? So, it's kind of nice to be able to do that.

GP7 and GP8
I: But going on to that learning thing, which gives you the best learning, does (the CDS) or PINCER?

R3: PINCER because that's the safety bit. (the CDS) is not safety for us, so PINCER is the audit, which makes sure we're prescribing in line with safety guidance.

R2: In terms of learning the new, it depends what you say you're learning doesn't it? Because if you're learning about patient safety stuff then obviously PINCER, but if you're trying to learn the vast, you know, formulary of what you should be prescribing then (the CDS) teaches you that because it just, it just tells you what you should be prescribing instead of that. Which, you know, realistically we can't, well I can't look up every single drug that I'm going to prescribe and check it is the first line on the formulary. There's little things that, the little icon that says it's on the formulary or not but if you've been asked to prescribe this specific thing by a consultant you go for that don't you unless there is like a generic, you know, if there's a generic version or there's a different drug that's exactly the same that's gonna be a lot less money, then that's why it's really useful when that comes up.

GP7 and GP9
R1: It just pops up every time you prescribe something yeah.

R2: I mean I, I think it's good because it does, it tells you all these switches doesn't it, which otherwise just, I remember when I was a registrar being told, oh, you should prescribe this instead of this, and you can't possibly hold them all in your mind, can you? So from that point of view I think it's really useful.

R1: Unless you get the wrong switch where you're told not to switch. For example, [Instillagel 0:02:08] and the alternatives because the nurse can't claim it back if we switch it as what (the CDS) tells us to do the practice can't claim it back or anything like that. So, there's a few drugs like that where…

GP7 and GP9
I: Have you seen, I mean we were talking about earlier and introducing things, about the prescribing safety indicators that are measured in the PINCER, so they would be thing around the exacerbation of asthma, and things like, the beta blocker and aspirin is one of them. Things around avoidance of gastric bleeds with things like NSAIDs in the over-65s that's one of them. Or NSAIDs with a history of an ulcer; so have you seen any of those coming up through the (the CDS) system at all?

R2: I don't think so.

I: Or do you see any of that around those sorts of things?

R1: I can't remember but those patients tend to be already on a PPI…

R2: We've had PINCER alerts sent to us by our practice pharmacist because she is around PINCER things and said, can you look at this one, can you look at that one? But I don't remember seeing them on Optimise.

I: Perhaps you're doing them so well in PINCER that they aren't coming up?

R2: Well to be fair she does, she does keep on top of all the PINCER stuff doesn't she quite regularly? And she sends us all individual task things, can you look at this one? Can you look at this one?

GP9
R3: But that's when you then re-learn, because honestly five or ten years ago we were being told at prescribing meetings to prescribe this brand or to prescribe that generic and we couldn't remember any of it. And now it's just simple straightforward, you just go through it.

GP10
Variety really. I think if the alert comes up, one thing's in viewing the highlights, is the importance of the interaction with (name of CCG pharmacist). And I suppose if the alert is bringing something up that Jill's said, it just reinforces it a million times. If the alert is bringing up something that is not on the radar already, then we always check to see why…or I personally, I'm sure…to check why that is, why it's coming up. But occasionally like this morning, there was one that it specifically…it wasn't a consultant but it was one of their specialists has asked for very specific medication that normally wouldn't be given, and of course the (the CDS) comes up with that immediately as, this is not the one that's first line or appropriate or whatever. Now, as it happens, that message from the nurse specialist was only in this morning anyway, so I knew straight away that I could override that. And then you get the ones where… So those are the most common, leaving just a smattering of a few that come up with, this is not appropriate or this is not following guidelines or whatever, where actually they've had it for yonks and the trouble of changing it probably far exceeds any benefits or any real gain. I mean, you have to make that decision whether it's raised at the time and obviously if it's something that's you know full well costing a small fortune or it really has been proven to be effective then we do look at it, but there are somewhere you go now, it's a regular thing and been had for 20-odd years, I isn't going to go there to change it now, yeah.

GP10
R2: I think while ever it's not overly-intrusive, i.e. I suppose almost intrusive to the nature of feeling obliged to go down the recommended course rather than your own judgment. I think 'cause as long as it's not intrusive to that extent, then there's no reason to turn round and say, no. There'd be no reason to turn it back on I can't see. Unless I thought the CCG had its completely own agenda, which I don't think's true. That would be the only other issue, if they'd lost the vision of the health service, then I might be tempted to say no, but I can't see that happening personally.

GP13
R1: So, I think, (removed potentially identifiable material) but the cost side is probably more important on a day to day basis, than the safety side. But it's nice to know that, we sort of have a mix now, with the (GP CLINICAL SYSTEM) flags up every possible little safety problem, but if (GP CLINICAL SYSTEM) and (the CDS) agree, then you probably really ought to look at that.

I: They shouldn't duplicate? If one fires the other one shouldn't, but it probably does, I have no idea.

R1: I couldn't say if that happens.

I: I think that's what we've been told, as to how it operates, but…

R1: Right, okay, it may well do. I mean, that leads to an interesting point, (the CDS) seems to work without the need for a vast amount of cognitive input into it, it's there when you want it and…well maybe it isn't. When it's there, you think, okay, I'll think about that, when it's not there, and I'm thinking as I'm talking, but maybe now if it's not there, then we assume everything's okay, and that could then be a hole in the safety net, I suppose, it could be one of the holes in the Swiss cheese, that's waiting to line up with a couple of others […] So yeah, maybe it's almost like we rely on it too much.

GP13
I: Not quite queuing out of the doors, or whatever. But then, you know, is it important to have this help then, in what ways does this help that workload?

R1: I think it's important, it does help, it definitely helps. It helps because the different directions of responsibility, so our prime responsibility is to the patient who's in front of us now. But, at the same time, we have a responsibility to our whole practice population, and to the country, of managing resources correctly. So, we have three different responsibilities that exist, almost like a holy trinity, they're all there at the same time, there's not one or the other. And so anything that allows you to focus on one, while monitoring your progress on the other, is likely to be helpful, or feels helpful. So, you know, if I have someone in front of me who needs an antidepressant, and the current evidence is that sertraline is better than citalopram but I didn't get to see that, if (the CDS) comes up and says, actually we're now supposed to be giving sertraline, I think, oh that's great, 'cause that's a helpful thing, 'cause it helps me monitor my other obligations at the same time.

GP13
I: Well, that's interesting isn't it, 'cause I mean, that sort of brings us back to what I was saying before, or what you were saying before, in terms of, that you're using that skill, knowledge, expertise of yours, as a doctor, as a GP, do you rely on these things too much, what's the balance, if you like, between your expertise and the systems?

R1: It's like a self-driving car isn't it, who's fault is it if it crashes? […] I mean, I think as GPs, we know, or should know, that if we sign the prescription, then it's our responsibility. But, I don't know, I think if you had a semi covert observation of my practice, I know that if I get an (the CDS)alert, I'll read it, I think, a 100 percent of the time, I think 100 percent of the time. I'll dismiss it without any further thought, 20 or 30 percent

of the time, and then I'll give it some proper thought the rest of the time, sometimes ending up in action. But, I think what I don't know, is how many prescribing decisions I make, where (the CDS)doesn't flash up, when had it been programme differently, it would.

I: Well yes, if there are other messages there, yeah.

R1: And then I'm thinking, and it will be subconscious, because when it's not there I don't think about it. I was slightly anxious about, I didn't know what we'd have to talk about today, 'cause (the CDS)is in my consciousness when it's on the screen, and then it disappears, which it wasn't when it was too slow, but now it is, it's just there or not there […] So, I think so it's the other side of the argument then, how do you capture, you could easily observe my day, and work out how (the CDS)affects me when it's there, but it will be much harder for me to see how it affects me when it doesn't fire. Yeah, that would be harder, and whether I am subconsciously thinking, this is okay, 'cause nothing's warning me it's not […] I've at least one example of that. We had an elderly lady in a residential home, who was on 40mgs citalopram for her anxiety, and I think a medical student, or our pharmacist did an audit, or something came from the MHRA, I can't remember, anyhow it flagged up, we ended up doing a search. And it transpired that I had a knowledge gap, and I wasn't the only one, that 20mgs was the maximum dose you should be on, if you're over a certain age of citalopram. Now, as far as I know, (the CDS)had never told me that, and (GP CLINICAL SYSTEM) had definitely never told me that, because I went in and tried it. And she'd been having that for some time, without any, and I'd been signing her repeat prescription. So, I guess my behaviour had been, there is no warning, it must be okay.

GP14
I: No. But in general, then, so, you will use your…what's driving that clinical judge…what's driving that decision making, if you like?
R: The drive is doing what you think is best for the patient and what's going to make them better whatever the situation is. If they're poorly, or we are treating their chronic disease well or whatever. So, obviously, that relies on your own experience, skills, confidence, that sort of thing. I think probably as maybe one of the relatively older GPs, having lots of, having a reasonable amount of experience and confidence in what you're doing, probably means that you cancel it off more often than a younger one. Don't know. It probably varies.

GP14
I see it as an adjunct, a positive adjunct, rather than…because I don't just type in what I think and then wait for the computer to come up with the answer and prescribe whatever they say. I've already got my idea of what I'm wanting to prescribe and it only comes up as an alert after you've decided to try and prescribe that and if it comes up saying, well, no that's potentially not safe, have a think about it and you follow that guidance, then that's great. It's given a positive effect for the patient's safety or cost effectiveness or whatever it might be. So, I see it as a positive thing.

Sub Theme 4b ii: Broader decision-making process

CCG Staff
CCG Pharmacist 2
So, the reporting is good, but it won't pick up everything that's going on in the surgery and what final decision has been made at that point.

CCG Pharmacist 2
Yeah, but we have had some where they've said, like, the pharmacy can't get this product, and then I can look into that and contact the pharmacy, so if I know it's, say, for example, a (name of place) practice round here, I know which pharmacies they'll probably use. I could contact them and say, is there a problem with this? Often, it might just be a localised problem with their own wholesaler, but the pharmacy down the road can get it, so then I can feed that back to the surgery.

CCG Pharmacist 2
R: Yeah, it's happening, yeah. I don't know, I've heard the odd thing from colleagues, I don't know whether you guys are going to be talking to patients at all, but you do get the odd rumblings of patients, sometimes get a bit fed up with, oh I had this last time, and I've got this, and I've got this, and now I've got this. You know, it's like different switches all the time, and the products change, and they wanted to keep to that brand, because it worked better, and it tasted better, or whatever it is, sort of thing. So there is that, being mindful of the patient at the end of the day, and making sure that it's not all become so cost orientated. And it's being mindful of, I mean we were having a discussion yesterday at our regional meeting, about, sometimes, safety and quality interventions aren't going to save you money. If you think about adding a PPI, you're adding something on.

I: Yes, precisely.

R: You're not taking something away, but at the end of the day, it's all about looking at the bigger picture of safety for that patient.

CCG Pharmacist 2
R: I mean on the plus side of all of it as well I mean obviously this isn't something that (the CDS) would be able to pick up on but on the flipside of it is if the NSAID gets stopped, please also stops the gastro protection. You shouldn't really be on it. Again, it's that whole holistic review of a patient, really, as well that's really needed alongside all of these alerts because you're only going to get one aspect of it, really, yes.

CCG Pharmacist 6
R1: And I think that that's why we've been really... So, like, for example, the self-care messages, there's a whole piece of work that's being done around self-care, so we've tried to actually use (the CDS) as an add-on to all work that we're doing, to support that.

I: And that's really interesting. So it becomes not just…

R1: An isolated tool, yeah.

I: It becomes part of the work that's being done.

R1: Absolutely. So like the self-care work that we're doing, we've been targeting like hay fever and emollients have been set up, and so what we'll do is we'll do a rolling turning on of messages, and off, so that we don't turn all the self-care messages on in one go, so it doesn't cause message fatigue, and we'll tailor it to what work we're doing. So if we're actually looking at a couple of areas in self-care from a practice pharmacist doing the work and the practice's point of view, we will then turn the messages on in Optimise, to support them in doing that. And then once that's been done, we'll turn them off, and then turn another few on, so that the GPs aren't bombarded with messages all the time. And it's actionable messages. The feedback we've got from GPs is the messages need to be actionable.

GP Staff

GP Nurse 1

I: How many times are you getting those sorts of alerts when you're prescribing?

R: To be fair, I don't get many alerts, it might be to do with my prescribing because even though we have the FE300 we all prescribe within our competence so I'm not sure. But it's usually the usual drugs for chronic disease and the drugs for pain relief and that sort of thing. So there doesn't seem to be a lot of problems but I think it's because the education that we have I wouldn't prescribe something in the first place because I would look at what the patient already has. So before I prescribe I'll look at have they had it before, what are they currently on, and then I'll look at renal function et cetera.

I: So you're already doing that before the system is alerting you?

R: Probably but because my turnover of patients is not as great so I'll have patients that I know because the caseload stays fairly static, well, not completely static but it's more static than if I was working in primary care. So it allows me to do that because I have a more holistic approach because I'm having the time to do that, and because I'm a nurse and I think we just get a bit more scared of making a decision.

GP Nurse 1

R: Well, community matrons, we're a band seven and we work with long term conditions, so we have the more complex, long term conditions and my particular role in this practice is to work with the care homes where a lot of our vulnerable, frail patients are. And that's to allow continuity and, again, it allows us to look at...when you've got the continuity you can look at the patient holistically because you get to know them. And that's why the caseload stays, it moves, it's a little bit fluid but it's a bit more static than maybe others. So I look at each patient holistically from their disease management into their prescribing, so we do de-prescribing as well. And so a lot of my prescribing will come around long term condition management such as, I don't know, COPD or heart failure. Then there's the acute management, so it might be infections or skin conditions or bowel management.

GP Nurse 1

R: Yeah, because it's when I'm going to be introducing a new drug so I'm just trying to think when I would, it's a difficult one, I don't think it's going to be more than weekly, I don't think based on what I prescribe for acutes.

I: Do you think that's because of, A, either what you prescribe...

R: I think it's to do with what I prescribe and my caseload being fairly static.

GP Nurse 1

I: And I think you talked about it, but I think one thing I found was really interesting in what you said was this holistic approach because I think...

R: I think we have more than ten minutes and we don't have the vast range of patients, I've got a certain group of patients and there comes a pattern with the prescribing and so you know what you don't prescribe and what you do prescribe, it becomes automatic because you're just dealing with that one group of people.

I: That's really interesting as well.

R: Where if I've got to think about...if I had to go and see, I don't know, a 30 year old, young man with different health issues I'd have to think a lot more because that's not what I'm used to. And I think as a GP or an ACP there's a vast range of people, you could get anything come through.

I: Precisely, you don't know what's going to come through the door.

R: Exactly, and I know my day's quite predictable.

GP Nurse 3

I: Yeah, it picks it up. Do you find that a useful thing then that it's linked to that patient?

R: Yes, because I can then go back into it and see exactly what it is.

I: Yes. So, would you then go into…

R: I would check the results. I would go in and check the result.

I: Right. Okay. So, it's a…working as a prompt in that respect to do those sorts of things?

GP Nurse 3

I: So, what are you…why are you doing that then? Why are you just not going, oh well? It's told me to do that. I'll do that.

R: Because sometimes it may mention going back to the EGFR again. Don't use in patients with EGFR below such a thing, but then when I go into actually check it, the EGFR is above the threshold, so I could still use the original.

I: Yes. So, you've got a…

R: Then other things, it might say, they're on such a thing. It's an interaction. In that case then I would find something different, so it really depends on what the message is, what I'd do with it.

GP Pharmacist 1
R: So, my approach is probably slightly different because I don't do diagnosis on any acute things. All of the patients I see are chronic disease review, medication review, so I am actually more focussed…

I: You're probably seeing more of those patients, yes.

R: Yes, so I'm more focussed on actually dealing with those complicated things that the GPs don't have time to deal with when they're seeing the patient acutely because there's something actually wrong with them. You know, they're coming in to see me routinely because it's their annual review and that's what I'm dealing with. So, I can't think of an occasion where I've had lots of different (the CDS) things on one patient. It's more a case of where a thing comes up that you disagree with because of x, y and z else that's going on.

I: Right, so the other bits of it.

R: Then perhaps the multi-morbidity or the frailty or the social situation of that patient or whatever it is means that I'm not going to do what it wants me to do for whatever reason.

I: Yes, because you've got to think about what? That patient?

R: I've got to think about the patient as a whole, not that just individual thing.

GP3
I: So the cost reminders are more niggling than…??

R: Again, they are. I know the policy, if I decide that I'm not going to follow the guidelines, and they are guidelines, so be it, that's my responsibility. As a GP it's still fundamentally way under budget. I've shown that I'm a competent prescriber. So I do get niggled by it a bit, and there's certain ones that I understand why they say that they're not cost effective and things like that, but I'm sometimes not prescribing them for being cost effective, I'm prescribing them for overall health budget. So rather than refer someone or ask someone to come back I'm doing no harm is the way I'm looking at it, and I might be prescribing something that cost £2.16 rather than £2.05, but I'll live with that.

I: Yeah. Because, as you say, the savings on hospitalisation on people being referred to outpatients…

R: The local one that I strongly disagreed with was the Gaviscon being converted to Acidex, and all you've got to do is taste some Acidex and you'll discover why everyone's been changed over, they've all come in and had appointments to see me, which is not cost effective.

GP4
R: Well, a good example might be particularly palliative care. And so you now get an elderly chap with prostate cancer that's spread to his bone and you know that an anti-inflammatory is probably going to be a good option in terms of the thing that might be likely to help, but they've got impaired renal function, they're 82 and all right, you give them the PPI but, you know, it's one of those ones where…and I might for example use Diclofenac in that case because I've found that Diclofenac, I'm less worried about cardiovascular risk perhaps in that patient, and I find that [inaudible 20:39] seems to affect a…tolerated from the GI side of things so I make a non-formulary decision based on all of those factors. And I think, it's an end of life or palliative care situation, so I suspend some of the cost concerns or some of the safety issues potentially that would be relevant for somebody younger or fitter or in different circumstances.

GP5
R1: Well, yes, you see, the thing about the NSAIDs and over 65s, it's a good one, and SSRIs, is that with the austerity measures, I don't want to make this political, but with the austerity measures, and cut backs and all the rest of it, and more and more difficulties with getting into services and what not, there's a massive increase in depression and anxiety, big increase in back pain and musculoskeletal problems, and expectations that everyone wants everything done yesterday. So, there's a good example of, okay, 'well which one do you want? Do you want the painkiller or do you want the antidepressant?' 'Well, actually I'd like both doc.' Okay, well there's going to be a slightly increased risk of, but at the end of the day, if you don't do this, I'm going to be suicidal doc, or I'm going to lose my job. So, it's an example where, actually it's the social determinants of health that are causing the problems, and all that's happening is (the CDS) is telling me, or the system is telling me, look out. Yes, we know that, but actually the answer isn't about non-steroidals and antidepressants, it's actually about, how do we sort out the social determinants of health?

GP5
R1: NHS is in fire-fighting mode. And most of my GP colleagues are trying to get out of GP surgeries, and going off and doing something different. Social prescribing. There's a lot let risk there than there is in prescribing drugs.

I: For what reason, then?

R1:	Yeah, absolutely. If I send you to go off to do some dancing and singing, you're not going to complain about that. But, if I prescribe a drug to you that causes you to have renal failure, well that's a complaint. So GPs figured that one out, so let's go off and do social prescribing. That's why you'll hear a lot about social prescribing, and, isn't that wonderful, and it's great, because as long as I refer you for social prescribing, there's no risk, or there's very little risk.

I:	So, is there a sort of…?

R1:	So, (the CDS) needs to direct people to social prescribing. That's how you reduce risk. It sounds crazy but…

I:	No, not at all.

R1:	…I can tell you now, that's exactly where things are going. You know? And it's a lot less stressful, and patients love coming back and saying, oh doc, I joined…you know, no one ever thanks me for prescribing a blood pressure tablet, but if I send them off for a singing thing, or a dancing thing, they'll come back a month later, saying, you've transformed my life.

And that, again, indicates that these are basically social determinants. These are patients who don't need another tablet. What they've got is loneliness. And they need to meet people. Which is why social prescribing is becoming so big in communities like this, because it's not tablets that they need. So, in a way, Optimise, every time you type in an anti-depressant, it should say, have you considered social prescribing? Now, if I was doing safety, from an holistic perspective, that's what I'd be wanting to point to, not GMMMG […] In other words, why are you prescribing a tablet? You should be prescribing talking therapies or whatever it might be […] But, that would only come from people who are looking at it holistically, rather than just, I'm here to do a medication thing, and this is to support medical prescribing.

GP5
R1:	Well, yes, you see, the thing about the NSAIDs and over 65s, it's a good one, and SSRIs, is that with the austerity measures, I don't want to make this political, but with the austerity measures, and cut backs and all the rest of it, and more and more difficulties with getting into services and what not, there's a massive increase in depression and anxiety, big increase in back pain and musculoskeletal problems, and expectations that everyone wants everything done yesterday. So, there's a good example of, okay, 'well which one do you want? Do you want the painkiller or do you want the antidepressant?' 'Well, actually I'd like both doc.' Okay, well there's going to be a slightly increased risk of, but at the end of the day, if you don't do this, I'm going to be suicidal doc, or I'm going to lose my job. So, it's an example where, actually it's the social determinants of health that are causing the problems, and all that's happening is (the CDS)is telling me, or the system is telling me, look out. Yes, we know that, but actually the answer isn't about non-steroidals and antidepressants, it's actually about, how do we sort out the social determinants of health?

GP5
R1:	Websites, information sites. The problem is GMMMG website, is constantly changing, but that's not being passed on. In a previous life I was the Map of Medicine clinical lead for the north west of England. Now that was the map of medicines, that wasn't just medication. 'Cause that's the issue with Optimise, it just doesn't go far enough. It's just purely about prescriptions. When actually, you want it around chronic disease management, and all the other bits and pieces, which is basically what the Map of Medicine would have been. So, although, yes, (the CDS)is good, it won't take too many brain cells to figure out, but why are we just limiting it to medication? Because I'm not a pharmacist, I'm a doctor.

GP6
Oh yes, no I like that, that's fine because actually if they've ever had a tendency to wheeze I'm going to be very careful then that if I'm going to still give them Propranolol I'm going to be very bloody certain I've warned them about the possibility of wheeze and made very certain because, you know, I'm doubly certain, more so than I would to somebody with no history of a wheeze.

GP6
R:	I think, yes probably because the human brain is so clever, I probably do actually pick out a something if it's actually relevant and useful and I think that's partly why this torsades de pointes business has been a problem because it's fugging them all up now.
I:	Right.
R:	It almost appears on every single alert. So that would then switch you off necessarily to [inaudible 19:00] everything else but I think probably, it's a weird thing but probably anything that's in the middle of the screen actually does make more of an impact in your mind than anything else.

GP6
If it's not, God forbid. But, I mean, we used to customise them ourselves, the CCG with Script Switch that used to happen. So for instance, if you went to prescribe Dosulepin and, like, these old fashioned tricyclic antidepressants, the system would pop up saying, please just do not do that, that is just not a useful appropriate drug to be giving. And I haven't prescribed anything like that for so long, I don't know, but that's the point with those, sorts of, alerts would be very important.

GP8
It is extremely complicated when there's multiple comorbidities in a patient. The actual warnings are…there are a large number. The nice thing with that, of course, is they're, sort of, star rated so you can give a quick flick down and just pick out those with the three red stars as opposed to the ones…Yes, because they're the ones that are absolutely critical. So yes and no. I think the issue there would be, I wouldn't… Personally, in a normal consultation, I can't afford to read every single one of those but I will look specifically at those that are marked.

GP10
Yes, so for that example you re-challenge, perhaps re-challenge, I mean it's probably a brave person if you've had a problem with them first time but anyway you re-challenge again and you have exactly the same side effect again then obviously that's then coded […] Yes, then at that point you wouldn't go down that line again […] There is a period of time and quite…you know, with most medicine, there's a period of time where there's very little that actually happens black and white overnight, especially in general practice. Much more in hospital-based, yes, maybe it does overnight and you can get a definitive change but here it's a change usually over time or progressive challenges. There're too many other factors

involved in general practice for it to be considered just the sole cause so you can never really, on one event, you know, code straightaway […] So, you're left quite often with grey areas of patients for a period of time until, you know, time will tell you whether that's the case or not.

GP13
R1: And, I think, we had a meeting yesterday about skill mix and what have you, and that's probably one of the issues in general practice, is that we all try and do everything, whereas in fact, what we're trained to do as doctors, is to do the difficult stuff, being pragmatic, the balancing two, three, four, competing risks, and working out what to do despite them, and how to manage them, and how to safety net them and all that sort of stuff, and (the CDS) definitely helps from that, because anything that helps remind you of what the risks are, helps you manage them better. And so, what might have been, 15 years ago, a prescribing error, you could do exactly the same thing, but at least you're doing it as a consciously managed risk now, rather than as a prescribing error.

I: Yeah, rather than just simple, oh you shouldn't have done that, it's a, well there's a decision process towards that, and it's around…?

R1: I can give you an example. So, we recently had a woman in a nursing home in AF, who 15 years ago, would never have been anticoagulated, because she sits in a chair in a nursing home, and is hoisted and stuff like that. But, because of decision support, not just Optimise, but various methods of decision support, and [inaudible 00:12:36] she was put on rivaroxaban for her AF, and it was managed appropriately, and then she fell, and she's recently died of an intracerebral haemorrhage. But I think 15 years ago, we would have been thinking, oh blimey, what did we do there, was that the wrong thing to do? Or, if she'd had a stroke and not had it, we would have gone, oh there we go, that's the… Whereas now we can go, actually, that was still the right management, she wasn't falling, she had AF, so whilst she's had a bad outcome, but actually she's been on rivaroxaban five years, and she might have had a stroke three years ago, and been disabled. So yeah, so that's an example of how, 15 years ago, it could have just been not even noticed as poor prescribing.

I: Yes, I'm with you.

R1: Whereas now it's a managed risk, which happens for that individual, sadly, to have gone wrong, but you can look back and say, actually, that was still the right thing to do, 'cause that's a…

I: Yeah, rather than simply, we don't know what we did, because the system has been able to track that through as it were.

GP13
R1: So, I think so it's the other side of the argument then, how do you capture, you could easily observe my day, and work out how (the CDS) affects me when it's there, but it will be much harder for me to see how it affects me when it doesn't fire. Yeah, that would be harder, and whether I am subconsciously thinking, this is okay, 'cause nothing's warning me it's not […] I've at least one example of that. We had an elderly lady in a residential home, who was on 40mgs citalopram for her anxiety, and I think a medical student, or our pharmacist did an audit, or something came from the MHRA, I can't remember, anyhow it flagged up, we ended up doing a search. And it transpired that I had a knowledge gap, and I wasn't the only one, that 20mgs was the maximum dose you should be on, if you're over a certain age of citalopram. Now, as far as I know, (the CDS)had never told me that, and (GP CLINICAL SYSTEM) had definitely never told me that, because I went in and tried it. And she'd been having that for some time, without any, and I'd been signing her repeat prescription. So, I guess my behaviour had been, there is no warning, it must be okay.

I: Yes, I'm with you. So, yes, and in some respects, that's really the other side of these alerts isn't it? If you're not getting them, your assumption is, everything's fine, I can go ahead, I can proceed. But it might just be that the messages haven't been set up.

R1: Yeah, and that's harder. And in fact, then what happened with that lady is we went and had a discussion with her, saying, you need to reduce this, and she and her attorney said, really, can we not just stay on this dose, because last time she tried to reduce it everything went mad? And we sat down and had a discussion and did the whole balance risk thing, and she stayed on 40 in the end, which was interesting. But that was then one of those managed risks, rather than, if a month earlier she'd had a cardiac arrythmia and died, that would have been a mistake.

I: Yes, I'm with you, yes, yes, precisely.

R1: But it then became a managed risk, which is okay. A managed risk is fine in medicine as far as I'm concerned. You know?

Sub-theme:4b iii Expertise and Prescriber Knowledge

Software developer staff
Software developer staff 1
R: One of the best descriptions of (the CDS) it's like having a medicines management pharmacist or technician sitting by your side, looking at your choice of medicine, going actually for this patient, you might not want to choose that; or what you're picking there isn't on our formulary; or hang on a minute, that's red; or whoa, look at their, you know, renal function. GPs have to be…they have to be a master at everything but they can't remember everything. Guidelines update on a regular basis. I think if you asked most GPs are they on top of all the new NICE tags, the new NICE clinical guidance, the new MHRA warnings. You know, if I think about, say, the Manchester area, so I know that the GMMMG formulary has just been recently updated in December for the new COPD guidance. Now, would I expect every single GP to have…it would be nice if they all sit down and read those guidance and go, right, I exactly know which inhaler I'm meant to be using, what FEV the patient's meant to have before I initiate a steroid, when am I meant to use…we can't expect them to remember all of that, and that is when…

I: No, precisely.

R: Yeah, our software can pull all bits of information and at that point of prescribing, steer them in the right path to say actually, this is the type of inhaler, or, you know, if it's an asthma patient, a gentle reminder that yes, there may be a steroid on their prescription and a long active

beta 2 agonist, but this asthmatic actually hasn't requested that steroid for 12 hours, but are always having their Salmeterol, that's, you know. It's more from the safety and best practice.

Software developer staff 1

R: Yeah, I mean, that's what I always say at a pilot is that this is decision support software, it's a support to make a decision, it's not going you must do. It's always please consider or, you know, are you aware. It's always worded in a way to say, just have a think about, you know. So whether it's metformin and they've got CKD stage four, you know, probably wouldn't recommend this...nationally, NICE say probably don't use this, guys. They can if they want to and if they're happy for the patient, that is their clinical decision to do so. And no one...that's the thing we need to get across, no one's going to hit them with a big stick because they rejected it, it's just they may not have been aware or they weren't aware that the latest U&Es indicated, you know, that their eGFR had changed.

Software developer staff 1

And again, I go across what the average acceptance rate is, so, you know, whether it's a cost message or a best practice. We normally say, I think, 30 for best practice, 20 for cost. Or actually, it might be the other way...no, it's the other way round, I think. I'll just say an average between 20 and 30. So they know that one in five...one in four messages they'll accept, the rest they don't have to. That actually puts them a little bit at ease, because the trouble is, is that some organisations will put an incentive scheme on acceptance rates. And it's the worst thing you could possibly do, because you're forcing a prescriber to accept something that they may not agree with or may not be clinically appropriate. I mean, I think they tend to do it more on cost messages because it's sort of they're a bit more no-brainer. It's harder to make a decision on a safety message when they may have already considered things. But I do that across as don't feel you have to accept...I said, I'd be worried if you did have a high acceptance rate because I'd be, like, are you actually reading the messages or feel that you should just accept it because it looks best practice, you know, you're still the prescriber, that's still your decision to make, we're just giving you a little bit of extra information.

Software developer staff 1

I think a lot of it is about educating prescribers, getting learnt behaviour so they know what to do. There is some element that GPs don't like to be told what to do, especially by a bit of software, they know best. And very more so in the smaller practices, your older GPs, they don't want to be told what to do, they've been doing this for 40 years, they know what's best. Often, they're the worst prescribers. However, but in some ways, because it's not directed at them as a person but as an organisation, they realise that it's not picking on them.

<u>CCG Staff</u>

CCG Pharmacist 2

I: As an aside, do the GPs know the difference between an (GP CLINICAL SYSTEM) message and an (the CDS) message?

R: Probably not. I think we've got a mixed bag, because if I get any queries, which are actually very few and far between, they're saying, this is coming up on my screen and is it from Optimise? And I often have to say, can you send me a screenshot, and sometimes it isn't, it's an (GP CLINICAL SYSTEM) message that's already embedded in there. I can't think of an example off the top of my head because it's not really happened a lot. But yeah, I do think because they have all sorts of systems embedded in their clinical system, things to remind them about QOF, and they might have their own internal messages to say, when "Mrs Brown" comes in, make sure we ask about x, y and z, and it pops up. And that's something they've put on, so you can imagine, you can kind of switch off to it if you have too much.

CCG Pharmacist 2

R: It's short of shaped by what happens in your region as well, like if you've had local incidents, and then on top of that near miss, somebody, I think, in the region, had a patient complain that they'd had a miscarriage using Ramipril for example, and they were like, no one ever told me it could have been a problem. So, then it builds this picture of actually, we should be seen to be doing something about this, even if it means an alert added in, just to alert the prescriber at the point of prescribing. But yeah, I think there's an issue with medicines used in pregnancy, because the only time the clinical system would flag up that as an issue, is once a patient has been recorded as being pregnant, then it will come up with, hang on a minute, this is contraindicated in pregnancy, but it's the before that, isn't it, that you're having to warn people before they get to that point, and the clinical systems don't do that, you have to use your own brain and knowledge at the point of prescribing.

CCG Pharmacist 2

R: I don't know, the way I see it is probably like a bit of a nudge. Like, are you sure you want to do that, or, are you sure that's the right? Or, do you want to do this instead? You know, like in terms of switching to a more cost effective option. I guess it's sort of an addition onto your brain, to help you be more cost effective, but also making it more safe. So I guess it completely ticks that massive box of QIPP doesn't it, 'cause it sort of deals with the quality, it's sort of improvement innovation, but it's productivity and performance, like that whole, yeah, all of that is done by that.

<u>GP Staff</u>

GP Nurse 1

R: To be fair, I don't get many alerts, it might be to do with my prescribing because even though we have the FE300 we all prescribe within our competence so I'm not sure. But it's usually the usual drugs for chronic disease and the drugs for pain relief and that sort of thing. So there doesn't seem to be a lot of problems but I think it's because the education that we have I wouldn't prescribe something in the first place because I would look at what the patient already has. So before I prescribe I'll look at have they had it before, what are they currently on, and then I'll look at renal function et cetera.

GP Nurse 1

I: So going into the GP, so when you come into a practice like this you go into the GP system and use the clinical record from the GP?

R: I will normally use the community module but like, for example, today I've been to a care home and there's a few repeat items they were asking about, and so I'll go into the GP module and then look at the prescribing history. And then if, for example, they'll say there's not enough medication to last the 28 days I will go into that repeat template and change it from there, from the GP module, rather than do it from the community module because it gets confusing. So I have to work with both and so when it comes to...when I do an acute medication, like a chest

infection, I prescribe antibiotics, I'll use my community module because that's what other people working for the community trust would do. So I have to try and work out which is the best way of doing it for communication.

GP Nurse 1
R: Yes, exactly, I wouldn't prescribe them, I don't describe them because most of my population can't have them so they get an NSAID gel, something against your skin, and then some of the anticoagulants and stuff I have to be careful, if they're COPD then I would consult the GP because I know there's interactions and contraindications.

I: And those alerts should be coming up as well.

R: Yes, they will come up but I probably won't see them because I'm already thinking it because of the way of how my role is.

GP Nurse 2
R: So I'm not totally ruled by the cost at all but if I can do, I will comply to guidelines but not totally, particularly in diabetes because I know through experience that some drugs work better than others. So I won't just follow the formulary completely.

GP Nurse 2
I: Yes, right, precisely. Do you think (the CDS) helps with that or do you think it sometimes needs overriding?

R: I think it helps but I still think you need to know. You can't rely on it completely, you still need to know your formulary, definitely.

GP Nurse 3
I: Yeah. One of the safety indicators that is within (the CDS) that is one of the PINCER ones is the prescribing of a non-steroidal to a patient over 65 without PPI, or without some form of gastric protection, and that's of particular interest to some people. Is that a message you see…

R: Yeah.

I: …frequently?

R: Reasonably, because I deal with a lot of chest, so I use Prednisolone.

I: Right.

R: So yeah, I do see that one quite often.

I: I suppose with any elderly patient, anyone over 65, the chances of even giving them NSAID is going to be fairly high anyway.

R: Yes. It is.

I: Because of various aches and pains and stuff. What do you…the message is just that it…that if you are going to…I can't remember how the message is worded but it's basically, if you're going to prescribe an NSAID for someone over 65, consider gastric protection.

R: Consider. Yes.

I: But what do you do…

R: …with that particular message?

I: Yes.

R: Depending on what I was giving it for and the length of treatment?

I: Right.

R: I maybe giving it to somebody with, say COPD, who's going to be taking them for five days.

I: Right.

R: So, it may not be appropriate but then you've got to take it on an individual basis as well.

I: Right. Yes.

R: But then on the other hand, if I was going to prescribe it to something else that was more long term, then yes, I would. I would give it. I would add it.

I: So, you'd add the…

R: I would add it. Yes.

I: You'd add the PPI…

GP Nurse 3

I: Yes. And so, it's…yeah. And as you say it might depend on the…

R: It may depend, yes. Because with the COPD patient, I would ask them, have they had it before? And if they said yes, I would say, have you had any trouble with it? Did it upset your tummy? If they say no…

I: Yes. Yes. Then you've got…

R: …then I would be happy to leave them for those five days without.

I: Yes. But that's where you're…

R: So that's the decision support going on isn't it there?

I: Yes, because you're not just saying I'll do that?

R: No.

I: You're making an…

R: …uh huh, an informed decision.

GP Pharmacist 1 and GP10

R: Yes, see, I think it's probably the ones that are…because those are the scary ones that we're on top of.

R2: That's right, yes, so we know the obvious.

R: It's the less…it's…

R2: So, I think we're aware of the obvious ones. It's the more subtle ones where there's a joint effect like…

R: Well, it's the diuretics [voices overlapping 38:15].

R2: I was just going to say, diuretics, but, yes.

R: Yes, it's the [inaudible 38:17] drugs, isn't it, the diuretics and the ACEs and the metformins and the non-steroidals, like many combinations of those. Even just flagging up patients whose renal function has gone below a certain level whereby you should actually be looking at all the medication thinking, actually, is this still okay? […] That sort of thing would be brilliant but we're not just there yet, I don't think. So in that respect, I think there's quite a lot of scope but then I suppose you don't want it popping up for every single patient because if you include too much stuff then you have complete overload.

GP Pharmacist 1 and GP10

I: I mean to some extent, then, you are overriding [voices overlapping 04:40].

R2: I'm overriding it. Yes, I am.

I: Yes, so what…on what [inaudible 04:44] very challenging, on what grounds, but for what reasons would you override, then?

R2: They're a bit varied as to…you know, it is individual. I'm trying to think of the last occasion.

R: Well, the one yesterday, actually, I overrode it with the beta blocker. So, a beta blocker was being considered for this patient. They had tried it and not tolerated it. There was still a conversation going on in the notes about trying it again but was not considered appropriate at this time but obviously whatever the search that (the CDS) does hasn't been able to pick that up. So, in this case, I'm ignoring it because I'm going, this is not appropriate.

GP1

I: Right. Is that on everything? Is that on the best practice and on the cost-saving messages?

R: As far as I'm aware it is, yeah. This is the problem with this, they blend in, you don't really realise what you're doing half the time.

GP2

You have quite a few, for example, if you prescribe something and there was an interaction between some other tablet, that's good. Yes. Then you see on probability if it is going to benefit patient or not. Basic things. Somebody who has asthma and or problems with their heart, they would benefit from the beta blocker and beta blocker can trigger it. So, it will come out in an alert. You know, beta blocker...this patient has asthma, are you sure? Are you sure that you're going to prescribe? But they already had before beta blocker and you're titrating, and the cardiologists and they didn't have any reaction, but the system doesn't know. The system is too persistent. It only do what it's been told to do. […] So, the system doesn't know that...okay, this patient has been on bisoprolol without any...1.25 or 2.5 and it's going to 3.5 and you're going there...are you sure, are you sure? Red alert is coming out. It can be quite embarrassing in front of the patients and you need to explain to the patient, yes, it's coming here but

you're taking already that tablets which isn't causing you harm which you will benefit. You need to take it because it's already reduces your chance of heart attack. So, does that make sense?

GP2
R: It depends from which... Experienced GP, they know what they're prescribing and how they want to prescribe and the reaction of the medications and you're dealing with a certain number of tablets which you are familiar with. So, your prescribing habits changes very little. You know, it does change but it's not as much. You know, your first line, second line, third line...you have your boxes in your head. However, with the younger generation, with inexperienced GP...you know, younger doctors, I think it's quite useful.

I: Yes. So, it seems as though there is an experienced part to what you usually say, you're…

R: Yes, but for example if somebody's on amlodipine you will automatically change the simvastatin to 20 milligrams or change the dose. It shouldn't normally cross...across mind of my junior colleagues because it's not anymore...it was pertinent maybe five, six, seven years ago but it's not anymore in our eyes. For us it's like second nature. Oh, interesting, that might be the problem...change. And similar things to, you know...

GP3
R: And now I've planned I go for what I think…I've got sort of used to how often it's going to come up and I've made a decision perhaps to go for one or two of the loose hanging first. I'll go for the really important ones.

I: As you say, if you've got to change 12 medications for a patient the patient is going to look from their point of view, do they want to have 12 medicines changed?

R: Another thing is, and I'm very careful about this, if there's been historical bad prescribing, to change that you've got to say to someone that wasn't right, and that's actually quite a difficult thing because you undermine either your own confidence or the confidence they have in the medical system. So it's a really thin line there sometimes to say what the bloody hell's that? I think my plan seems to be working not too bad. Again, it's one of those things that I'm not quite sure where we'll be in three months' time. It's like the Forth rail bridge, I'm painting it and going back to the start again.

GP3
R: And that's my concern with it. Certain things I'll just flick through in (the CDS)because it's not worth the effort or this potential argument or time that I'd have to spend with a patient to actually change things, and I make a judgement about that. But when it's a safety one it's really good, because you learn things. I pick up things and I think I didn't know that, and I think I'm fairly competent at interactions. And it'll flow past them. It'll be ones that you've perhaps not got time to just look at the 28 repeat medications as thoroughly as possible, and it will always pick up…one this week, the amlodipine and simvastatin that I might potentially missed if I hadn't just had a little reminder. So I like the safety reminders. They help me. The cost reminders don't.

GP3
I: So the cost reminders are more niggling than…??

R: Again, they are. I know the policy, if I decide that I'm not going to follow the guidelines, and they are guidelines, so be it, that's my responsibility. As a GP it's still fundamentally way under budget. I've shown that I'm a competent prescriber. So I do get niggled by it a bit, and there's certain ones that I understand why they say that they're not cost effective and things like that, but I'm sometimes not prescribing them for being cost effective, I'm prescribing them for overall health budget. So rather than refer someone or ask someone to come back I'm doing no harm is the way I'm looking at it, and I might be prescribing something that cost £2.16 rather than £2.05, but I'll live with that.

GP3
R: When it comes up it will give me a suggestion of what to do. It will give me some information that most of the time I've seen before, so I know the next step. So it's whether I agree with it or I do the "not suitable for this patient" or some other information down that side. In some ways I think my default in over 50 per cent is "not suitable for this patient" at the other place. Here my default would probably be to put the switch screen that says use original, and that's fine, I just click on the convert. There's one that I struggle with, and again it's my age, when you've got a default trade name. I struggle with this. Because in all my training in all my life it's been beaten out of me that I'm never to do this, and then suddenly tells me it's all right.

I: Because you normally default to generics.

R: Yeah, I have a generic default. It's a very strong default. So, for example, the amoxicillin to amoxo, it hurts. It doesn't feel right. And again it's an easy one and it makes no sense, it's no cost, but the worst thing I would ever get was a patient saying I don't want amoxicillin, I want amoxo. So I'm always just a little bit wary about those switches. I do do them. The creams and things tend to be the interesting ones because they tend to be quite seasonal, the changes. The one I can think of recently is the beclomethasone to betnovate, which again just feels a bit odd. But if I've got enough time I click on as many as I think is reasonable.

GP3
R: Yeah. Much that it would be good if we can minimise pop ups. But I think for the safety ones I don't mind that at all. That's fine. I never object to a good safety pop up. Even the nonsteroidals, that's still a good one, even though it should be engrained in us by now. Occasionally you get the weird one that comes up and you think that's really interesting, I didn't know that.

GP3
R: It doesn't annoy me that way, no. I don't get surprised. The only intuitive ones is when I know it's going to come up and I just wish it wouldn't, and I'm going to override you anyway.

GP3

I: Yes, right, okay. That's really interesting. You said here you've done a lot of previous work around prescribing. What sort of things have you done then?

R: Many moons ago I was the prescribing lead in a big bit of the city, and I did a lot of work on red drugs, the hospital only prescribed ones, the black ones, and I used to share this with all the GPs. We used to have a peer support group almost for prescribing, and the only way that (name of practice) was able to interact there, because I had to lead by example, so I had to be at the top of the table for everything. So, for example, the nonsteroidals, the diclofenac in particular, we had to be at the top, because I couldn't tell other people to do it if I wasn't doing it myself. So we had a real sea change in mentality about these things, and then we had league tables published, so we had comparative…

I: Within practice or across practice?

R: Within practice, but even within doctors. Not quite so easy to do with doctors because the repeat prescribing distorts it, but for the acute stuff we could totally do it.

I: You would work out who's prescribing what.

R: Yeah. And if we got to certain numbers we could almost name and shame. It's a bit of a cruel one to do. But also I do it in a nice way. So I think we did that for five years to about two years ago. We made a real effort. We've always been inside our indicative drug budget, which again is a bit of a weird one as well because we've got a tiny little drug budget. It doesn't match our population, but we've always been inside it, and I think we are just traditionally good prescribers in a good place here. I think the other practices have got more churn, have got more staff turnover. Stability I think creates much improved prescribing.

GP3

R: But I'll be honest and say the pharmacists in the past, the medicines management pharmacists, they're so QIPP and cost savings obsessed. Not even cost savings, I'd say demonstrable, identifiable cost saving obsessed, that they sometimes are counterproductive, and they're not as safety led as we are. Among other things we have regular significant events meetings about either drug errors or drug problems or drug interactions, and myself and Doctor [name 0:08:48] used to be the prescriber on JPAG many years ago, and we'd almost have them on a daily basis. That's what we do. We were just pharmacology nerds.

GP4

R: No, I do, yeah, I override things. Either because…but usually it comes on that tiering again, it's how important do I think it is. So to some extent I'm just using my judgment about that and then experience, so it's not always evidence-based, it may be they're just a personal prejudice about…prescribing habit comes in possibly and I think, well, yeah, fair enough, that's a risk that I take. That's a decision I make with a patient and I'm on this occasion not going to take notice of that because I think the indication outweighs the risk that you've highlighted, so… And I think if that was happening a lot, I think if that was constantly jarring and it was telling me to do things that I was finding irritating, then I would say so but I don't really get the impression it is hugely and when it does I close it down.

GP4

R: Well, a good example might be particularly palliative care. And so you now get an elderly chap with prostate cancer that's spread to his bone and you know that an anti-inflammatory is probably going to be a good option in terms of the thing that might be likely to help, but they've got impaired renal function, they're 82 and all right, you give them the PPI but, you know, it's one of those ones where…and I might for example use Diclofenac in that case because I've found that Diclofenac, I'm less worried about cardiovascular risk perhaps in that patient, and I find that [inaudible 20:39] seems to affect a…tolerated from the GI side of things so I make a non-formulary decision based on all of those factors. And I think, it's an end of life or palliative care situation, so I suspend some of the cost concerns or some of the safety issues potentially that would be relevant for somebody younger or fitter or in different circumstances.

GP5

R1: Okay? Because they haven't been through five years of medical school, they haven't been through six years of training on the wards. They haven't had that 11 years of experience of what safety is about, and suddenly they're given the golden pen, after they've done some kind of prescribing course. […] Well, there's your risk then, isn't it? But then, you can't turn around and say the risk is to do with the IT. The risk is to do with the fact that we've got a different group of clinical staff, and someone has now decided we're going to let you, we're not going to put you through medical school training, but we're going to give you the same equivalent power to be able to prescribe as someone who has done […] The lies the risk. And that's a wider one for everybody. The defence organisations are worried sick, because they think there's going to be a big increase in complaints.

I: Because of that broadening out of prescribing?

R1: Yeah […] Until we find out what's safe prescribing and what isn't. And it's possible that (the CDS)could potentially provide that. But then, you could turn round and say, but this is a system that is more designed for someone who hasn't been through medical school, hasn't had six years of training et cetera, et cetera, et cetera.

GP6

R: It probably is helping because as I said, you know, even though I said, I don't think I do pay attention, I think that probably the odd one where it's…because if there is a whole pile of them, if the first thing that flashes up is about the EGFR, it will make me think about checking the EGFR. If you come in with your Naproxen it might actually help me to remember to give you stomach protection. So if there is a something that pops up, I suppose there is a higher chance of me looking it up but I'm just aware of the fact that if there is loads of red mess, almost my default mechanism is to go, oh pile of shit, you know and ignore it which actually is probably the very worst thing I could be doing. But I think you also go off your own...you have an in-built sensor don't you as well and I've been doing this job for a long time and you're thinking, yes but it'll be alright

because I'm only going for a low dose or something like that or I'll stop something else. I'm really not sure that...I know I have never sat there and laboriously gone through all the alerts.

GP6
And I think the problem is, that I've been a GP for a really long time so I've actually got a vague idea, in terms of what might constitute a side effect from something or other but it's actually.

GP6
The problem is if it's digging out old conditions and old medications and that actually is one of the...I've just suddenly thought, that is one of the things it's shit at. It can say, this is going to interact with Cephalexin which they had on an acute prescription six months ago.
I: Right yes.
R: And that is the sort of thing that pisses you off because you're just, like, well, you know, what a load of rubbish is that, they haven't had that for six months, obviously it's not going to interact with it. Can, so can you see what I mean, if things like that are occurring it makes you just ignore it.

GP6
R: Well I don't know, maybe to me but I don't know what the system think is important. For instance, there is a huge drive in our CCG about prescribing Trimethoprim.
I: Right.
R: Okay, so this is the first thing that pops up.
I: Three.
R: This is an old lady, so what I'm saying is, that there are more than one page but I don't think I've ever done that to get to the bottom of the page. So I've never finished scrolling that down to see what the bottom says. And this...so this is the thing I was saying to you about what it says about what if her renal function is this, this, this or this so it gives me a variety of options and it talks about caution in the elderly. What it doesn't say at any point is, this antibiotic should not be prescribed for UTI's because resistance is really high and in (name of place) there is 60 per cent resistance, refer to guidelines. First line should be Nitrofurantoin. That is what it should say, but it hasn't got any of that on there. It's got all these alerts and this isn't a blood dyscrasia, you know, I'm really glad that I had that lady here and I've been able to show you that. Because that's the kind of thing that I mean and to be truthful I would just override that.

GP 11
R: Yes, I think more on the… for me I think for me there's a distinction between safety ones and cost-effective ones and I think it's useful to know the cost-effective ones and knowing when something is ill advisable from an NHS point of view. But I think this is something that the different members of the GP principals would vary on. And I think even out of the cost effectiveness ones a couple of the partnership would follow those relatively absolutely and then I think a couple of others, probably myself included, would be more inclined to say - if it's clinically effective then, fine, and have a lower threshold to override.

I: Yes. So, you would be overriding some of this, but on the safety, you would be more prepared to follow the safety ones?

R: Yes, very much so.

GP 11
I: But from what you've described, do things do then fall through the first net and are captured on the second net?

R: I think they do. That's how I think of it in my head anyway because I think it's so easy to override (the CDS)for correct or incorrect reasons and different people will have different things on it. So, it's useful information to have but it doesn't tie you and there's good and bad about that. So, I think having something else that then says, alright well here's the audit outcomes of how are you prescribing for good or bad? Well, for bad, isn't it? So, let's have a look at it.

GP 11
I: One of the things that I've sort of recently been asking people is, do you think it has improved care and do you think it has the potential to continue to improve care?

R: It's definitely a way of disseminating, I guess, CCG prescription decisions and evidence. So, I think that's the biggest, quickest thing I can think of because probably most people don't read the new formulary guidance or APC or whatever. So, yes, I've certainly come across anecdotal things, either myself or with other people, where safety things where...well, I was going to do this but there's this alert, let's rethink of that. I mean, the cost effectiveness is a tricky one. But you could then argue, improvement of care across a population level, how do we best use the resources et cetera?

I: Yes, precisely. Because if you're going to save money here, you can put resources into this group of people here.

R: Yes. And I think if you didn't do that alert, everyone would always continue to do the same thing that they did having not read the emails before. So, yes, I think it's a really efficient way to tell people of the changes in that pathway.

GP 11
I: When you decide not to do something, you have to give a reason in the box, do you?

R: Yes.

I: What do you tend to sort of write in that?

R: Yes. This is probably the bit where I don't do it accurately to be honest. It's normally just the one where you don't have to write anything purely because of speed, I think, which is probably unhelpful for the system and is more a case of, this is a quick way to override it once I've decided.

I: I think some of the (the CDS) sort of has got a drop-down, haven't they, where you can drop-down as to why, I think.

R: As, is it sort of 'not suitable for this patient' and all that sort of thing.

I: Yes, not suitable for this patient and so on.

R: 'Disagree with rule' and that sort of thing. […] Yes, I'm struggling to think of specifics. I've got a feeling I sort of override without typing things.

GP13

R1: Yeah, generally…is it just general practice, I think GPs tend to feel that we're better than anybody else, 'cause we manage without immediate test results and things, but general practice is about managing risk, it's all about managing risk. It's often, I'm no gambler, but I'm interested in the mathematics of poker and probability although, I don't play and I don't win when I do, but it's a very similar process, it's, you know, looking at ranges of possible outcomes, and what are the likelihood of them, and how can I shift those probabilities to be in favour? It's not about, do this, and this will happen. And some of it is, there's an adage, I don't know how true it is, that a good protocol will manage 80 percent of the patients that go through it, and the other 20 percent need a doctor. So, the pragmatism comes from the doctors.

GP13

R1: And, I think, we had a meeting yesterday about skill mix and what have you, and that's probably one of the issues in general practice, is that we all try and do everything, whereas in fact, what we're trained to do as doctors, is to do the difficult stuff, being pragmatic, the balancing two, three, four, competing risks, and working out what to do despite them, and how to manage them, and how to safety net them and all that sort of stuff, and (the CDS) definitely helps from that, because anything that helps remind you of what the risks are, helps you manage them better. And so, what might have been, 15 years ago, a prescribing error, you could do exactly the same thing, but at least you're doing it as a consciously managed risk now, rather than as a prescribing error.

I: Yeah, rather than just simple, oh you shouldn't have done that, it's a, well there's a decision process towards that, and it's around…?

R1: I can give you an example. So, we recently had a woman in a nursing home in AF, who 15 years ago, would never have been anticoagulated, because she sits in a chair in a nursing home, and is hoisted and stuff like that. But, because of decision support, not just Optimise, but various methods of decision support, and [inaudible 00:12:36] she was put on rivaroxaban for her AF, and it was managed appropriately, and then she fell, and she's recently died of an intracerebral haemorrhage. But I think 15 years ago, we would have been thinking, oh blimey, what did we do there, was that the wrong thing to do? Or, if she'd had a stroke and not had it, we would have gone, oh there we go, that's the… Whereas now we can go, actually, that was still the right management, she wasn't falling, she had AF, so whilst she's had a bad outcome, but actually she's been on rivaroxaban five years, and she might have had a stroke three years ago, and been disabled. So yeah, so that's an example of how, 15 years ago, it could have just been not even noticed as poor prescribing […] Whereas now it's a managed risk, which happens for that individual, sadly, to have gone wrong, but you can look back and say, actually, that was still the right thing to do, 'cause that's a…

I: Yeah, rather than simply, we don't know what we did, because the system has been able to track that through as it were.

R1: Yeah, and the role of software like Optimise, is that is reminds you of that, and gives you the confidence to do it. So, when you're seeing that woman about cellulitis, which she had a lot of in the last year of her life, you know, whenever we went it would have said, had she not been on anticoagulants, remember she's got AF and she should be anticoagulated, and she's fallen 300 times a year, which she wasn't, so yeah.

I: Well, that's interesting isn't it, 'cause I mean, that sort of brings us back to what I was saying before, or what you were saying before, in terms of, that you're using that skill, knowledge, expertise of yours, as a doctor, as a GP, do you rely on these things too much, what's the balance, if you like, between your expertise and the systems?

R1: It's like a self-driving car isn't it, who's fault is it if it crashes? […] I mean, I think as GPs, we know, or should know, that if we sign the prescription, then it's our responsibility. But, I don't know, I think if you had a semi covert observation of my practice, I know that if I get an (the CDS) alert, I'll read it, I think, a 100 percent of the time, I think 100 percent of the time. I'll dismiss it without any further thought, 20 or 30 percent of the time, and then I'll give it some proper thought the rest of the time, sometimes ending up in action. But, I think what I don't know, is how many prescribing decisions I make, where (the CDS) doesn't flash up, when had it been programme differently, it would.

I: Well yes, if there are other messages there, yeah.

R1: And then I'm thinking, and it will be subconscious, because when it's not there I don't think about it. I was slightly anxious about, I didn't know what we'd have to talk about today, 'cause (the CDS) is in my consciousness when it's on the screen, and then it disappears, which it wasn't when it was too slow, but now it is, it's just there or not there. […] So, I think so it's the other side of the argument then, how do you capture, you could easily observe my day, and work out how (the CDS) affects me when it's there, but it will be much harder for me to see how it affects me when it doesn't fire. Yeah, that would be harder, and whether I am subconsciously thinking, this is okay, 'cause nothing's warning me it's not […] I've at least one example of that. We had an elderly lady in a residential home, who was on 40mgs citalopram for her anxiety, and I think a medical student, or our pharmacist did an audit, or something came from the MHRA, I can't remember, anyhow it flagged up, we ended up doing a search. And it transpired that I had a knowledge gap, and I wasn't the only one, that 20mgs was the maximum dose you should be on, if you're over a certain age of citalopram. Now, as far as I know, (the CDS) had never told me that, and (GP CLINICAL SYSTEM) had definitely never told me that,

because I went in and tried it. And she'd been having that for some time, without any, and I'd been signing her repeat prescription. So, I guess my behaviour had been, there is no warning, it must be okay.

GP13
I: Which of those two approaches is more valuable, and do you need one or the other, or are they complimentary? Sorry, that's three questions in one.

R1: No, no, 'cause the questions work through the thought processes as I was having them, I think you need them both, because if you don't have the PINCER type thing, the retrospective look back at how it did, then that's the equivalent at taking the windows out of a Google car. It's putting complete faith in the (the CDS), and saying, we don't even need to find out how good it is […] And also, there's the danger that there is the possibility that, if subconsciously our prescribing risk management, if we subcontract that risk to (the CDS) subconsciously, and don't know we're doing it, then actually our PINCER stuff could get worse, so you need to know. I think anybody signing a prescription who understands what their signature actually means on that prescription, would always want to look back as well as forward.

I: Because you want to know might happen as a consequence, is it taking that responsibility for signing that prescription?

R1: Yeah, I think that's…at some point in a doctor's career, you realise how important the squiggle is on the bit of paper, or whatever equivalent it is, it's a pin number into a thing now, on a smart card, but medical students don't understand it. I used to work at the university, and I overheard a foundation year doctor telling a medical student it doesn't matter what you write on the prescription, 'cause the pharmacist will come round and correct it if you get it wrong. And that's the only time I've ever really bolllocked a junior doctor. I normally have quite a supportive, inclusive approach to education, but that doctor understood the value of their signature at that point, and what their signature actually meant. And I think you do, at some point in your career as a doctor, you understand that your signature is actually, when you make that mark, what you're saying is, the rest of my career says that this is okay. And it's that level of responsibility. And not just the rest of your career, that's a very selfish way to put it, but this patient's life says it's okay, or, this patient's health says it's okay. And I don't think, in my lifetime, artificial intelligence is going to be, that entire responsibility is going to be abdicated to artificial intelligence of any sort, because even if the artificial intelligence gets good enough, I'm too old to change the idea that my signature means that much. So, I think, if tomorrow, artificial intelligence that makes better prescribing decisions than me, comes along, then I'll use it, but every prescription I'll sign, will still be my responsibility. Maybe the next generation of doctors, growing up in that environment, won't feel that.

I: Yeah, because they've got the…

R1: But then I'm not sure why they would call themselves a doctor. That is what being a doctor is really, it's about…

I: Taking that responsibility for…

R1: It's about balancing various risks, and taking the responsibility to say, that's okay. If a computer can do that, we can shut the medical schools then.

GP13
R1: But it's balance with that not really knowing how it affects the whole of my decisions, so I'm aware, because of the signature on the prescription being the responsible thing, I'd like to know how it affects me subconsciously, when it doesn't flash up or…

I: Is it making your more cautious or less cautious when it doesn't flash up?

R1: Well, I don't know. Both are possible. […] And more cautious or less cautious when it does. So maybe, if it suggests that I should be prescribing something else, and I go, oh you need something else, and actually it's something that in 2008 they had and were allergic to, it could make you more trigger happy and less cautious, 'cause a certain amount of, it depends how much you trust it.

<u>**Sub-theme 4b iv Prescriber decisions and decision-making processes**</u>

<u>**Software developer staff**</u>
Software developer staff 1
R: Yeah. So my background is, before coming to First Databank, was working for another decision support company used within practices. That was very different in that it was an executable file added onto clinical systems and it couldn't integrate with patient records. And that's when I first heard about (the CDS) was obviously working for somebody else, and then we learn about, oh wow, it can pull out data such as age and sex, and look at blood pressure results and things like that. So it did seem, you know, quite impressive.

Software developer staff 1
R: One of the best descriptions of (the CDS) it's like having a medicines management pharmacist or technician sitting by your side, looking at your choice of medicine, going actually for this patient, you might not want to choose that; or what you're picking there isn't on our formulary; or hang on a minute, that's red; or whoa, look at their, you know, renal function. GPs have to be…they have to be a master at everything but they can't remember everything. Guidelines update on a regular basis. I think if you asked most GPs are they on top of all the new NICE tags, the new NICE clinical guidance, the new MHRA warnings. You know, if I think about, say, the (name of place) area, so I know that the GMMMG formulary has just been recently updated in December for the new COPD guidance. Now, would I expect every single GP to have…it would be nice if they all sit down and read those guidance and go, right, I exactly know which inhaler I'm meant to be using, what FEV the patient's meant to have before I initiate a steroid, when am I meant to use…we can't expect them to remember all of that, and that is when…

I: No, precisely.

R: Yeah, our software can pull all bits of information and at that point of prescribing, steer them in the right path to say actually, this is the type of inhaler, or, you know, if it's an asthma patient, a gentle reminder that yes, there may be a steroid on their prescription and a long active beta 2 agonist, but this asthmatic actually hasn't requested that steroid for 12 hours, but are always having their Salmeterol, that's, you know. It's more from the safety and best practice.

Software developer staff 1
I: For that particular patient?

R: For that particular patient. And people say…I think one of the biggest issues is on (GP CLINICAL SYSTEM), on repeat authorisation, it just slows it down. Because if they're on 12 items, we will check if there's a message against all, and they're like, I haven't got time for that. My counter argument is, so you would have just re-authorised it and not looked and not looked at anything, and sent them out the door, yet we've pinpointed there's a cost you could have done, there might be a dose reduction, there might be some monitoring that's required. We, in essence, do a lot of that review for them in that piece of software. That's saving them a job, because if they send that person out and you've not done a set of bloods, and we actually realise that their white cell, you know, count has dropped through the floor or whatever, because they didn't even think about it, and we've brought that to their attention, that may have prevented something happening in the future.

Software developer staff 3
I: Oh absolutely, that seems to fit in very nicely with quite a lot of the things we've been seeing on the ground. I mean, one of the things I just made a quick note here to say, is, we've found that there is, in a very limited sample of interviews so far, that there might be some distinctions being made around acute prescribing to prescribing for the more longterm conditions.

R: Aha, yeah.

I: And what do you think, do you think that's something that probably is, I mean, sometimes, it could be that it's more valuable for acute, or it's more value for LTCs, but it does seem there might be some sort of distinction around there. I wonder what your thoughts on that are?

R: I mean, that's a great point. So, acute prescribing, generally is done quite well, through (the CDS) we think. As in it feels like it's something that makes sense to a GP. Someone has come in with an acute problem, they need this drug for this length of time, and (the CDS)helps me do that […] The problem is with LTCs, are one, patients can have multiple LTCs. When they come into a consultation, it's really unclear, until you talk to the patient, why the reason they're there. And it may be a completely different problem, and actually that's not related to their longterm management. So, I guess, we see the role of GPs as changing. So, it's almost like there's a stratification. There's the kind of easy, quick and dirty acute stuff, which they're always going to have in their inbox, like they're always going to have those types of patients. Those patients are starting to also be seen by non-medical prescribers. Then the more longterm condition management, but the standard longterm, I'm a person with diabetes, whose HbA1C is running at 7 to 8, I'm taking me meds, I do exercise, and I watch my diet. So, I just need an annual check-up, and if I have any issues, I don't necessarily need to go and see a doctor, I could see a pharmacist, or I could see a nurse practitioner. So, for those kind of patients, actually, a point of care tool like (the CDS)isn't very useful, because you're already going to be on the medications you should be on hopefully. So you'll find that we're probably not offering too much then.

Software developer staff 3
R: Because now you're able to kind of capture patients where it may be appropriate to consider whether they should be started on a new treatment, which we can't do in Optimise, you can't do, 'cause the trigger is a medication being prescribed, you can't advice that there's a patient who has been diet controlled for their diabetes, but actually their HbA1C has gone up, so we should consider starting them on some sort of oral hypoglycaemic agent. But, with an audit tool you can start doing that. You can start identifying opportunities for new intervention. You can also start thinking about opportunities for escalation of therapy. So, those are definitely areas where we're looking at, and you can start doing mass bulk changes, or you can start understanding who needs what test. And that kind of management of patients, we think is going to be take up more and more by non-medical staff.

I: By other people than GPs, yeah, indeed.

R: Exactly, and that kind of leaves the GPs, to deal with the more complex patients.

I: Yeah, acute diagnosis. The reason why, that example you gave with the diabetes, just checking back there, the reason why that wouldn't then necessarily be useful with Optimise, is because the diagnosis isn't already in there, or the change isn't already in there, so you have nothing to support that decision making, is that right, have I got that right?

R: No, I think, well, it's more likely that you do have the information in there, but if you think about what it means to, with the ority of those patients, not much is going to change. And I guess you're right, it's when things do change, are those elements put into the system?

I: Yeah, precisely.

R: But even then, unless you're making a prescribing change, or it's a new drug being prescribed, that information isn't going to be taken account of.

<u>CCG Staff</u>
CCG Pharmacy Technician 1
But the feedback was generally people welcomed the greater functionality of (the CDS), and it did seem to overcome some of the deficiencies in the Script Switch system where Script Switch couldn't actually look into the individual patient's clinical records, and so we found instances where medication was being recommended on the basis of a cost efficiency, but it was actually recorded as an adverse drug reaction in some people's notes, or intolerance.

CCG Pharmacist 1
So there was a recognition really that one of the or deficiencies around the Script Switch system is that they could not give bespoke recommendations. It gave general recommendations, and if that happened to conflict something there was almost an expectation that okay, we've changed from statin A to statin B, we don't know if that's contraindicated, but we expect you to go away and double check that before you expect…whereas the GPs did not know that initially it couldn't look inside the clinical records and they felt that if it was giving a recommendation it had done all the necessary background checks. So it did cause some consternation around that particular aspect, and I think that was one of the reasons that we ultimately looked to decommission Script Switch in favour of a more sophisticated system, even if that was slightly slower. Something that could actually interrogate the individual patient's clinical record.

CCG Pharmacist 2
Plus, we had more guarantees that things were triggered to more of an individual status, whereas the original software we had, the messages that we put on, whether be it cost, quality, whatever, it would flag all the time, so it wouldn't be individualised to a person. And we found a lot of GPs and clinicians would get pop-up fatigue; they'd get so fed up of it all. So, you slowly saw the acceptance rate declining, so that was another thing. They said that this is more individualised. It would only flag for specific criteria, so we thought, well, that's better.

CCG Pharmacist 2
R: Exactly, and then they'll only pop up if specific things trigger them from the practice end. So, if, like, someone's read coded with a certain condition, for example, or if someone's already tried... Say, it was a cost-saving, if someone's already tried that, what we're asking, it wouldn't flag again, because they've already had that conversation, they've already done it and we don't want to keep mithering the patient all the time. There might be a valid reason for them not. Say, it was a cream, maybe there's something in that cream that they particularly had a reaction to, for example. So, yeah, I suppose a lot of it, we depend on what is from the practice end, what's read coded, and in terms of conditions. And also the medication side, if it's a trigger against an actual drug, that's a bit easier, because if they've had it before, it won't trigger. So, I think it just depends on what's on their system.

CCG Pharmacist 2
R: Yeah, and I think that's one thing, that when they were sort of selling it to us, the prescribing thing, was, that it is quite an intuitive, intelligent system, that you won't be getting that alert come up for every single patient who's on that drug, it's when they trigger, with, for example, poor renal function, and this matches and this matches and this matches, then it will come up.

CCG Pharmacist 2
R: And if there are tweaks we need, we'll feed that back to them or whatever. But I think it's really good that it's intuitive, in the fact that it will only flag, for example, with the trimethoprim one, it should only flag for patients who are over 70.

I: Yes, it's tailored towards the individual patient.

R: Yeah, which I think is really a good thing, that that's the case. Because then, the way that prescribers then hopefully will see it, is that it's important enough for it to have come up, because it is tailored to that patient.

CCG Pharmacist 5
I: I mean your acceptance rates you talked about there are, I think, pretty much average acceptance rates that people use and not just (the CDS)[inaudible 30:10], other things as well, but in some respects, it still means that four out of five or it means four out of six messages are rejected by… Why do you think that is?

R: I think sometimes it's, I haven't got the patient in front of me. I physically can't do anything about that so I need the patient there to talk to them. They might not have time to deal with it then and there. Some of them, they may well have accepted it in their head but don't want to do it now so they'll reject it or close it but do it anyway. It also fires for test patients so some of this could be people training or looking things up. It might be our team doing switches and because we're recording it elsewhere…

<u>GP Staff</u>
GP Nurse 2
I: What, sort of, confidence and trust do you have in Optimise?

R: Yes, quite a lot really. I don't think any one tool you should totally go for and [voices overlapping 26:10] not one thing because, yes, you shouldn't rely on just one thing at all. You are still responsible for prescribing and what you prescribe so you've still got to justify it. You can overrule and then we can take off. I mean that can be an absolute nightmare if you've got people with previous allergies and sensitivities, trying to get beyond that, but is that a bad thing? That's a good thing, really, isn't it?

GP Nurse 2
R: So I mean initially, although I wasn't prescribing, sometimes I would generate prescriptions but certainly once I'd started prescribing then you would see the alerts and the different options. That's when I realised it, really, and I find it quite helpful, yes. I, as a new prescriber, definitely find it helpful because it gives you the options and more and the cost and different… Then I don't want to get confused with like…because our CCG also put flash-ups on about certain drugs and obviously there's the formulary as well. So we've got that many things we're looking at and I know some can actually be turned off, can't they, on the prescriber?

GP Nurse 2

I: Right, okay, how much do you find you are overruling things, then, or not accepting alerts?
R: Yes, quite a lot, really, I think. Just because the (name of CCG) meds management that our actual formulary is linked to I don't always agree with or it's not quite up-to-date as it should be.
I: Right, okay, how are you coming to that decision, then? Is that based on what you know?

R: Yes, it's based on, sort of, clinical decisions of like, you know, prescribing it. So, there's one, a particular one that's on formulary which is just the DSN's, the diabetic specialist team at the hospital, they don't use that one either but [voices overlap 07:23] still the top one on formulary so it will always try and veer you towards prescribing that one.
I: Right.
R: We just overrule it.
I: Is it because the formulary is behind other…you know, it hasn't been updated to other advices coming from the specialists or from…?
R: It's not always updated [inaudible 07:47] but also, it's sometimes just that the drug of choice which is slightly cheaper is probably not very effective.
I: Right, that's interesting.
R: [Inaudible 07:59] which we just ignore and I know all the diabetic team, the one that's on formulary for that particular injection, we don't prescribe that one anymore.

GP Nurse 2
I: Do you use it for doing medicine reviews of some of these patients as well? Do you go through all their medicines?
R: Sometimes I do med reviews and it'll come up, particularly the ones that we're encouraging patients to buy over the counter. I do find that quite helpful, yes.
I: In what sort of ways?
R: So, it's quite a good way of explaining to the patient that actually we can't prescribe you that anymore because that's something that you need to be getting over the counter.

GP Nurse 2
I: Yes, I mean last time we talked about your confidence and trust in (the CDS) I mean you said you had quite a lot of confidence in it. Do you still feel that or do you think it's still a good system to be using?
R: Yes, I do because I think anything that helps or cuts down the risk… So I would never just use it on its own.
I: Yes, that's interesting. Do you feel it's part of other…?
R: Definitely.
I: Yes.
R: Yes, definitely, so I would always use it alongside other stuff. Say I was prescribing an antibiotic which I've not prescribed for a long time, actually, when I go on it, it tells me the ages and the doses but I'd still check it with the BNF.

GP Pharmacist 1 and GP10
I: Is that because holding all that stuff in your head is…it's e…
R2: Impossible.
R1: It's impossible…
R2: Impossible.
R1: …and becoming increasingly more impossible in my opinion. Maybe that's just 'cause I'm getting old.
R2: It's impossible.
R1: But yeah…
R2: It's truly impossible.
R1: …because it's endless and also changing constantly and I think there's so many more variables now, so we now have much more issues now than we did this time last year with stocks shortages and having to prescribe alternatives because of things were out of stock and then maybe prescribing things that we wouldn't normally prescribe, for example when the Ranitidine was out of stock, we're starting to prescribe Cimetidine which we've not prescribed for years. So yeah, and just the amount of information, advice, guidance out there is just mind-boggling.

GP Pharmacist 3
I: Right. So, you are in front of a patient, you go to prescribe something, something is popping up, what's the…if you like, talk me through the thought processes that are happening there then.
R: Okay. I will normally look at is the recommendation going to help with concordance of medication therapy.
I: Right.
R: I think the big one I've found is down to calcium tablets, where the recommendation is a one a day tablet, which is rather large, in actual physical tablet size, whereas the more frail patients cannot swallow the recommendation and would rather stick to what they've got.
I: Right.
R: That is one of the ones that really stick to my mind, when I'm doing medication reviews.
I: Yeah.
R: The other one that pops up for diabetes is metformin, and when I've got someone whose quality of life is really suffered with metformin side effects, gastrointestinal side effects, spontaneous diarrhoea, tummy ache, abdominal cramps, I'm trying to prescribe sustained release metformin and the system is telling me don't, or strongly recommend normal release metformin.

GP Pharmacist 3
R: If I put a weighting to it.
I: Yeah.
R: Patient, clinician, software.
I: Right.
R: In that order.
I: That's really interesting. I'm going to say why?
R: You are going to ask me why! There is no point having a cut for – in my perspective as a pharmacist I do see, my consultation is structured differently to a nurse and a GP.
I: Yes.

R: I have sat in with GPs and I've sat in with nurses and I've also had registrars and STs sitting with me and watch me work, and they've said to me, you structure your consultations differently, you converse with the patients in a different way. My weighting on having a successful consultation with good outcomes weighs on the patient being happy with the clinical reason they've been given.
I: Yeah.
R: Hence, the weighting is on the patient.
I: Is that something to do with the types of patients you are seeing? Because by large you are seeing people with long term conditions I presume.
R: Yeah. Correct. However, some patients like pain management, have to be told differently. So, then the weighting will tend to rest more towards me and the software.
I: Yeah.
R: If I'm trying to prescribe, let's say, Pregabalin, but the software says Gabapentin is clinically equivalent and safer and more cost effective, that will weigh more than what the patient is after but they will still get the same outcome, because they never knew whether they were going to get Pregabalin or Gabapentin.
I: Yeah.
R: But then that's a decision that me and the software have made together.

GP Pharmacist 3
R: Fairly, yeah. That's a fair assessment. They are coming in from a financial assessment, also with PINCER, safety always. In some cases, they don't consider the patient. Particularly not in this area, they are very very good. In other areas where I've seen blanket switches being made by medicine management, it's been a lot of kerfuffle going on.
I: Yeah.
R: And I've had to, as a practice pharmacist, then pick up on that and bring the patients in, have a chit chat, and tell them why the changes have been made, are you happy with it, would you like to be changed back.
I: Yeah.
R: We see it differently. In this area, we complement each other better because we talk, and there is no attrition, from my professional relationship with medicines management.
I: So, a lot of that is based around that relationship.
R: Yeah. I think that relationship is most important.
I: Yeah.
R: Especially when medicine management understand what clinical and practice pharmacists are in practices for.

GP3
Very rarely will I do an acute that an alert comes up on. Normally it's when I'm doing a medication review that's when I see it. Because my acutes, hopefully I've worked them all out in my head and I've just had a look and I've matched it. Because it's easy to do, check one against eight, but to actually do eight against eight is a far more difficult thing, if you see what I mean. So I'm checking this one fits in, that's relatively easy, but to check they all fit in with each other is a far more difficult thing. So [inaudible 0:28:31] is coming up more when I'm doing my medication reviews. Medication reviews I do here. This probably might explain why we've got less here. We do a six month or annual review on people that are only on two things here, and we've done it for so long that it's relatively slick. Whereas other places where they've not had review I'm going into a jungle almost of interactions and possible problems. They've never had any safety system before, so there's lots of alerts will go on. It's amazingly different, the more I think about it. I'll leave it at that, sorry.

GP3
I: Right, okay, yeah. How about patients themselves? Presumably within the consult…does it lengthen the consult or make it more difficult or longer?

R: It potentially would. Again, that's what I'm saying, it depends how I react to… In some ways I'll let it deliberately lengthen the consultation if I'm at the other place and I just need its help to go through things to be safe. So it will take a bit longer. I don't mind that if it's thinking time and I'm incorporating it. Sometimes the bit that it doesn't do that I have to add to it is does this patient need this medication in the first place. And it doesn't do that for you yet. So I have to do that bit. It might suggest reducing dose or change…but it rarely says is this indicated, which is the bit I've got to do when I do a medication review.

I: Yes.

R: So I need to fit in with that. And if I get enough red star warnings I've got to think is this worth the risk and how do I impart that to the patient that this has got some risk, what level of risk it is.

GP3
I: The system obviously ties to the individual patient. Those alerts are about that patient or that patient's medicines. Do you think that is a good thing? Do you think that helps?

R: Yeah. Otherwise I'd ignore it.

I: Yeah. If it was just a generic rule, don't prescribe X with X, is that…?

R: No. Being individualised really helps doesn't it? Because otherwise you're missing a trick. Because that's the bit I would do anyway in my head I would hope. So in some ways it's adding a little bit more on safety wise. Again, the safety is not an issue. That seems to be well referenced and appropriate. It's just the other bits.

GP3

I: No, it's more difficult to. And in terms of actually using the system, if you can just describe how from your point of view how you use it, how it's part of your work as it were.

R: When it comes up it will give me a suggestion of what to do. It will give me some information that most of the time I've seen before, so I know the next step. So it's whether I agree with it or I do the "not suitable for this patient" or some other information down that side. In some ways I think my default in over 50 per cent is "not suitable for this patient" at the other place. Here my default would probably be to put the switch screen that says use original, and that's fine, I just click on the convert. There's one that I struggle with, and again it's my age, when you've got a default trade name. I struggle with this. Because in all my training in all my life it's been beaten out of me that I'm never to do this, and then suddenly tells me it's all right.

I: Because you normally default to generics.

R: Yeah, I have a generic default. It's a very strong default. So, for example, the amoxicillin to amoxo, it hurts. It doesn't feel right. And again it's an easy one and it makes no sense, it's no cost, but the worst thing I would ever get was a patient saying I don't want amoxicillin, I want amoxo. So I'm always just a little bit wary about those switches. I do do them. The creams and things tend to be the interesting ones because they tend to be quite seasonal, the changes. The one I can think of recently is the beclomethasone to betnovate, which again just feels a bit odd. But if I've got enough time I click on as many as I think is reasonable. There's often as I'm going through a surgery and when I first did the other surgery I had over 300 patients on over 25 medications, and that is impossible for (the CDS) to help me. I've got to be really quite selective about what I do. Because if I've got 20 alerts up when I'm doing a medication review I cannot deal with 20. But I've learned to be a bit more selective about it, and I'll go for the low hanging fruit, and I'll perhaps do a little bit in the notes to say discuss such and such next time. Hopefully we get maybe less and less with time. I think they probably have already. The first month I was there it was [inaudible 0:18:25].

I: So do you think there is a distinction or a contrast between the tool running as a useful aid to prescribing for polypharmacy, people with multi-morbidity, people with 25 medicines or whatever to people who are coming from just one…?

R: Yeah. The people that would be best dealing with sometimes the polypharmacy are the pharmacists that have got a bit more time rather than ten minutes, because it would be a good reminder to them. Because they're human like me. It's a good safety and cost reminder, but you just need a bit more time for it. The original polypharmacy I said to them if someone's got over 25 medications I need more than ten minutes because I just cannot do it, it's not humanly possible. But I haven't got more than ten minutes so I'm in a catch 22. So perhaps it's thinking about the pharmacist when she uses that on the multiple ones, she takes a bit more time and maybe has a plan for the patient. Because I think probably there's no point changing 12 things at once. It will not succeed. And I am very male about this, I take a single task and if I can get that single task done then I'll move on to the next one. And I think the difference between us as good prescribers here and pretty shocking prescribers elsewhere, that's why (the CDS) works in a very different way. But we are getting a little bit of battle fatigue with it, and the fact that I will go to "not suitable for this patient" a bit quicker than I would have three months ago even […] how long I might be going to use that thing for so I might for example choose to override a concern because I've decided that I'm only going to use it for two weeks or something, whereas the problem that it's highlighted might apply to a longer term use.

GP4

R: I think it's just the fact that it…that would be the same for all of the alerts I suppose. It's just for some of them it's a disadvantage because it's intrusive and with (the CDS), it's neutral in terms of its distraction value, I suppose.
I: Right, yes.
R: It's not taking you away from what you are wanting to do, if anything it's augmenting the task that you are doing, it's making it safer I suppose or making you prescribe something which is less likely to get you wrapped on the knuckles by the pharmacy advisor or something.

GP4

R: Yeah, but that's not to say that I'm right. It's an odd…it's just there's so many things you're juggling at the same time about… So technically what (the CDS) is telling me is correct, but I'm also judging that against what I think the patient might be willing to take…

GP4

R: That's a decision I make with a patient and I'm on this occasion not going to take notice of that because I think the indication outweighs the risk that you've highlighted, so… And I think if that was happening a lot, I think if that was constantly jarring and it was telling me to do things that I was finding irritating, then I would say so but I don't really get the impression it is hugely and when it does I close it down.

I: So when you feel that your expertise takes over, as it were, oh no, I'm doing this, yeah? 'Cause that's really…that's…I find that really an interesting area, that whole …it's not a decision system, it's a decision support system.

R: Yeah. I think that's right.

I: The expertise of prescribers has still got to be there.

R: Yeah, but that's not to say that I'm right. It's an odd…it's just there's so many things you're juggling at the same time about… So technically what (the CDS) is telling me is correct, but I'm also judging that against what I think the patient might be willing to take…

I: Yeah, precisely.

R: …how long I might be going to use that thing for so I might for example choose to override a concern because I've decided that I'm only going to use it for two weeks or something, whereas the problem that it's highlighted might apply to a longer term use.

GP4

R: Yeah, but that's not to say that I'm right. It's an odd…it's just there's so many things you're juggling at the same time about… So technically what (the CDS) is telling me is correct, but I'm also judging that against what I think the patient might be willing to take…

I: Yeah, precisely.

R: …how long I might be going to use that thing for so I might for example choose to override a concern because I've decided that I'm only going to use it for two weeks or something, whereas the problem that it's highlighted might apply to a longer term use.

GP4
R: So some of that must be working well if…'cause I don't notice it. It would be irritating if every time you open up a 7 year-old's – well, that's a bad example, you wouldn't try and prescribe a 7 year-old with a non-steroidal really would you – but if it happens every time you…somebody with a sore knee, he was 30, gave you a blanket…or a warning about non-steroidals and all the things that might happen then that would be annoying. And that doesn't seem to happen.

GP6
R: Yes and that's the problem, you see. So if that popped up it might actually, I mean, obviously I'm fantastic clinician and I would never describe an anti-inflammatory with that but, you know, it might not have occurred to me that person was over 65 and things, so that would be useful in a one off. I suppose the problem is that you're actually 76 and you've got six different co-morbidities and that's when it just becomes overwhelming because obviously there are lots of issues that are potentially already going on with their other medications, so it all pops up at once. And it's interesting when you're doing medical reviews, you see, because then things pop up then as well but they are all generally about changing to cheap alternatives. I mean, fortunately sometimes it makes good recommendations, like it says, you should be giving Ventolin rather than Salbutamol, sometimes like that. So those sorts of recommendations I would say I would pay more attention to than the alerts which actually is a bit of a worry because clinically the alerts are probably the more important things to be paying attention to.

GP6
And it should know what your most recent EGFR is. The problem is, that if that person has at any point in the past had some, sort of, renal issue it might be that, that's what's triggered it but it's just so annoying because if that person actually has got an EGFR of under 30, I wish it just popped up saying, not safe, this person's EGFR is less than 30 but it doesn't, it just doesn't. It gives too many random options. It then makes me, to be truthful, I was just thinking I had just randomly said I just ignore it and I actually don't, because I think sometimes a little reminder like that maybe does make me go on to just check their recent renal function and make sure that it is suitable. But I think it's actually also a little bit random in that, I'm not sure that it always does it appropriately. I think, for instance, there are drugs like Nitrofurantoin and Trimethoprim which do rely on good renal function to be…to work, because of their function through the kidney. Well actually Nitrofurantoin does and Trimethoprim actually can damage your kidneys if your function is too poor. But those are both drugs that you would tend to use in old folk with urine infections but it's a bit random about telling you about whether they are suitable to give to a person. They only flash up, you know, so it's just too unreliable I think for me to want to read it every time it pops up.

GP8
I think the only think I would say on that is that it's a lot easier if we're trying to be persuaded to prescribe less, so if (the CDS)is coming up saying we recommend the patient buys this over the counter. It is a lot easier to say that on an acute than going back on something that people have had prescribed for years. So when people have had their anti-histamines and nose sprays prescribed for years and to go to re-authorise them and it suddenly says they need to buy it over the counter, people take that less well I think than if it's an acute prescription and you say, oh no, we can't prescribe this, you need to go and buy it.

GP10
I: I'm quite interested in the idea that it's – as well – that (the CDS) is this expert, this knowledge base, this thing that's telling you what to do. And that bit is a bit sort of like, well, to what extent then do you rely upon that? Or are you doing rely upon you when you come to…when you see those alerts, are you just going, oh, yeah, I'll do that, or are you then thinking about your own…?
R2: Variety really. I think if the alert comes up, one thing's in viewing the highlights, is the importance of the interaction with (name of CCG pharmacist). And I suppose if the alert is bringing something up that Jill's said, it just reinforces it a million times. If the alert is bringing up something that is not on the radar already, then we always check to see why…or I personally, I'm sure…to check why that is, why it's coming up. But occasionally like this morning, there was one that it specifically…it wasn't a consultant but it was one of their specialists has asked for very specific medication that normally wouldn't be given, and of course the (the CDS) comes up with that immediately as, this is not the one that's first line or appropriate or whatever. Now, as it happens, that message from the nurse specialist was only in this morning anyway, so I knew straight away that I could override that. And then you get the ones where… So those are the most common, leaving just a smattering of a few that come up with, this is not appropriate or this is not following guidelines or whatever, where actually they've had it for yonks and the trouble of changing it probably far exceeds any benefits or any real gain. I mean, you have to make that decision whether it's raised at the time and obviously if it's something that's you know full well costing a small fortune or it really has been proven to be effective then we do look at it, but there are somewhere you go now, it's a regular thing and been had for 20-odd years, I isn't going to go there to change it now, yeah.

GP 11
I: Would there be any sort of decision making there? Would you sort of look at the alert and think, do I agree with that one?

R: Yes. So, I think it's always useful information to have. The times that I think probably all of us would be overriding is when we've shared that information and patient dissent, I think. I mean, I think this is coming from more of a pharmacist review, but, you know, when we're trying to do one of the tricyclic high dose in elderly, practice-wide switch. And actually, lots of the patients are really quite stuck on it. And increasingly, every time we're trying to prescribe correctly, it's flashing up as did you know this is horrendously dangerous? And we're sharing this, and we've got a lady who, she's got ECG changes and [if we say 07.06], okay, if we treat your anxiety, literally your heart might stop. Yes, that's fine. Informed dissent, you know.

GP 11

I: Yes. So, you wouldn't do it and if you'd shared that information with the patient? It's also interesting as well because talking to other people, people have sort of talked about that if an alert came up on the screen, they wouldn't actually tell the patient that, that was actually happening. Or they wouldn't actually talk about why they were changing... Well, this computer has told me change the medicine. So, you would be prepared to talk through some of that with patients?

R: Yes, I think so. Well, it's thinking of the terminology with it. And again, there seems to be some variety in the categories of this. I think particularly now that I've been here a couple of years and there's been a few practice-wide changes that have been as part of (names of places) wide changes. I think knowing where they've come from and, you know, are they based on safety and or cost effectiveness, I think getting into a bit of patter of we're looking to change Seretide to Fostair and, you know, this is through a mix of effectiveness and de, de, de, what do you think? And then, see where people fall with that.

GP 11

R: Yes. So, whether it's a difference between long-term patient safety versus acute, I think that's pretty much exactly how I think of the divide in it.

I: In what sort of way?

R: So, I think lots of the things that PINCER picked up is the sort of stuff we miss because it's long-term safety. So, things like the naproxen has continued on for a stupid number of years. Let's try and sort that out, for example. And I think it's quite easy when dealing with the acute, which is keeping patients safe but in a very different way, to ignore that and defer the meds review for another time which doesn't happen because the workload of the acute is high.

I: Yes. So, you're constantly parking the meds review because you're dealing with what needs to be dealt with now and so [actually 18.15] the safe issue is this bit and that safety bit in a way. So, PINCER has that effective [period 18.21] with looking at that stuff then, doesn't it?

R: Yes, it does. And I think particularly without acute because that's what most of our patients naturally do. So, in lots of cases we are trying to chase down the long-term. And, still, patients, here in particular, will often present acutely chaotically and you do other things in a mess.

GP 11

R: Yes. So, I think complexity and the relative acute versus long-term demand and arguably, NHS wide, the supply demand mismatch. But yes, I think particularly for most of the doctors dealing with the acute it's pretty much a full-time job.

I: Yes. So, you're dealing with the here and now problem and not something that is to be dealt with. If you take the PINCER approach and the Optimise, so that you've got your safety messages at the point of prescribing, safety messages through an audit and a pharmacist, coming to doctors saying you shouldn't have done that or whatever, no not saying you shouldn't have done that, discussing that prescribing in the practice where they are based, which of those approaches, do you think, serves people better, serves clinicians better, serves patients better?

R: Yes, interesting. I wonder if (the CDS) gets used more routinely in a wider range of scenarios. And thinking of acute long-term, we're probably more specifically through that. From my memory, most of the people who are on PINCER are more long-term and [if I was 21.29], you know, pretty much PINCER would pick up everybody.

I: [As you said 21.35], it's been prescribed for two years.

R: Yes, absolutely.

I: And they've never ever had gastric protection with it.

R: Yes, definitely. So, I think that would be an advantage of (the CDS)as the everything. I think, as I've mentioned, there's the variation in how used and overridden (the CDS) is, I think. And I would guess, but I'm not sure, that there's more variation with that than with PINCER or something. Yes, I think PINCER probably pins you down to do something.

GP 11

I: But from what you've described, do things do then fall through the first net and are captured on the second net?

R: I think they do. That's how I think of it in my head anyway because I think it's so easy to override (the CDS) for correct or incorrect reasons and different people will have different things on it. So, it's useful information to have but it doesn't tie you and there's good and bad about that. So, I think having something else that then says, alright well here's the audit outcomes of how are you prescribing for good or bad? Well, for bad, isn't it? So, let's have a look at it.

GP 11

I: And having more information, even though you get all those alerts.

R: Yes. Because, as I say, as far as I can think, the only way to make it less overwhelming would be make it less true.

I: Yes, and in some respects that brings on to a little bit here in terms of the information it provides, it says here time irrelevant and useful. What I'd go on to say further about is how important is it that the information is time irrelevant and useful?

GP 11

I: One of the things that I've sort of recently been asking people is, do you think it has improved care and do you think it has the potential to continue to improve care?

R: It's definitely a way of disseminating, I guess, CCG prescription decisions and evidence. So, I think that's the biggest, quickest thing I can think of because probably most people don't read the new formulary guidance or APC or whatever. So, yes, I've certainly come across anecdotal things, either myself or with other people, where safety things where...well, I was going to do this but there's this alert, let's rethink of that. I mean, the cost effectiveness is a tricky one. But you could then argue, improvement of care across a population level, how do we best use the resources et cetera?

I: Yes, precisely. Because if you're going to save money here, you can put resources into this group of people here.

R: Yes. And I think if you didn't do that alert, everyone would always continue to do the same thing that they did having not read the emails before. So, yes, I think it's a really efficient way to tell people of the changes in that pathway.

GP13
R1: And, I think, we had a meeting yesterday about skill mix and what have you, and that's probably one of the issues in general practice, is that we all try and do everything, whereas in fact, what we're trained to do as doctors, is to do the difficult stuff, being pragmatic, the balancing two, three, four, competing risks, and working out what to do despite them, and how to manage them, and how to safety net them and all that sort of stuff, and (the CDS)definitely helps from that, because anything that helps remind you of what the risks are, helps you manage them better. And so, what might have been, 15 years ago, a prescribing error, you could do exactly the same thing, but at least you're doing it as a consciously managed risk now, rather than as a prescribing error.

I: Yeah, rather than just simple, oh you shouldn't have done that, it's a, well there's a decision process towards that, and it's around…?

R1: I can give you an example. So, we recently had a woman in a nursing home in AF, who 15 years ago, would never have been anticoagulated, because she sits in a chair in a nursing home, and is hoisted and stuff like that. But, because of decision support, not just Optimise, but various methods of decision support, and [inaudible 00:12:36] she was put on rivaroxaban for her AF, and it was managed appropriately, and then she fell, and she's recently died of an intracerebral haemorrhage. But I think 15 years ago, we would have been thinking, oh blimey, what did we do there, was that the wrong thing to do? Or, if she'd had a stroke and not had it, we would have gone, oh there we go, that's the… Whereas now we can go, actually, that was still the right management, she wasn't falling, she had AF, so whilst she's had a bad outcome, but actually she's been on rivaroxaban five years, and she might have had a stroke three years ago, and been disabled. So yeah, so that's an example of how, 15 years ago, it could have just been not even noticed as poor prescribing.

GP13
I: Which of those two (PINCER and Optimise) approaches is, not better, but which of those two approaches is more valuable, and do you need one or the other, or are they complimentary? Sorry, that's three questions in one.

R1: No, no, 'cause the questions work through the thought processes as I was having them, I think you need them both, because if you don't have the PINCER type thing, the retrospective look back at how it did, then that's the equivalent at taking the windows out of a Google car. It's putting complete faith in the Optimise, and saying, we don't even need to find out how good it is […] And also, there's the danger that there is the possibility that, if subconsciously our prescribing risk management, if we subcontract that risk to (the CDS) subconsciously, and don't know we're doing it, then actually our PINCER stuff could get worse, so you need to know. I think anybody signing a prescription who understands what their signature actually means on that prescription, would always want to look back as well as forward.

GP14
I: Yes, precisely. Does it…in which case when you're…in fact, I can…this issue around multiple morbidity and polypharmacies and which has been raised by other prescribers and, I've had two different messages. One which is said, well, actually that's where (the CDS) is quite useful. Others have said, (the CDS) is useful for the one of acute and actually when you've got a polypharmacy patient, you've got… it's far too much to deal with and there's too many alerts happening and, to be honest, it's just confusing the picture. So, which of those do you think is…?
R: I mean, the first one. I think you're better getting the alerts and be aware of it and make your own decision to ignore or cancel it, because these are just suggestions, they're not…you don't have to follow through with it. But from my personal point of view, particular from a safety aspect, I would rather know even if it's flashing up loads and ignore them all, if I'm going to take that responsibility and carry on with prescribing whatever that job might be. I would personally rather know but I can see there's two different angles from it in a sense isn't there?

GP14I: Yeah precisely. Let's talk through, about what you, how you use the system. Could you, I mean, this has come back into some of that stuff you were talking about with the patients and how you would respond to those alerts. Can you just talk me through, you're sitting there prescribing and alerts come up? What do you do, what happens?
R: So, if I was prescribing, I don't know for example, say I saw, I don't know, a sick kid or something like that, and I was prescribing some paracetamol suspension. I think, one of the alerts it comes up is that the sugar free suspension is slightly cheaper than the sugared one or something. So, it would come up as I'm prescribing, because you'll often click on the one on top of the list thinking that's the most commonly prescribed one, seems fine, and then it will come on and give you that suggestion. Then you read and take stock of that suggestion, patient still in the room, and go, yeah, okay, and then read and do your prescribing a different way and prescribe the alternative. Or sometimes, you'll go, well actually I'm not going to do that, and you can click ignore, and ignore it and carry on prescribing what you were going to prescribe in the first place. So, it varies according to the circumstance and what's popped up based on that.

GP14I: Yeah. One of the things you talked about when you were talking about the complex patients particularly was you talked about the, that responsibility around what would, you take it in those ones, well I'm not going to accept that. To what extent…what are the occasions when you don't accept the alerts and what's the thinking behind some of that?

R: I need to try and think of some examples about that now. I don't know. I'm trying to think of an example, I can't think of a real one but for example, I don't know, maybe if it came up with an alert over an antibiotic you're trying to prescribe, maybe there was an alternative one that they suggest.
You could think, well actually clinically they need this on for whatever reason, because they can't chuck everything about the patient that's in front of you there.
So, there are times where you might think that your clinical judgement can override whatever their guideline or suggestion is. I can't think of a very good example of that really, that's not the best one to be honest.

GP14
I: Yeah. And you talked about responsibility there, so, is the system taking away responsibility to do it, or have you still got that responsibility?
R: No, overall your scribble is on that signature or if it's an electronic signature, you'd have responsibility for that medicine that you've just given that patient. Whether it's been advised on the computer or not it's still your responsibility.
So, if you follow that guidance or whatever you want to call it, then it's still your responsibility to justify why you did, or whether you changed what you were going to prescribe. And, likewise, if you decided not to go with the suggestion it's still your responsibility and would need to justify why you did that, in your own mind or if anything God forbid happened to the patient.

Sub theme 4b. v: Relevance and reliability of alerts

<u>CCG Staff</u>
CCG Pharmacist 5
I: And you talked about making the alerts timely and relevant for the GPs which is a, yeah, I mean, that's something that we've picked up on as being important. Some GPs, not necessarily within this area, but certainly some GPs around, you know, they've just, sort of, moaned, as GPs do, moaned about alerts. Too many, far too many, loads of stuff all over the place, we're getting too many. And one of the things I've...it's...and there's not just alerts coming from (the CDS) there's alerts coming from [inaudible 00:10:31] as well.
I: I know, they come from everywhere, yeah.
I: So, how do you make it that the one, you know, that the important safety alert, how prescribers...you know, the PINCER alert that's embedded in (the CDS) ,how do you make it that that GP sees that alert as being timely and relevant?
R: I don't think you can change the look of the message to make that one stand out against all others, so the feedback we've had from GPs is, if they haven't heard about it from elsewhere, they generally ignore the message. So, I think the message to our team is (the CDS)alone won't deliver an action on that safety message. But, if we back it up with an alert to GPs saying, look, sodium valproate, you need to counsel your patients who might become pregnant, you need to stop it, you need to do this, that. They then have that knowledge and (the CDS)is a reminder. And that's where, I think, (the CDS) works best, where the GPs already know the information, the message pops up and they go, oh, thank goodness that popped up because that stopped me from doing this.

CCG Pharmacist 5
I: Yes, I mean, I can see that. But, you said that you feel that the safety ones, the cost saving ones are not getting a look. Because one of the things that I was thinking was that, from what we've been finding is that, you know, where this, you know, you're getting constant bombardment of cost saving, and often GPs will say, oh, it's just to save a penny. And...
R: That's when we try and limit the number of cost saving messages that it gets, so we try not to turn on the really low value ones. And yeah, I think possibly, the most valuable ones we perhaps wouldn't just do as a simple message, we'd put an explanation with it, so that you could put in the titles something like, massive cost saving all over it], I don't know. Yeah, but most of them are fairly average sorts of savings and they'll just accept it. So...
I: And the problem is that, you know, even if it is a 20p savings, you have to think, it's not just you doing it, is it? It's across a CCG, across the country.
R: Exactly.
I: Once you've done all of that though, if it's a very, very commonly prescribed drug, it's going to add up, isn't it?
R: Yeah. I think one of the problems with the cost saving messages is often that cost saving is done per dose, so it doesn't look that huge. Whereas, if they gave the cost saving per month or per year, I think that would have a bigger impact .
I: Oh yeah. So, if it's 10p per day, but they're taking it four times a day...
R: It would be like, oh that 10p...

CCG Pharmacist 5
I: I mean, in both those, sort of, bits of the, you know, it's not just feedback is it, the relationships you have with the GP, it's the the relationship you have with the software developers. What are the things within those relationships that are really like, key to keeping it going and, you know, avoiding GPs just switching everything off or avoiding the CCG saying, no we don't want something?
R: I think accuracy and quality, and understanding of what we're actually trying to achieve. So, yeah, if we ask for a message, we need to know that they absolutely understand their side of it and technically, this is going to work. But also, clinically, checking that what we're asking is actually clinically relevant, and being able to have that professional discussion with them and know that they do actually understand what we're trying to achieve and what national guidelines are out there really. So, I think yeah, quality of staff and their knowledge is really important. And yeah, the understanding of practice pharmacy and general GP practices and how they work and how little time they actually have.

<u>GP Staff</u>
GP Nurse 2
I: I mean you said you get alerts a bit more targeted now but, you know, when you've got a patient there with lots of different medicines are you getting lots of alerts coming through?
R: No, not really. It's more when you're actually trying to prescribe something. Obviously, I think the ones where there's more as well is ones that have been probably taken off formulary like the antihistamines. It's pushing you to not prescribe it to get the patient to buy it over the counter. Also, the contraception ones it'll always give you the alternative, cheaper version. [...] So, yes, they're the ones that you tend to see quite a lot. Any contraception that I try to prescribe, it will always veer me towards the cheaper version or the formulary one.

GP Pharmacist 1 and GP10
R2: [Voices overlap 08:39] more appropriate for acute. I don't think it's not appropriate for chronic. My personal feeling is that I don't ever mind pop-ups coming up. Yeah, whether it's acute or chronic. The only slight rider to that is that I'm probably more likely to tick, not suitable for this patient, if it's something that's a chronic condition, that they've had something for a long period of time and I know it's not on anybody's radar and I know that it's not gone against guidelines and so on and then I'm likely to put, not appropriate for this patient. Yeah. So, I personally don't mind it. I don't think [inaudible 09:17]. It's minimal amounts really of interference…
R1: 'Cause you just…
R2: …in a day's work.

GP4
R: I think it's…I'm trying to get to the nub of why, what would make it feel intrusive versus any other sort of reminder or pop up, and I think for me it's two things, it's quite often it's useful and I can see the point of it, and also it's easy to overrule it if I don't want to…and, for example, the QOF pop up that might be saying to you, you know with patient, not that it's irrelevant to what you are doing because obviously it comes up when you are trying to prescribe something. So, you have got the patient in front of you, you need to prescribe them something, you have decided that's what you want to do, and this system makes me feel that it's helping me to do that in a cost effective way, and in a way that's safe. Whereas a QOF pop up, for example, can be just completely left field, it can be it's just noticed that this patient hasn't been asked about smoking or they need a BP doing, but actually they've come because they are distressed because they've lost their job or something. So, they can be very intrusive and not relevant. It's sort of a non sequitur really, whereas the (the CDS) comes up when you need it really, it's not something that just bounces up in your face when you're doing another unrelated task really

I: ...and they're taking it four times a day and they're taking it all day.... for the next year, it starts getting to be a lot more.
R: Yeah, it's that...so I think that might be useful if they could, yeah, change the way they've...they might have done it already, but..

<u>Theme 5: Work practices and workflow - fitting (the CDS) in, the impact of (the CDS) on work.</u>

<u>CCG Staff</u>
CCG Pharmacist 5
R: Generally, most GPs don't complain that they get too many messages. And I think many realise that if they're getting a lot of messages perhaps they need to question whether or not they're coding their patients properly. You know, if they're actually, perhaps not up to date with what they know. But...
I: Because, if the coding is not good…
R: Yeah, if the coding isn't good they'll get more messages than they should do because all of the messages are tailored, so they're very specific messages, most of them. So, there's a logic behind them, they'll only pop up for patients with certain conditions, and if you haven't coded that condition then, or have coded the condition incorrectly, you're going to get...
I: Or if you've coded it more broadly.
R: Yeah. Or, if you're diagnosing something and you prescribe before you add the read code to the record, you're not going to get the tailored messages. So, you might get extra ones saying, well you can't prescribe that because this patient hasn't got that condition. But it's just they did it in the wrong order, so teaching [voices overlap 00:25:58].
I: Yeah, so you should rather diagnose before, diagnose the symptoms then...
R: Yeah, code it first and then prescribe.
I: Yeah. And because it's so dependent upon that, and presumably as well, if, as you say, there's a broader...if they get it wrong they just get all the messages come, because there's too many in there.
R: Yeah, you will get the generic ones.
I: The generic, precisely, yeah. It cuts out that very, very specific message for that particular patient.

<u>Sub Theme 5i: Fitting within or alongside other interventions and technologies</u>

<u>CCG Staff</u>
CCG Pharmacist 5
I: I don't know whether it was yourselves or whether it was somewhere else, people were saying that, sort of, usu..Yes, are the CCG…either yourselves or some other CCGs were talking about the using (the CDS) for specific things. And I think, and maybe that's also, I've had that come down from FDB as well, have you done that, or have you thought of using it in that way?
R: Yeah. So, the example that a new type of message, I don't think we really twigged it was a new type of message, but splenectomies, so people who've have their spleens removed need vaccines to prevent infections in the future. And so, we've done a big piece of work in (name of place) where we've audited to see if practices do give those vaccinations. And it showed that, actually, they weren't always giving what they should. So, we wanted a message to check if somebody's coded as having a splenectomy, we wanted the message that (the CDS)check which vaccines they'd had and give a warning that you've got a splenectomy patient, they're missing this one, this one. So, they've done that for us, which is really helpful. So, it's backing up the work we've already done, we've already trained the GPs, they know that that's what they should be doing and hopefully, this is a reminder to actually, oh, yeah, I need to do that, I need to book that patient in. And it's clever enough to also pick up patients who declined it. So, you won't get a message if the patient has been offered the vaccine but they've said, no. So, we've given the GPs a raft of codes to use to say, no, you're not having that one.

CCG Pharmacist 7 and CCG Pharmacy Technician 2
R1: I mean, by now most of them expect some sort of message to come through the messaging system because it's like coming up for nine years that they've had it now, and certainly when we were looking to change it from (a previous system) to (the CDS) or considering whether we were going to change or not... You know, because one of the options obviously we had was, well, we don't have any system, but no-one didn't want any system; they all said they wanted messages. So that was…there was a sort of consensus. I think, based on the time they've had some sort

of messaging system, they all kind of wanted it, and they all expected it. They don't kind of think, why am I getting this message? They just expect it.
I: Do you…so you don't get any sort of negative sort of, oh we get too many alerts, or anything like that?
R2: No, I've not had any.
R1: I've not heard that. I think because it's…because of the mechanism for triggering the message it's a lot more targeted than Script Switch was. So Script Switch we used to get that because, like, if you put one on for an antibiotic it'd hit every single issue of antibiotic, whereas with this it might be…you know, you can target it by age, you can target it by condition. So the trigger points…so you're actually seeing…the practice is probably seeing less. It's not that sort of blunder bus approach, if you know what I mean, it's a bit more targeted. So I think that probably does help a lot, because we did with Script Switch, have people moaning. In fact the last one switched off, didn't they, one of the practices?
R2: Yeah.
R1: Whereas that's not been the case with this, so...

CCG Pharmacist 7 and CCG Pharmacy Technician 2
R2: Yeah, because there's other ways you can do that work.
R1: Yeah.
R2: And sometimes you need that human touch instead to them...
I: That's really interesting because that's…can you go into that in more detail?
R2: So for example if there's a switch from, say, modified release docs as a [inaudible 00:21:56] to the plain docs [inaudible 00:21:58], as a switch, that might involve…well, it is really involved, you have to check someone's blood pressure a couple of weeks after. So if you add that on as a switch a lot of people wouldn't be bothered to accept the switch because then they have to arrange an appointment and they've got to speak to the patient, whereas then I moved it into our technicians' work stream, which is what we have, the technicians, all the work streams we work on, so when it went into that, it gets picked up that way. So we can run off the list of patients and actually go and speak to the practice and say, we've found this, this is what'll be involved, do you want us to make the switches, send a letter out to say, you need to book it in two weeks? Or some GP surgeries have the blood pressure machine in the waiting room. So just having a bit more of a human touch to it…
If there are too many steps involved with a pop-up it's not going to work, or if there's something that it's flagging you need to review this, it doesn't always work either because, well, I'm not…I'm doing this…I'm in this consultation for something else, I don't have time to do this review. You know, so for things like that what we've found is, there's point having that message keep coming up, you need to review this patient, you need to… So with certain things, you know, like for example, inhaler reviews, it's not always the best way to flag that up.
I: So basically, the better approach is for you as a CCG to be involved in supporting the practice, identify those patients than doing…?
R1: Yeah, there's other ways of doing the work.

CCG Pharmacist 7 and CCG Pharmacy Technician 2
R2: There's just too much, yeah. Well, that's handy for us to see, because there are things you can raise from it, you know, or if you're doing a practice review. Say if you had a meeting and you see a lot of quality stuff getting ignored, there might be some that don't necessarily need a lot of work, but actually this shows you've got so many patients on these kind of drugs that could actually…you could add up a lot of falls and accidents from that. Just to take that to a meeting and highlight it that way is sometimes better than having it keep popping up.
I: Yes, but you're finding out about that because of the rejection and the alerts.
R1 & R2: Yeah.
I: So there's a sort of feedback system happening there, isn't there, as well?
R2: Yeah, because you can put messages on mute to see whether…how often it would fire, so you can practise things as well or see how things run in the background. So if a practice was…you know, it would literally…if it was popping up all the time and it was a safety message then that's quite handy to have that information, especially if there was like a CQC coming up or they'd failed a CQC.

CCG Pharmacist 7 and CCG Pharmacy Technician 2
I: One of the intriguing things I found about that was that they were sort of rather…I'd said about review there – they picked up on this concept that, you know, (the CDS) would then be acute and Analyse would be more looking at long-term conditions and the long-term management of patients for reviews and stuff. Do you think therefore that (the CDS) is actually better just as an acute sort of alert, or is it something which can be part of…?
R1: No, because when you think about it most of these scripts done are repeat scripts, and so if it's making an intervention on a repeat system then, you know, I don't see that it then should be just like an acute intervention. My understanding from the little bit I've seen of Analyse is that it's a more sort of proactive thing, so you're utilising the similar mechanisms but you're actually going looking for things rather than waiting till they present themselves.
I: Yeah, precisely.
R1: So obviously that's… But then there's other things that do that as well, so…you know what I mean, so you get SMASH, PINCER, [Pinga 39:35], you name it. So it's already in a crowded field whereas the (the CDS) to my knowledge there's always (the CDS) and Script Switch that do that, so it's a less crowded field. But it's up to them if they want to try and develop it but they're going up against some quite robust and already well developed systems – SMASH, PINCER, [Pinga 39:57], you know what I mean, that have got back in…they're already in the system that are well, sort of, tested, that have got Health Innovation (name of place), so regional funding backing. So I don't know.

<u>GP Staff</u>
GP Nurse 2
I: I remember you talking about how that felt…you know, it was different but the system was very helpful because of [inaudible 01:55]. How do you feel, you know, what, we're a year and a bit on from when we spoke? Is it still helpful in that way?
R: Yes, very much so but one thing that we have done, I don't know if you've heard of it as well but we've also got a company called Ardens in and we use their templates.
I: Oh, right, yes.
R: As part of their templates they have the formulary on so, for example, if you see a patient who's had an animal bite, then you can look on the formulary for animal bites and it will guide you to what… So, I guess it's another safety net, really.

GP Nurse 2

I: You talked about that other system, the Ardens system that you were…again, would that be part of that, if you like, using it in complement to other things, as it were?
R: I think I would, yes, because with the Ardens template they've got formularies, say, for example, migraines so you can look at that and use it alongside as well. It's just another tool so you've got all your tools there, then, that you can use. It's got the formularies, so for example, there's one for, I don't know, let me have a look. I'll have a look on it for you. Antihistamines, for example.
I: Right.
R: Then there'll be formularies so there'll be things on there that it'll be saying to you which ones you can prescribe with the ages which I find quite helpful so you know for your children it'll work that out for you which is excellent.
I: Right, yes.
R: So, in that dosing if you're using it through (the CDS), it's belt and braces then, isn't it?
I: Yes.
R: Yes, so I find it all really helpful to be honest.

GP Pharmacist 1 and GP10
I: Yes. Do you think some of those projects that…as you say, that input from CCG pharmacists coming in, how does that align if you like with the work that's happening in Optimise? Are they saying similar things and you getting similar alerts, or…?
R1: Yeah, so they'll pick up if we've got a big lot of prescribing that's out of line. So, the most recent example is the Vitamin D, isn't it?
R2: Yeah.
R1: Can't remember if there's an (the CDS) on that or not. So, we had a lot of generic Vitamin D supplements, so Colecalciferol, and that's no good because then they can spend virtually anything and it costs you a fortune. So, then she'll say, actually, you should be prescribing whatever the current favourite is. It was [inaudible 03:32], then it was [inaudible 03:33], now it's going to be something else. But those are the kind of things (name) will…even though she's not here routinely, she's routinely looking at our data and she'll fire us a little email and keep us informed and then yeah, I…

GP Pharmacist 1 and GP10
I: I'm just thinking that connection then to the CCG, and that link through (name) to the CCG, because one of the things that actually I'm becoming interested in is the way in which (the CDS) as a system, it's okay, it's giving you all these alerts, but you've also got to have that other bit that's coming from them, haven't you?
R1: Well, it's like the PINCER. It's like the PINCER theory, isn't it? You've got the information but if you've got somebody who's going to fix it for you or highlight it to you then you're more likely to do something with it, aren't you?
I: Right. Yeah.
R1: So, (name) will pick up… I mean, obviously, she's not going to pick up individual things which PINCER or (the CDS) will pick up, your one-off things…
I: One-off.
R1: …as you're prescribing them. But if there's an area that…'cause she'll look obviously at our ePACT prescribing data, so they monitor that continuously. So, she picks up on an area, then she'll take it up with me and then I'll take it up with everybody and we'll do something about it, so that's how it works. And that works very well for us because yeah, we work well together and that works.

Sub-theme. 5ii.Efficiency, Functionality, Ease of use, User interface

Software developer staff
Software developer staff 1
The actual usability hasn't changed much for prescribers. That's the only, I think, downfall is that we've not been able to make it slicker for prescribers. The often complaint is there's too many clicks. And that's because, one, we all say, oh, there's a…because we'll have a button for, do you want to see the reference information, or do you want to read all the text, do you want to see the alternate products. And these are all clicks that in a very time limited consultation, GPs may or may not utilise.

Software developer staff 1
And I try and teach GPs that, so just again, I get, I haven't got five minute. It's like, I have no time [inaudible 0:18:03]. You know, and there's a patient perception that they've come in and if they see a GP typing, they're not listening, then patients get annoyed. And you can understand that, it's like, hang on, I'm here, stop looking in your computer.

Software developer staff 1
R: But I like the fact that it appears at the very beginning of prescribing, not at the end. So if they've started a product, they don't have to go through putting a dose and a quantity, they'll appear before all of that. I like the fact that we reference everything. We do not provide any messaging, apart from a cost swap, and then you don't need evidence because it's like that's cheaper than that and it's the same product.

I: That is the evidence.

R: That is the evidence, yeah. And often, that's localised and we keep up to date with it. And we have a suite of people in (name of place), to do that, so I think, yeah.

Software developer staff 1
We took some of the learnings from that in (GP CLINICAL SYSTEM) in that we put everything on one page, so there's less to click. And it would be nice to be able to go back and do that with System One. But that is…as I said, we can give them all the tools to do that but they need to do that their end. And that is our sticking block. So we get a lot of grief from GPs saying it doesn't look any different. And it's like we'd love it to look different and we've got all of your feedback, but we can't do anything about it, that's System One. We do encourage the end users to vote on System One and (GP clinical system), there are vote buttons that we would like (the CDS) to do this. So if we can get everyone on (the CDS) voting, it becomes a higher priority for System One, but it's getting prescribers to do that.

Software developer staff 2

R: Yeah. There are probably some changes we would like to make with how (the CDS) is displayed, to make it a little bit more simpler in terms of the [inaudible 0:41:55] GPs use. That requires vendor involvement, so we can never commit to any timescales on those. And unsustainability, again I think a lot of it is driven by the content, keeping on never forgetting who the end user is and what their requirements are. Although [inaudible 0:42:21] end user requirements, ie the GP can sometimes be different to the CCG requirement, and that's a conversation we often have. Some might like to have an alert for almost everything, and that's not…

I: And others would like to have no alerts.

R: Yeah. The GPs want no alerts. And there's that balance to hit the sweet spot really.

Software developer staff 3

R: Well I mean, well again this is where it's a complicated question. A lot of it was to do with the way it was designed, and how we made those decisions around the design. So, how did we present the options to [inaudible 00:20:34] users, so a lot of this is down to how you do mock ups or wireframes, and how you start to ask those really pernickety questions about, should this button be here, should it be here? What does this do, what do this? So, getting that kind of feedback in that initial stage is very helpful, in terms of design. To be fair, we didn't crack it, because you don't always get these things right. And I guess, some of the problems we've faced, once we've got wide user feedback, saying, this needs to change or, that needs to change, then convincing the vendors, GP vendors, to make a change, is actually quite difficult.

I: Yeah, quite.

R: So I think whilst one of our partners may be very responsive, there's definitely another one which hasn't made a change to their workflow in over two or three years.

I: Yeah.

R: So, in some ways, that implementation has got drawbacks, because it's not up to date in the UI we would want it to have. And it's a problem we've faced previously, it's not dissimilar to the problem we had with our core CDS, and there the vendors just did what they did, in (the CDS) we've given a blueprint which they've stuck to, but then updating that blueprint is difficult. Some of the newer stuff we're trying to do, going forward, we're actually taking an approach, potentially, where actually the vendor actually gives us a bit of real estate, which we're able to configure and update as required, which is actually a much more sensible approach.

Software developer staff 3

I: Yeah right. Because in some respects, anything that's going to interrupt what the GP is doing, is going to be necessarily, it's something, a GP's now got to do something different, or a prescriber has got to do something different. So, I suppose, that's the challenge isn't it, it's finding the ways to do that, that…?

R: And we haven't cracked it, in any means. Even if you take something like antimicrobials, and trying to make sure people are prescribing the right antibiotic, which is aligned to local formulary for the right condition. The problem is, as a clinician, when you go, and you don't think about the data entry side, you see someone who's got a chest infection, you examine them, you take a history, it sounds like they've got a chest infection, stick them on amoxycillin et cetera. But the problem is, what they do is they stick in amoxycillin, without putting any of the supporting information. So you're not able to then, that supporting information is actually really important, because it guides our decision support to some extent.

I: Yeah, absolutely.

R: And so, there are other examples of workflow where actually you need the right data to be inputted, for (the CDS) to work, and it's something where we're kind of looking at, not necessarily for a new product, but in terms of the modules, or in terms of areas where it could be improved.

Software developer staff 3

R: I guess the other piece is thinking about who the prescribers are, or the different types of prescribers, or people within the practice who need different types of information. Right now, (the CDS) focuses very much on the GP prescriber, who has a certain requirement for certain types of messages, certain types of information. But actually, if you take something like drug monitoring for instance, when someone prescribes something, at point of care, telling them that they need to do a three month TFT or LFT, or whatever check, it's not necessarily the best place to tell them, 'cause in their head it's three months away, do you want me to do this now? Well if you do want them to do it now, you need to make the workflow much more easy for them to do that. So, can they get one click, where they populate their order entry screen, and it's done? And right now, the technology or the ability to do that, isn't there. But another way of skinning the cat is, can you present that information to a different person? So, if you got more pharmacists working in practice, or if you got pharmacists text from a support perspective, can you start surfacing those requirements around drug monitoring, or TDM, to a different person who's more likely to action it? So, I think that's where we're trying to push the needle a little, with Optimise, and probably with the audit tool that we're now building, it's really trying to understand some of those use cases, within the way a practice works, and how the information, there's a wealth of information that needs to be served up, but actually how do we start focusing it to the right person, and [inaudible 00:41:54] in the workflow that matters to them?

Software developer staff 3

R: And then the feeling was we also needed to make sure the workflow, and how clinicians interacted with [voices overlap 00:04:40], more from us, to say, this is how that information should be presented, and how it needs to be recorded, et cetera. So, we started having much more ambitious conversations with our primary care vendor partners, to explore how we put, essentially, our vision of the solution into the system. So, as you may know, when a user uses (the CDS) the look and feel of it is very much similar to their system that they're using. […] But we have given the vendors much more of a clearer description of what that may look like, so there's been a lot of partnership working between us and the vendors, and then users, to say, this is how we want it configured, or this is how we want the UI to look. And these are the clear trigger points,

these are the clear touch points, this is the data we want to collect. Which is very different from the approach that we had in place, from a Multilex, or our old CDS offering.

CCG Staff

CCG Pharmacist 2

R: I think part of it was just getting a better insight, in terms of what their processes at (the CDS) were, like how they decide what they would include in the message and what they wouldn't and why and what the purpose of the messages are. So I think I mentioned a little bit earlier and what I was saying was about if an alert pops up, what is it that you're trying to…? Are you trying to nudge them into a different direction or nudging them towards the national guidance and can that briefly be put in there or is it…? They don't really want to duplicate obviously what's already in the clinical system and I can understand that. I think this is more of a clinical system issue probably more than (the CDS). A lot of the alerts and messages, whether they're pop-up ones or not, sometimes they're warning alerts within the system, they're not sometimes very easy to see or they get drowned out by other messages within the same page or they're a bit further down, especially with System One. I think the way it works, depending on the patient's history, if they've got a load of history in terms of clinical history as well as drugs, a lot of stuff sometimes gets pushed down the screen. So things like contraindications or drug interact…you know, all those sort of things that you might want to just double-check and stuff and precautions, things like that that people have then said, oh, we don't see those and we could do with an (the CDS) message. I can see where (the CDS) are saying, it's already in the clinical system so you wouldn't want to duplicate everything.

CCG Pharmacist 2

I: Just thinking in terms of some of the stuff we said last time, we were talking last time around your role and some of the communication with and from GPs. One of the things that you mentioned was the reporting of medicines, for instance, from primary care. Is (the CDS) helping at all with that? Has (the CDS) been able to help with any of this reporting back from GPs about…or have you been able to look at the hit rates of things as well?

R: It's a good question. I haven't actually investigated any hit rates as much as maybe I could do. I don't know what it would show because I think isn't there something, I'm just trying to remember now, when it comes up… Unfortunately, see, I don't have access to the clinical systems, I've only got access to System One demo and that's not linked into (the CDS) so I can't actually see them come up and interrogate the system and see when they come up and what it looks like. So I think some people have said when an MHRA comes up I think… I can't remember what the screen looks like but you either can accept it or reject the message or something like that. I can't remember. I think some of them are rejected but I don't know what the… I think it depends on how good that data is, do you know what I mean? […] It's almost like, how good is that data? What does that actually mean if they press reject? Did they mean to or is it just because it's on the wrong side of the mouse, do you know what I mean?

I: Or it's just that it's a click, click, click, click, click to get rid of all the alerts?

R: Yes, exactly. Yes, I think when we looked at it, I might be wrong, but you know when you're on your mouse, you left click, don't you, and it's automatic. I think the left click was the reject. I can't remember.

CCG Pharmacist 2

R: No, it could be an admin person or, yeah. Interestingly, I think my own understanding of what (the CDS) messages are intended for, and how they fit in with clinical systems, has developed as time's gone on, obviously going to these meetings on Optimise, and obviously with (name) being there and explaining things, I'm like, oh I can see where you're coming from. You know, like in Prescribing Group sometimes, you're like, at least it's a quick fix, just put an (the CDS) message in. And it's like, well hang on a minute, some of this stuff is already in the clinical system, it's whether people are choosing to see it. And I think people have just become, it's become like white noise, like, especially in System One, the way it's set up, I can see, like allergies get missed, or interactions are a bit further down here, or contra-indications, depending on the history of the patient, it could be down here somewhere […] Whereas, I think (GP CLINICAL SYSTEM) is a bit more, a pop up alert comes up.

I: As a box, yeah.

R: And apparently, System One can be set up that way, but people choose not to…

I: So they don't see them.

R: Yeah, don't see it as much. So, we've had prescribers admit, that they don't always see the stuff that's down here, because they've got to scroll.

CCG Pharmacist 3

R: And it was just finding out…and because I asked so many questions, like for instance, one of the things when we were setting it up they said that was really, really good was putting things on mute. Because then you can see how often it fires. But what they didn't tell us was that slows the system down. Because it's still firing, so it's still utilising energy. You just can't see it. So it's still working in the background but it slows the system down.

CCG Pharmacist 3

So one of the things I did was yes, we have things on mute, but we made sure we only had a few. Whereas they had hundreds in (other part of the county).

I: And then you've got to look at the data on the hits on those…

R: And of course, you haven't got the time to do it.

I: Yeah, precisely, and then you've got to know whether to unmute them if you're getting lots and lots of hits. Or take them off if you…

R: Exactly. So in our implementation plan, where we were setting up the profile, because I have a little team working with me, when we were setting up the profile, our aim was to have the number of mutes. I think we're looking at teens, but at least definitely two digits. But preferably below 20, I'm not sure how many we've got now because I don't deal with the profile on a day-to-day basis. But we kept it minimal, so it was things like that that I think helped us. So we didn't have a lot of complaints of it's slowing the system down.

I: You mean slowing the clinical system down.?

R: The clinical system, yeah.

I: Yeah, because when in…because it's supposed to be seconds, isn't it, but…

R: And it takes longer, and it was knowing the things that people had complained about in the (other part of the county). So I was able to put that in from our FAQs when we sent it out to practices here, because I said to them, it will slow the system down but it's only a couple of seconds. Whereas if I'd said, no it won't, that would have been a problem. But I said it will slow it down. But you should not notice it and if you think okay, you're spending an extra 20, 30 seconds for the safety of the patient, is that worth it? Because we did actually have a GP in (other part of the county) who actually did an experiment and I think it worked out it was 30 seconds.

I: Of extra time?

R: Yeah.

I: Per patient or one in five?

R: Per patient.

I: Per patient.

R: Because he complained about it slowing down. So between him and my colleague, (Name) in (other part of the county), we actually put together an experiment to actually see how much it slowed the system down. And it worked out on average about 30 seconds. Which can be a long time.

CCG Pharmacist 4

What has been an issue is when it goes down. So we've had a couple of outages recently, and practices don't like that. So one day, afternoon, it went down for about 40 minutes and there was big delays, and obviously if it's slowing the clinical system down that does concern me I guess because you're going to potentially disenfranchise them with it.

CCG Pharmacist 5

I: I mean have there been any, sort of, technical problems and technical issues that have slowed things down or caused any problems apart from the fact that they don't change your messages very quickly?

R: I'm trying to think. There were more in (name of place) I think there were problems with like the on-call service so not everybody could get the messages. I think it depended how they logged onto the system as to whether or not they'd see the messages so that took a little bit of sorting out but (name), the account manager was very able to help with that and they produced good step-by-step guides to helping through it. I think turning it on initially in the practices was difficult for some practice staff. I think they thought it was more complicated than it actually was so I think we learned from…or I learned from (name of place) about the information that that practice might need and the support. Then when we merged in (name of place), we were able to provide that support and train up the practice pharmacist to be able to help the staff, then, to do it. So, we were able to change all of the county profiles onto the city profile in four weeks which was impressive. I think it involved a bit of bribery but, yes, we've not had many technical issues here.

CCG Pharmacist 5

R: Generally, most GPs don't complain that they get too many messages. And I think many realise that if they're getting a lot of messages perhaps they need to question whether or not they're coding their patients properly. You know, if they're actually, perhaps not up to date with what they know. But...

I: Because, if the coding is not good…

R: Yeah, if the coding isn't good they'll get more messages than they should do because all of the messages are tailored, so they're very specific messages, most of them. So, there's a logic behind them, they'll only pop up for patients with certain conditions, and if you haven't coded that condition then, or have coded the condition incorrectly, you're going to get...

I: Or if you've coded it more broadly.

R: Yeah. Or, if you're diagnosing something and you prescribe before you add the read code to the record, you're not going to get the tailored messages. So, you might get extra ones saying, well you can't prescribe that because this patient hasn't got that condition. But it's just they did it in the wrong order, so teaching [voices overlap 00:25:58].

I: Yeah, so you should rather diagnose before, diagnose the symptoms then...

R: Yeah, code it first and then prescribe.

I: Yeah. And because it's so dependent upon that, and presumably as well, if, as you say, there's a broader…if they get it wrong they just get all the messages come, because there's too many in there.

R: Yeah, you will get the generic ones.

I: The generic, precisely, yeah. It cuts out that very, very specific message for that particular patient.

<u>GP Staff</u>

GP Nurse 2

I: Do you find it changes, then, some of the way that the consultations work, when you're talking to patients and so on?

R: I think the only thing that I'm mindful of is that you spend a lot of time looking at the screen. A, you know, you do have be safe, but there's so much going on and sometimes I think…I actually did have a patient say to me, you're looking…and I can like still touch type so also I can… You do spend a lot of time looking at your screen, looking at bloods, checking that. I don't know how you'd get round that. You can't get round that, can you?

GP Pharmacist 1

I: Which is I suppose part of the shared decision-making, isn't it? Yeah. Do you find that with when you're doing…with the more…your patients or your chronic patients, or you are doing reviews? Do you find it also fits in nicely with…?

R1: Well, yeah, because it's the perfect time, isn't it? Because they know they're here because we're looking at reviewing their medication, that's often the purpose of the consultation. So that's good and then I can explain to them why, well, actually, you've been on this for a long time but now, this is what's recommended and this is why. So, you have the opportunity to explain it to them there and then, and then change it there and then if that's the right thing to do.

GP Pharmacist 1 and GP10

R2: No. There's no big change. I think one of the things that highlighted last time was that there was quite a few groups that would come up as recommend a change, nothing underneath as to what the recommendation was. Which is annoying. And those issues appear to be a lot more ironed out. I think occasionally come across one…

R1: I haven't come across any…

R2: …or two, but…

R1: …of those yet.

R2: …but not…there used to be quite a few. So, I think that's a big help. And I think then the other issue was also sometimes when you had the suggestion, this is the alternative that you could look to prescribe it, when you clicked on it, it either didn't give you the correct thing, so it wasn't directly giving the correct information…

R1: Alternatives.

R2: …there and then. And again, I haven't come across one of those since to be honest, no. Not recently, anyway.

I: So, some of those teething problems seem to have been ironed out?

R2: Yeah, definitely.

GP Pharmacist 1 and GP10

I: Yeah. 'Cause one of the things that I've also found, going round, is that sometimes it's not clear whether an alert is an (the CDS)alert or is coming out of your clinical system.

R2: Yes, true, that is true. Sometimes it's difficult to determine which is which. Yeah.

I: And are you getting lots of other alerts as well as…?

R2: So, there's a few others coming up as well, yes. I'm not sure that it is necessary to know – as an end user – which…

I: Yes, where they come from.

R2: …one it is. No, I don't think it's absolutely necessary to know that. But I must confess, it really does take a lot of effort to work out…

I: Where it's come…

R2: …which one's which. And often the (the CDS), you know only because of your next step which is [own 30:25] override, if it's the override you're going to take. Because it's the only one that offers the drop-down list of different one…yeah. So you know that [voices overlap 30:34]…

I: And it's quite well-embedded into the system.

R2: It's quite well-embedded. Yeah.

GP Pharmacist 1 and GP10

I: One of the things you said before is if it comes at the right time. Is fitting the alerts into that workflow, into what is a fairly challenging ten minute consultation or can be and so you're juggling lots of things in your head at the same time and there's lots of stuff happening around here. Is that…

R2: So, I'm fairly experienced. I prefer it at that point, if a patient leaves and you have to pick up the pieces after, it's not so good.

R1: It creates more work, isn't it?

I: Yeah.

R2: Yeah, it's not so good. It's not…

R1: [Voices overlap 32:40].

R2: …very professional, to be honest. I prefer to say, oh, I was going to give you this, but actually I've just realised – you don't actually have to say the computers have reminded you – but you can say, I've just realised that actually we should be looking at this and this.

GP Pharmacist 1 and GP10

I: Well in some respects one of the further questions is about having confidence in the system and trust, do you have confidence in it?

R2: Yes, I think so. I don't think it's turned up anything that is… It's like any programme, it's got its niggles.

R: Yes, I think very occasionally it's perhaps not kept up-to-date and things have changed locally.

R2: Yes.

R: So, I don't know how that works in terms of updating it locally. I can think of one example not that long ago when something had been considered by the APC and agreed some drug in a specific situation. The next time I went to add it, I got the (the CDS)thing telling me it was still grey when I knew it wasn't. That was a matter of days, maybe a few weeks.

I: Yes, I think there is…..

R: So, if you didn't know that you were up-to-date then it's telling you the incorrect information. I can think of one occasion when that's happened.

GP Pharmacist 2
I: Yes. But, it fires when re-authorised.

R: But, not everyone re-authorises. Depends on what your policy is in practice, as to whether you use re-authorise. If you use re-authorise, it kicks in, but if you were to just...because with (GP CLINICAL SYSTEM), you can just keep issuing repeats, like, there isn't a point where (the CDS) will kick in, (the CDS) kicks in if add a drug, or if you try and replace it with something, or switch it. Then it does, but no, if you just, on reception, just clicking issue, issue.

I: Clicking to press re-issue, re-issuing the repeat.

R: Nothing will come through.

GP Pharmacist 2
R: Well, by name definition, it's to (the CDS) your prescribing, I suppose, to make it better or more improved in some ways. Yes. And that's why, I think, clinicians are just like, well, it's not optimising my performance. If they have to stay late because of a clinical system, they begrudge that, rightly so, you know. They work quite long days when they are in. And if a patient is there and the system is whirring, you know, you don't look very professional, and that's what they are faced with. And, you know, whether it's the internet or the speed or the connection, you just don't know, but right there, it's frustrating, isn't it.

I: Yes, that it's slowing things down.

R: And, if you bring in PINCER, you know, if you think there's going to be more things added to it, is that going to slow it down even more. And then, I don't know whether clinicians can turn it off or on, but they're not supposed to.

GP Pharmacist 2
R: So, obviously, the CCG rolled it out. We have (GP CLINICAL SYSTEM), whereas most of the...now, a lot more of the practices have got (GP clinical system).

I: (GP clinical system), yes.

R: And we're planning on going to (GP clinical system) as well. So, initially, you get that whole, is it slowing the system down, you know, because, for the clinicians they don't like to be slowed down by their computer. So, there was that initial, sort of, period of time, and it, sort of...you then wonder whether it's coincided with an (GP CLINICAL SYSTEM) update or if it's the actual software. But, yes, clinicians are always going to blame the (the CDS) for the slowing down.

GP Pharmacist 2
I: Yes, so that could tailor what it was going to alert to. So, rather than the profile management being done at CCG level, what if it could be done at practice level?

R: Oh, I think that they would…

I: Do you think it would be workable, or unworkable.

R: No, they just wouldn't want anything...I've seen how the profile is, and it's hard work, to build the profile, it's too hard work. Yes, it should just be done, like, surely, I mean, you know, it's a national thing, like our prescribing, the drug tariff is a national cost, so therefore, it should just be nationally, we all do the same thing. That's what we're supposed to do, you know. So, it should be as easy at grass roots, as easy as possible, and free of complications. Yes. I mean, it would be great if it were clever enough to text patients and tell them, the NHS says no, you can't have that Cetraben anymore. You're going to have to buy it, or whatever.

GP1
I: Right. And in terms of it being put onto your system here and being used within the practice was there any sort of training, any resources attached to that?

R: I don't recall any, no; I'm not sure somebody came in and showed us how, basically it was fairly similar to the previous system and it pops when you're prescribing with information, so it didn't really take a lot of getting used to, we were fairly au fait with what was going to be happening anyway.

GP1
I: Once you've gone through that process of getting the tool here, once it was started to be used, broadly just describe how it is used by yourselves and others in the practice?

R: Well, it's used probably in virtually every consultation where a prescription is issued it will tend to [inaudible 0:09:20] it doesn't always come up when…depends on what you're prescribing; if you put paracetamol it doesn't come up.

I: It's not going to. Yes.

R: So it's used continually; and this is why it's hard to remember what it exactly does because it fades into…it's just part of the system.

GP1
I: So when it was first introduced, if you can remember back to that time, is there anything that particularly went well, or could have been better, or whatever?

R: It went very smoothly, I mean I don't recall any great problems. The only problem in that respect we've had with it, the previous system, if when you'd made a suggestion and you'd pressed that you wanted to follow that suggestion, would put the dose in and it was basically it was a one-click affair; so, you know, change from paracetamol capsules to paracetamol [voices overlap 0:03:03] paracetamol with the same dose [voices overlap 0:03:06] the (the CDS)system doesn't put the actual dosages in, which is a bit of a drawback because then it becomes it's not quite as seamless, it's…

GP2
Yes. But it's just...the system managed quite good but if somebody is doing some sort of audit or search on the system it takes the priority and the whole system slows down.

I: Right.

R: When you have this on top of it and you have a patient which is...and you need it to pull the information out of it, it slows down even more.

GP2
R: We have only ten minutes per appointment. If somebody's 85, 86 by the time they get here, they'll have already lost two minutes. Sit...another minute. You are really struggling for the time.

I: As we walked through there...it's actually a reasonable distance from where they're sitting in the waiting area to here and if you say if they're elderly... So, how long have you got to make that prescribing decision? It's not long, is it?

R: No. It's not long but it's...when everything slows down it slows down the whole process. You know what I'm going to do, and everything is there and it's just going through the clicks and...

GP4
R: It's slowing it down in the sense that it's there, it's something that wasn't there before. But I think for the benefit you get from it, the time isn't intolerable really. And I think…I've not personally had problems with it sticking, or me not being able to close it down, or it being awkward.

GP4
I: Any other positives about it in terms of the way you use it then, or any negatives?

R: I think the negatives are always just another thing that…distracting you from the patient, I suppose it depends…the whole transaction really between patients and doctors has changed throughout the last 23 years and it's just another thing that's distracting your attention. So you get…it's a newish thing for us, so you're ending up trying to prescribe something, you're trying to prescribe something smoothly and efficiently and quickly and in a way that doesn't detract from what you're talking to the patient about and then the thing pops up and you're, oh, right, sorry, oh, hang on, and you're usually…

I: Do you find that…how does that impinge on that relationship, I mean, is the patient aware that you're making those changes, or…?

R: No, not always, no, 'cause I wouldn't always vocalise that, so they might just find themselves looking at my ear for a few moments. And I'd guess they'd probably surmise that I'm…that my concentration has moved really from them directly but I guess that happens a lot if we're looking at results or we're looking at a letter from the hospital, so I suppose patients aren't particularly surprised by that really.

GP4
R: Well, I suppose…and also, it's like a confidence thing, isn't it, in that if you say, right, I'm going to prescribe you this and then you start umming and ahing about it…but actually, I do see that as a positive really. Once you get beneath that slight clunkiness that it causes, it's actually…we shouldn't be prescribing things whilst we're half chatting to patients and being distracted. We should be focused on that task. I mean, general practice you're often mentally juggling quite a lot of balls so it's quite easy to drop one. You dropping a prescribing ball is potentially very serious. So yeah, I think it's just that I…I find it relevant and I don't resent it. I think of why. I've got on with it really.

GP4
R: I was perhaps not expecting it to be as smooth as it is. We've had lots of pop-ups that we've done ourselves over the years to remind people to record things for QOF. And I don't know whether all my colleagues would agree with me but I find it quite useable, so sometimes…we were talking earlier about things coming up at the wrong time or not necessarily being relevant to what you were doing or the task that you were performing. It doesn't seem to have that, it does seem…and also you can navigate through it quite quickly.

GP4
R: So you're actually at that point when you're prescribing, so it's not popping up at a point in the consultation when it's removing your focus from something else you're supposed to be doing, it complements the activity.

I: Yeah, that's really interesting, yeah.

R: I don't find that a problem.

GP4
I think it went reasonably smoothly. I don't think there was any…there have been glitches along the way where I think System One were concerned that it might have been slowing the clinical system. But in terms of our concerns about it I suppose it was like any pop-up type system really and worrying that it was going to be intrusive or it was going to take a long time or it was going to be difficult to ignore or shut down. But in principle the sort of areas that it was going to help us with were things that we were quite interested in really, so it wasn't like some of the QOF-type stuff where it can be intrusive because it's seen somebody with a mental health problem, it's prompting you to ask about smoking data or something, it was something that was important and we felt motivated to try and improve on really, so I think we were relatively open-minded to it. So I don't remember there being any huge problems. I think I was pleasantly surprised in some ways that it first of all acted as a useful prompt but also it was reasonably easy to get out of if you decided you didn't want to proceed with whatever advice it was giving you.

GP4
How does (the CDS) fit within your workflow? How does it fit into what you're doing in terms of the consultation?

R: I think it fits in reasonably smoothly. I mean, we're wedded to the system, so having something which comes…and we're all increasingly used to using templates and opening up different boxes and things like that. But because it only really seems to appear when you're actually doing the prescribing task it seems to fit reasonably smoothly.

GP4
R: We haven't really talked about it on a practice level actually, which would be a quite interesting thing to do. I mean, I think it's been useful. So I definitely see a benefit from a person…

I: Anecdotally that you mentioned earlier. Yeah. And in terms of patient outcome do you think that's the…as well as perhaps the costs of it?

R: I think it must do, yeah, I think it must do, I mean, it's…actually what would be quite interesting would be to write…I mean, one thing before you came, I was thinking I need to try and – in the day or two before you came – just 'cause I was thinking about it, I thought I need to try and think of or try and notice examples of when it's come up because I think actually I've probably just accommodated to it, it's in…

GP5
R1: We got told, that …'cause we had something else before that, I've forgotten the name of it. But, we were told that this was an alternative. It was better. We would find it more useful. It was better integrated into the (GP CLINICAL SYSTEM) system. But it was similar to what we already had anyway, and so we shouldn't, besides the screens looking a little bit different, it shouldn't really impact on our workflows or anything like that. And essentially, by and large, they were right. It had a few little extra bangs and whistles, particularly I think, to connecting to links to websites, if we wanted to know more, which seemed a bit more better integrated, so we had all the functionality of previously, plus a bit more, and it was a bit tidier, and a bit more Windows.

GP4
R: We haven't really talked about it on a practice level actually, which would be a quite interesting thing to do. I mean, I think it's been useful. So I definitely see a benefit from a person…

I: Anecdotally that you mentioned earlier. Yeah. And in terms of patient outcome do you think that's the…as well as perhaps the costs of it?

R: I think it must do, yeah, I think it must do, I mean, it's…actually what would be quite interesting would be to write…I mean, one thing before you came, I was thinking I need to try and – in the day or two before you came – just 'cause I was thinking about it, I thought I need to try and think of or try and notice examples of when it's come up because I think actually I've probably just accommodated to it, it's in…

I: If it's in the background?

GP4
R: So I think this doesn't seem to have those problems as far as I've seen, so far. But I think one thing that might be interesting would be actually almost to jot down over the course of a day or two every time one's popped up so you can remind yourself of what sort of things you were prompted about and how useful it was. Because otherwise it's just one of many stimuli that happen…

GP4
I: One of the things you talked about last time was, you know, whether the system was over intrusive or whether the prompts were useful and things like that, what's your feelings now, a bit further down the line, about them?
R: I don't find them too intrusive really. I mean it is another thing that happens that didn't happen before but it's as we discussed before, it was they are relatively easy to override and the suggestions that are being made generally feel quite sensible. So, you know if it's a cost change that you think, well it's not that important and you can override it very easy. And if it's a safety thing, then generally I've been quite pleased that it's happened because it's given me an opportunity to make a change before potentially something that might cause me a problem later down the line or caused a patient a problem later down the line.
I: Yeah, quite.
R: I don't mind it really. So, two observations I've got really, one is that when you're invited to make a brand substitution or some sort of formulary change, it can be quite difficult to find the suggestions. Other doctors have commented on that before. So, in a way it sort of makes it difficult for you to do something positive. You'd say, right okay, yeah, that's a fair suggestion I'm happy for you to now give me a recommendation. If you then click on show suggestions, they don't always come up without you ticking a very small box in the corner – and I can't remember what it

says – but you sometimes open up the suggestions and it's just got a blank box, and then you have to tick a very small box in the corner which then brings up the options.

I: Oh I'm with you, yeah.

R: I'm certain that sometimes what people have done is, they've opened up that box and gone, oh well there's nothing there, they don't know how to access it, so they override it and carrying on with their work. That was one bit of feedback. It would have been easier to generate suggestions which you could click on very quickly and action.

GP4

R: I'm generally not aware of it until it pops up and then when it does pop up it doesn't appear to slow anything down particularly. So, it's like any other pop up really, that we've got some of our own that have just been set at practice level, so if you are trying to prescribe PPI for example, it reminds you, have you considered sending this patient for a helicobacter test, type of thing, which is just a practice level thing rather than something that has come down.

GP4

I think it went reasonably smoothly. I don't think there was any…there have been glitches along the way where I think System One were concerned that it might have been slowing the clinical system. But in terms of our concerns about it I suppose it was like any pop-up type system really and worrying that it was going to be intrusive or it was going to take a long time or it was going to be difficult to ignore or shut down.

GP5

R1: So it's rare when it comes on. But, that's because, I think it's when you're adding new drugs. So, I think, the reception staff, when they're using it, with new patients joining, they're possibly seeing more of it. It's rare for it to come, for me, it's when I'm initiating a tablet.

I: Yeah. Yes, it shouldn't fire on repeat.

R1: Exactly. So, it's not annoying, let's put it that way. If every time I was doing a prescription, I kept getting Optimise, that would annoy me.

GP5

R1: We got told, that …'cause we had something else before that, I've forgotten the name of it. But, we were told that this was an alternative. It was better. We would find it more useful. It was better integrated into the (GP CLINICAL SYSTEM) system. But it was similar to what we already had anyway, and so we shouldn't, besides the screens looking a little bit different, it shouldn't really impact on our workflows or anything like that. And essentially, by and large, they were right. It had a few little extra bangs and whistles, particularly I think, to connecting to links to websites, if we wanted to know more, which seemed a bit more better integrated, so we had all the functionality of previously, plus a bit more, and it was a bit tidier, and a bit more Windows.

GP6

R: Yes and our screens are very, very busy as well and I think that it's fair to say that what's happening now with (the CDS)probably is, that while all the changes to the generic stuff is very obvious pops up and it's clear, what I'm probably then not doing is acting when it pops up with alerts because I think you just become blind. I think there are that many things that pop up and I think if I'm honest, it's only very recently I've even noticed there are red things flashing up on it going something about, what everything says torsades de pointes so everybody ignores those. But I don't think I have ever actually read one of the alerts.

I: Right so you basically...

R: I think I just delete, I just ignore them.

GP6

R: Oh no the cost one, I mean, that's what's so funny, the cost ones I think we've been doing for so long because we used to have ScriptSwitch and very genuinely sometimes there are decent savings to be made and it makes sense and I don't give a stuff. You know, that's okay and if I quite like it being a branded alternative as long as they keep getting the same brand, it doesn't matter to me but the alerts bother me because they don't all fit on one screen.

I: Right okay yes.

R: So half of the important messages that are coming up probably roll off the bottom so I can't even see them anyway when they pop up and there's just too much of it, too many of them, bing, zoom, there's just loads of it.

GP6

R: I think, yes probably because the human brain is so clever, I probably do actually pick out a something if it's actually relevant and useful and I think that's partly why this torsades de pointes business has been a problem because it's fugging them all up now.

I: Right.

R: It almost appears on every single alert. So that would then switch you off necessarily to [inaudible 19:00] everything else but I think probably, it's a weird thing but probably anything that's in the middle of the screen actually does make more of an impact in your mind than anything else.

GP6

R: And when you actually are prescribing things, some things are black and some things are grey and I don't think any of us has a clue what that actually means.

GP6

R: Yes my brain works fast but the system in some ways is too slow and irritable because there are so many things that are chucked at it now and the referring, I couldn't even refer people this morning because everything is so slow. So I think that is partly a real issue that all these bolt-ons are making it worse.

I: Slow down. and it's too much.

R: And for the prescribing, you see when you're going through, when we've got our repeat prescriptions to attend to each day, that come through electronically, you can't function when everything is this slow. But every single prescription that you go to then sign, the ones that are allocated to me, could you work on a system that's like this.
I: It's so, that, yes, well, no.
R: It's just clunky and ridiculous but these are the ones that are waiting for me to be signing today but if there are any of those, sort of, alerts as well, all of them need reviewing and things. There are just too many things.
I: Yes.
R: Right I think I've made my point!

GP6
R: Yes and our screens are very, very busy as well and I think that it's fair to say that what's happening now with (the CDS)probably is, that while all the changes to the generic stuff is very obvious pops up and it's clear, what I'm probably then not doing is acting when it pops up with alerts because I think you just become blind. I think there are that many things that pop up and I think if I'm honest, it's only very recently I've even noticed there are red things flashing up on it going something about, what everything says torsades de pointes so everybody ignores those. But I don't think I have ever actually read one of the alerts.

GP6
R: And when you actually are prescribing things, some things are black and some things are grey and I don't think any of us has a clue what that actually means.

GP7 and GP8
R3: And so then it had dawned on me, I don't know how it happened, I clicked the box and they appeared and it was like a sort of a light bulb, oh they're the ones I should be prescribing rather than the ones I'm doing. So then I realised but that should almost be automatic.

R2: Automatic, it should be there and you…

R3: That would have been great. And the other, the other thing that slows you down is if you don't want to select one of them you have to over-rule or over-rule with a reason. So again that's another step that you have to do.

GP7, GP8 and GP9
I: Are you getting a lot, I mean you said it sort of it uses every prescription, well not every prescription but when you're getting those multiple alerts what's, is that, does that, I mean how does that affect the workflow and in your consultation?

R1: It just slows you down a bit and just makes you just think, huff and puff but there's nothing you can do. It makes you frustrated but you still have to go through it.

R2: It can be a bit frustrating I think. Like yesterday I was trying to do a prescription and I kept accidentally clicking the wrong things. Because I was basically trying to do an HRT prescription, move it onto a different type of HRT and it was saying, it was grey and it didn't want me to prescribe that one. So then I was trying to go back out of it to look at what they'd previously been on to see what the alternatives were and I couldn't close it and it was just going round and round in circles but I was obviously just pressing the wrong thing but it was annoying.

R3: You try to shut down and it's not over.

R2: I was trying to shut down all these boxes and they were going if you're doing this you need to do this and then it was wiping the drug that I'd put in and…

R3: With regard to HRT there does seem to be a problem because they are coming up greyed.

R2: They are, yeah, I've noticed that. I've asked them, but I don't think it's all of the HRTs because actually when I looked at this particular one, it's not on our formulary. So it was to do with the fact that it's not on our formulary, but I don't know why?

Because what it actually said was, there is insufficient evidence of this drug. It is a grey drug and I thought, well there isn't because it's a formula of HRT, a formula, which is perfectly reasonable and the alternative that you're telling me I can prescribe is basically exactly the same thing with a different brand name. So I think it's actually a cost saving switch not a grey lacking in evidence.

R3: Because I've had that same problem…

R2: With HRT, I think that is the problem.

R1: Yeah, there's a couple of them isn't there?

R2: But it's, you know, it's a few extra clicks but I do think it helps with safety and I do…

R1: I don't think it helps as much.

R2: …think it will be saving loads of money.

R3 And I suppose the rationale is over time you should be getting fewer and fewer clicks because you remember…

R2: To not prescribe that thing.

R3: Whatever, yeah.

GP7, GP8 and GP9

R3: Because it was a bit of an "oh my goodness" this box pops up and I know for me originally, I'm not very IT savvy I couldn't work out where the alternatives were.

R1: I don't think that's very obvious still.

R3: So it said there were alternatives.

R2: But you have to tick on the box don't you, it's not, it should just appear straightaway.

R3: And you have to reveal the alternatives and for a while, I think maybe a month or two, I carried on doing what I was doing.

R1: I didn't realise for ages the alternatives were there.

R2: It would make more sense to do the alternatives without having to do that little extra click on the box…

GP8

R2: I think in terms of the safety ones like the one, the thing where it comes up with the amber, you know, this is amber two or it should only be prescribed after…like a secondary pair or whatever, that can be useful because, because occasionally you accidentally go to prescribe something that you maybe realise that you shouldn't be. And I don't know, I don't know this, but does it save what you've put so it wouldn't come up again next time?

I: For the individual patient, yeah.

R2: Because if, for example today I did a prescription for [inaudible - 0:06:16] on the cardiologist's advice and it came up with it saying it should be specialist initiated. So, I put specialist initiated. So I don't know whether that would be saved and it wouldn't do it again…

GP8 and GP9

I: So that's switches and that. You said it's very, it was fairly straight forward because it was, it was there.

R2: It's just there.

I: And it's just there in the system. So how do you, each of you feel about the way you're sort of using it. So are you using it as it pops up a lot? Do you get lots of alerts or…?

R1: You don't get a choice to not use it. It pops up every single time. Even if it's something simple pretty much.

I: So you get alerts pretty much every time you prescribe?

R1: A lot of drugs. It's not every drug you prescribe, but obviously if there was a switch or if it was an amber drug or something like that it comes up. So it can feel like it's quite a lot and it can feel like it's adding a lot more clicks to try and do a prescription sometimes.

R2: I think yeah, if you're trying to give something as a one-off to somebody who's on lots of medication I think that's when a lot of the time it will feel like there's always a pop up, because obviously there's lots of interactions with other things to be aware of.

I: Yes, quite, yeah.

R2: Whereas with someone who is not on medication it won't pop up because there's nothing to interact with.

GP8

I: And in terms of numbers of alerts and things, it's been said to me before that there's a, there's a difference when you are dealing with something like (the CDS)as to your patient who is sitting in front of you. So a patient, to make the comparison, or a patient, one off acute prescription, compared to a patient who is already on, you know, [inaudible - 0:15:33] on complex poly-pharmacy and on something like 20 – 30 medications, as sort of those two extreme examples. Have you found, you know, in terms of alerts, any difference in the way alerts come up or in the way you deal with those alerts with those sort of two extreme examples as it were?

R2: I think the only think I would say on that is that it's a lot easier if we're trying to be persuaded to prescribe less, so if (the CDS)is coming up saying we recommend the patient buys this over the counter. It is a lot easier to say that on an acute than going back on something that people have had prescribed for years. So when people have had their anti-histamines and nose sprays prescribed for years and to go to re-authorise them and it suddenly says they need to buy it over the counter, people take that less well I think than if it's an acute prescription and you say, oh no, we can't prescribe this, you need to go and buy it.

GP9

R: It won't then go on… So for Instillagel you can ignore it but it won't go on to put Instillagel in the prescription. It changes it to Lidocaine so then you have to delete the box and then type in Instillagel again and then find it that way. It just creates an extra step or two which as soon as I press ignore shouldn't actually come up.

I: Yes, so what you want is to be able to go…

R: If I ignore it then just give me what I want.
I: Yes, basically, and not have to have that extra bit.
R: Yes.
I: So are you finding, therefore, it's interrupting your consultations?
R: It doesn't interrupt my patient face-to-face consultation, it just interrupts the speed at which I can complete before I go onto my next patient so it's just interrupting that side of things.

GP9
I: Yes, precisely. So that's interesting, isn't it, because it's, sort of… Is that a negative thing, then, that slowing things down, [voices overlapping 03:28]?
R: Yes, I'd say so. For me, personally, and I guess for most people who are obviously time-pressured anyway anything that slows you down or stops what you've always been used to just gets in the way really or becomes more of a hindrance.

GP9
I: Are you seeing any sort of benefit then because I mean last time that we talked to you, you know, it seemed to be the benefits might be around safety but there were some benefits around cost. Are you seeing any benefits now or do you think it should be there? Is it a good thing to have?
R: Well, if it's linked to the formulary then, yes, I think it's a good thing to have. If it's linked to my local formulary it will still remind you of some of the cheaper alternatives out there so in that sense, yes.
I: Yes, but it's still interruptive.
R: Yes, it still is interruptive. There are some things which it interrupts too much or there's two or three sections that pop up when you think, well, why just not put it in one section?

GP9
I: Yes, or are you thinking, actually, I don't think that's right? So is it, I disagree with the alert or is it just it's not suited for that patient?
R: Usually it's either not suitable for that patient and even if you disagree with the alert, sometimes that's the easiest option to tick to get it out of the system, yes.

GP9
R: I still see it more as cost-saving.
I: Right.
R: I think the safety thing comes up on a different screen prior to that.
I: Right, which system are you on, (GP CLINICAL SYSTEM)?
R: (GP clinical system).
I: (GP clinical system), right, okay.
R: So the safety bit comes up when you actually prescribe it, not the (the CDS)bit so I use (the CDS)as the cost-saving bit.
I: Right, so you're not seeing safety messages in Optimise.
R: I think there are a few, aren't there, sometimes with statins and clarithromycins so, yes, but…
I: Yes, but by and large you're seeing…
R: They've come before anyway.

GP9
I: Yes, just going right back to the beginning and you talked about what you do, responding to it and you sometimes find it disruptive or find it, you know, the extra clicks, bit of extra time and so on. You said there's more alerts, do you find, you know, the whole thing, is it really disruptive or is it just part and parcel of the way things…?
R: It's part and parcel of my work so when I say disruptive I mean in the sense that sometimes it's just making my consultation longer but it's still part and parcel of my work.

GP10
I: So, it is look. You don't look like you're moving out of your clinical system. 'Cause it's interesting 'cause one of the things that I think is a consideration is that if other systems…your clinical system and other things are banging out lots of other alerts, it's not just…then that can be the smoke that hides the important (the CDS) alert coming out as well, isn't it? So, it's perhaps the balance within all of that as well. It's just important.
R2: Actually, I haven't seen that. Yeah, I can believe that it could be a potential problem but in reality, in two…a couple of years, I've never seen it. Yeah, it's quite clear. Yeah. And even where you get a couple of alerts, you move through one, the other one will appear shortly after that. It's very rare that that's happened, but where it has it's usually actually from a…and I don't know which one was which but looking at it from a slightly different angle if you like, rather than… Yeah, I'm trying to think of a very good example. It's only very rarely happens. No, I can't think. But yeah, it hasn't…I think it's potentially more of a problem than it is in reality, yeah.

GP10
R2: I think…I suppose final thing is is that when it initially came, it did appear to be…I'm quite Luddite, so yeah, just bear with me on this thing. But equally I do embrace change. It did appear quite clunky and quite intrusive. It's become significantly less, so whatever the reasons, I suppose it's very hard to say, and got to the point where it strikes quite a nice balance in my opinion between giving me enough information regarding safety and patient education and cost-effectiveness. Again, it's just getting on with the work that I've got to do, i.e. I've got a set amount of work in that day, so I don't want to be spending just my time [voices overlap 36:18].

GP 11
R: Yes. So, I suppose my personal experience has been a bit appealing, you know, when we try to prescribe things. It might have been that before I joined as a relatively late member, that there had been training on it, but from my point of view it was more of a...

I: It was just there in the system.

R: Exactly, yes. You'd try and do it and the things get promoted. So, that happened.

GP12

R: Yes. Well, I mean, the big change is that we changed from one (GP CLINICAL SYSTEM) to another (GP clinical system), so that happened. So we migrated in August, so that was a massive change to our whole computer system, to how everything looked like on the computer screen, to how we were dealing with things. And that took, it took quite a while for us to sort out prescriptions and prescribing drugs, a lot more clicks on (GP clinical system), a lot more complicated.

So the whole prescribing area has been a massive change and took us about, you know, I'd say two to three months before we really worked out the prescribing system on (GP clinical system) because it's just been completely different from (GP CLINICAL SYSTEM) . I think unexpectedly different, and much more convoluted and much more complicated.

I: Right. In what ways has that impacted on the (the CDS) alerts then?

R: Well, I think what's interesting is that it was difficult to... Whereas before, I knew what was in (the CDS)because when (the CDS)was introduced it was obviously new. So we knew, oh, that's (the CDS) as opposed to the rest of (GP CLINICAL SYSTEM). Whereas actually what happened in (GP clinical system), for me, and I suspect my colleagues, is that actually we didn't recognise the difference, recognise what was (the CDS) because everything else was new.

So it felt like (the CDS) was just another box that was part of (GP clinical system) that we were trying to negotiate. And I only recently, and that's after four months, you know, of being, where are we now? August, September, October, November, December, yeah, three to four months it took me to actually recognise what was Optimise. So I thought that was just another box that we had to go through in (GP clinical system). I think it's possibly because of the way that (the CDS) looks in (GP CLINICAL SYSTEM) is it kind of looks very different.

So it kind of stood out and maybe I recognised it as different. Whereas actually in (the CDS) in the way that it appears in (GP clinical system) is very similar to (GP clinical system). It's the same sort of smaller type, same sort of background, and I just thought it was just another thing to negotiate around in (GP clinical system).

GP12

R: Well, it just means that because we've been trying to survive (GP clinical system)... When I first saw the box, I actually had a nurse standing with me who had been used to (GP clinical system) before and I was kind of, oh, you know, oh, what's this, sort of, thing. I didn't know it was (the CDS)and she said, oh, what you do with that is, I think she tried to get me to prescribe a nurse's dressing or something like that. The box that came up, I remember it was the first time I saw the box, and I didn't know what it was. I just thought it's another bit of (GP clinical system) to negotiate through in order to do the prescription and she just, oh, what you just do is you just override all oh, you 'proceed with original override, all' those two buttons. In my head I was proceed with original, override all, when I see this box. So for ages I was actually proceed with original, overriding all, as just another click through (GP clinical system).

I: In which case you were overriding all the alerts?

R: I was overriding all the alerts, that's right, yeah.

GP12

R: Whereas actually in (GP CLINICAL SYSTEM) it was much better presented. Somehow it just looks simpler. It was the tight spacing, the whole issue that it was a smaller box. (the CDS) is written in a bigger type face. You knew what it was. It just seemed to be, I don't know whether there were less buttons but it just seemed that in the (GP clinical system) version of Optimise, it just seems so much, you know, so much more potential for different buttons that you're expected to kind of get right. But actually, you just don't have that time when you've got a patient with you and the patient is talking to you. They're telling you about their next problem while you're trying to prescribe their last drug that they want. And it's just, it's all I can do to remember, override or, you know, just two buttons to get through quickly before, you know, while it's going on.

GP12

I: Last time we talked, one of the things that you focused upon was that from your perception (the CDS)was very much a cost saving package, because (GP CLINICAL SYSTEM) was sending a lot of the safety alerts, now that you're on (GP clinical system), is that the same sort of thing you see or you're just not seeing them because of all this?

R: Do you know, to be honest with you, I've absolutely no idea what I'm seeing.

I: Right. And is this getting used to the system or do you think this is a going to be...?

R: A long-term thing?

GP12

R: It is, it is, it is. This is during the consultation that we're tending to see (the CDS)because I don't see (the CDS)when I do, it's very rare that I seem to see (the CDS)when we're doing the prescriptions because it seems to work out that what happens is that prescriptions in (GP clinical system) come to you attached to a task. What happened when we started in (GP clinical system), and practices don't realise this because they've got used to, you know, they haven't got the (GP clinical system) off the shelf brand new. When you got (GP clinical system) brand new off the shelf, every prescription comes in a task and it comes in a task to a particular person. So we were getting about 30 tasks and we had to un-attach the prescription to a task and do things to it and sign it and it was just ridiculous paperwork [voices overlap 10:19]

I: So this is on reauthorisations and...?

R: Yeah, and it was just removing it from the task and doing what needed to be done. And then we couldn't work out what were other practices doing because, clearly, they weren't taking two hours to get through their prescriptions or whatever. So then we worked out that what other practices were doing is they use a prescription clerk to remove the task, the prescription from the task, and then put it neatly in a box where all you have to do is go down very quickly, go approve, approve, approve, approve. No (the CDS), no nothing comes up. You just approve this batch load of prescriptions that could be about, I don't know, 40 of everybody's. You hardly have, you don't see them, you hardly see the drugs. It's a bit like the old, here's a load of prescriptions in a basket, sign them all for the day. It feels a bit like that on a computer. So we've worked out that we now do all that. So the only time that you would therefore see an alert is either if you get a task to say meds overdue. And I don't see many (the CDS) on meds overdue partly because I think our repeat meds are probably not too bad in terms of our prescribing, what we prescribe and cost effectiveness and stuff like that are not too bad, so we don't see it then. So the most likely time you're going to see an (the CDS)alert now is with a patient in front of you when they request something funny or you're prescribing something acutely.

GP13
Yeah, so we thought we'd give it a go. We had reservations that I guess everybody had, would it slow down work, would it create difficulties, would it crash, would it run? But, we thought we'd give it a go, and it just became part of life then. Initially, without really any problems at all, and we were all a bit surprised how easy it was. Then there was a time when I think it got a bit clunkier. I guess as it developed, it became very slow and it did slow things down a lot, and we were…

I: It slowed other systems down, or slowed the consultation down?

R1: Not consultations, because I think the way I use it, I don't often use it in consultations, because I suppose that's one of the problems of it, I don't know if you want me to speak about the problems, but the time it crops up in workflows most, is when you're signing today's 60 repeat prescriptions, so the patient's not in front of you, but there's a medication review overdue, everything's been done, the patient's had their blood pressure, had everything done, so you don't want to get the patient back, so you can update the review date, and then (the CDS)flashes up and tells you that something minor could need changing, and you sort of let it go. But there was a time, maybe about a year ago, when we were seriously thinking of saying, we don't want this anymore, because every time medication was issued, it was obviously checking five or six times, and it was slowing down repeat prescribing quite a lot. And then that just seemed to disappear, so I guess that was part of an update process, so it was an IT thing. And then it sort of became much more like it had been at the start, which is almost not there at all, except when you need it, which sort of makes it feel more like it what I imagine it was always meant to be.

I: Running in the background, sort of thing, yeah?

R1: You said, mostly, you see it when you, you were saying around re-authorisations and repeats, are you seeing it when you're going to prescribe with a patient, do you see it when the patients are in front of you sometimes?

GP13
I: Yes. What about acute prescriptions, do you ever see it firing now?

R1: Yes, it fires sometimes on acute prescriptions. I'm trying to think of an example recently, 'cause it doesn't happen that often, and it isn't that intrusive. As I think about it, I wonder if it's actually too non-intrusive, because it might be too easy to dismiss without acting on it. In terms of choosing formulary stuff, so drugs that are on formulary, I have a habit of checking the formulary regularly, because it changes so often, that often…

I: They meet once every two months or something, don't they, or something like that?

R1: Yeah. And so I have a habit of checking the formulary quite regularly anyway. Particularly if it's something I don't prescribe very often, so it's already on formulary, so I guess (the CDS)wouldn't flag up anything other than safety issues at that point, and then (GP CLINICAL SYSTEM) flags up many safety issues anyway.

GP13
R1: And I can't tell you, if the prescriptions I signed before that audit, it had flagged up and I'd ignored it, or it had not flagged up at all. I just don't know, because it's now so good, in terms of not bothering unless it needs to, but you don't notice it if it doesn't.

I: Yeah.

R1: Yeah, there might still be that.

I: And you wouldn't see those things unless there's those sort of audits.

R1: Exactly yeah. There must be some sort of analogy with type one and type two error, the type one errors are the things it makes you do when it flashes up, and the type two errors are things you never know.

I: You never know about, yeah.

R1: Because it doesn't flash up.

GP14I: What are those challenges prescribing wise?
R: So, prescribing wise, if patients are on long lists of medication already, you know, sometimes our (the CDS) will flash up saying, there are over 40 current medications to check against, we can't do it, which is never a good sign. So, we have lots of patients who are on already long lists of medications. Perhaps they've already tried various other medications as well and other challenges if they're under secondary care or other clinics elsewhere.
Often we get letters that ask us to prescribe things that are perhaps beyond our expertise and might be a little bit more expensive. For example, just because they've got complex medical needs, we have lots of kids with intractable epilepsies and PKU and lots of, lots of odd prescribing just because that's our patient demographic, we just seem to have lots of complex patients.
I: Odd complicate…odd mixes as it were of that case.
R: Yeah, very much so.
I: So, do you find then, the next step on that is in what way is (the CDS)then useful or, isn't it, is it useful in those circumstances?
R: It's still useful because it will check your medication you're prescribing against, as long as they haven't got over 40. medications on there and others.
I: They should raise the figure [voices overlap 07:49].
R: Yeah. I've got a couple of patients and it always flashes up when I try and prescribe something.

I: So, it stays over 40 but you…
R: Over 40, can't check them all, yeah. So, yeah it doesn't devalue it essentially.

Sub-theme: 5iii (the CDS) and the context of General Practice work - spaces, people, roles

Software developer staff

Software developer staff 1
Yeah. So my background is, before coming to First Databank, was working for another decision support company used within practices. That was very different in that it was an executable file added onto clinical systems and it couldn't integrate with patient records.

Software developer staff 1
And so that was probably going back almost two and a half, three years ago, and since then the tool itself has developed. Sadly, not so much within the clinical system, more within the tooling itself, in that sort of the portal people log…you know, their customers can log into, the ability with what they can do with the messages within the portal. For prescribers themselves, they've seen a couple of updates, more so within (GP CLINICAL SYSTEM), it looks a lot cleaner. I think when the first iteration came through, they didn't know whether it was an (the CDS) message or a clinical safety message. (the CDS)wasn't quite clear on that. And so they never knew whether it was one of our messages or something from System One or from (GP CLINICAL SYSTEM).

Software developer staff 1
So I've seen probably more development from a user end rather than from an end user - using the software end. We are limited on that respect because we rely on the clinical system providers to support us developing that side, they have lots of other priorities. So I think no one will really know this this but there's something called SNOMED, which is a new way of READ coding, and that on everyone's priority. So if we say, oh, can we change this in the box, that is not a huge priority for System One, SNOMED and everything is.

Software developer staff 1
We took some of the learnings from that in (GP CLINICAL SYSTEM) in that we put everything on one page, so there's less to click. And it would be nice to be able to go back and do that with System One. But that is…as I said, we can give them all the tools to do that but they need to do that their end. And that is our sticking block. So we get a lot of grief from GPs saying it doesn't look any different. And it's like we'd love it to look different and we've got all of your feedback, but we can't do anything about it, that's System One. We do encourage the end users to vote on System One and (GP clinical system), there are vote buttons that we would like (the CDS)to do this. So if we can get everyone on (the CDS) voting, it becomes a higher priority for System One, but it's getting prescribers to do that. So I think from the other end, so for the CCG, the purchase of, they've seen a huge development from how to enable to messages, how to run reports, and now giving them the flexibility to go in and alter messages themselves without having to go through our content team. And even in the last 18 months, that development has really rolled on. Where I was before, it was crazy because that was our own piece of software, so really we could have done any development requests. And I was with that company for six years and even after six years, we hadn't delivered the one thing that people wanted, which is the ability to have coloured text, for instance. So it's been really good for me to come to a company that we try and listen and development. Our sticking block is not our end, it's our partners' end.

Software developer staff 1
I: And you don't work with Vision.

R: No. So that's going to be interesting. We want to work with Vision and Vision said they want to work with us. The trouble is, is that Vision, I don't know what's going on at Vision, they're trying to develop more of a mobile platform. And because of that, it meant that the legacy bit of Vision has had no support or development, and so the new Vision has sort of taken a little bit of a backburner, but their priorities are not to work with us to develop…what we said to them I think, even when we had these talks with them two years ago, was let us build the tool into the old version at least. And they were like, no, because that's going to retire. Now, of course, that's not going to be retired now 'til potentially next year, so we could have been in there for the last couple of years, which is really frustrating for us. But they've got their own issues I think around development and costings. So that's going to be…I don't know how that's going to play out.

Software developer staff 2
R: Yeah. So my interpretation of this is that (the CDS)will continue to be really useful for initiation of drugs, probably less useful at reauthorisation. But we get some, but it's more hit and miss and will vary at a practice level how they work. But Analyse has really been developed to support the emerging practice pharmacist role who are likely to take on a lot of the repeat maintenance of prescribing within a practice. So we want them to be complimentary. That's the goal. But yes, we will have to see how they kind of play together if you like, and that's one of the objectives of the early adopter phase.

CCG Staff

CCG Pharmacist 2
R: Yeah, so some surgeries, and perhaps probably most surgeries, will say to the reception staff, if that pops up, you're not to accept it, because a lot of it, it needs a clinician to accept it, so they might just put, like, a full stop on the rejection thing or… They don't actually indicate that they're not clinicians, and I might ask them to start typing that in, if they're not a clinician, because then I can see how many people are not clinicians and see in in that information, or even just a code to put in, so I can pick it up.

I: It's an interesting thing, because I've heard this before. That you might get someone, when the repeats are being re-authorised or something like that, and it might just be a question of administrative staff, obviously because they've got to go into the record, in doing that. And, as you say, it might be useful to have that, so you know who's…

R: Yeah, a lot of them will take screenshots and print it off and give it to the GP for them to look at later, but then that rejection box has gone and that data's come back.

I: Yes, quite. So you don't get that reason for rejection?

R: Yeah, so we don't always, and I'm sure there will be surgeries that the non-clinical staff will just ignore it and not put a reason, because there's a lot of blanks when they...

CCG Pharmacist 2
I: That's good, yeah. That's, and how, has that, I mean in terms of making changes that's helped but how has that helped, and in changing the profile, sort of more broadly how has that helped with the system do you think, in terms of, you know, what's happening with the GPs and so on? It's use in practice?

R: With having these reviews do you mean? […] So basically I'm due one next month and they'll look at our profile and maybe we'll go through things that seem to be working, which they can, you can run reports yourself but they'll do all that for me because I just don't have the time at the moment. And they will show me which ones don't seem to be useful, because no one is really accepting them. Or they'll show us, so bring forward some ideas of what we could maybe change it, how it, and maybe that would make it, the uptake might improve, or whether it's just something that just doesn't, that simply doesn't work so we need to address it in a different way, as in sort of like a project within the surgery, where (the CDS)wouldn't be the platform to use it. For example, like there was a [inaudible - 0:08:16] modified release tablet and we would say to change it to the standard release because there's, that is safe to do that, plus there's a cost saving for this particular one. But having that on (the CDS)is not helpful because if that, anybody logs into (GP CLINICAL SYSTEM), sees that message. So it could be someone on reception, it could be, you know, someone who can't make that clinical decision but also you can't just change someone's blood pressure medication without having monitoring put in place. So it could, it's actually better that the technician flags a list of patients to the GP or practice nurse, or practice-based pharmacist and they can speak to the patient, change the medication and also invite them in at that point.

CCG Pharmacist 2
I: You said that it's not the ultimate solution, so is it just a small part of medicine safety or is it, is that what you're saying?

R: No, I think it does play a big, well I don't know what to…

I: A part, yeah.

R: A part of it but it's very difficult to measure because everybody has different views of technology. It might, anything that pops up on the screen might irritate someone. I would find that hard to know who that would be because some of them might just ignore any pop ups and just, because they know best or whatever. They might not fill in the feedback of why they're rejecting things. So it is quite hard to see how useful it is but I do think it does play a part in this, yeah, definitely.

CCG Pharmacist 2
Or equally, it has to integrate well with the existing systems, I think that's the key thing, so it's an add on to, and it tries to enhance the system that's already there, I think. I think it's more clinical, rather than practical, operational, but I think there's lots of things that are in the clinical systems that get missed, that prescribers could see, but they're not necessarily seeing, depending on how it's displayed. So, that's unfortunate.

CCG Pharmacist 2
R: No, it could be an admin person or, yeah. Interestingly, I think my own understanding of what (the CDS) messages are intended for, and how they fit in with clinical systems, has developed as time's gone on, obviously going to these meetings on Optimise, and obviously with (name) being there and explaining things, I'm like, oh I can see where you're coming from. You know, like in Prescribing Group sometimes, you're like, at least it's a quick fix, just put an (the CDS) message in. And it's like, well hang on a minute, some of this stuff is already in the clinical system, it's whether people are choosing to see it. And I think people have just become, it's become like white noise, like, especially in System One, the way it's set up, I can see, like allergies get missed, or interactions are a bit further down here, or contra-indications, depending on the history of the patient, it could be down here somewhere.

I: Yes.

R: Whereas, I think (GP CLINICAL SYSTEM) is a bit more, a pop up alert comes up.

I: As a box, yeah.

R: And apparently, System One can be set up that way, but people choose not to…

I: So they don't see them.

R: Yeah, don't see it as much.

CCG Pharmacist 2
R: One thing that did come up as a bit of a concern, and it's been in the back of my mind, and it keeps coming to the forefront, and I'm like, oh I've forgot about that, but there was an issue once where, and it wasn't (the CDS)'s fault necessarily, but it was the way it integrated with the clinical system. So, the example was, I think it was azithromycin, I can't remember which was around it was, it was meant to be switched from capsule to tablet for example, because it's more cost effective. So, it did do, the System One did do that, 'cause (the CDS) said, you might want to do this, it's cheaper or something, it did do it, but what ended up happening, was the dose then changed to the default for that tablet, in System One, so it ended up being the wrong dose, and frequency. […] And then that then raised an issue about, oh, oh, are there any other drugs that this could have happened to, is it significant if the dose is changed, do the doses change for those drugs in System One? And in some cases they did or didn't. So then, a lot of work had to be done with System One, I think (the CDS) really did help with that, I think, getting patient safety sort of hats on, and saying, how can you change this in System One, to make it, so another alert had to come up to say, prescriber, do you want to maintain

the original dose you put in, or, do you want it to go to the default dose, that is now changed to, and which is this? So, at least it's alerting them, 'cause if you don't know at all, and you've just assumed, it's carried across, yeah.

CCG Pharmacist 2
R: Yeah, and I think what I found interesting was that (GP CLINICAL SYSTEM) are quite picky and choosy about what they want, so if you've got this little e with a little slash through, you know that message is not on (GP CLINICAL SYSTEM), because they have their own. And that's what I mean about (GP CLINICAL SYSTEM) being a bit more, I think they're a bit more intuitive, they're a bit more proactive, in terms of their messaging, compared to System One.

CCG Pharmacist 2
R: Yeah, so some surgeries, and perhaps probably most surgeries, will say to the reception staff, if that pops up, you're not to accept it, because a lot of it, it needs a clinician to accept it, so they might just put, like, a full stop on the rejection thing or... They don't actually indicate that they're not clinicians, and I might ask them to start typing that in, if they're not a clinician, because then I can see how many people are not clinicians and see in in that information, or even just a code to put in, so I can pick it up.

I: It's an interesting thing, because I've heard this before. That you might get someone, when the repeats are being re-authorised or something like that, and it might just be a question of administrative staff, obviously because they've got to go into the record, in doing that. And, as you say, it might be useful to have that, so you know who's…

R: Yeah, a lot of them will take screenshots and print it off and give it to the GP for them to look at later, but then that rejection box has gone and that data's come back.

I: Yes, quite. So you don't get that reason for rejection?

R: Yeah, so we don't always, and I'm sure there will be surgeries that the non-clinical staff will just ignore it and not put a reason, because there's a lot of blanks when they...

CCG Pharmacist 2
Before (the CDS)came in, it was like, shall we think about having a protocol in System One, or do we just want this to go out as a key massage, via our Medicines Management Team? So, they have that face to face conversation with the practices. Or, it could just be as simple as, we'll add it to the next newsletter, we've got like a Derbyshire Medicines Management newsletter, that goes out.

CCG Pharmacist 3
There were a couple of practices that we had problems with but they have not switched it off, and theirs was about who generates the prescription. It should only be a prescriber who generates the prescription. Some practices have a system in place whereby they have an admin person generating prescriptions.

I: Would that be on repeats or just…?

R: Not repeats because it only fires for acute and new. So admin people should only be doing repeats, generating prescriptions for repeats. They shouldn't be generating prescriptions for an acute drug, and they shouldn't be generating a prescription for a new drug. Okay? Apparently in some practices they do and that's the way they work. Whatever safety they have in place I don't know but as I said, we cannot condone that behaviour, so we cannot sanction it to say, oh, we know that you're doing it and because of that we're going to try and alter (the CDS)in some way or the other. It's not supposed to be done. It's your system, it's your loss because we activated it only for prescribers. We didn't activate it for admin people.

CCG Pharmacist 3
It's the different systems they use and I know it goes on, so it's not unique. And they might have prescribing clerks…

I: Right, I'm with you, yes.

R: …generating prescriptions, but they are not prescribers.

I: Yeah, precisely.

R: So they should not generate a prescription but what's happening is in some cases, they generate the prescription and they give it to the doctor to sign. And they should only be doing that for repeats. They shouldn't be doing it for new scripts. So let's say for instance somebody's had something previously and it was on as acute, just once. Somebody phones up and says, oh, they want that same item again. Now, the whole point of putting it for acute is that at that time, they could not make a decision as to whether it needed to be continued. So therefore they need to see the prescriber again to see if it needs to be continued. If it needs to be continued, then they can put it onto repeat. It's not up to the prescribing clerk to make that decision and there's always the likelihood that they can generate the prescription and the doctor just signs it without checking. But then the prescriber is 100 per cent liable. So it is a problem, and it's not unique to us. I know it goes on. But if we've put a safety mechanism in place to help them, and they decide to bypass it…

I: And it doesn't generate…(the CDS)doesn't fire when you go to do a repeat prescription…

R: No. it doesn't. Which is fine. Which is what we would want because if it fired… Because what happens is that you put on a new prescription, you have all the relevant messages pop up. So that's fine. You put on a new repeat, you have the messages pop up. But let's say for instance you've put on something for six months and for instance you've got a medication that needs to have a blood test every six months…

I: That would then [inaudible 31:12]…

R: …okay? It's not going to fire every month because you don't need it. You don't need the blood test for six months.

I: Yes, quite.

R: And as a rule, it is non-medical staff that would generate an issue, a reissue or a fulfilment of a repeat. If it came up then, it would be very annoying because first and foremost the non-medical staff would not be able to make a decision……as to whether they should accept it or not accept it. But the risk with accepting it means that they could accept something which could be unsafe. The risk with not accepting it is that the prescriber now doesn't see a relevant message. But if you've given somebody something for six months, unless somebody comes back and says they've had a severe reaction or it's contraindicated, you've taken that decision at that time that it's safe for this person to have it for six months. So you do not need to be presented with the same message month after month after month.

I: Yes, precisely.

R: And that would not have got engagement because that would have slowed down their systems immensely. And they

<u>GP Staff</u>

GP Nurse 1

I: So going into the GP, so when you come into a practice like this you go into the GP system and use the clinical record from the GP?

R: I will normally use the community module but like, for example, today I've been to a care home and there's a few repeat items they were asking about, and so I'll go into the GP module and then look at the prescribing history. And then if, for example, they'll say there's not enough medication to last the 28 days I will go into that repeat template and change it from there, from the GP module, rather than do it from the community module because it gets confusing. So I have to work with both and so when it comes to...when I do an acute medication, like a chest infection, I prescribe antibiotics, I'll use my community module because that's what other people working for the community trust would do. So I have to try and work out which is the best way of doing it for communication.

GP Nurse 1

I: Right. So I was thinking there so you're, if you like, in between secondary and primary in some respects, aren't you?

R: Yes, I kind of made myself that way because I'm trying to integrate with the surgery and use my role, and then I have to think about the best way of...and the community trust know this because I talk with my managers because they would say, you only can use a community module. And I'd say, but I need to use a GP module when I'm doing this and it's all about the patient.

GP Nurse 2

R: Yes, but I think for probably a new prescriber or someone that hasn't got the confidence, it's like [inaudible 13:50] trying to prescribe some of the more regular stuff that you know is safe, it comes up the selected product is brown. That's to do, again, with our meds management and the way they've probably got it set up but that's to veer you away from prescribing the more slightly expensive ones, I guess, isn't it?

I: Yes, and with those reviews as well, I mean do you see lots of alerts or is it as you're going through a review?

R: I don't seem to see too many when I'm doing a medication review, no.

I: Right, okay, and do you do those reviews with the patient there?

R: Yes.

I: Yes, so it's a, well, I don't know what level you call that but there is, isn't there?

R: It's more a case of going through and saying, you know, do you know what this is for [voices overlapping 14:44]?

I: Yes.

R: Yes, but if you just actually click on the medication review at the top of the medications then nothing comes up, actually, so it depends which way you do it. If you just click on the review date and then the re-authorise restart, nothing clicks up.

GP1

R: It's quite a long time ago, I think it was through the medicines optimisation. We had a previous software, I can't remember what else it was called any more; it was another CDS.

I: Yeah, another sort of CDS system.

R: The guys had looked at Optimise; I think it was cheaper, and that they felt it was giving us what…I think there'd been a problem with the other system, that they weren't updating it brilliantly; they'd put the price up and then they were wanting a commitment that they weren't willing to make, so they looked at the (the CDS)system and decided it was pretty much the same. I think, I can't remember now, there was one very positive thing in its favour as well, which I can't remember what that was now.

GP1

I: And who uses it within the practice? Is it just prescribers, or other people as well?

R: I think it pops up for some of the repeats in the office, but I don't know, because I'm not there when that happens, and I have in passing noticed it; I'm not sure they actually respond to it though, because they haven't got the authority to. But I mean possibly (name), one of the practice managers, deputy practice manager, who does a lot of prescribing, can respond to it, [voices overlap 0:02:09] the suggestions it makes he would pass to us to make the final decision [voices overlap 0:02:15].

GP4

What if you're getting a lot of prompts for particularly…I'm thinking perhaps with patients with more complicated multiple conditions, multiple morbidity, do you find you're getting lots of different prompts for the same patient, lots of messages coming up or…?

R: Not in a troublesome way, that's not a feeling I would have had if you'd have asked me just to think of problems. It may be the case, and I think sometimes what happens in those cases, I sometimes just shove them down, I think, well, I'll make a judgment, I suppose if it's something that really jumps out to me as being really significant or important, then it'll draw my attention to it, but sometimes you think, well, this is a patient on ten or more medications, somebody along the way has considered these things, they've had a medication review, today I'm concentrating on this particular problem. So you're trusting to some extent in the system and sometimes you just don't have enough mental space or physical time to actually go back and to act on all those things, so sometimes we park things a bit, and it's a longer term project I suppose. So that…in those circumstances the prompts are about giving you a heads-up about something that might be dangerous. But some of the lower tier things I might just shove down. And think, well, I'll have to deal with that another day.

GP5
R1: We got told, that …'cause we had something else before that, I've forgotten the name of it. But, we were told that this was an alternative. It was better. We would find it more useful. It was better integrated into the (GP CLINICAL SYSTEM) system. But it was similar to what we already had anyway, and so we shouldn't, besides the screens looking a little bit different, it shouldn't really impact on our workflows or anything like that. And essentially, by and large, they were right. It had a few little extra bangs and whistles, particularly I think, to connecting to links to websites, if we wanted to know more, which seemed a bit more better integrated, so we had all the functionality of previously, plus a bit more, and it was a bit tidier, and a bit more Windows.

GP8
I: Actually thinking about that, since we're talked about PINCER, do you think it's better to have the sort of pop up message and make the change there and then at the point of prescribing, or is it better to have the PINCER approach where you've, if anything slips through the net the pharmacist looks at it, comes to you and says, oh you need to think about this broad range you've prescribed, or you need to think about this or these things?

R2: I think you probably need a bit of both. Because I think the (the CDS) is really good because it stops you doing it at the time or it makes you think about it at the time and really question what you're doing. But I don't think for example if somebody over-rode that and put something on a repeat prescription for 12 months that that would necessarily get picked up when the receptionists issued a medication. Because I don't think they get the Optimise, they won't get the (the CDS) box popping up when they just…

I: Well it shouldn't come up on repeats, it shouldn't.

R2: So if something has slipped through the net or if somebody prescribed something and it hadn't been on (the CDS) at that time or something like that, it's not going to be picked up potentially until the medication review and then it depends on how careful people are when they are doing medication reviews, which is variable isn't it across everybody how they're done and what you think of at that time.

Sub-theme: 5iv. Receiving too many alerts in practice

Software developer staff

Software developer staff 1
The actual usability hasn't changed much for prescribers. That's the only, I think, downfall is that we've not been able to make it slicker for prescribers. The often complaint is there's too many clicks. And that's because, one, we all say, oh, there's a…because we'll have a button for, do you want to see the reference information, or do you want to read all the text, do you want to see the alternate products. And these are all clicks that in a very time limited consultation, GPs may or may not utilise.

Software developer staff 1
I: Yeah, that's good. Are there…I mean, you said the READ coding which in some respects it's not a part of the system, the system requires some READ coding, but then so does lots of things require accurate READ coding. But do you think there are any limitations and difficulties within the system?

R: I would say, yeah, too many clicks is a huge issue, and that's what I hear a lot, you know, it's sort of…especially in System One, it's all expanding. So as soon as they go to prescribe, bumf, this huge box appears with all of this text. And then there's almost too many buttons on there. Because at the top, you've got…you know, there's about six buttons and it's sort of to try and…all you want to know is click proceed or okay or reject and then reject with a reason.

And now we've got the ability to see what I've now called the quit count, because you can either accept or reject, or the third option is to ignore it and just close the box, bumf, I'm not even going to read it. And we can pull that data off now. For some CCGs, that quit count is huge. I mean, we've got a new customer on and we're going out to…me and (name) – oh, sorry, I shouldn't say – another account manager - are going out to go and see them at the end of the month. Because we always recommend customers, especially if they've not had any decision support before, to go very small. This organisation has turned everything on and not even looked at what they've turned on, they've got duplicates. Oh, it's just a nightmare. So we're having to go and help and sort those guys out. But you just think, wow, that GP has never seen anything before, he's now having multiple pop-ups of sometimes saying almost saying the same thing, and their quit count is huge. You don't want that for a new customer…

Software developer staff 1
I: And I was at a meeting recently where I was talking about our evaluation, and someone, I think it was a GP, said "too many alerts". And that's…is that something you hear or is that…?

R:	It's a difficult one because there's not only our alerts, there are the inbuilt CDS. So (GP CLINICAL SYSTEM) have obviously a whole lot of alerts as well, and we will then…so there'll probably be a CDS alert, then we'll appear and they'll just be like, bloody hell, it's another chuffing box. Obviously, if it's in (GP CLINICAL SYSTEM), our message will not be the same as what their message is, at least that's, I think…again, if they knew that, it's just like, oh, you know, so they don't ignore it. One of…you can always…sometimes counter argue that if they'd prescribed the correct thing, then they wouldn't see an alert, which really gets their goat up. And you can…

I:	I can imagine, yeah.

Software developer staff 2
I:	Well, GPs almost by nature, they like something brief, to the point, concise. It was something I think that came out of the user group, I think someone was saying about that, that the change in the wording was going to help.
R:	Yeah. But I guess in terms of nationally have acceptance rates gone up? They've not. They've kind of levelled. But there's a big driver for that in that this year a lot of CCGs have enabled self-care messages, the messages that say you shouldn't be prescribing [inaudible 0:26:34], you shouldn't be prescribing pain killers, they should be going to the pharmacy and buying it. Now, we produced a suite of messages in response to NHS England, and we had a lot of customer pressure to do that. We were always concerned that these were going to fire indiscriminately, even though we've brought in as much logic as we can think of to try and rule out patients for whom that wouldn't be appropriate. So, CCGs who have enabled those, the message there as a whole only gets accepted around ten per cent of the time. There is variation in that. So that is kind of dragging down their overall acceptance rate. CCGs that don't have these enabled, generally have a higher overall acceptance rate. Again, as account managers, we'll often highlight that, and these can account for 15, 25 per cent of the messages being seen. It's huge. It's like 30 messages are counting for up to a third of the hit count. So we're always trying to make CCGs aware of the decisions that they're making and how it's influencing… We get a whole spectrum. We get the well, this is really important, we're being beaten at the head by NHS England, we've got to do it, to well, we take a more pragmatic view and we're cycling the messages and… Because we know there's an alert fatigue. But then that creates, oh well does that mean if the message doesn't fire, it's okay to prescribe it [inaudible 0:28:15] complex.

Software developer staff 3
R:	Yeah, I mean that's a difficult question to answer, so from my personal perspective, I think there's kind of two elements to it. For me, as a clinician, how can I be supported to, by technology, to make decisions which are truly useful to a patient's care?

I:	Yeah.

R:	And, the problem we have is, there's a lot of alerts and pop ups and noise, as you know, that are part and parcel of a vendor's system, that really don't make it easy for clinicians to do the job they have to do, in the time they have, and not make them frustrated or annoyed, by what's occurring. So, I think for me, and I sometimes when we brought it out, it was aspirational in a way, but some of the stuff we were doing around the best practice, suggesting what you should be doing, or what you should be stopping, or, in this situation for this patient, this is the most appropriate therapy. If we could start doing the right things, and so building a base of trust with GPs, so that when they see those alerts, they're more likely to trust them, and to read them and to follow them, for me, has always been the thing we should be focusing on.

CCG Staff
CCG Pharmacist 2
I don't know about (the CDS) specifically, but I know that there were a lot of concerns when we were thinking of adding protocols or things, to System One, about alert fatigue. And I think alert fatigue has been picked up in an incident that happened a few years ago, that was quite a serious one, and it was almost a bit like, the prescriber was like, yeah, I just sort of tend to ignore them. And it was like, so actually an alert did come up that could have prevented something, but…

I:	So, the prescriber had had too many alerts, or they felt they had?

R:	Not at that point, maybe, but generally, they have a lot of alerts or whatever it is. It was a general statement about alerts and fatigue, yeah.

CCG Pharmacist 2
R:	No, it could be an admin person or, yeah. Interestingly, I think my own understanding of what (the CDS) messages are intended for, and how they fit in with clinical systems, has developed as time's gone on, obviously going to these meetings on Optimise, and obviously with (name) being there and explaining things, I'm like, oh I can see where you're coming from. You know, like in Prescribing Group sometimes, you're like, at least it's a quick fix, just put an (the CDS) message in. And it's like, well hang on a minute, some of this stuff is already in the clinical system, it's whether people are choosing to see it. And I think people have just become, it's become like white noise, like, especially in System One, the way it's set up, I can see, like allergies get missed, or interactions are a bit further down here, or contra-indications, depending on the history of the patient, it could be down here somewhere […] Whereas, I think (GP CLINICAL SYSTEM) is a bit more, a pop up alert comes up […] So, we've had prescribers admit, that they don't always see the stuff that's down here, because they've got to scroll.

CCG Pharmacist 2
I:	Yes, I'm wondering, one of the things around the…it's, sort of, where I was coming from there was around the alert fatigue. The other side of the alert fatigue is that if GPs are getting more confident around…feel better about getting the (the CDS)alerts if they're relevant and timely and they're thinking, oh, yes, that's helped me there, does that then actually make them feel more…? Is that being reflected in GPs being more happy to report back to you or to be more accepting of alerts because they're getting those relevant ones?

R:	I think there was a survey done locally about what GPs thoughts were on (the CDS) and I think the feedback that came back ranged from, we don't like it to, yes, it's great, but the average was in the middle when you looked at the number crunching and stuff. One of the questions was, what do you think of the safety alerts and, again, taking the mean average, it came up to about 5.3 on a scale of 1-10 or something like that […] you do have some GP practices that love it and then you've got some that are like, oh, we don't like it and we'd like to switch it off if we could. Then you get some that like, the example in the morphine case, the Zomorph case, you do then get some GPs that say, oh, can we get an (the CDS)

message to try and help fix this? So they will try and…they'll remember it and go, oh, that might be a good way of…you know, so they do think about it when it comes to safety stuff and try and…. Usually via our team, they'll say, oh, can we have an (the CDS) message for this? It is considered.

CCG Pharmacist 2
R: Yes, so in some ways I think the safety messages…I don't know, there's maybe some vigilant GPs who would read them more often than not but I think a lot of the messages have been like cost related ones.

I: Yes, the switches.

R: Then not long ago we had a lot of self-care related messages and I think some of them had to be taken down because they just felt like alert overload. They were coming up quite a lot for your regular items that they probably would see on a…which says a lot, actually, maybe that they were prescribing those things more frequently than they thought they were. So the basic things where we're trying to encourage people to buy their own products like over the counter.

I: Yes, so ibuprofen, paracetamol, sort of thing.

R: Yes [voices overlapping 21:41].

I: Things that people can get over the counter, yes.

R: Like I say, they had to get rid of some of those messages because it got a bit too much apparently, so we had to pick and choose which ones to put on.

CCG Pharmacist 4
R: So we have perhaps got fewer messages enabled than other CCGs, but certainly now…I've not looked at south Derbyshire's recently, but certainly Erewash's hit rate is only about five per cent. So when people do moan, and they don't moan a lot about it, it's only one in 20 consultations. So that's two or three consultations a day you're going to get a message. And actually if you're getting more than that then there might be something you need to think about with prescribing. Certainly some practices and some GPs say they never see it because they probably don't need it because their prescribing's good. So sitting there and watching our prescribing is pretty good, so I think that does explain again why the hit rate's quite…

CCG Pharmacist 6 CCG Data analyst
I: Yeah, so you've got the...

R1: Or muted.

I: Or muted, yes, so you know what they would have fired, which is handy.

R2: But the highest percentage that we get is... I think that they said that the offer rate they wanted to look sort of six/seven/eight per cent, nothing more than that. They don't want a high offer rate. If you've got a higher offer rate, we've got to mute some of those messages, it turns people off.

R1: You've got too many messages.

I: Because you've too many messages going out?

R2: Yeah, and then they'll stop and they'll start ignoring.

CCG Pharmacist 6 CCG Data analyst
R1: I think we've been really, really mindful of not having too many messages.

R2: Yeah.

R1: So we don't get message fatigue.

R2: But wasn't that the problem that we had with Script Switch though?

R1: Yeah.

R2: That, again, it was too many, so people just start turning it off. So again, so some months, when we were running the Script Switch savings, we had two or three practices where there was nothing.

R1: Yeah.

R2: And when you asked them, it was, oh, we turned it off because they were just firing all the time.

CCG Pharmacist 6 CCG Data analyst
R2: And I think from working across obviously all six of the CCGs as well, I think that has been obviously a lot more noticeable now than it was before with the other system that we used. There were practices that were just turning it off because there were too many that were firing all

the time. Whereas now, like I say, and the average, as I say, for the CCGs is around the same and nobody is sort of over six per cent for the offer rate. And again, that's obviously what (the CDS)are saying, is, sort of, like I say, six/seven/eight, so you don't want any more than that, because then it does start to affect everything else.

I: So if it's firing, basically, you're going to get a message come up, on six per cent of prescriptions, which is manageable, rather than every time you go to prescribe something.

R2: Yeah, absolutely, and I think, again, if you had one that was firing all the time, then again, you'd have to sort of look at tailoring that and obviously again agreeing the [voices overlap 0:15:01].

CCG Pharmacist 6 CCG Data analyst

I: And the other one, finally to finish off with, is somebody said to me, and I'm going to paraphrase this, something along the lines of: the safety messages I like, they help me, the cost-savings don't, they annoy me. So again, is that something you see reflected in practices and do you think that is something which is...? How do you...? It might be somewhere else.

R1: So my answer to that would be: so it depends on the GPs and it depends on GPs... I think, again, it goes back to our GPs are quite engaged with supporting the CCGs in their prescribing budgets. So I'd like to think that most of them are quite happy to accept the switch and they just trust that our profile is up-to-date and correct and happy with our profile. Because it's saving the NHS money at the end of the day, and that's how we kind of sell it, is the fact that actually you're saving the NHS money and there will be more money for other things. If you can just accept that message, because it's a simple switch, but it's whether it's their priority or not, and that's the difference I think. Is the fact that it might not be a priority for them, but from a CCG point of view, obviously money is. But it's got to serve both.

<u>GP Staff</u>

GP Nurse 2

R: They can be a little bit annoying and I will reject some. So, for example, for the progesterone-only pill I know that although they are like-for-like but actually from feedback from patients, there is a slight difference. So I will just overrule it but it makes you think, oh, actually if I don't need to… otherwise you would just keep using the same one. It does remind you of cost-effectiveness and guidelines and stuff.

GP Pharmacist 1 and GP10

I: I mean I talked [inaudible 28:18] in some respects, do you think that's a view shared across primary care? Do you think other, you know, the routine use of this across primary care would be something that would be accept…? Do you think other practices would be, sort of, like, you know…? Is it something when you have a conversation…?

R: I think that's a GP question, isn't it?

R2: Yes.

I: Do you think GPs like having lots of things telling them what to do?

R2: Oh, dear, dear, dear. It doesn't bother me.

R: Well, I was going to say I…

R2: I know it doesn't bother my partner too much. I think perhaps it bothers her a little bit more than me but I can see that it would bother some people.

I: That there would be…yes.

R: Yes, so I was going to say, you can't do a blanket answer to that, really, can you?

I: No.

R2: I can see that it would bother some.

GP Pharmacist 1 and GP10

I: Is that pop up a good thing? I mean it's a good thing because then you see it but it…

R: I sometimes find it annoying, I have to say.

R2: Yes, and I do [inaudible 26:50]. You do, don't you, because you think, oh, I've got to…

R: You think you've thought it through and you've got your plan and then it's just like, oh, no.

R2: Throws the googly ball.

R: Yes, that's a tricky one, isn't it?

R2: I think it's still appropriate only at prescription rather than…

R: Yes, I can't see how else you would do it.

GP Pharmacist 3

I: There's too many alerts.
R: Correct, yeah.
I: Is that something you've found?
R: Yeah. Especially when I'm trying to do a polypharmacy medication review, I've got ten minutes to go through 15 items.
I: Yeah.
R: Yeah. I can probably do some of the major switches but then I really do need to sit in front of the patient for 20 minutes, which is what is recommended for a level three medication review in practice, to go through each one and make sure they are happy to go for it.
I: Yeah.
R: So, yeah, sometimes we just can't action them all. Or I think of what our clinicians would do is just ignore it all.
I: Yeah.
R: Because if the patient is stable on what they've had for the past 12 months, why change it and add extra complication to your consultations, when they are coming two weeks later to say, I've ended up with this new tablet, what's it for.

GP Pharmacist 3

I: Do you think that's something that is shared across the practice or do you think people would use it differently, or is, I mean you said about people just switching off that, would be just ignoring those.
R: Yeah. The clinicians here are very diligent in the work that they do, but it was just discussed a couple of days ago, that they do follow software recommendations, yeah, and they do find in general that recommendations when accepted it's good. In previous practices I've worked, I think the messages were just plainly ignored.
I: Yeah.
R: Because the clinician really just wants to crack on with his day and get on with it.
I: Yeah, is that a time consideration then?
R: It's time consideration, yeah. Especially when GPs have got a very complex patient in front of them, presenting for one issue but it's quite a complex consultation, they've already made their prescribing decision in their mind, I don't really want to be railroaded by a piece of software telling him to do something else.

GP Pharmacist 3

I: Part of that would be, you talked earlier about pop up fatigue or alert fatigue. Do you, are those alerts coming too frequently or are there too many of them or too few?
R: I think it's well balanced actually on the whole, yeah. From (the CDS) it's fine, I think once…it's actually (GP clinical system) software will also punch up its own pop ups regarding adverse drug reactions and interactions.
I: Yeah.
R: With every single drug that's coming up, even if it's just saying basic messages, monitor Us and Es, monitor LFTs whatever. If you've got 15 drugs, you've probably clicked off about 20 messages before (the CDS) has had its go as well.

GP1

R: Yeah. And I think the amount of warnings almost numbs you to it a little bit because you haven't the time, you simply couldn't read through every single thing that was up there, so you glance at it; and it does – I think again this (GP CLINICAL SYSTEM) – has the high severity warnings, medium and low; so I think, thinking about it I think (the CDS) is more about the cost effectiveness than the safety a lot of the time. I may be wrong in that.

GP1

R: Yes, I think it probably does marginally increase the time you spend on things, because, as I said, the actual changeover process is a little bit fiddly, I don't know if it would [mean 0:12:48] if you accept something…yeah, it just ticks it but it doesn't…it brings the product up but you've then got to put in the dosage, it doesn't transfer the dosage from what you had originally put in; so that's one thing that's worse [from 0:13:07] the previous system which did transfer. So yeah, paracetamol capsules, two qds, you have to put the two qds in, in Optimise, even though it's the same chemical you're giving out; whereas the previous system would have entered [voices overlap 0:13:25]…

I: It would have changed that?

R: Changed that dose, where it was a correct thing to do. [Certain 0:13:31] formulations where you would give a different dose, so like for like it would change it for you, that's an extra click, sometimes an extra looking it up, which when you're in a rush you'd probably just dismiss it and give the original product.

I: Right. So if you've got those extra clicks to do that's going to…

R: It puts you off.

I: It puts you off it?

R: Yeah.

I: Because that was also something that's been put to me, it's the number of clicks.

R: Yeah. I mean it all sounds very petty, an extra couple of clicks, but magnified over a week by sort of 40, 50 patients a day, an extra couple of clicks with each one is a finite amount of time. And a click, then it takes a little bit of time to translate onto something [voices overlap 0:14:22]…

GP1

R: People think you're being petty but it's a finite part of your day that it takes up, and when something goes wrong, even longer, when you don't get [voices overlap 0:14:53]…

I: When you don't get it; yes.

R: It doesn't [translate 0:14:56], that it actually can be a right nuisance, and then you'd tend to just go, oh, forget it, and go back to the original product.

GP1
I: So how often would you be doing that then? How frequently would you be sort of giving up on it, as it were?

R: If it's something like Arjun ear drops you'd give up on it, you don't bother, because for a penny I'm not going to get involved now, you just dismiss it, you don't accept it. Where there's a significant saving, or [we're not really…don't have 0:15:34] a safety risk, it's more the savings, for these sort of things. Yeah, if it says this is five quid cheaper a week, then you're going to, you know, it's significant that [voices overlap 0:15:48] because we measure how much we spend as well, so you'd probably go through it; but again, depending on whereabouts in your… [if 0:15:54] you've got an hour wait, and people are getting annoyed out there; then I would tend to ignore it full stop because you're just trying to get people in and out as quick as you can.

GP1
I: Therefore because of that what's the impact upon the practice as a whole has it had? Has it changed things in many ways?

R: It must have done, but I wouldn't know, other than myself how it had changed; we don't meet to discuss this; we might sometimes air a grudge about it, that sort of thing, why is it popping up to save us a penny, it's ridiculous, and we'll go on to the prescribing adviser about it, and occasionally it'll get changed then somewhere down the feedback mechanism.

I: Yes. Do you get an opportunity to feed back centrally then to the CCG?

R: We don't seem to get them specifically for that; we do have them, because we have a pharmacy adviser who pops in and does some work in here, so if we've got something to moan about we'll go and tell him and he'll say, oh, yeah, I'll have a word with them. I don't know if he does. But, to be fair, it's much more likely that this doesn't seem to be causing a great deal of problem in that respect, so as I say, the previous system I was always moaning about things it was bringing up, saying why is it telling us to write Piriton instead of chlorphenamine, it's taking me longer to get rid of it than the penny it's saving, that sort of thing. This one doesn't seem to be quite as much of that; or we're just getting used to it. I think sometimes you just give up bothering to mention things because it takes you longer to mention them than it benefits you, so you don't, you despair.

GP1
I: I mean that just brings us straight onto that; where do you think that balance is then between the sort of cost saving and the best-practice ones?

R: I would say at the moment it's probably more the best-practice one, there are numerous warnings come up whenever you prescribe anything; which again sometimes I'm sure when you're five patients behind it just gets clicked through. It is anonymous, this, isn't it?

I: Yes. Definitely.

R: Because some of the warnings are too…basically that it's giving warnings for virtually everything we prescribe, it doesn't necessarily give an idea of how common these problems are.

GP2
R: It's popping up for example if you do the test page in here...test. So, if you try to prescribe something it comes out with alert. It's not always appropriate alert. It's not always good. So, you have to change it, but it comes with the alternatives as well or stop what is not in formulary. But a lot of things are on the NICE guidelines but they're not in local formulary.

I: Right.

R: So, if hospital gives us to prescribe something and they will like to prescribe, it will come out again and again are you sure? It's a brown so you need to change it overall.

GP2
R: The problem with the alerts...it's like with everything else. You shouldn't ignore, you should read them again but if you are coming to the same again and again and again it's becoming like automatic again.

I: Okay.

R: Click and overrule.

GP2
R: No, it's the safety. You have quite a few, for example, if you prescribe something and there was an interaction between some other tablet, that's good. Yes. Then you see on probability if it is going to benefit patient or not. Basic things. Somebody who has asthma and or problems with their heart, they would benefit from the beta blocker and beta blocker can trigger it. So, it will come out in an alert. You know, beta blocker...this patient has asthma, are you sure? Are you sure that you're going to prescribe? But they already had before beta blocker and you're

titrating, and the cardiologists and they didn't have any reaction, but the system doesn't know. The system is too persistent. It only do what it's been told to do.

GP2
I: Yes, it does because actually what you're saying there is that the alert says...and the alert does say, don't use a beta blocker with a previous...

R: With a history of asthma.

I: ...with a history of asthma. And that's what the alert says or consider. But you've got to make then a judgement around that patient.

R: Yes. I'm doing it at all time I believe. It helps. It should be...helps but it does at sometimes it really hinders because so many...you know, if we are dealing with a patient who has multiple diseases who has 20, nobody should be, but we have patients who are on 20 medications and when you're doing the review it pops like all the time and I'm thinking okay, is there any way to switch if off by the time I finish this.

I: Yes.

R: Because it's just taking extra time.

GP3
My concern would be that it means that you do all the ones here because they're quite rare, and it gets even better, and you get a total two-horse race. And I'm a diligent prescriber, but the other place I struggled to do all the… And also the fact I do default that the quickest way to do it is to go "not suitable for this patient" rather than perhaps write down what's really…and I really don't want to get in a clinical debate with people at JPAC so I very rarely put I disagree with this rule. But there is times I do. I have had my moments, and I think that's just not right.

I: Right, okay. But that's based upon what?

R: Yeah. But perhaps I should probably be a little bit more challenging, but life's too short and too busy.

GP3
R: On it went, yeah. There was no fear about it. In our systems we are driven by pop ups, we've got so many, and we were trying to resist having pop ups on everything. Another thing is we're not sure whether other things would interfere. We were going to get [inaudible 0:09:34] for ages, and that sort of does a little bit of (the CDS)as well. So we didn't want pop ups of pop ups. So we were very happy to do it and nobody desperately minds it here probably the same, because it's not that intrusive. But elsewhere it is quite intrusive.

GP3
I: Yeah. You're not getting that many… Right. Where it is intrusive, what's…?

R: I think if it's too intrusive it makes you defeatist, and what you do is you just default to say "not suitable for this patient" and move on. And you might do one of the alerts but you won't do the 13 that come up, and I think that's just a practical way. My concern would be that it means that you do all the ones here because they're quite rare, and it gets even better, and you get a total two-horse race. And I'm a diligent prescriber, but the other place I struggled to do all the… And also the fact I do default that the quickest way to do it is to go "not suitable for this patient" rather than perhaps write down what's really…and I really don't want to get in a clinical debate with people at JPAC so I very rarely put I disagree with this rule. But there is times I do. I have had my moments, and I think that's just not right.

GP3
R: When it comes up it will give me a suggestion of what to do. It will give me some information that most of the time I've seen before, so I know the next step. So it's whether I agree with it or I do the "not suitable for this patient" or some other information down that side. In some ways I think my default in over 50 per cent is "not suitable for this patient" at the other place. Here my default would probably be to put the switch screen that says use original, and that's fine, I just click on the convert. There's one that I struggle with, and again it's my age, when you've got a default trade name. I struggle with this. Because in all my training in all my life it's been beaten out of me that I'm never to do this, and then suddenly tells me it's all right.

I: Because you normally default to generics.

R: Yeah, I have a generic default. It's a very strong default. So, for example, the amoxicillin to amoxo, it hurts. It doesn't feel right. And again it's an easy one and it makes no sense, it's no cost, but the worst thing I would ever get was a patient saying I don't want amoxicillin, I want amoxo. So I'm always just a little bit wary about those switches. I do do them. The creams and things tend to be the interesting ones because they tend to be quite seasonal, the changes. The one I can think of recently is the beclomethasone to betnovate, which again just feels a bit odd. But if I've got enough time I click on as many as I think is reasonable.

GP3
R: Yeah. The people that would be best dealing with sometimes the polypharmacy are the pharmacists that have got a bit more time rather than ten minutes, because it would be a good reminder to them. Because they're human like me. It's a good safety and cost reminder, but you just need a bit more time for it. The original polypharmacy I said to them if someone's got over 25 medications I need more than ten minutes because I just cannot do it, it's not humanly possible. But I haven't got more than ten minutes so I'm in a catch 22. So perhaps it's thinking about the pharmacist when she uses that on the multiple ones, she takes a bit more time and maybe has a plan for the patient. Because I think probably there's no point changing 12 things at once. It will not succeed. And I am very male about this, I take a single task and if I can get that single task done then I'll move on to the next one. And I think the difference between us as good prescribers here and pretty shocking prescribers elsewhere,

that's why (the CDS)works in a very different way. But we are getting a little bit of battle fatigue with it, and the fact that I will go to "not suitable for this patient" a bit quicker than I would have three months ago even.

GP3
R: Yeah. Much that it would be good if we can minimise pop ups. But I think for the safety ones I don't mind that at all. That's fine. I never object to a good safety pop up. Even the nonsteroidals, that's still a good one, even though it should be engrained in us by now. Occasionally you get the weird one that comes up and you think that's really interesting, I didn't know that.

GP4
I think it went reasonably smoothly. I don't think there was any…there have been glitches along the way where I think System One were concerned that it might have been slowing the clinical system. But in terms of our concerns about it I suppose it was like any pop-up type system really and worrying that it was going to be intrusive or it was going to take a long time or it was going to be difficult to ignore or shut down.

GP4
What if you're getting a lot of prompts for particularly…I'm thinking perhaps with patients with more complicated multiple conditions, multiple morbidity, do you find you're getting lots of different prompts for the same patient, lots of messages coming up or…?

R: Not in a troublesome way, that's not a feeling I would have had if you'd have asked me just to think of problems. It may be the case, and I think sometimes what happens in those cases, I sometimes just shove them down, I think, well, I'll make a judgment, I suppose if it's something that really jumps out to me as being really significant or important, then it'll draw my attention to it, but sometimes you think, well, this is a patient on ten or more medications, somebody along the way has considered these things, they've had a medication review, today I'm concentrating on this particular problem. So you're trusting to some extent in the system and sometimes you just don't have enough mental space or physical time to actually go back and to act on all those things, so sometimes we park things a bit, and it's a longer term project I suppose. So that…in those circumstances the prompts are about giving you a heads-up about something that might be dangerous. But some of the lower tier things I might just shove down. And think, well, I'll have to deal with that another day.

GP6
Yes and our screens are very, very busy as well and I think that it's fair to say that what's happening now with (the CDS) probably is, that while all the changes to the generic stuff is very obvious pops up and it's clear, what I'm probably then not doing is acting when it pops up with alerts because I think you just become blind. I think there are that many things that pop up and I think if I'm honest, it's only very recently I've even noticed there are red things flashing up on it going something about, what everything says torsades de pointes so everybody ignores those. But I don't think I have ever actually read one of the alerts.
I: Right so you basically...
R: I think I just delete, I just ignore them.

GP6
R: It probably is helping because as I said, you know, even though I said, I don't think I do pay attention, I think that probably the odd one where it's...because if there is a whole pile of them, if the first thing that flashes up is about the EGFR, it will make me think about checking the EGFR. If you come in with your Naproxen it might actually help me to remember to give you stomach protection. So if there is a something that pops up, I suppose there is a higher chance of me looking it up but I'm just aware of the fact that if there is loads of red mess, almost my default mechanism is to go, oh pile of shit, you know and ignore it which actually is probably the very worst thing I could be doing. But I think you also go off your own...you have an in-built sensor don't you as well and I've been doing this job for a long time and you're thinking, yes but it'll be alright because I'm only going for a low dose or something like that or I'll stop something else. I'm really not sure that...I know I have never sat there and laboriously gone through all the alerts.

GP6
R: Why was...interestingly, you say that I have genuinely actually sometimes it says, caution if EGFR is less than this, da-da, if it's less than this, da-da-da and actually often that just irritates me because I just think, well you've given me 12 different options then.
I: Yes it should know.
R: And it should know what your most recent EGFR is. The problem is, that if that person has at any point in the past had some, sort of, renal issue it might be that, that's what's triggered it but it's just so annoying because if that person actually has got an EGFR of under 30, I wish it just popped up saying, not safe, this person's EGFR is less than 30 but it doesn't, it just doesn't. It gives too many random options. It then makes me, to be truthful, I was just thinking I had just randomly said I just ignore it and I actually don't, because I think sometimes a little reminder like that maybe does make me go on to just check their recent renal function and make sure that it is suitable. But I think it's actually also a little bit random in that, I'm not sure that it always does it appropriately. I think, for instance, there are drugs like Nitrofurantoin and Trimethoprim which do rely on good renal function to be...to work, because of their function through the kidney. Well actually Nitrofurantoin does and Trimethoprim actually can damage your kidneys if your function is too poor. But those are both drugs that you would tend to use in old folk with urine infections but it's a bit random about telling you about whether they are suitable to give to a person. They only flash up, you know, so it's just too unreliable I think for me to want to read it every time it pops up.

GP6
I: So experience of having messages that you don't trust. I mean, that's one of the things we [inaudible 09:52] in here, in terms of what your trust and confidence in the system would be. I mean, on that basis would you have any...do you have a lot of trust in it?
R: No. I mean, I think in all honesty I think it is a bit too random and there is too much guff on there, there are too many things.

GP6
R: Well no that was about medications and then the alerts all seem to come at once.
I: Oh right, yes.
R: The red shit just appeared.

GP6

R: Yes my brain works fast but the system in some ways is too slow and irritable because there are so many things that are chucked at it now and the referring, I couldn't even refer people this morning because everything is so slow. So I think that is partly a real issue that all these bolt-ons are making it worse.

I: Slow down. and it's too much.

R: And for the prescribing, you see when you're going through, when we've got our repeat prescriptions to attend to each day, that come through electronically, you can't function when everything is this slow. But every single prescription that you go to then sign, the ones that are allocated to me, could you work on a system that's like this.

I: It's so, that, yes, well, no.

R: It's just clunky and ridiculous but these are the ones that are waiting for me to be signing today but if there are any of those, sort of, alerts as well, all of them need reviewing and things. There are just too many things.

I: Yes.

R: Right I think I've made my point!

GP7 and GP8

I: The safety messages you say you get in System One, are they helpful? Because they might be the…

R3: I use them occasionally. If I see three stars then I tend to have a careful look, if I see one star or two, I might not be too interested.

R2: I think the problem is that it comes up with a message for every single drug.

R3: Yes, so every time you, yeah.

R2: So every time you do anything pretty much it comes up with an entire page of interactions and everything. To be honest I don't find it very helpful because it's too much.

R3: It is too much.

R2: It's too time consuming.

I: So you're already getting, as soon as you go to prescribe you're already getting System One firing some alerts at you.

GP8

I: Then you get the alerts on the cost saving on Optimise, do you find that's, is that then, are there too many alerts or not enough or is that manageable or what?

R2: If there was a way that (the CDS) could just give you the high-quality alerts if that makes sense, so the ones that are really going to make a difference and make you think, oh no, actually I shouldn't be prescribing that. Rather than here is an entire list of every single side-effect for this drug and every single possible interaction.

I: Yes, right.

R2: It would be a lot better.

I: You see, yeah, yeah, yeah.

R2: Because it's too much to go through every single…

GP9

R: It seems to be it's coming up with a lot of things rather than just a few before.

I: Yes, is it coming up, therefore, with more safety messages or more cost-saving messages?

R: No, sometimes it's just a drug I want to prescribe and like Instillagel and it keeps asking me to change it and I don't want to change it but it doesn't accept that fact and it's just annoying. It slows down your work.

GP9

I: You did before, so you feel it's, sort of… Yes, and it's not something like…because one of the things that people have said to me is there's so many alerts, it gets in the way, it's too much, there's too…you know, you get this alert fatigue thing. Do you feel that or do you…?

R: Sometimes, it depends if it's just a… Sometimes the alerts look bigger than other alerts so it depends on how many things. Like I said before, sometimes it's more of a hindrance than it is a help but it depends on which drug and which patient and how many you've put on, really, and how many times the alert comes up.

GP10

R2: I think while ever it's not overly-intrusive, i.e. I suppose almost intrusive to the nature of feeling obliged to go down the recommended course rather than your own judgment. I think 'cause as long as it's not intrusive to that extent, then there's no reason to turn round and say, no. There'd be no reason to turn it back on I can't see. Unless I thought the CCG had its completely own agenda, which I don't think's true. That would be the only other issue, if they'd lost the vision of the health service, then I might be tempted to say no, but I can't see that happening personally.

GP12

I: That's good isn't it, because I mean as you say, you don't want that sort of, it's starting to get…..but has it got irritating?

R:	It got irritating a while back and I am not sure whether they changed things or whether I'm just less irritated by it, but it doesn't seem to be so irritating at the moment.

I:	Right, so it's not firing quite as much as it perhaps was.

R:	Yeah, that's right, yeah, yeah.

GP12
R:	And it's so bad. Actually, at our last partners meetings we were all saying the drug alerts are terrible on (GP clinical system) because we don't even know what are the important drug alerts anymore and what should be coming out. And we asked (name) and our pharmacists to try and work out what drug alerts we want to see or don't want to see, whatever, because, yeah, there's just so many alerts, there's just so many alerts.

GP12
I:	In terms of, so you say you get those alerts flying up and you said before with the CCG they were getting irritating, is there a lot, is there too many or what, or is it sort of manageable in terms of…

R:	As it is at the moment, as it is at this moment in time, I think it's okay.

I:	And what do you see….

R:	Because you can cancel it very easily. One click and you can get rid of it if you want to get rid of it.

I:	Yeah, so you just over ride it?

R:	You just over ride it, that's right.

GP14
I:	Yes, precisely. Does it…in which case when you're…in fact, I can…this issue around multiple morbidity and polypharmacies and which has been raised by other prescribers and, I've had two different messages. One which is said, well, actually that's where (the CDS)is quite useful. Others have said, (the CDS)is useful for the one of acute and actually when you've got a polypharmacy patient, you've got… it's far too much to deal with and there's too many alerts happening and, to be honest, it's just confusing the picture. So, which of those do you think is…?
R:	I mean, the first one. I think you're better getting the alerts and be aware of it and make your own decision to ignore or cancel it, because these are just suggestions, they're not…you don't have to follow through with it. But from my personal point of view, particular from a safety aspect, I would rather know even if it's flashing up loads and ignore them all, if I'm going to take that responsibility and carry on with prescribing whatever that job might be. I would personally rather know but I can see there's two different angles from it in a sense isn't there?

<u>Sub theme 5v. Technology - development, problems, limitations, solutions; relationship of technology to the engagement of users.</u>

<u>Software developer staff</u>
Software developer staff 1
And so that was probably going back almost two and a half, three years ago, and since then the tool itself has developed. Sadly, not so much within the clinical system, more within the tooling itself, in that sort of the portal people log…you know, their customers can log into, the ability with what they can do with the messages within the portal. For prescribers themselves, they've seen a couple of updates, more so within (GP CLINICAL SYSTEM), it looks a lot cleaner. I think when the first iteration came through, they didn't know whether it was an (the CDS) message or a clinical safety message. (the CDS) wasn't quite clear on that. And so they never knew whether it was one of our messages or something from System One or from (GP CLINICAL SYSTEM).

Software developer staff 1
So I've seen probably more development from a user end rather than from an end user - using the software end. We are limited on that respect because we rely on the clinical system providers to support us developing that side, they have lots of other priorities. So I think no one will really know this this but there's something called SNOMED, which is a new way of READ coding, and that on everyone's priority. So if we say, oh, can we change this in the box, that is not a huge priority for System One, SNOMED and everything is.

Software developer staff 1
We took some of the learnings from that in (GP CLINICAL SYSTEM) in that we put everything on one page, so there's less to click. And it would be nice to be able to go back and do that with System One. But that is…as I said, we can give them all the tools to do that but they need to do that their end. And that is our sticking block. So we get a lot of grief from GPs saying it doesn't look any different. And it's like we'd love it to look different and we've got all of your feedback, but we can't do anything about it, that's System One. We do encourage the end users to vote on System One and (GP clinical system), there are vote buttons that we would like (the CDS) to do this. So if we can get everyone on (the CDS) voting, it becomes a higher priority for System One, but it's getting prescribers to do that. So I think from the other end, so for the CCG, the purchase of, they've seen a huge development from how to enable to messages, how to run reports, and now giving them the flexibility to go in and alter messages themselves without having to go through our content team. And even in the last 18 months, that development has really rolled on. Where I was before, it was crazy because that was our own piece of software, so really we could have done any development requests. And I was with that company for six years and even after six years, we hadn't delivered the one thing that people wanted, which is the ability to have coloured text, for instance. So it's been really good for me to come to a company that we try and listen and development. Our sticking block is not our end, it's our partners' end.

Software developer staff 1

And then now, it's like we have calls, there are so…like, that's the other thing, we have so many calls, it's like calls about this, that and the other, it's like…the trouble is software will always have issues, that's the problem with it. I don't know anyone that has software and it works perfectly, it doesn't go there.

Software developer staff 2
I: And couldn't get their head around where they were or what was happening, were they firing, et cetera, because of the change from one system to the other. That's I suppose something which we've seen the impact of that. Do you see that at all or are you aware of the different way that it works with the different clinical systems?
R: Yeah. It does work differently, the two main systems. It looks different because of the way we integrate and (the CDS) and (GP CLINICAL SYSTEM) looks more (GP CLINICAL SYSTEM) like in terms of [pop-ups 0:21:25], and similarly in (GP clinical system). However, essentially they do work the same, but there are differences in terms of the click patterns and how you dismiss the window or action the window. But they do still fire at the same point, sort of, ie when you're adding a drug, when you reauthorise a drug. [Inaudible 0:21:51] is unique to (GP CLINICAL SYSTEM). It's slightly different in (GP clinical system). That's interesting feedback, because I thought you were going to say that they were going to say that changing clinical systems is the worse thing they've probably ever done. But everything is new.

Software developer staff 2
I: When you have those meetings and you've talked about any problems they might have had or any issues, what sort of issues do they have then?

R: It normally is flagged as an IT issue, it's slowing them down, it's slowing the system down, but nine times out of ten it's actually an engagement issue in that somebody in the practice really doesn't like it, doesn't like the extra few seconds it will take to dismiss the window if they're not in agreement or to navigate the workflow. So it's usually about particular people rather than IT. I mean, if it is IT then we do our best to support, flag it, and usually we have to go back to the vendor system, (GP CLINICAL SYSTEM) or System One or [inaudible 0:16:04]. But if it's an engagement issue, again, we want to try and work with them to resolve that.

Software developer staff 3
I: Yeah. And you talked about workflow as well. I mean obviously you and I know, both, that workflow is really important with these sort of systems. What did you do then, in terms of how it was implemented, that dealt with any issues that you might perceive happen with workflow?

R: Well I mean, well again this is where it's a complicated question. A lot of it was to do with the way it was designed, and how we made those decisions around the design. So, how did we present the options to [inaudible 00:20:34] users, so a lot of this is down to how you do mock ups or wireframes, and how you start to ask those really pernickety questions about, should this button be here, should it be here? What does this do, what do this? So, getting that kind of feedback in that initial stage is very helpful, in terms of design. To be fair, we didn't crack it, because you don't always get these things right. And I guess, some of the problems we've faced, once we've got wide user feedback, saying, this needs to change or, that needs to change, then convincing the vendors, GP vendors, to make a change, is actually quite difficult […] So I think whilst one of our partners may be very responsive, there's definitely another one which hasn't made a change to their workflow in over two or three years […] So, in some ways, that implementation has got drawbacks, because it's not up to date in the UI we would want it to have. And it's a problem we've faced previously, it's not dissimilar to the problem we had with our core CDS, and there the vendors just did what they did, in (the CDS)we've given a blueprint which they've stuck to, but then updating that blueprint is difficult. Some of the newer stuff we're trying to do, going forward, we're actually taking an approach, potentially, where actually the vendor actually gives us a bit of real estate, which we're able to configure and update as required, which is actually a much more sensible approach.

Software developer staff 3
I: Yeah right. Because in some respects, anything that's going to interrupt what the GP is doing, is going to be necessarily, it's something, a GP's now got to do something different, or a prescriber has got to do something different. So, I suppose, that's the challenge isn't it, it's finding the ways to do that, that…?

R: And we haven't cracked it, in any means. Even if you take something like antimicrobials, and trying to make sure people are prescribing the right antibiotic, which is aligned to local formulary for the right condition. The problem is, as a clinician, when you go, and you don't think about the data entry side, you see someone who's got a chest infection, you examine them, you take a history, it sounds like they've got a chest infection, stick them on amoxycillin et cetera. But the problem is, what they do is they stick in amoxycillin, without putting any of the supporting information. So you're not able to then, that supporting information is actually really important, because it guides our decision support to some extent. […] And so, there are other examples of workflow where actually you need the right data to be inputted, for (the CDS) to work, and it's something where we're kind of looking at, not necessarily for a new product, but in terms of the modules, or in terms of areas where it could be improved

Software developer staff 3
R: Where it does get interesting, is when you move more towards the audit side.

I: Right, yes, yeah, right.

R: Because now you're able to kind of capture patients where it may be appropriate to consider whether they should be started on a new treatment, which we can't do in Optimise, you can't do, 'cause the trigger is a medication being prescribed, you can't advice that there's a patient who has been diet controlled for their diabetes, but actually their HbA1C has gone up, so we should consider starting them on some sort of oral hypoglycaemic agent. But, with an audit tool you can start doing that. You can start identifying opportunities for new intervention. You can also start thinking about opportunities for escalation of therapy. So, those are definitely areas where we're looking at, and you can start doing mass bulk changes, or you can start understanding who needs what test. And that kind of management of patients, we think is going to be take up more and more by non-medical staff.

Software developer staff 3

R: But even then, unless you're making a prescribing change, or it's a new drug being prescribed, that information isn't going to be taken account of. […] I'm just trying to think of an example. So, patients on an oral hypoglycaemic agent, and actually their HbA1C has been in control, but their renal function has been going off […] At that stage, you may say, right, it's time to add in an ace inhibitor, or any ARB too as part of their long term management plan. So, we need to stop x, y and z. So, they may have had their renal function tested, and they may have had their albumin-creatinine ratio measured, and those values may have been put into the system, but unless, (the CDS) isn't going to tell you to consider an ARB, unless you're putting it in, you're actually prescribing something for it to go, ah, I'm running this rule, I'm looking at a renal function, and I think, right, we need to do this.

Software developer staff 3
I: Yeah, absolutely.

R: And so, there are other examples of workflow where actually you need the right data to be inputted, for (the CDS) to work, and it's something where we're kind of looking at, not necessarily for a new product, but in terms of the modules, or in terms of areas where it could be improved.

I: Yeah, I mean other people have talked to me, and said that, you know, the system's very much dependant on good diagnostic coding, because without the good diagnostic coding, particularly since it's attached to the specific patient's record, if the diagnosis isn't there, the interaction is not going to fire.

R: Or, it does, so what you have right now is, if you put in an antibiotic, and then you may get completely irrelevant pieces of guidance coming up. So, the way we write it would be, if you think they have this condition, if you think they have, so they get presented back, potentially, three or four different types of alerts, because that initial diagnosis isn't in the system.

<u>CCG Staff</u>
CCG Pharmacist 2
R: Yeah, and I think what I found interesting was that (GP CLINICAL SYSTEM) are quite picky and choosy about what they want, so if you've got this little e with a little slash through, you know that message is not on (GP CLINICAL SYSTEM), because they have their own. And that's what I mean about (GP CLINICAL SYSTEM) being a bit more, I think they're a bit more intuitive, they're a bit more proactive, in terms of their messaging, compared to System One.

CCG Pharmacist 2
Before (the CDS) came in, it was like, shall we think about having a protocol in System One, or do we just want this to go out as a key massage, via our Medicines Management Team? So, they have that face to face conversation with the practices. Or, it could just be as simple as, we'll add it to the next newsletter, we've got like a Derbyshire Medicines Management newsletter, that goes out.

CCG Pharmacist 2
Or equally, it has to integrate well with the existing systems, I think that's the key thing, so it's an add on to, and it tries to enhance the system that's already there, I think. I think it's more clinical, rather than practical, operational, but I think there's lots of things that are in the clinical systems that get missed, that prescribers could see, but they're not necessarily seeing, depending on how it's displayed. So, that's unfortunate.

CCG Pharmacist 2
R: No, it could be an admin person or, yeah. Interestingly, I think my own understanding of what (the CDS) messages are intended for, and how they fit in with clinical systems, has developed as time's gone on, obviously going to these meetings on Optimise, and obviously with (name) being there and explaining things, I'm like, oh I can see where you're coming from. You know, like in Prescribing Group sometimes, you're like, at least it's a quick fix, just put an (the CDS) message in. And it's like, well hang on a minute, some of this stuff is already in the clinical system, it's whether people are choosing to see it. And I think people have just become, it's become like white noise, like, especially in System One, the way it's set up, I can see, like allergies get missed, or interactions are a bit further down here, or contra-indications, depending on the history of the patient, it could be down here somewhere.

CCG Pharmacist 2
I have confidence that obviously it will trigger and deliver the message you want it to. But, again, it's the end user that you're having to rely on, in terms of whether or not they read it, act upon it, or over-ride it in some way, or whatever it is. So, I think it does what it says on the tin, if you know what I mean, I have confidence in that, and when I've read some of the messages, I'm like, yeah, that really encompasses what the drug safety update was about. And if there are tweaks we need, we'll feed that back to them or whatever.

CCG Pharmacist 2
R: One thing that did come up as a bit of a concern, and it's been in the back of my mind, and it keeps coming to the forefront, and I'm like, oh I've forgot about that, but there was an issue once where, and it wasn't (the CDS)'s fault necessarily, but it was the way it integrated with the clinical system. So, the example was, I think it was azithromycin, I can't remember which was around it was, it was meant to be switched from capsule to tablet for example, because it's more cost effective. So, it did do, the System One did do that, 'cause (the CDS) said, you might want to do this, it's cheaper or something, it did do it, but what ended up happening, was the dose then changed to the default for that tablet, in System One, so it ended up being the wrong dose, and frequency.

I: Right.

R: And then that then raised an issue about, oh, oh, are there any other drugs that this could have happened to, is it significant if the dose is changed, do the doses change for those drugs in System One? And in some cases they did or didn't. So then, a lot of work had to be done with System One, I think (the CDS) really did help with that, I think, getting patient safety sort of hats on, and saying, how can you change this in System One, to make it, so another alert had to come up to say, prescriber, do you want to maintain the original dose you put in, or, do you want it to go

to the default dose, that is now changed to, and which is this? So, at least it's alerting them, 'cause if you don't know at all, and you've just assumed, it's carried across, yeah.

CCG Pharmacist 2
I: Just thinking in terms of some of the stuff we said last time, we were talking last time around your role and some of the communication with and from GPs. One of the things that you mentioned was the reporting of medicines, for instance, from primary care. Is (the CDS)helping at all with that? Has (the CDS) been able to help with any of this reporting back from GPs about…or have you been able to look at the hit rates of things as well?

R: It's a good question. I haven't actually investigated any hit rates as much as maybe I could do. I don't know what it would show because I think isn't there something, I'm just trying to remember now, when it comes up… Unfortunately, see, I don't have access to the clinical systems, I've only got access to System One demo and that's not linked into (the CDS) so I can't actually see them come up and interrogate the system and see when they come up and what it looks like. So I think some people have said when an MHRA comes up I think… I can't remember what the screen looks like but you either can accept it or reject the message or something like that. I can't remember. I think some of them are rejected but I don't know what the… I think it depends on how good that data is, do you know what I mean?

I: Yes.

R: It's almost like, how good is that data? What does that actually mean if they press reject? Did they mean to or is it just because it's on the wrong side of the mouse, do you know what I mean?

I: Or it's just that it's a click, click, click, click, click to get rid of all the alerts?

R: Yes, exactly. Yes, I think when we looked at it, I might be wrong, but you know when you're on your mouse, you left click, don't you, and it's automatic. I think the left click was the reject. I can't remember.

CCG Pharmacist 4
What has been an issue is when it goes down. So we've had a couple of outages recently, and practices don't like that. So one day, afternoon, it went down for about 40 minutes and there was big delays, and obviously if it's slowing the clinical system down that does concern me I guess because you're going to potentially disenfranchise them with it.

CCG Pharmacist 5
I: No. And I think (GP CLINICAL SYSTEM) practices, some of the safety messages that are at (the CDS) come through (GP CLINICAL SYSTEM).
R: Yes, (GP CLINICAL SYSTEM) don't have all of the messages that (GP clinical system) have because they've got their own internal ones that do the job.
I: Yeah. So, (the CDS)works very differently with (GP CLINICAL SYSTEM) than it does with (GP clinical system), as I understand.
R: Right, I don't really understand the interface so much, but I, yeah, I know they are different.

CCG Pharmacist 5
R: There are limits to it so it only triggers when you prescribe something, whereas we want it to trigger on other things so like if they enter a read code so if somebody's newly diagnosed with something, that would be really helpful [voices overlapping 48:35].

I: Yes, because if you entered a read code on a diagnosis and at that point it then fired a message to say, right, well now this person's got x, you should be taking them off…

R: This is what you need to do.

<u>GP Staff</u>
GP Nurse 1
I: So going into the GP, so when you come into a practice like this you go into the GP system and use the clinical record from the GP?

R: I will normally use the community module but like, for example, today I've been to a care home and there's a few repeat items they were asking about, and so I'll go into the GP module and then look at the prescribing history. And then if, for example, they'll say there's not enough medication to last the 28 days I will go into that repeat template and change it from there, from the GP module, rather than do it from the community module because it gets confusing. So I have to work with both and so when it comes to...when I do an acute medication, like a chest infection, I prescribe antibiotics, I'll use my community module because that's what other people working for the community trust would do. So I have to try and work out which is the best way of doing it for communication.

GP Nurse 2
I: I remember you talking about how that felt…you know, it was different but the system was very helpful because of [inaudible 01:55]. How do you feel, you know, what, we're a year and a bit on from when we spoke? Is it still helpful in that way?
R: Yes, very much so but one thing that we have done, I don't know if you've heard of it as well but we've also got a company called Ardens in and we use their templates.
I: Oh, right, yes.
R: As part of their templates they have the formulary on so, for example, if you see a patient who's had an animal bite, then you can look on the formulary for animal bites and it will guide you to what… So, I guess it's another safety net, really.

GP Nurse 2

I: You talked about that other system, the Ardens system that you were…again, would that be part of that, if you like, using it in complement to other things, as it were?
R: I think I would, yes, because with the Ardens template they've got formularies, say, for example, migraines so you can look at that and use it alongside as well. It's just another tool so you've got all your tools there, then, that you can use. It's got the formularies, so for example, there's one for, I don't know, let me have a look. I'll have a look on it for you. Antihistamines, for example.
I: Right.
R: Then there'll be formularies so there'll be things on there that it'll be saying to you which ones you can prescribe with the ages which I find quite helpful so you know for your children it'll work that out for you which is excellent.
I: Right, yes.
R: So, in that dosing if you're using it through Optimise, it's belt and braces then, isn't it?
I: Yes.
R: Yes, so I find it all really helpful to be honest.

GP1
R: It's quite a long time ago, I think it was through the medicines optimisation. We had a previous software, I can't remember what else it was called any more; it was another CDS.

I: Yeah, another sort of CDS system.

R: The guys had looked at Optimise; I think it was cheaper, and that they felt it was giving us what…I think there'd been a problem with the other system, that they weren't updating it brilliantly; they'd put the price up and then they were wanting a commitment that they weren't willing to make, so they looked at the (the CDS) system and decided it was pretty much the same. I think, I can't remember now, there was one very positive thing in its favour as well, which I can't remember what that was now.

GP4
I: One of the things you talked about last time was, you know, whether the system was over intrusive or whether the prompts were useful and things like that, what's your feelings now, a bit further down the line, about them?
R: I don't find them too intrusive really. I mean it is another thing that happens that didn't happen before but it's as we discussed before, it was they are relatively easy to override and the suggestions that are being made generally feel quite sensible. So, you know if it's a cost change that you think, well it's not that important and you can override it very easy. And if it's a safety thing, then generally I've been quite pleased that it's happened because it's given me an opportunity to make a change before potentially something that might cause me a problem later down the line or caused a patient a problem later down the line.
I: Yeah, quite.
R: I don't mind it really. So, two observations I've got really, one is that when you're invited to make a brand substitution or some sort of formulary change, it can be quite difficult to find the suggestions. Other doctors have commented on that before. So, in a way it sort of makes it difficult for you to do something positive. You'd say, right okay, yeah, that's a fair suggestion I'm happy for you to now give me a recommendation. If you then click on show suggestions, they don't always come up without you ticking a very small box in the corner – and I can't remember what it says – but you sometimes open up the suggestions and it's just got a blank box, and then you have to tick a very small box in the corner which then brings up the options.
I: Oh I'm with you, yeah.
R: I'm certain that sometimes what people have done is, they've opened up that box and gone, oh well there's nothing there, they don't know how to access it, so they override it and carrying on with their work. That was one bit of feedback. It would have been easier to generate suggestions which you could click on very quickly and action.

GP4
I: Yeah, precisely. So, in some respects that, as you say, there is two things there, either it's slowing down your workflow but it's also potentially just give up because you are not seeing those suggestions. Yeah.
R: The other thing was about, and this came off the conversation I was having with the clinical pharmacist when she'd been looking at the log, she'd noticed in the log that somebody had overridden something which she found quite surprising and I think it was something to do with a warning coming up when somebody tried to prescribe Nitrofurantoin and I think the EGFR had been less than 30 or something, and somebody had overridden that, she thought that was quite surprising. And then it had occurred to her that it didn't necessarily mean that the clinician hadn't actioned it, and I think that's something to do with how the log might work.
I: Right.
R: So, for example, if you, and I definitely know this has happened in my case, that I've had a warning come up and then I've gone on to show suggestions and I've not been able to make that work, and so I've just closed the whole thing down. But then actioned for suggestion, just by prescribing it from the acute drugs within (GP clinical system), so I've actually made the change but I've done it myself, I haven't done it through the (the CDS)system. So, whether the log would see that or whether it would just look like it had overruled it.
GP5
We got told, that …'cause we had something else before that, I've forgotten the name of it. But, we were told that this was an alternative. It was better. We would find it more useful. It was better integrated into the (GP CLINICAL SYSTEM) system. But it was similar to what we already had anyway, and so we shouldn't, besides the screens looking a little bit different, it shouldn't really impact on our workflows or anything like that. And essentially, by and large, they were right. It had a few little extra bangs and whistles, particularly I think, to connecting to links to websites, if we wanted to know more, which seemed a bit more better integrated, so we had all the functionality of previously, plus a bit more, and it was a bit tidier, and a bit more Windows.

GP5
R1: More times than not, I'd just follow it through. Just 'cause, it's the time factor. But, you know, on the odd occasion, when I've though, oh, I wonder what that's about, that's possibly…'cause often it'll flag up something that's possibly a learning need for me.
[…] Which, would have been nice, 'cause if I can then go to the page, I can then make a note of it, and then go away and read up about it.

I: So, having that sort of evidence?

R1: Yeah. So, if you take the example of, I didn't know that beta blockers cause, or can make asthma worse. Oh, that's a doctor's educational need […] I can go away and learn. But then, of course, if it says that and then the link doesn't work?

GP5

R1: A big problem we've got locally is opioid prescribing.

I: Right, yeah.

R1: Okay? So our co-codamol prescription is through the roof. It's probably one of our top ten drugs. (the CDS)doesn't really help with that.

I: Right.

R1: And yet, we need to think about, how do we reduce our opioid prescribing? So, and again, that's where the Map of Medicine would come in. Because often times, these are depressed patients. Lost their job, they've got pain, painkillers aren't working. But the reason they've got the pain, is 'cause of the emotional stuff, it's not the physical stuff. But, we cant do anything about the emotional stuff, so what do we do? We just whack up the painkillers.

I: Yeah.

R1: Where's the safety in that, what does Optimise…and you can see can't you, that (the CDS) isn't designed for that.

GP7, GP8 and GP9

I: So I mean just to start off with, perhaps describe how, any one of you, or you know how you came to use Optimise, how it was introduced into this practice?

R1: By a pharmacist wasn't it? We had to have a discussion with her about how it could potentially help us.

R2: I don't know. And then we had a trial period.

I: There was a, right, okay.

R2: I think we had a trial period.

R3: My understanding it was just introduced onto the IT system in the patch.

R2: System on, yeah, the area.

I: All at once, yeah.

R3: So, it was tagged on or whatever they do and then (name) our pharmacist, gave us a bit of a talk and more or less stood us in line and said we should use this.

I: So there was no sort of like, was there any sort of training, any sort of, apart from this is what it's going to do, any sort of…

R2: No, I don't think so.

R1: I don't think you needed much training, to be honest, for it.

R2: It's fairly straightforward, isn't it?

GP7, GP8 and GP9

I: So are you finding it a helpful or a useful tool to have?

R3 The answer to your question, it wasn't completely seamless.

I: Oh right.

R3: Because it was a bit of an "oh my goodness" this box pops up and I know for me originally, I'm not very IT savvy I couldn't work out where the alternatives were.

R1: I don't think that's very obvious still.

R3: So it said there were alternatives.

R2: But you have to tick on the box don't you, it's not, it should just appear straightaway.

R3: And you have to reveal the alternatives and for a while, I think maybe a month or two, I carried on doing what I was doing.

R1: I didn't realise for ages the alternatives were there.

R2: It would make more sense to do the alternatives without having to do that little extra click on the box…

GP12
R: Yes. Well, I mean, the big change is that we changed from (GP CLINICAL SYSTEM) to (GP clinical system), so that happened. So we migrated in August, so that was a massive change to our whole computer system, to how everything looked like on the computer screen, to how we were dealing with things. And that took, it took quite a while for us to sort out prescriptions and prescribing drugs, a lot more clicks on (GP clinical system), a lot more complicated. So the whole prescribing area has been a massive change and took us about, you know, I'd say two to three months before we really worked out the prescribing system on (GP clinical system) because it's just been completely different from (GP CLINICAL SYSTEM) I think unexpectedly different, and much more convoluted and much more complicated.

<u>Sub-theme vi: Time</u>

<u>Software developer staff</u>
Software developer staff 1
And people say…I think one of the biggest issues is on (GP CLINICAL SYSTEM), on repeat authorisation, it just slows it down. Because if they're on 12 items, we will check if there's a message against all, and they're like, I haven't got time for that. My counter argument is, so you would have just re-authorised it and not looked and not looked at anything, and sent them out the door, yet we've pinpointed there's a cost you could have done, there might be a dose reduction, there might be some monitoring that's required. We, in essence, do a lot of that review for them in that piece of software. That's saving them a job, because if they send that person out and you've not done a set of bloods, and we actually realise that their white cell, you know, count has dropped through the floor or whatever, because they didn't even think about it, and we've brought that to their attention, that may have prevented something happening in the future.

<u>CCG Staff</u>
CCG Pharmacist 2
So, because we're a very small team here, we're, I think, probably one of the smallest, as in teams, per patient population of a CCG, and time is a big problem. Well, not a problem, it's a big commitment to put in software like this, so it was the additional support that came with this system that was attractive to us. And a lot of the work that we had to do, using the previous system, would take up so much time, that this seemed more attractive. And the fact that a lot of the safety and quality stuff is automatically done and updated for the system and the users was very attractive. Because the other system, we had to do that ourselves and update it and find the information, and then update each line, so it would take an awful lot of time, so, that was a big pull for us.

CCG Pharmacist 2
R: No. I found, when we used the other system, I would send quarterly reports and nobody looked at them, very, very few feed-back. And to be honest, an everyday comment that you hear when you're in surgeries, or even on this floor, we get bombarded with emails. So, for me, what I think, if I'm doing a project in a surgery and I just wanted to check if I can use Optimise, to say, oh, is that complementing what we're doing? So that would be useful. So, I've not used it as that yet, but, moving forward, I think when we get more of the outcomes side of it, and getting used to using that, which we're not at the moment, we're still developing, so I think when we start to move towards that, we'll probably use the reports a bit more.

CCG Pharmacist 2
R: And then, I don't know, actually I don't know the answer to this question, but it's just come to my mind, and I just wonder whether, has it released our team a bit more, from things that they might have been asked to do with regards to switches, for example? So, like, if you're relying on our team to do those switches, probably the or ones you still do, the ones that you're saving a lot more, but you need to do it a lot quicker, or you have to do it in a bit more of a streamlined manner, like inhalers, there might not be like for like, or something. But I just wonder whether that's released the team a bit more, because (the CDS) is now doing that, more at the point of prescribing anyway. So, you're not having to do as many switches, because it's doing it for you, and then you can be released to do other things.

CCG Pharmacist 4
I: Well, in terms of getting it going in practices.

R: Yeah, in practices I was there turning it on for them in terms of that process. Because it was implemented quite quickly we perhaps didn't go through the usual work up of having a meeting with practices, telling them about it, giving them a demo, that sort of thing. It was just launched because we didn't really have time to do that. But yes, I was involved on the ground and answering queries and switching it on…

CCG Pharmacist 4
What has been an issue is when it goes down. So we've had a couple of outages recently, and practices don't like that. So one day, afternoon, it went down for about 40 minutes and there was big delays, and obviously if it's slowing the clinical system down that does concern me I guess because you're going to potentially disenfranchise them with it.

<u>GP Staff</u>
GP Nurse 3
I: So, do you think it's a good idea to have the system?

R: Yes.

I: And would you…say for instance, if the CCG said, we're not having it any more, we're not replacing it, what sort of changes would that make to your job then?

R: The BNF would be well used, to be honest, because I mean that's the time it saves. You'd be looking up every…if you had poly-pharmacy going on, you might be looking up ten drugs. An interaction to whatever you want to give, whereas that would tell me.

GP Pharmacist 3
I: Do you think that's something that is shared across the practice or do you think people would use it differently, or is, I mean you said about people just switching off that, would be just ignoring those.
R: Yeah. The clinicians here are very diligent in the work that they do, but it was just discussed a couple of days ago, that they do follow software recommendations, yeah, and they do find in general that recommendations when accepted it's good. In previous practices I've worked, I think the messages were just plainly ignored.
I: Yeah.
R: Because the clinician really just wants to crack on with his day and get on with it.
I: Yeah, is that a time consideration then?
R: It's time consideration, yeah. Especially when GPs have got a very complex patient in front of them, presenting for one issue but it's quite a complex consultation, they've already made their prescribing decision in their mind, I don't really want to be railroaded by a piece of software telling him to do something else.

GP5
R1: I do, but it's fairly rare. And, it's a bit like the clinical system, that you know, how often do we actually follow through on the safe measures, and how often does it actually change practice? Or, do we just close it down and move on, because we've got 50 other repeat prescriptions to sort out?

GP5
R1: But, in the grand scheme of things, it probably adds another five minutes to my day, which considering the amount of pressure I'm under, it's not. Those extra five minutes, means that I'm saving the health economy money that can be ploughed into, and maybe help to support some of the other challenges that we've got. So, in that regard, if you said to me, I'm switching off Optimise, I'd kick up a right stink […] So, does that give an answer to that question about trust and confidence?

GP6
R: Yes probably one of the many reasons why our systems now run so slowly.

I: Yes now that's something that also been mentioned to me, have you found that, yes?

R: Well we think it must be. Well the problem is that constantly changes happen to all sorts of bits of our system, like, Docman and LexCom and (the CDS) and all these bolt-ons that are now a part of the system. And we don't know whether that's what... I mean, I was actually just trying to call a patient when the girls messaged me to say you were coming in, well you were here but the system literally was taking, like, four minutes to give me the patient contact details for me to even call her. It drives us all crazy and we never know which of the many bolt-ons, so I would love to blame (the CDS) but it might not actually be their fault.

GP 11
I: Yes, precisely. When you're getting all these alerts, does that have an impact upon time?

R: Yes, and probably part of this is the bigger thing of what a doctor intends to do with a meds review and how much time it would and does take to do properly versus what a patient thinks it is. If you've spent 12 minutes on a minute 10-minute consultation and it's, while here, can I just have the pill again or something like that or can you review my meds? Just a click of a button and nothing else. And so, I think it's that mismatch of communication which is probably a bigger thing. When do you not and when do you get stricter on...no, this actually needs a good five to 10 minutes in its own right to look through?

GP12
R: Whereas actually in (GP CLINICAL SYSTEM) it was much better presented. Somehow it just looks simpler. It was the tight spacing, the whole issue that it was a smaller box. (the CDS) is written in a bigger type face. You knew what it was. It just seemed to be, I don't know whether there were less buttons but it just seemed that in the (GP clinical system) version of Optimise, it just seems so much, you know, so much more potential for different buttons that you're expected to kind of get right.
But actually, you just don't have that time when you've got a patient with you and the patient is talking to you. They're telling you about their next problem while you're trying to prescribe their last drug that they want. And it's just, it's all I can do to remember, override or, you know, just two buttons to get through quickly before, you know, while it's going on.

GP13
I: Yeah, well I mean, that's actually a question I was almost about to go onto, was sort of what sort of trust and confidence do you have in the system?

R1: Sitting her in the context of an interview, a cautious, positive trust in it. When I've got 68 prescriptions to sign and it's eight o'clock at night, probably absolute trust in it, because I don't…

I: 'Cause it's about time again?

R1: Yeah, because my brain, at that point, isn't ready to start analysing, you know, I'll trust it, and I've paid Defence Union fees. Which, that's another form of risk management isn't it, paying Defence Union fees. But, yeah, so probably anything varying from a fair bit, I don't like a scale, a fair bit of trust, to far too much probably, depending on the other things that are trying to attack my brain at the same time.

THEME 6: Learning, Education and Change

<u>Sub Theme 6i: Learning and behaviour change</u>

<u>Software developer staff</u>

Software developer staff 3

R: So, how do you start doing that? So, that's something we're looking forward to. There's different types of reports we're looking to create, so the concept of, it's all very well understanding the accept rate, but actually, your accept rate, that we're reporting, may drop because actually, because we're triggering these pieces of information, people are learning not to prescribe inappropriately to start with.

I: Yes, precisely yes.

R: So, the idea of a report that says actually, looking at your behaviour, it is possible to say that, because some of the activity you may be seeing from what's been prescribed or what's being dispensed, may be attributable to the impact that that RX is having.

I: Yeah, I'm with you. So, there's two things there isn't there? There's one that's sort of saying, 'cause the actual, I mean the hit rate, it could well be as you say, it could just simply be that they're getting better at prescribing. But in terms of, also there, you were talking around grouping things around. And that grouping would be around disease specific things, or just specific groups of drugs would it?

Software developer staff 3

I: Yeah, precisely. And what sort of place did you think then, the tool has in terms of creating change or in terms of prescriber education and change in prescribing habits then?

R: I think we are touching the tip of the iceberg, if that makes sense.

I: Yes.

R: Yeah. I think a lot of this is about, as with any decisions point, the right information at the right time, in the right workflow, and to the right person.

I: Yes, absolutely.

R: And, the more specific you can make that information, the more likely you are going to drive change.

So, I think there's work for us to do to show customers how change can happen, 'cause right now I don't think we do a very good job communicating how our content should be used. So that's an area for improvement, you know, I think as much as we know that your configuration is important, I don't think we necessarily support them as well as we could, to manage that configuration. So, that's an area of focus going forward.

<u>CCG Staff</u>

CCG Pharmacy Technician 1 and CCG Pharmacist 1

We have some nurse independent prescribers as well who also get use of the system, and two of our surgeries have got pharmacist prescribers actually working in there at the moment, and obviously they are switched into the system as well, and sometimes if I've got a comment about those it's sometimes because we have shared care protocol requirement for all DMARDs and so that prompts do not prescribe without a DMARD shared care protocol. If they don't find one they write to the patient sometimes and say we can no longer prescribe these. So I think it's sometimes shone up things which have gone wrong in the past, and okay, we've overlooked this, maybe we just need to get this rather than telling the patient we can't now give it, because that's the wrong approach. It just shows you what you need, not that you can't do it. So sometimes we've got an unexpected consequence of the messages that are coming up.

CCG Pharmacy Technician 1 and CCG Pharmacist 1

…and as we get more clinicians of any professional working…so in time as we get more pharmacist prescribers perhaps on the patch, and I know there's more nurse prescribers that will be coming through, and whether there will be other groups, physicians' assistants might be able to prescribe but obviously you're going to need a legislation change around that, we hope that more people will be able to see the recommendations and act in accordance with what we're asking them to do. But on the flip side of that the more you enlarge something the more difficult it is to control and the more complicated it becomes, and if you can't differentiate clinician A who happens to be a GP from a nurse, from a pharmacist, these anomalies I think will grow. So it's very clear that we need to be able to drill down to the individual who made that decision so that we're not trying to talk to 40 different people, we can find the individual who actually needs that education and actually tackle that directly.

CCG Pharmacist 2

And I don't know, I'm just thinking from a clinician's perspective, I wonder whether there's more, doing it in the Pincer way, there's more of an underlying understanding that occurs, because you're actually seeing what's going on in your practice, and going, wow, we have ten patients on this, have we got a bit of an education issue, that we need to deal with in our clinicians? Or, what are the reasons, is there a pattern, is it because it's been suggested by specialist consultants or something, and we need to feed that back to the hospital, that something's not right? Or just generally, just going, oh, this patient has been going on for a while, have they had an episode of bleeding? Then you're doing a bit more of a thorough review in somebody, in those cases.

CCG Pharmacist 2

Think about things a bit more. Not that the (the CDS) is…'cause the ideal is, at that point of prescribing, you want that person to be safe, and there's been a change made or whatever. So like, the addition of an NSAID, I need to put a PPI in, or whatever it is, 'cause that's the time you want to do it. But, it could be that if people slipped through the next, it's like, how has that happened? And again, is there a message that things are

being over-ridden, is that why that's happened? It's asking themselves those sorts of questions, or like an education issue or something. But both of them, how I see it is, you know the Swiss cheese model?

CCG Pharmacist 2
I: Yes, precisely, I mean that, sort of, follows onto another question I had written down here which is, are the benefits of (the CDS) to improve safety, do you think they are now being realised or continuing to be realised?

R: I think so. Again, it's like it all goes down to this, you know, whoever's reading them and appreciates them really will appreciate them and then you've got some that probably think, oh, this is just a message and I'm just going to click yes, whatever, sort of thing and carry on. I don't know. Without speaking to some of the GPs it's difficult to tell but I mean, like you say, one way we could try and look at it is look at the data and the hit list or whatever it is to see what's happening there. It's not very often that you get somebody… The thing is I think it's the nature of how systems work and the way the organisation works and the NHS or whether it's GP practices or the hospital is, you only ever hear of things where they've gone wrong. So like why did that get prescribed despite the fact that there was an alert in place? What about all those times…? It's hard to quantify and it's hard to capture if that alert hadn't have come up, could a harm happen? Could a harm happen as a result of the fact that there wasn't a message or a safety net, a bit like we were talking about the Zomorph thing […] If that wasn't there, would that patient x be harmed? Did that alert prevent something from happening? Say, for example, did a GP go to prescribe 100mg and when that alert came up they were like, oh, no, I didn't mean that, it's ten, but you never hear about that, do you? You never hear about those ones.

I: Yes, if it hasn't happened, you're never going to hear, you know.

R: No, exactly, you're never going to hear about it, sort of thing.

CCG Pharmacist 4
I: And do you think the messages get accepted quite readily or do they get rejected generally across the…?

R: One of the things I, and we've, been doing is looking at what their acceptance rates are. So that is something that we look at quite regularly. Historically we've certainly been higher than the national average for both the acceptance rates for the safety messages and the cost saving messages. We're perhaps on par now. So the acceptance rate is pretty much around the average for both. But that's probably what you'd expect as changes get made and prescribers get used to it really, I suppose behavioural change. What is noticeable is our hit rate is quite low.

CCG Pharmacist 4
I: Yeah, precisely. How has (the CDS)changed things, either about the way you work or about the things that happen in the practices? Or perhaps it hasn't.

R: It's probably all under the radar. So a lot of our work historically has been around the housekeeping type roles and changing brands and prescribing, that sort of thing. I probably could get access to the figures, but I guess some of that will have shifted onto the (the CDS)line rather than to the savings that we make personally by reactively working in practices really. So that's probably made a difference. I can't say there's been a eureka moment where I think (the CDS)solved that, but equally I'm doing reviews with practices at the moment around low value meds and de-prescribing those in line with the NHS England prescribing, and my practices are really, really low. So you could argue, and you probably could look at the reports to see if they've de-prescribed dosulepin or de-prescribed lidocaine as a function of an (the CDS)message trigger, and you could probably look at that. But certainly that work that I've got to do around that is quite low. It's done in most practices because it's literally a handful of patients. So I think there probably is some, albeit quite anecdotal and subjective evidence to say that prescribers are probably getting it right first time.

CCG Pharmacist 4
R: I think we talked about Pincer and I think Pincer's probably quite a good example, and I think where (the CDS) is good is it's at the point of prescribing for GPs. So if you're going to invoke any behavioural change, I think that's where we always struggle, by mopping work up you don't really make them think, whereas (the CDS) at least does make them think actually.

I: So you think the point of prescribing's more effective at making them think?

R: Definitely. And I don't think you can measure it particularly, other than through a surrogate marker of what the trigger rates are. But I think we probably are affecting some behavioural change. So I know we've done academic detailing before, I know the evidence is there to say academic detailing does… that's where Pincer comes in, doesn't it? […] Does it affect behavioural change, and actually I think there's probably anecdotal evidence. Surrogate markers of triggering rates, it probably is having an effect. Whether it's because, like you say, they're avoiding the message by where they're actually thinking through the clinical decision tree I don't know, but…

CCG Pharmacist 5
What do you think of, firstly, the PINCER approach to the (the CDS)approach? How do they compare? How do they work together or do they work together? Are they different?

R: I think they do work together. I think you couldn't claim that (the CDS) did PINCER. I think Optimise…

I: Has the PINCER messages, yes?

R: …assists with PINCER but without the pharmacist intervention. You're not changing practice, you're just… I think if you get to the stage where an (the CDS)message fires, in a sense, the PINCER process isn't really working. It's the safety net to stop those patients being injured but without the pharmacist education then the GPs aren't going to change their practice to stop those patients becoming a risk in the first place.

CCG Pharmacist 7 and CCG Pharmacy Technician 2

R2: Yeah, because there's other ways you can do that work.
R1: Yeah.
R2: And sometimes you need that human touch instead to them...
I: That's really interesting because that's…can you go into that in more detail?
R2: So for example if there's a switch from, say, modified release docs as a [inaudible 00:21:56] to the plain docs [inaudible 00:21:58], as a switch, that might involve…well, it is really involved, you have to check someone's blood pressure a couple of weeks after. So if you add that on as a switch a lot of people wouldn't be bothered to accept the switch because then they have to arrange an appointment and they've got to speak to the patient, whereas then I moved it into our technicians' work stream, which is what we have, the technicians, all the work streams we work on, so when it went into that, it gets picked up that way. So we can run off the list of patients and actually go and speak to the practice and say, we've found this, this is what'll be involved, do you want us to make the switches, send a letter out to say, you need to book it in two weeks? Or some GP surgeries have the blood pressure machine in the waiting room. So just having a bit more of a human touch to it…
If there are too many steps involved with a pop-up it's not going to work, or if there's something that it's flagging you need to review this, it doesn't always work either because, well, I'm not…I'm doing this…I'm in this consultation for something else, I don't have time to do this review. You know, so for things like that what we've found is, there's point having that message keep coming up, you need to review this patient, you need to… So with certain things, you know, like for example, inhaler reviews, it's not always the best way to flag that up.
I: So basically, the better approach is for you as a CCG to be involved in supporting the practice, identify those patients than doing…?
R1: Yeah, there's other ways of doing the work.

<u>GP Staff</u>

GP Nurse 2

I: Yes, Optimise, then, when you're getting those alerts coming forward, do you think it has changed the way you prescribe? Have you learned anything through seeing all those alerts and stuff? Do you think there's an educational element, I suppose?
R: Yes, so if you're trying to, say, prescribe metformin on someone whose renal function is bad, it'll alert you and although I'm aware of that, I think not everybody is. So, I think certainly, yes, definitely. I think anything that offers some sort of help has got to be better than nothing.

GP Pharmacist 1 and GP10

I: And do you have confidence in the system? Do you think it's, you know, it's valued and it works, it's fine. It's, what it's suggesting seems to make sense or what?

R1: Yeah.

R3: Yeah, I do.

I: In what sort of ways?

R3: Well like because it encourages you to prescribe, think of cost. It breaks your habits, so if you've been used to prescribing something historically or whatever the drug reps have asked you to prescribe, you can, you are re-directed. It changes your behaviour […] You know, it changes my behaviour and I think I do learn a little bit. You know, I do learn to prescribe [inaudible - 0:24:30] or whatever, or [inaudible - 0:24:32] or whatever so.

GP Pharmacist 1 and GP10

I: Within that I mean PINCER has a learning effect or a learning part to it, is there learning in Optimise?

R2: I'd argue not.

R: I don't think so.

R2: No, I'd argue not. I think you could say that because it's warning you for the next time, you won't do it but actually that's not the case because, to be honest, it's done there and then. It's a very small part of the whole consultation. It's not something that you're going to take in physically, to be honest, so, no.

GP4

I: But that's interesting, isn't it? 'Cause it's an interesting balance between your expertise and the system giving you that little nudge, but it's also as you say there, learning. Do you think learning is part of the using of the system?

R: I think it must be at some level, I think it's just through…because it's giving you that longer term sort of behaviour change, isn't it, because if I didn't have that, what would happen is that I would just default back to my usual behaviour, so I wouldn't have learnt at all. Whereas this way…so some things I will have learnt I guess, then I'm no longer getting the (the CDS) prompt, and the things that I'm struggling to learn keeps keeping poking me, so at some point that will hit home I guess. Or I'll carry on getting the prompt. Either way, my prescribing will have improved I think.

GP4

I: Do you find here you get many alerts from it at all, is it kind of…?

R: Yeah, a fair few, yeah. Whether it's 'cause I'm not learning the lessons 'cause I'm not as fast as some of my other colleagues, but it just reminds me in a way ingrained behaviour can be and how you can really default to things that you've done for a long time, and even if you know at some point intellectually that you should be prescribing Amoxil at the moment, obviously when I'm not thinking about it directly what I'm tending to do is just default to things, Amoxycillin, and then the thing comes up, so…so even if you were interviewing and saying, have you learned, do you understand that you're supposed to be prescribing Amoxil instead of Amoxycillin, I'd have probably said, well, yeah, I know that. It's the fact that this (the CDS)thing keeps coming up shows that I do need the prompt.

GP4
I: And going slightly back actually to the education bit, do you think it's…the learning effect then, that has…'cause that might be changing the way you work, is there a learning effect there at all, or…?

R: There might be a learning effect but I don't know that that's changing the way I work. I suppose it's perhaps changing the end result, in the sense that my prescribing on those occasions when it's come up to prompt me will be more accurate than it would have been, that some of the time that's not going to matter because it's going to be…I'll have saved a few pence on some Amoxil. Other times it will be more crucial. In terms of the process of me working […] I can't see that…[…] can't see that there's…I can't put my finger on a tangible change to that really.

GP4

R: Yeah. I suppose that was the other strand we talked about last time. The educational value of it.
I: Precisely, yeah.
R: I think it must have done, yeah, because I think these things all happen. You know I think it's very easy in general practice to not know where your blind spots are because you can, generally even if you do something which is not quite right and not according to the guidelines, most of the time you are going to get away with it because fortunately bad things tend to happen reasonably rarely. So, even if you described Nitrofurantoin to somebody whose EDFR is less than 30, for example, well they are not likely to come to any great harm because of that, it might be that their UTI doesn't go away as well as they hoped or as quickly as they hoped or they might end up on another antibiotic, there might be some outcome from it but it might not be immediately obvious, so it's quite easy to sort of continue to compound errors, you know, by having them in a blind spot. And I suppose all this is doing is it's just making sure that things that you might have been in the habit of doing which weren't quite right, you know, just pinged up…you start to notice them, you start thinking, well hang on, that didn't come up before when I tried to do that. So, even though it's very difficult for me to give you an example and say, yes I've put this in my appraisal because you know that's the definite learning point that's being addressed, I find it difficult to believe it hasn't improved means of prescribing.

GP 11
I: Yes. Well, that's really interesting. Also within that then is, where's the learning effect with either of those approaches?

R: Yes, that's interesting. Yes, I think there certainly has been. And I can think of that with audits in general, you know, learning that still continues to change practice afterwards. Yes, I know I said prescribing is different after PINCER than it was before. So, in terms of these not these open ended 84 tablets per 28 days for the rest of your life things or at the very least do a script note of, please review after X number of months or something. So, I think that has been something that's changed with that. So, I think that's something with audits in general. For something with Optimise, it's probably sort of the change of guidance that keeps happening. I think that's really useful. So, something like Nefopam. That's something that I've changed because of that reason. So, the fact that it flashes up as Amber 2 every time I try and do it. And I still sometimes do... I think it's worth overriding that CCG rule because there isn't anything better but actually, it's useful that I understand that this is relatively cost-effective badness that I'm chucking out.

GP 11
I: Yes, and in some respects at, I think, the CCG level you can see acceptance and hit rates and alerts and so on. You know, and I've asked people at a CCG level, do you think it's fed back to GPs? Apparently, it's not.

R: No, not for individuals.

I: If you could see... Say, for instance, an alert and then you said, well, this actually was fired 25 times in the last month for you and you've accepted it on five occasions, how would that feedback actually be of benefit? Or would it just be another irritating thing to get in the way of work?

R: It's interesting, isn't it? I wonder if it's how it's delivered. I think the CCG pharmacist we've now all got a lot of respect for. So, I think honestly probably the initial thing would be GPs in general would get defensive about it and say well it doesn't matter because…. But actually, if there's a CCG pharmacist who knows their stuff and is respected, certainly like ours is, she's got a good relationship with us all, then saying this is the reason why and what can we do about it? Because at the moment, our pharmacy-based meetings are quite financial often and you can very easily tie that in as well. So, she's mentioned the nefopam because of expense, for example, in those meetings but then tying it in with...all right, well, there are the Amber alerts and it's always ignored [voices overlap 27.44].

GP14
I: Yeah, and that actually links to something you said quite way back in the interview around learning. Do you think, I mean, do you think the system's got that potential to help your learning?
R: I think it's probably got a lot of potential to help learners learn. Me, personally definitely because we are always learning but for those with less experience particularly as trainees, and all the F2 doctors that we have here who aren't even in GP training, then that's going to alert them to lots of different things and registrars and F2 prescribing is far from perfect. So, we do de-briefs with them every day. We do notice at times they've perhaps they've not really prescribed as you might have done or where the evidence or the guidelines might suggest.
So, if things are popping up in front of them, I'd be interesting to interview one of them, then you might find they're getting more alerts and accepting, hopefully more of the alerts themselves. So, yeah there is definitely learning because I learned from them, I didn't know you could get that tablet cheaper in that form or whatever.

<u>Sub Theme 6ii: Learning, feedback and education</u>

<u>Software developer staff</u>

Software developer staff 1

I think a lot of it is about educating prescribers, getting learnt behaviour so they know what to do. There is some element that GPs don't like to be told what to do, especially by a bit of software, they know best. And very more so in the smaller practices, your older GPs, they don't want to be told what to do, they've been doing this for 40 years, they know what's best. Often, they're the worst prescribers. However, but in some ways, because it's not directed at them as a person but as an organisation, they realise that it's not picking on them. Because, you know, medicines management team will come round once a year to do an annual prescribing visit and will say, bring some graphs, there you are in [quinolone 0:11:11] prescribing, that's awful and that's all down to you; and look where you are on cost and this, and look where you are on your [SIP feeds 0:11:20] you're rubbish. Whereas we can just gently say, well, actually this person's not had a MUST assessment, so have they got swallowing difficulties, do they need ONS, their BMI suggests, you know, at 29 that they probably don't need oral nutrition. You know, it's to try and get them into a better way of prescribing, but it's just a way to remind them of anything that's new on the data that they just may not be aware of, a gentle reminder of what the traffic light status is of the products. And so, yeah, I just see it as an add-on to the medicines management.

Software developer staff 1
Yeah, I mean, that's what I always say at a pilot is that this is decision support software, it's a support to make a decision, it's not going you must do. It's always please consider or, you know, are you aware. It's always worded in a way to say, just have a think about, you know. So whether it's metformin and they've got CKD stage four, you know, probably wouldn't recommend this...nationally, NICE say probably don't use this, guys. They can if they want to and if they're happy for the patient, that is their clinical decision to do so. And no one…that's the thing we need to get across, no one's going to hit them with a big stick because they rejected it, it's just they may not have been aware or they weren't aware that the latest U&Es indicated, you know, that their eGFR had changed.

Software developer staff 1
I think a lot of it is about educating prescribers, getting learnt behaviour so they know what to do. There is some element that GPs don't like to be told what to do, especially by a bit of software, they know best. And very more so in the smaller practices, your older GPs, they don't want to be told what to do, they've been doing this for 40 years, they know what's best. Often, they're the worst prescribers. However, but in some ways, because it's not directed at them as a person but as an organisation, they realise that it's not picking on them.

Software developer staff 1
I: That sort of brings us onto what we can have as the final question. What do you think builds confidence in the system?

R: Being listened to. So if a GP has fed back and something's been done about it, then that builds, because they just feel they're not being listened to. So I know, you know, I think some of my customers, if I have feedback a particular message, they might contact me going, can we make this a bit more specific because it's triggering a bit too much, can we add in this logic. We certainly can, shall we do that. Brilliant, let's do that. Or shall we just turn it off. And again, even if it's more generic, so even if an MM team has not done that work, we will do it for them at QRM and we'll go, right, in the last three months you've had 12,500 messages hit, 5,000 of those are just to this one message, which is accepted one per cent of the time. That means we do a number of things. One, we turn that message off; they're seeing it, they're not doing anything about it. Two, do you need some education to your prescribers of why the message is there and why it should be listened to. Or three, do we change the logic so it appears less frequently, and what can we do about it. Because then, all of a sudden, that message stops appearing, the GPs think, oh, thank God that message isn't appearing anymore.

Software developer staff 1
So, you know, I do know in the (name of place) area, we're really trying to tackle trimethoprim. So how can we do that? Well, we can have a message saying, you know, with a UTI, try nitrofurantoin or this. But then it's like, actually do we have a message if they try and prescribe trimethoprim, because that's not part of NHS England. But there's murmurings that perhaps we should have a message to say, look, we really…I know you love trimethoprim and we've done three days of 200 milligrams BD for years but actually that needs to stop now. Because GPs just need…sometimes may need a gentle reminder but they might not want that message, like, I know but I've picked it anyway.

Software developer staff 2
R: That's to do with insulin passports, and they wanted to make sure that all patients had them. They said could we produce a message, and we said, well, yes, we could, that fired when the patient was on insulin. But we've kind of steered against that because it would over alert. So what we actually did was we did a huge education campaign. We did create the message but they put it what we call a muted state where it doesn't actually fire and present to the prescriber but it can count how many times it would have appeared if it had been switched on. You can kind of see the size of the population they were addressing. And then they did their education, lots of passports were handed out, then they switched the message on and a sort of catchment at the end if you like to pick up the people that hadn't been addressed, and the data. It just supplemented a piece of work that they were doing. (Name of CCG) have done some work, again quite bespoke work, about patients that have had a splenectomy, around spleen and they have specific needs around antibiotics [inaudible 0:10:25].

Software developer staff 2
I: That's really interesting actually, because that's not using (the CDS) as an acute prescribing position is it? It's almost more old fashioned if you like. We'll do an audit, we'll find out what the problem is. But then rather than doing traditional meds management work on that then using (the CDS)afterwards…it's really interesting.
R: Well, they made a lot of the changes and picked up the patients that had gaps, but then they've put in (the CDS) afterwards almost as a safety blanket to say we know we've got to a good position and now we're going to use (the CDS) to…
I: To maintain that.
R: Yeah. And they've raised everybody's awareness of the issue. So when they see them they'll understand them. Whereas if you'd just put them out without all that background work they might not have been well received. And I think they haven't actually fired very much. They might see an uplift, like influenza I think. I haven't looked, but that's what we're anticipating.

<u>CCG Staff</u>

CCG1 Pharmacist 1

So in a way the tool is quite useful because we can guide [inaudible 0:19:48] formula recommendations, red, amber, green, do not prescribe, et cetera, but in terms of actual usefulness about so if something has happened, you've prescribed some of this, the ability to go in and look at that individual clinical record needs both the CCG and practice based link so that that information can be obtained directly by the pharmacist or technician working in that practice and be part of a learning time discussion with the GPs in the surgery.

CCG1 Pharmacist 1
some trainee pharmacists working with that whereby they can actually get all this data off and then go and look at what happened, and then use perhaps some of these as learning opportunities for GPs whereby well, we know you did this, you authorised this, we just want to discuss this with you

CCG1 Pharmacist 1
we're looking at PPIs and NSAIDs both of which can affect renal function, and so you shouldn't be giving these combinations to people with long term diabetes or hypertension, et cetera, because they can further exacerbate renal deterioration. Now, if you're not picking that up through the system or it's there but you're ignoring those messages then that might need a further elaboration of that, this is why you're getting the message and yet this person's EGFR is 22, why are you still giving this combination to this patient when we know that this can affect it. You've had the opportunity at repeat medication review but you didn't pick it up, why is that? So drilling down to that unique opportunity I see as probably the next stage of implementation of this.

CCG Pharmacist 2
The thing with (the CDS) as well, I can't remember if I said this last time round, is that…and the whole thing about PINCER is it's a snapshot in time. Unless you do that search again for new patients who might have come through, those patients could slip through the net, whereas (the CDS) is there at the point of prescribing for every single prescription for that patient who… Do you know what I mean, say it could be somebody new comes along and they're like, oh, they're on an NSAID and doctor x had seen them before and hadn't considered giving them a PPI. Have they missed that alert or do they need a bit of training and educating on that front? I've picked it up now so I'm going to try and do something about it now […] So it will be a trigger all the time and as we know as well, the thing about GP systems and I know learning from incidents that have happened, is that sometimes some things just fall off the prescription new list. Sometimes it could be like something was issued as an acute and then it's never got onto the repeat or a patient stops ordering x. You know, they might be like, oh, yes, I need that. I need naproxen for my pain but I really don't like taking omeprazole because it makes me feel ill. Ever since I've started taking that I feel really hmm. No, I don't really need that but my pain's better so I'll just carry on with that, so they may not order that and it might fall off the repeat list or the [inaudible 34:03] says they don't need that, they're not ordering it. Hopefully not because they'll see the benefits of them being on a PPI and hopefully have that conversation with them but you can see how things get taken off.

CCG Pharmacist 5
R: I hope so, yes. No, there would be so it reinforces a message but if the GPs don't understand a message, they might reject it and assume that that just doesn't apply because I've never heard of that before. Whereas if it's accompanied by a, oh, no, you must give a PPI for this reason and then that message comes up, it just reinforces what they've learned and they're like, oh, yes, I remember.

I: Yes, from the PINCER approach.

R: So yes, I think if they keep seeing the same message over and over again it, hopefully, would trigger something.

CCG Pharmacist 5
I: I don't know whether it was yourselves or whether it was somewhere else, people were saying that, sort of, usu..Yes, are the CCG…either yourselves or some other CCGs were talking about the using (the CDS)for specific things. And I think, and maybe that's also, I've had that come down from FDB as well, have you done that, or have you thought of using it in that way?
R: Yeah. So, the example that a new type of message, I don't think we really twigged it was a new type of message, but splenectomies, so people who've have their spleens removed need vaccines to prevent infections in the future. And so, we've done a big piece of work in (name of place), where we've audited to see if practices do give those vaccinations. And it showed that, actually, they weren't always giving what they should. So, we wanted a message to check if somebody's coded as having a splenectomy, we wanted the message that (the CDS)check which vaccines they'd had and give a warning that you've got a splenectomy patient, they're missing this one, this one. So, they've done that for us, which is really helpful. So, it's backing up the work we've already done, we've already trained the GPs, they know that that's what they should be doing and hopefully, this is a reminder to actually, oh, yeah, I need to do that, I need to book that patient in. And it's clever enough to also pick up patients who declined it. So, you won't get a message if the patient has been offered the vaccine but they've said, no. So, we've given the GPs a raft of codes to use to say, no, you're not having that one.

CCG Pharmacist 7 and CCG Pharmacy Technician 2
I: And the other thing related to that, I mean, you mentioned PINCER and SMASH and [Pinga 45:47] and so on, some of those things have a sort of, you know, inbuilt learning part to them, PINCER particularly, obviously. Does…is…do you think there's a learning effect for prescribers from this, or do you think they just sort of see the alert and just go, oh yeah.
R1: No, I mean, I think that if someone sees something enough of the time, and it depends how often it's being triggered, then they do hopefully learn something from it. I suppose the other thing is, well, the differentiation between those other systems, and that is that, like, certainly with PINCER, you know, you need, like…it was based on the practice pharmacist being present to do it whereas this isn't, so it's just sort of director / prescriber. You're never sure how much they're kind of remembering but you'd hope that if they see something enough times, they will remember it. I don't know.

<u>**GP Staff**</u>
GP Nurse 2
I: How does it fit in with what you do in your work in terms of, has it changed how you do things? I suppose because you came in after the system was there, it's difficult to say, isn't it? Do you think it changes things?

R: Yes, I think it probably does, particularly for probably the clinicians that aren't as good at keeping up-to-date or reading minutes. That group of people that would just keep prescribing the same things. Maybe it does make them stop and think, oh, well, actually, not supposed to do this one anymore so yes, definitely.

GP Pharmacist 2
I: And the background, and why, yes. Do you think actually, just to finish off then, you know we talked about PINCER and the learning part of it, do you think (the CDS) has learning impact?

R: Yes, I think so.

I: In what sort of ways?

R: It's interesting to know why the prescribers reject things or ignore them or, you know, yes. I think that's probably not been fed back to them enough over the last, you know, year and a half.

I: Yes. If they knew their acceptance rates, their hit rates, what they were accepting, and so on. Yes, that might be...

R: Because you'd expect that there should be both a learning [fault 30:28] from (the CDS), but also a learning amongst the clinicians.

I: Amongst the clinicians, yes.

R; Yes, to go oh, why did you reject that, and why didn't I, or why are we all doing that? Yes.

I: Which is from building on, and it's sort of doing a...it's using Optimise, but then the PINCER learning stuff as well, isn't it really, that sort of approach. That's really interesting actually, because that is something which, yes, would be interesting about using the system.

GP1
R: I think it's…well, certainly the cost effectiveness is good, because we can't keep up to date with the prices of things, I guess it depends how regularly it's kept up to date; you do find some things that when you look into it and it's perhaps out of date a little bit. Safety warnings again, they are very…and it's actually educational because a lot of them you know the warning before it comes up because you've seen it so many times so you're already aware of it. So I mean some of the things I know now are because of it, so there is an educational element to it.

I: Yeah. So in that way do you feel it's changed some of your prescribing then?

R: Oh, definitely, yeah.

I: Your prescribing habits?

R: Yeah. Yes, definitely it does, yeah. And bringing up some of the risks as well, the long-term risks, fracture risks in certain drugs and things like that it's brought up, which you probably wouldn't have thought about at the time; so it's definitely beneficial from that point of view.

GP1
R: Yeah. The other thing that happens is, I mean for instance with the olive oil ear drops, it's actually quite annoying when that pops up because it's an extra click you've got to do to get rid of it, and save a penny; well, probably the time it takes [inaudible 0:10:46] work it out, the GP's time getting rid of it costs more than a penny, so it's not cost effective in that sense. But the way it does work educationally is that I would tend to write Arjun ear drops just to stop it popping up […] So you're anticipating what it's going to tell you, you write A-R-J and it will come up with the ear drops, and so you'd never get to see it.

I: So as you use it over time you're sort of…

R: You begin to anticipate some of its moves.

I: …working with it. Yeah. Precisely, you're sort of getting a step ahead of it. That's really, really interesting, isn't it, because that's like the dynamic of the tool is changing in terms of the way it's alerting people.

R: Yeah. And I guess unfortunately then it doesn't show up in the figures of how many changes it's making; and I think that's something that happened with the other system, that with that, that was actually easier to anticipate for some reason, I'm not sure, maybe there were just more drugs that we were just still changing then.

GP3
R: And now I've planned I go for what I think…I've got sort of used to how often it's going to come up and I've made a decision perhaps to go for one or two of the loose hanging first. I'll go for the really important ones.

GP3
R: It doesn't annoy me that way, no. I don't get surprised. The only intuitive ones is when I know it's going to come up and I just wish it wouldn't, and I'm going to override you anyway.

GP3
R: I don't think the patients notice, no.

I: The patients know anything.

R: Because I normally know when it's going to come up and I normally know I'm moving onto the next bit before. So I've worked out my plan before. Certainly here.

GP4
I: What do you think (the CDS) is actually there for? What's the point of it?

R: To save money, formulary adherence, and the safety are the two things that…so preventing prescribing errors really. Two or things. I suppose you could perhaps put education in there as well if GPs…education about formulary I guess at this stage. I can't remember to what extent they give you information about formulary choices for things like antibiotics. I don't recall whether…I don't know whether you know?

GP5
R1: More times than not, I'd just follow it through. Just 'cause, it's the time factor. But, you know, on the odd occasion, when I've though, oh, I wonder what that's about, that's possibly…'cause often it'll flag up something that's possibly a learning need for me.
[…] Which, would have been nice, 'cause if I can then go to the page, I can then make a note of it, and then go away and read up about it.

I: So, having that sort of evidence?

R1: Yeah. So, if you take the example of, I didn't know that beta blockers cause, or can make asthma worse. Oh, that's a doctor's educational need […] I can go away and learn. But then, of course, if it says that and then the link doesn't work? […] You've lost the opportunity, and that's for me, the safety issue, it's not the fact that it's just about that one prescription. It's about, here's a doctor who suddenly realised something he doesn't know, and there's no follow through on that.

GP7 and GP8
I: But going on to that learning thing, which gives you the best learning, does (the CDS) or PINCER?

R3: PINCER because that's the safety bit. (the CDS) is not safety for us, so PINCER is the audit, which makes sure we're prescribing in line with safety guidance.

R2: In terms of learning the new, it depends what you say you're learning doesn't it? Because if you're learning about patient safety stuff then obviously PINCER, but if you're trying to learn the vast, you know, formulary of what you should be prescribing then (the CDS) teaches you that because it just, it just tells you what you should be prescribing instead of that. Which, you know, realistically we can't, well I can't look up every single drug that I'm going to prescribe and check it is the first line on the formulary. There's little things that, the little icon that says it's on the formulary or not but if you've been asked to prescribe this specific thing by a consultant you go for that don't you unless there is like a generic, you know, if there's a generic version or there's a different drug that's exactly the same that's gonna be a lot less money, then that's why it's really useful when that comes up.

GP7, GP8 and GP9
R3: You try to shut down and it's not over.

R2: I was trying to shut down all these boxes and they were going if you're doing this you need to do this and then it was wiping the drug that I'd put in and…

R3: With regard to HRT there does seem to be a problem because they are coming up greyed.

R2: They are, yeah, I've noticed that. I've asked them, but I don't think it's all of the HRTs because actually when I looked at this particular one, it's not on our formulary. So it was to do with the fact that it's not on our formulary, but I don't know why?

Because what it actually said was, there is insufficient evidence of this drug. It is a grey drug and I thought, well there isn't because it's a formula of HRT, a formula, which is perfectly reasonable and the alternative that you're telling me I can prescribe is basically exactly the same thing with a different brand name. So I think it's actually a cost saving switch not a grey lacking in evidence.

R3: Because I've had that same problem…

R2: With HRT, I think that is the problem.

R1: Yeah, there's a couple of them isn't there?

R2: But it's, you know, it's a few extra clicks but I do think it helps with safety and I do…

R1: I don't think it helps as much.

R2: …think it will be saving loads of money.

R3 And I suppose the rationale is over time you should be getting fewer and fewer clicks because you remember…

R2: To not prescribe that thing

GP 11

I: So, how does it work within the consultation then? Is it something that's got seamless or it's just there or, what?

R: I think it is useful because... I think often changes have happened either from a cost effectiveness point of view or from a, the research base has changed in some ways. And actually, maybe that doesn't filter down, even to all of us, at partner level but particularly to all of the training doctors. And I think that makes a big difference. So then, it's not uncommon that we'll get, either during the consultation or after, "hi, I was going to prescribe this, but an alert came up, what do I do?" And we'll have an alternative conversation about it [...] So, I think it does change the outcome of what the practice on the whole does.

GP13
R1: So, I think so it's the other side of the argument then, how do you capture, you could easily observe my day, and work out how (the CDS)affects me when it's there, but it will be much harder for me to see how it affects me when it doesn't fire. Yeah, that would be harder, and whether I am subconsciously thinking, this is okay, 'cause nothing's warning me it's not [...] I've at least one example of that. We had an elderly lady in a residential home, who was on 40mgs citalopram for her anxiety, and I think a medical student, or our pharmacist did an audit, or something came from the MHRA, I can't remember, anyhow it flagged up, we ended up doing a search. And it transpired that I had a knowledge gap, and I wasn't the only one, that 20mgs was the maximum dose you should be on, if you're over a certain age of citalopram. Now, as far as I know, (the CDS) had never told me that, and (GP CLINICAL SYSTEM) had definitely never told me that, because I went in and tried it. And she'd been having that for some time, without any, and I'd been signing her repeat prescription. So, I guess my behaviour had been, there is no warning, it must be okay.

I: Yes, I'm with you. So, yes, and in some respects, that's really the other side of these alerts isn't it? If you're not getting them, your assumption is, everything's fine, I can go ahead, I can proceed. But it might just be that the messages haven't been set up.

R1: Yeah, and that's harder. And in fact, then what happened with that lady is we went and had a discussion with her, saying, you need to reduce this, and she and her attorney said, really, can we not just stay on this dose, because last time she tried to reduce it everything went mad? And we sat down and had a discussion and did the whole balance risk thing, and she stayed on 40 in the end, which was interesting. But that was then one of those managed risks, rather than, if a month earlier she'd had a cardiac arrythmia and died, that would have been a mistake.

I: Yes, I'm with you, yes, yes, precisely.

R1: But it then became a managed risk, which is okay. A managed risk is fine in medicine as far as I'm concerned. You know?

GP13
R1: And I can't tell you, if the prescriptions I signed before that audit, it had flagged up and I'd ignored it, or it had not flagged up at all. I just don't know, because it's now so good, in terms of not bothering unless it needs to, but you don't notice it if it doesn't [...] And you wouldn't see those things unless there's those sort of audits.

R1: Exactly yeah. There must be some sort of analogy with type one and type two error, the type one errors are the things it makes you do when it flashes up, and the type two errors are things you never know.

I: You never know about, yeah.

R1: Because it doesn't flash up.

GP13
R1: So, I think, (removed potentially identifiable material) but the cost side is probably more important on a day to day basis, than the safety side. But it's nice to know that, we sort of have a mix now, with the (GP CLINICAL SYSTEM) flags up every possible little safety problem, but if (GP CLINICAL SYSTEM) and (the CDS)agree, then you probably really ought to look at that.

I: They shouldn't duplicate? If one fires the other one shouldn't, but it probably does, I have no idea.

R1: I couldn't say if that happens.

I: I think that's what we've been told, as to how it operates, but…

R1: Right, okay, it may well do. I mean, that leads to an interesting point, (the CDS)seems to work without the need for a vast amount of cognitive input into it, it's there when you want it and…well maybe it isn't. When it's there, you think, okay, I'll think about that, when it's not there, and I'm thinking as I'm talking, but maybe now if it's not there, then we assume everything's okay, and that could then be a hole in the safety net, I suppose, it could be one of the holes in the Swiss cheese, that's waiting to line up with a couple of others. [...] So yeah, maybe it's almost like we rely on it too much.

GP13
I: …and it was the thing I wrote down to come back to. What sort of learning effect is there from Optimise?

R1: There's definitely some. So, the current change, the sertraline, citalopram thing is a good example of that. I've become aware that whereas citalopram was a first line SSRI, it's now sertraline, and I've become aware I don't know why, and so this is in the last month, so at some point, I'm going to go and find out, is that a cost thing, or is it a clinical effectiveness thing? And once I've answered that, I'll then know a bit more about sertraline and citalopram [...] So, it has a genuine sort of CPD learning affect. It has a strong learning affect as to which is the cheapest brand of progesterone only contraception at the moment.

I: Because you…?

R1: Because it tells you, you know?

I: It tells you, yeah.

R1: I suppose, that's interesting, because I know that it tells you to prescribe a particular brand of progesterone only contraception, I know that's for cost reasons. The fact it's telling me to prescribe sertraline and not citalopram, I don't know if that's cost or clinical effectiveness yet. But either, I've learnt something, once I've filled that educational need, it's learned for a bit, until it changes back to citalopram in six months' time, but that's the

GP13
I: But, in comparison to the PINCER approach, that learning is different isn't it, it's a different type of learning?

R1: Yes, it is, 'cause it's a sort of…the PINCER approach is learning too late isn't it? 'Cause you've already done it wrong. It's like a Minority Report type of learning, you know, intervening just before the crime occurs, which has got to be a good thing really, as long as it's programme correctly, and like all tools, it can be used for good and used for evil, so as long as I trust the people who are programming it, and they're using it for good, and they're not being sponsored by the sertraline manufacturers, then it's a tool for that. So yes, a lot of learning and prescribing senses, but again it's the type one, type two argument again, it promotes the learning, but it might encourage complacency about the stuff it doesn't [voices overlap 00:39:44]...

THEME 7: Reflection and monitoring

Sub-theme7i: Using (the CDS) as a feedback system

Software developer staff
Software developer staff 1
R: Yeah. We'll keep an eye on…we used to have a really good report that pulled off good practices, and we can't get that anymore, that was always really useful. But what we will do as part of our meeting is, I'll pull off a list of their contract, so who should have it, and then I pull off the report of who's turned on, and then I can see who's turned off, who's turned on, who's merged. Because sometimes it looks like someone's turned off but they've actually just merged a practice, so it's not as bad as it always looks.

I: Yes, which is happening quite frequently these days.

R: Yeah, oh gosh. All those small single-handers are just disappearing, yeah, and they're just getting sucked into somewhere else. And that's always part of the work that we do, we will highlight any practices that look like they've either disengaged. But then we can use the reporting data to do that. So if someone got a really high rate and then a really low acceptance rate. Nine times out of ten, when I take that data to the team, they'll go, oh, I know which practice that is. So where I was yesterday, there was one just like that and they were, like, oh, it's a dispensing practice, that's why they're not accepting anything. Which is frustrating for them, because we've only looked at the best practice. Now best practice is nothing to do with brand A, B or C. […] And there was also rejection. So it's like what can we do to engage with those guys, what can we do to help. And that's the type of things we'll also help with.

Software developer staff 1
I: And I was at a meeting recently where I was talking about our evaluation, and someone, I think it was a GP, said "too many alerts". And that's…is that something you hear or is that…?

R: It's a difficult one because there's not only our alerts, there are the inbuilt CDS. So (GP CLINICAL SYSTEM) have obviously a whole lot of alerts as well, and we will then…so there'll probably be a CDS alert, then we'll appear and they'll just be like, bloody hell, it's another chuffing box. Obviously, if it's in (GP CLINICAL SYSTEM), our message will not be the same as what their message is, at least that's, I think…again, if they knew that, it's just like, oh, you know, so they don't ignore it. One of…you can always…sometimes counter argue that if they'd prescribed the correct thing, then they wouldn't see an alert, which really gets their goat up. And you can…

Software developer staff 2
R: Yeah. So there's little things that [inaudible 0:24:03] the little things add up to this. So one of the things we try to encourage our teams to actually actively seek feedback from their end users. Often it's kind of like, well, if they don't say anything then it must be okay, rather than actually proactively prodding and seeing if there are things that they can improve on. So challenging them really about whether they've got mechanisms in place, maybe doing an ad hoc survey, although they tend to be self-selecting in that you just get a bit of a Marmite effect, you'll either get the people that love it or hate it or in between that respond. Or making sure that if they've got teams based in practices, if they hear things on the ground that they know how to feed it back, and also kind of close that feedback loop so if they do get any feedback and they make a change actually feed that back. So (name of CCG) are quite good at that, they try and actively promote that they might have turned off messages that were highly alerting, and it gives the practice a feeling that it's not just something that's being pushed at them and they're not interested in their experience of it. So that's one thing. Tinkering with messages, so turning off ones that are highly alerting or deciding to do some education around them or asking us whether there's something we can do to make the message more specific. Internally we're also looking at that. We've got a mini project going on about the wording of messages, can we make them more succinct. We realised that less is sometimes more.

Software developer staff 3
R: So, the idea of a report that says actually, looking at your behaviour, it is possible to say that, because some of the activity you may be seeing from what's been prescribed or what's being dispensed, may be attributable to the impact that that RX is having.

I: Yeah, I'm with you. So, there's two things there isn't there? There's one that's sort of saying, 'cause the actual, I mean the hit rate, it could well be as you say, it could just simply be that they're getting better at prescribing. But in terms of, also there, you were talking around grouping things around. And that grouping would be around disease specific things, or just specific groups of drugs would it?

R: Yeah, it could be, it's probably going to be more condition specific, so allowing, so yeah, the functionality will be group any of any, but the use case would be for a CCG or meds management team to say, right, we're focusing on nutritional products, we've got a big drive on that, so we want to group these together and be able to understand what's being used, what's not being used, what do we need to tweak et cetera. So, for me, when they come to report their activity, and their success, they don't do it on our basis, that's how they view the world, they look at condition specific, or, as you said, drug area specific.

I: Yeah, precisely, things like antimicrobials, yeah.

R: Exactly, yeah.

Software developer staff 3
R: And they turn everything, and that's fine. And them you'll get your reporting usage, and then they can tweak or adjust as required. You'll get other customers who'll go, well we were using the other system, and all we want to replicate is the message from the other system, and put that in place, 'cause that's all we care about. So, what you'll get there is customers who are very cost focused, but don't really think about best practice, or safety. And then there's customers who are really clued in, and say, actually we just want to turn on three - four hundred algorithms, and it's because it aligns to x. y and z in our plan, and we want to track and monitor, and see how successful we are. And really we want to get everyone on to that last course, so they're putting some sort of consideration into why they're switching something on. [...] So, there's this concept of profile management, and profile optimisation so we're actively working much more closely with our AMs, to make sure they are, one, that they understand, well, they're making sure customers understand which messages deliver what type of value, and also with that, hence the grouping bit becomes important. So, if you have to understand, your focus is on this I see, from your strategic priority, or you've got an incentive plan, so these are the messages you should be grouping together, and then we'll show you how those are being adopted.

Software developer staff 3
R: I think the biggest problem, well one of the biggest problems, is the maturity of the CCG itself.

I: Right, yeah.

R: So, some of the best, again, I don't get out that much to talk to everyone these days, but the best CCGs are the ones who've got a clear meds management, meds optimisation plan.

I: Right.

R: What are they trying to achieve? They've got a clear cost target, and they've also got a very clear outcome, or outcome for different areas of focus or work.

I: Yeah.

R: 'Cause in some ways, (the CDS)is a change tool, in a lot of ways. It allows you to understand where you are now, and where you want to get to, and by putting in these groups of messages, you can impact change, and we give you the ability to measure that change, or to track that change.

So, if you come to this tool, just thinking about it, as, I want to save as much money as possible, and put in all my cost messages, apply them, I mean that's what you're getting out of the tool, and we can give you that, but what we're finding it, the ones who come to us and go, actually, we know that cost savings are low hanging fruit, we've been doing it for years, we just need you to continue that, but we want to focus on these areas. And I think the market are moving, I think costings are moving. But, it takes quite strong leadership and direction for that message to get from your heads-of down to your pharmacist and your technicians, who are working in the teams or in practices. And so, it's very clear when you go and meet somebody at MMTs, which side of the fence they lie on, or have they got a clear feeling for what their priorities are?

CCG Staff

CCG Pharmacist 2
I: I mean you've talked about, we talked around, over what sort of acceptance rates you were getting on messages with the practice, has that changed at all from…?

R: Our acceptance rates are exceptionally good. You know, so we're above average.

CCG Pharmacist 2
I: Right, yeah, yeah, and in terms of acceptance rates, is it acceptance rates for cost saving or safety or both then?

R: I would say both.

I: Right. And do you think the GPs, the practices are sort of, do they get how, what the point of the system is or do you think, I mean what sort of feedback do you get from GPs in that respect?

R: Well I don't really get a lot of feedback, which, to me, I always see that as a good thing because if there was negativity out there you tend to get those kinds of things…

I: Yes, they tend to be, yes, quite.

R: Yeah, I think I could probably in the future go out there and maybe promote it a bit more but then why bother when our acceptance rate is above average and we've got so much more other things to be doing.

CCG Pharmacist 2
I: Just thinking in terms of some of the stuff we said last time, we were talking last time around your role and some of the communication with and from GPs. One of the things that you mentioned was the reporting of medicines, for instance, from primary care. Is (the CDS) helping at all with that? Has (the CDS) been able to help with any of this reporting back from GPs about…or have you been able to look at the hit rates of things as well?

R: It's a good question. I haven't actually investigated any hit rates as much as maybe I could do. I don't know what it would show because I think isn't there something, I'm just trying to remember now, when it comes up… Unfortunately, see, I don't have access to the clinical systems, I've only got access to (the clinical system) demo and that's not linked into (the CDS) so I can't actually see them come up and interrogate the system and see when they come up and what it looks like. So I think some people have said when an MHRA comes up I think… I can't remember what the screen looks like but you either can accept it or reject the message or something like that. I can't remember. I think some of them are rejected but I don't know what the… I think it depends on how good that data is, do you know what I mean? […] It's almost like, how good is that data? What does that actually mean if they press reject? Did they mean to or is it just because it's on the wrong side of the mouse, do you know what I mean?

CCG Pharmacist 2
I: Yes, I'm wondering, one of the things around the…it's, sort of, where I was coming from there was around the alert fatigue. The other side of the alert fatigue is that if GPs are getting more confident around…feel better about getting the (the CDS)alerts if they're relevant and timely and they're thinking, oh, yes, that's helped me there, does that then actually make them feel more…? Is that being reflected in GPs being more happy to report back to you or to be more accepting of alerts because they're getting those relevant ones?

R: I think there was a survey done locally about what GPs thoughts were on (the CDS) and I think the feedback that came back ranged from, we don't like it to, yes, it's great, but the average was in the middle when you looked at the number crunching and stuff. One of the questions was, what do you think of the safety alerts and, again, taking the mean average, it came up to about 5.3 on a scale of 1-10 or something like that […] So you do have some GP practices that love it and then you've got some that are like, oh, we don't like it and we'd like to switch it off if we could. Then you get some that like, the example in the morphine case, the Zomorph case, you do then get some GPs that say, oh, can we get an (the CDS) message to try and help fix this? So they will try and…they'll remember it and go, oh, that might be a good way of…you know, so they do think about it when it comes to safety stuff and try and…. Usually via our team, they'll say, oh, can we have an (the CDS) message for this? It is considered.

GP Pharmacist 2
I: Yes. So you're not part of the profile management at all now?

R: No. So, (name) is part of that, she is like our key, I suppose, our link. So, whenever we talk about Optimise, (name) talks about that in the meetings. So, she must be the key (name of CCG) with (CCG partnership). But my understanding is that we just need to use it in practice, and I'm not updating the profile or anything. If you wanted to go and look at why, you know, clinicians were ignoring messages, or whatever, you could. But that information will be fed back.

GP Pharmacist 2
I: And the background, and why, yes. Do you think actually, just to finish off then, you know we talked about PINCER and the learning part of it, do you think (the CDS) has learning impact?

R: Yes, I think so.

I: In what sort of ways?

R: It's interesting to know why the prescribers reject things or ignore them or, you know, yes. I think that's probably not been fed back to them enough over the last, you know, year and a half.

I: Yes. If they knew their acceptance rates, their hit rates, what they were accepting, and so on. Yes, that might be...

R: Because you'd expect that there should be both a learning [fault 30:28] from Optimise, but also a learning amongst the clinicians.

I: Amongst the clinicians, yes.

R; Yes, to go oh, why did you reject that, and why didn't I, or why are we all doing that? Yes.

I: Which is from building on, and it's sort of doing a...it's using Optimise, but then the PINCER learning stuff as well, isn't it really, that sort of approach. That's really interesting actually, because that is something which, yes, would be interesting about using the system.

CCG Pharmacist 2
R: Yeah. So, I mean, as I mentioned before, anything, like you've got the national picture, so anything that comes out nationally, that you can try and help support our GP practices at that point of prescribing, can (the CDS)help with that? Is there something already in the system that can already help as a block barrier in that? And then, anything like, regionally, if we feel, hang on a minute, this might be, if you're having a problem in Derbyshire, we're having a problem in Nottingham, then it's that building a bit of a picture about that.

I: So, there's an angle there, around not just about how it's beneficial to prescribers in practice, in terms of actually, oh perhaps I shouldn't be doing that, but by generating reports? Is that something which is useful?

R: Yeah, I mean to be honest, I've not got to that point yet, of actually interrogating (the CDS), to try and get a picture of what, is that what you mean, trying to get, so the hits or whatever it is, and being a bit more meaningful information like that.

CCG Pharmacist 4
R: I think where we perhaps haven't maximised is with the reporting aspect. So the reporting aspect has historically been quite clunky, and I think there is a realisation within higher levels that we've got all this information, we're not doing a lot with it yet, we're not sharing a lot with practices. That's a double edged sword because they don't want too much information. But certainly I've shared with them top five messages, ad hoc, but here's your top five messages that are triggering, here's the top five that you're accepting, here's the top five you're rejecting.

CCG Pharmacist 4
R: One of the things I, and we've, been doing is looking at what their acceptance rates are. So that is something that we look at quite regularly. Historically we've certainly been higher than the national average for both the acceptance rates for the safety messages and the cost saving messages. We're perhaps on par now. So the acceptance rate is pretty much around the average for both. But that's probably what you'd expect as changes get made and prescribers get used to it really, I suppose behavioural change. What is noticeable is our hit rate is quite low.

I: That's quite good.

R: So we have perhaps got fewer messages enabled than other CCGs, but certainly now…I've not looked at south Derbyshire's recently, but certainly Erewash's hit rate is only about five per cent. So when people do moan, and they don't moan a lot about it, it's only one in 20 consultations. So that's two or three consultations a day you're going to get a message. And actually if you're getting more than that then there might be something you need to think about with prescribing. Certainly some practices and some GPs say they never see it because they probably don't need it because their prescribing's good. So sitting there and watching our prescribing is pretty good, so I think that does explain again why the hit rate's quite…

CCG Pharmacist 4
R: This is what we do as a group. So we didn't do this yesterday, but last time we met we looked at that and we did switch on some messages that were firing a lot and not being accepted. Equally there were some there that…

I: So they were ones that were muted but…

R: No, we don't use mutes. So we've made a decision not to use mutes, so it's either on or off. Because mutes do slow the system down, and there's almost not much point really. So it's really looking at printing the reports off or reading the reports and see which messages are firing the most, what's the acceptance rate, do we need that message or not. So what is the value? So one at the moment which I fought to remain on is the aspirin in primary prevention. That's by far the most popular message that's firing for safety. It's got 2,000 fires. But it's not been accepted very much. It's four or five per cent. But I think that needs to stay on, because why are we prescribing a drug just because we've always had it, because it doesn't do that much harm? Well, it still can do harm. One in 250 people a year. So I think that needs to stay on. But some of the less contentious ones do get switched off.

CCG Pharmacist 4
I: Right, okay, yeah. But you say generally perhaps you feel people are generally happy with it or…

R: I can only speak for the practice that I work in, obviously, so it's only a fraction of the CCG, and I am quite lucky I think, my practices do tend to be quite good practices, and I don't have any of the really more outspoken perhaps prescribers. And they're quite happy with it, yeah. I've had nobody saying I want it switched off, basically. I guess being on the group can help because I can give them information. So if they're saying this message is annoying me I can show them straight away, I can actually pull up what their hit rate is and that can be quite useful, and if you compare it to other practices as well. So that can be powerful really.

CCG Pharmacist 5
R: Yes, so I checked our acceptance rates. We've got about a 23 per cent average acceptance rate over all of the messages. The safety messages, so the best practice and safety messages, have got the lowest acceptance rate in (name of place). They're only 16 per cent which sounds awful but I think that's fairly normal for (the CDS) to have quite a low acceptance rate. I think 20 per cent is about their average, yes.

I: It's round about that, yes. The, sort of, teens, twenties, yes. I think, yes.

R: I must admit I was quite surprised when I saw that the safety ones were the lowest one. Then the formulary and the budget messages are both about 25 per cent acceptance rate which is higher than the national average which is quite nice to know. So, to me that suggests we need to do a little bit of work on which best practices and safety messages we've actually got on and to try and figure out why they're being rejected.

CCG Pharmacist 5
R: Yes, so some of them are quite angry messages like, 'no way' or, 'I've done it already'. Some of the messages it's just a case of what order they did things in so if they prescribe a drug and then it says but you need a PPI with that, 'I was going to do that', say some of the messages, 'that's what I was going to do next'. So, some of it is irrelevant, really, and we can ignore those ones. Other ones, some of them, I don't agree with this and those are the ones that we want to look at.

I: Yes.

R: A lot of them, 'patient doesn't accept this'.

CCG Pharmacist 5

What else do we do? We've started to look at rejected messages so (name of place), because they've been established for longer, they have a really good system where they review their rejected messages every month and look at the reasons that the GPs are giving. Some of the reasons aren't really appropriate but some of them are really insightful and we're able to see, oh, that message just isn't working because they can't action it because of x, y or z. So, then we look at those messages, we flag those messages up to talk about in the meetings and turn them off or do whatever we need to do with them or change them if they're not worded properly. That's a good way for the GPs to flag up if there's stock issues with a particular brand that we're recommending and we can sort that out. So yes, we're finding it's flagging up more problems, not problems but issues than just the (the CDS) message. It's flagging up problems with the formulary that we can then take to the Area Prescribing Committee so it's really helpful and also the supply issues are useful because that's…

I: So, because your formulary's all embedded into Optimise, all those messages, then any issues there, (the CDS)is…yes.

R: Yes, so it's like a feedback system for our formulary as well which is…

I: Yes, I never thought of that, actually.

R: Yes, we use it like that quite a lot.

CCG Pharmacist 5

I: Yes, it's interesting so you can use the system not just in terms of cost-saving and safety but you can also use that as to knowing what's happening in practice and what's being prescribed and what's…

R: Yes.

I: Yes.

R: Yes, it's really good. I think it's nice for us… Well, it's probably not nice for the GPs but it's good for us to know that there's a monitoring system there. It's like they can't fly under the radar anymore by prescribing drugs they shouldn't be prescribing.

I: Yes, because you'll know.

R: They know that we're going to spot it.

CCG Pharmacist 5

R: I had a look before we came. I don't know about the hit rate. I know in (name of place) we monitored the hit rate because it was fairly newly established. I'm pretty sure when I was in (name of place) we started using it in (name of place) and we saw that the hit rate [interruption]. Yes, so the hit rate started really, really high and then came down and the acceptance rate, I think it initially went up and then started petering off as the messages… So easy problems to sort, we sorted and accepted as the system was new and the messages were new. Then as those easy to solve problems were solved, you were then left with a baseline of quite difficult problems so the acceptance rate fell off.

I: Yes.

R: I mean speaking to colleagues here, the same happened here as well so when they were…

CCG Pharmacist 5

R: Yeah. I think they could probably improve GP involvement. At the moment nothing's really changed, it's the same as it was. So, they give their feedback either by, well normally, just by speaking to their practice pharmacist, so there's a CCG pharmacist attached to every practice, and the GPs don't hold back if they've got a problem with Optimise, they do tell them, so that gets fed back to us. So, we've got a query log that all of our pharmacists can input into, so if they get a comment from their practice, they'll whack it on the query log so that we can review it. And then, GPs have the option of the rejection. So, if they're going to reject the message they always get the option to write a message to us and explain why, and we keep an eye on the rejection messages every month and just see if anything, if there's any common themes or any particular messages that are annoying people.

But yeah, I think, like you say, it's there's so many messages popping up, I don' think they always twig it's Optimise. So, yeah. And I don't...

CCG Pharmacist 5

I: I mean your acceptance rates you talked about there are, I think, pretty much average acceptance rates that people use and not just (the CDS)[inaudible 30:10], other things as well, but in some respects, it still means that four out of five or it means four out of six messages are rejected by… Why do you think that is?

R: I think sometimes it's, I haven't got the patient in front of me. I physically can't do anything about that so I need the patient there to talk to them. They might not have time to deal with it then and there. Some of them, they may well have accepted it in their head but don't want to do it now so they'll reject it or close it but do it anyway. It also fires for test patients so some of this could be people training or looking things up. It might be our team doing switches and because we're recording it elsewhere…

I: [Inaudible 31:05]. So, part of that is…yes.

R: …we'll reject the message because it will come up for us as well.

I: Yes, it's never going to be that high. It's never going to be like, you know, the other way around. I was thinking in terms of, again, linking that with the way a patient…you know, like I know patients don't necessarily like switches and things like that so it could maybe impact as well.

R: Yes, I think that GPs are becoming less accepting of switches than they were especially with the shortages that we're having with medicines at the moment. Prescribing by brand is becoming to be a real pain for them. So I can see that if Brexit does happen and shortages become more common, we may end up prescribing more generics or prescribing generically and undoing all of the cost-saving work that we've done in the past.

CCG Data analyst
I: And from your point of view as well, I mean, in terms of a feedback tool, what sort of learning from the CCG is there from having (the CDS)out there? What sort of things do you gain out of that?

R2: Well, I think because obviously we used Script Switch before, I think the biggest thing, and again, from the way that my role works, is again, if it is based on cost-saving, is that when there were too many messages firing, then people stopped obviously actioning them and the cost savings stopped. Whereas now, because obviously they've learnt from that, they've tailored it, and again, we can actually tailor it more now, there's less messages. So, yes, there are still savings obviously showing, which is great, but again, from a best practice point of view, people are learning because it's not the same ones in different practices, sort of thing, that are firing all the time, there are different ones. So when we check through the message, you might find that there's a big saving in one practice one month, and then again, sort of two or three months down the line, it might hit another practice, and it's that same message, so it's still kept on with it. Do you know what I mean? Because obviously that message is still needed, isn't it?

CCG Data analyst
So obviously we feedback all of the messages to the (the CDS) group to show them which ones are the big ones, and again, the main part of the work that we used it before was when we had the four separate ones, was to say, look, these are firing a lot more in this CCG than they are in this one, but, again, we don't sort of do that anymore because it's all one, which makes it obviously a lot easier

CCG Pharmacist 7 and CCG Pharmacy Technician 2
R2: Yeah, because there's other ways you can do that work.
R1: Yeah.
R2: And sometimes you need that human touch instead to them...
I: That's really interesting because that's…can you go into that in more detail?
R2: So for example if there's a switch from, say, modified release docs as a [inaudible 00:21:56] to the plain docs [inaudible 00:21:58], as a switch, that might involve…well, it is really involved, you have to check someone's blood pressure a couple of weeks after. So if you add that on as a switch a lot of people wouldn't be bothered to accept the switch because then they have to arrange an appointment and they've got to speak to the patient, whereas then I moved it into our technicians' work stream, which is what we have, the technicians, all the work streams we work on, so when it went into that, it gets picked up that way. So we can run off the list of patients and actually go and speak to the practice and say, we've found this, this is what'll be involved, do you want us to make the switches, send a letter out to say, you need to book it in two weeks? Or some GP surgeries have the blood pressure machine in the waiting room. So just having a bit more of a human touch to it…
If there are too many steps involved with a pop-up it's not going to work, or if there's something that it's flagging you need to review this, it doesn't always work either because, well, I'm not…I'm doing this…I'm in this consultation for something else, I don't have time to do this review. You know, so for things like that what we've found is, there's point having that message keep coming up, you need to review this patient, you need to… So with certain things, you know, like for example, inhaler reviews, it's not always the best way to flag that up.
I: So basically, the better approach is for you as a CCG to be involved in supporting the practice, identify those patients than doing…?
R1: Yeah, there's other ways of doing the work.

CCG Pharmacist 7 and CCG Pharmacy Technician 2
R2: There's just too much, yeah. Well, that's handy for us to see, because there are things you can raise from it, you know, or if you're doing a practice review. Say if you had a meeting and you see a lot of quality stuff getting ignored, there might be some that don't necessarily need a lot of work, but actually this shows you've got so many patients on these kind of drugs that could actually…you could add up a lot of falls and accidents from that. Just to take that to a meeting and highlight it that way is sometimes better than having it keep popping up.
I: Yes, but you're finding out about that because of the rejection and the alerts.
R1 & R2: Yeah.
I: So there's a sort of feedback system happening there, isn't there, as well?
R2: Yeah, because you can put messages on mute to see whether…how often it would fire, so you can practise things as well or see how things run in the background. So if a practice was…you know, it would literally…if it was popping up all the time and it was a safety message then that's quite handy to have that information, especially if there was like a CQC coming up or they'd failed a CQC.

<u>**GP Staff**</u>
GP3
I: Yes. And then see what you're going to do in the future in your next time you come to prescribe…

R: And it's in the past, does it matter?

I: …an NSAID for somebody who's 65. Whereas with (the CDS) you're not actually going to do that, or you might, but you might decide that the patient needs the NSAID.

R: Or they might need it at 55.

I: Yeah. Or they might not need the NSAID.

R: Yeah. It just makes you think about it in a fuller way. And it puts GPs in league tables. All GPs just strive to be in the middle of all the tables. They never strive to be at the top.

I: Right. As long as I'm doing okay.

R: Yeah. We were saying this yesterday, we were saying where are we with our training feedback, oh we're in the middle, that's fine.

I: Yeah.

R: Don't want to be very good or very bad, just want to be in the middle.

Sub them 7iii: Does (the CDS)work

Software developer staff

Software developer staff 2

We've also responded nationally to the product shortages that are affecting a lot of patients, pharmacists, GPs, when drugs are not available. We try to produce content to support where we know it's national and it's a very difficult picture because it's not always national.

I: Yes, quite.

R: But when there's been something that's come from the Department of Health or something… And we've had to try and really get to these people at Department of Health. Often some of this information that you think is public is sitting behind paywalls or you need privileges to access. So we've gone right to the top, (name) has, and spoken to the Department of Health and shown them what we do, and they're like oh, this is quite innovative isn't it, and quite useful, yeah, of course you can have the information. So we've tried to get that flowing a bit better so that we can respond quite quickly. It's only part of the story for GPs, but hopefully if they see a little alert, they're about to prescribe something that might not be available it can at least prompt a conversation with the patient to say, I'm prescribing you this, you might not actually be able to get it at the first pharmacy, would you try a couple more, and if you can't get it at all speak to the receptionist and I'll have made a note of what else to give you. Rather than they get frustrated when patients come back, they can't get stuff and things, at least if they're forewarned at the time of prescribing they can maybe adjust their plan.

Software developer staff 2

I: That's really interesting actually, because that's not using (the CDS) as an acute prescribing position is it? It's almost more old fashioned if you like. We'll do an audit, we'll find out what the problem is. But then rather than doing traditional meds management work on that then using (the CDS) afterwards…it's really interesting.

R: Well, they made a lot of the changes and picked up the patients that had gaps, but then they've put in (the CDS) afterwards almost as a safety blanket to say we know we've got to a good position and now we're going to use (the CDS) to…

I: To maintain that.

R: Yeah. And they've raised everybody's awareness of the issue. So when they see them they'll understand them. Whereas if you'd just put them out without all that background work they might not have been well received. And I think they haven't actually fired very much. They might see an uplift, like influenza I think. I haven't looked, but that's what we're anticipating.

CCG Staff

CCG Pharmacist 3

I: Are you able to see therefore, see how those costs, so how much if you [inaudible 0:11:59] (the CDS) is saving across the CCG each month? Is that what you're saying?

R: Yes.

I: And how helpful is that?

R: It helps us to monitor, because we have a QIPP amount attached to it, so we're able to monitor progress.

I: Right. Yeah.

R: So I think we are falling a little bit behind at the moment but that's as a result of a couple of practices switching off.

GP Pharmacist 3

I: And one final question then; how much work is required to sustain, to keep that level of sustaining the intervention going, to keep it going?

R: That's difficult to quantify at the moment, because we have been working differently, and we're moving to a different system.

I: Yeah.

R: Might be expected to reduce.

I: Right.

R: I think there is still a significant amount of time. I mean I would have to… I mean I think you will be speaking to a couple of the people who actually work on the profile, and they'll be able to give you an idea of some of the time they spend updating the profile. But theoretically the amount of time that they spend updating the profile should have been reduced over the year, and it's been reduced even further, because if you think we spent a certain amount of time updating the profile in (name of place) they spent a significant amount of time updating the profile in (name of place). Now because we worked together we were both able to cut down on the time that we used from both sides; but as this team that I have in place would be updating the first half for the whole of (name of region) that is going to cut the time drastically. I wouldn't say we've cut it in half exactly, initially, but it will cut it drastically. And then also there are other updates on (the CDS) which they've done, which does involve us doing things ourselves.

I: Right.

R: And me, I feel that may well increase the amount of time we do use updating the profile; but then again until we work with it for the next year or so we don't know how significant that will be. So they've given us more functionality, which is fine, but then the onus is on us to do more ourselves.

CCG Pharmacist 5

I: I think, probably last time, I'm going to round up on this, but I think we've...I'm like, throwing some questions out there really, it's just, sort of, it's a follow-up, and I think we probably touched on it in our last interview, I think the value of having something like (the CDS) going forward, what is that value? What's it going to do for patients and...?

R: For me, it's the ability to get a message out to all prescribers, even if they don't engage with the CCG, they are going to see that message. So, I think, yeah, cost saving, great, if they accept those, brilliant. But, for the safety ones, that message will definitely get seen at some point by all the prescribers, if that patient needs it. And I think that's something we can't do, if somebody doesn't want to engage with us, they won't. Whereas, at least we're giving them the chance to [voices overlap 00:35:46].

I: So, they've at least got the information there.

R: Yeah. So, that's from the safety point of view. From the cost saving and QIPP work, it saves us an immense amount of time because the GPs are doing it themselves and it's quick, and they can do it while the patient's there, they can have a quick chat to the patient and it's sorted. Whereas, if we have to go in, do an audit, change it, it's quite time consuming, so it's...

CCG Pharmacist 5

I: I mean, what is that balance, is that balance still, is (the CDS) there for medication safety or is it there to save money?

R: I think it's there because it saves money, but the safety aspect is brilliant. And if it didn't save money, the CCGs wouldn't fund it at the moment. I think that they are that strapped for cash, if something doesn't save money they can't justify it, which is obviously, very, very short-sighted, but that's the situation they're in. I think if you'd asked me ten years ago, I think they would have funded it on safety grounds alone.

I: Just purely as a medication safety?

R: Yeah, absolutely. So, the thing that we need to do is try and put a cost saving on safety measures and that's the bit that we're trying to work on at the moment, how do you, sort of, say, okay yes, we've saved a life or we've saved a hospital admission, how much is that worth to the CCG? And if we can put a value on that, then we're more likely to get the safety aspect of it funded. The CCG obviously care about patients.

I: Yes, [voices overlap 00:07:56].

R: It's just the position that we're in at the moment, it's ridiculous, it's like we've got to save millions and the only way we can do it is by making short term savings, at the moment, and...

CCG Pharmacist 5

R: I think they see it as an annual thing to be reviewed and check it's still worthwhile having. Whereas, I think the users, so the end users and the pharmacists here, say, that's just what we do, that's Optimise. We're getting asked at the area prescribing committee meetings now by members there, oh, could you put an (the CDS) message on for that new decision. So, they see it as a business as usual method of getting messages across to people, so, yeah.

I: So, in that respect, it is about, you say therefore, it's about that, sort of, getting that information very quickly out to a lot of people. So that, when they have it, even if they don't do as you say they do.

R: Yeah, even if they wouldn't…yeah, some people won't ever get messages from our teams because they just don't engage. Yeah, so yeah, it's useful and saving us lots and lots of time.

CCG Pharmacist 7 and CCG Pharmacy Technician 2

I: One of the intriguing things I found about that was that they were sort of rather…I'd said about review there – they picked up on this concept that, you know, (the CDS) would then be acute and Analyse would be more looking at long-term conditions and the long-term management of patients for reviews and stuff. Do you think therefore that (the CDS) is actually better just as an acute sort of alert, or is it something which can be part of…?

R1: No, because when you think about it most of these scripts done are repeat scripts, and so if it's making an intervention on a repeat system then, you know, I don't see that it then should be just like an acute intervention. My understanding from the little bit I've seen of Analyse is that it's a more sort of proactive thing, so you're utilising the similar mechanisms but you're actually going looking for things rather than waiting till they present themselves.

I: Yeah, precisely.

R1: So obviously that's… But then there's other things that do that as well, so…you know what I mean, so you get SMASH, PINCER, [Pinga 39:35], you name it. So it's already in a crowded field whereas the (the CDS) to my knowledge there's always (the CDS) and Script Switch that do that, so it's a less crowded field. But it's up to them if they want to try and develop it but they're going up against some quite robust and already well developed systems – SMASH, PINCER, [Pinga 39:57], you know what I mean, that have got back in…they're already in the system that are well, sort of, tested, that have got Health Innovation (name of place) so regional funding backing. So I don't know.

CCG Pharmacist 7 and CCG Pharmacy Technician 2

I: Do you…because, you know, we're sort of two and a half years down the line, in what ways has… And one of the things that we're interested in, you know, from an evaluation point of view is how this is sustainable going forward. What's…if you like, so what are the key things that have sustained its use, in terms of the CCG or within practice, up to this point? And what are the key things that are going to sustain its use going forward from here?

R1: So I think that the support from practitioners around having kind of a quality message reminder system because, like I said, they've had one version or other in place for nine years now, so… We did give them the option when we changed them over to have nothing and no-one picked that as an option. So I think that there's an expectation they'll have something that reminds them and it's a sort of failsafe. And also, the…ultimately, I suppose the finance within the organisation has got to sort of agree that they're going to fund the licences, so as long as they're happy that it's giving the return it's giving….

So obviously this is where the sort of reporting structure is important because it's…you know, when you can show you've got a high acceptance rate and, you know, you can show that there's a…so you can put a sort of projected potential saving onto that, although I know there might be some elements of double counting because things might be done by different means. But generally speaking, if you've got some simple kind of reports that you can capture once a year to sort of show the benefits of having the system then I think pretty much… They're kind of used to paying it out now. So obviously when you start you've got to make a big thing about, you know, is it worth it, are we going to get the return on this? But I think that as it gets…because it's embedded there's sort of an acceptance that it is worth it. And, you know, the…and we're never questioned, are we, to say, well, you know, why should we buy this again, should we pay for these licences again? So yeah, I think…

R2: I think they're pretty confident, aren't they, that it does what it does and is continuing to do.

R1: Yeah. So finance are happy with that, so, obviously, ultimately they give the okay to make the payments, so that's it.
I: Yeah, so basically because it's making…it's paying its way…
R2: Yeah.
R1: Because ultimately if it wasn't, they wouldn't do it, you know. So I mean, and that comes back to the reports that the system generates. So it's important to be able to kind of…you know, if we are sort of questioned on it, to be able to demonstrate high acceptance rate, you know, estimated savings from number of quality interventions, you know, all the sort of reports, it's quite important to have that functionality.

CCG Pharmacist 7 and CCG Pharmacy Technician 2
R1: I mean, I think it's important that obviously FDB as a company keep looking at how they can…the sort of…how they can manipulate the data that they capture to sort of show the benefits of the system, because obviously if you give…if there's a safety message that comes out, say, it comes out ten times, how would you know…? You might not say ten events because, you know, it might not go wrong ten times, it might go wrong once or it might go twice or whatever, but obviously there's data in, you know, the greater system to show that per…you know, numbers need to harm, for example, or that sort of thing. So you'd put a numbers need to harm on an event. So cross-referencing that with the number of interventions would be an obvious way of saying, well, okay so as long as need to harm is 14 and then, I don't know, say, that intervention, that warning was sprung, like, 15 times, well, there's probably the one event been saved. And, you know, the cost of that event, says, I don't know, something to do with [inaudible 00:37:28] and it's an hospitalisation, is whatever. So, you know, average cost of a bleed…a gastro bleed from…linked to an NSAID is going to be, I don't know, like, £500 or whatever it is. And that way you can start to put a cost on it.
R2: Yeah.
I: You can start to put other sort of costs onto that, yeah.
R1: So I mean most drugs have numbers need to harm trials data and I think doing that sort of thing is… So it's important that they keep looking at, you know, the [repost 37:59] suite and how they can sort of update it and not just do the simple things like, well, that was switched to that and that said that amount, but…

CCG Pharmacist 7 and CCG Pharmacy Technician 2
Do you…in terms of the team here, you know, in the two and a half years you've had it, has it changed the way you work at all as a team? Is it done…is it different, or is it more…is there more work or…?
R2: Usually a lot of the work I do as a tech is…if there's new cost savings that we think would be useful, you'd have to find out whether it's viable and if the pharmacist can get hold of it, if it's long term et cetera, so that takes a bit of digging. I don't tend to do a lot of them now; we've kind of got a set amount of things that we do and regularly switch, that have already been checked out. So that…although it could…although the stock shortages, that's quite a hefty piece of work at the moment. But if there's long-term manufacturing problems we can put it onto (the CDS)but also we can, you know…it sort of impacts on our daily work as well. So getting that message on (the CDS) if I've not already seen it then I'd be able to say, well, this is happening for so many months, and then everyone can go and have a look and see if it's going to affect their, sort of, work in the practices.
R1: I mean, I think that having (the CDS) gives us a sort of additional resource or a capacity or a different level of action. So we're…I mean there's loads of things we get usually, so a costing is often linked to a sort of branded generic but there's often…there's things come through whereby someone will say, well, if you do this you'll save that, or change everyone onto this and you'll save I don't know how much. And we've got a limited amount of staff time and everyone's doing stuff so, you know, you then have to take people off doing whatever they were doing and go onto that, whereas you have got the option at least… But if you think that, well, that's probably…it's not a sort of real headline thing that we've got to pursue but we do want to pick it up if we can, then you've got a sort of fall back to put it on Optimise, and know that you can do that with…all it needs is to go and make sure the right message gets on.
I'm not saying you'll get it every time or, you know, switched over is sort of [inaudible 00:43:10] if you have the [potential 43:12] of doing it. But if you haven't got the capacity to do it, it's either…you know, it's better than nothing, and it's kind of an automated thing. And maybe it's just a sort of low level thing where you think, well, there might be some benefit to it but I'm not exactly sure how much but I don't want to just completely ignore it, so that's a sort of initial resource. So it's like having a sort of background member of staff, if you like, that you can kind of give some automated stuff to. So it is a sort of additional resource that can sort of help you with things like that.

CCG Pharmacist 7 and CCG Pharmacy Technician 2
I: One of the final questions, or possibly the final question, in fact I've been asking quite a lot of people in practice about this, is, in what ways can it improve care, or does it improve care?
R1: So I think in terms of if it's a safety intervention then obviously…and they act on it, then it… Well, I mean, I suppose you're never going to have exactly… Like I said, because someone, like, read it and potentially did something about it, you don't know that then it would've gone wrong if they didn't, but from your numbers need to harm, you can probably make an extrapolation. So I think you can…you know, it definitely has but it's just unless you've done all those analytics it's difficult to say, but if you do the analytics you could probably put a much greater sort of estimation on the level of benefit. But it will be some, for sure, but I don't know how much.

<u>GP Staff</u>

GP Pharmacist 1 and GP10
I: I'm just thinking that connection then to the CCG, and that link through (name) to the CCG, because one of the things that actually I'm becoming interested in is the way in which (the CDS) as a system, it's okay, it's giving you all these alerts, but you've also got to have that other bit that's coming from them, haven't you?
R1: Well, it's like the PINCER. It's like the PINCER theory, isn't it? You've got the information but if you've got somebody who's going to fix it for you or highlight it to you then you're more likely to do something with it, aren't you?
I: Right. Yeah.
R1: So, (name) will pick up… I mean, obviously, she's not going to pick up individual things which PINCER or (the CDS) will pick up, your one-off things…
I: One-off.
R1: …as you're prescribing them. But if there's an area that…'cause she'll look obviously at our ePACT prescribing data, so they monitor that continuously. So, she picks up on an area, then she'll take it up with me and then I'll take it up with everybody and we'll do something about it, so that's how it works. And that works very well for us because yeah, we work well together and that works.

GP Pharmacist 1 and GP10

I: One of the things that I've also found, going round, is that sometimes it's not clear whether an alert is an (the CDS)alert or is coming out of your clinical system.

R2: Yes, true, that is true. Sometimes it's difficult to determine which is which. Yeah.

I: And are you getting lots of other alerts as well as…?

R2: So, there's a few others coming up as well, yes. I'm not sure that it is necessary to know – as an end user – which…

I: Yes, where they come from.

R2: …one it is. No, I don't think it's absolutely necessary to know that. But I must confess, it really does take a lot of effort to work out…

I: Where it's come…

R2: …which one's which. And often the (the CDS), you know only because of your next step which is [own 30:25] override, if it's the override you're going to take. Because it's the only one that offers the drop-down list of different one…yeah. So you know that [voices overlap 30:34]…

I: And it's quite well-embedded into the system.

R2: It's quite well-embedded. Yeah.

I: So, it is look. You don't look like you're moving out of your clinical system. 'Cause it's interesting 'cause one of the things that I think is a consideration is that if other systems…your clinical system and other things are banging out lots of other alerts, it's not just…then that can be the smoke that hides the important (the CDS) alert coming out as well, isn't it? So, it's perhaps the balance within all of that as well. It's just important.

R2: Actually, I haven't seen that. Yeah, I can believe that it could be a potential problem but in reality, in two…a couple of years, I've never seen it. Yeah, it's quite clear. Yeah. And even where you get a couple of alerts, you move through one, the other one will appear shortly after that. It's very rare that that's happened, but where it has it's usually actually from a…and I don't know which one was which but looking at it from a slightly different angle if you like, rather than… Yeah, I'm trying to think of a very good example. It's only very rarely happens. No, I can't think. But yeah, it hasn't…I think it's potentially more of a problem than it is in reality, yeah.

GP Pharmacist 1 and GP10

I: One of the things you said before is if it comes at the right time. Is fitting the alerts into that workflow, into what is a fairly challenging ten minute consultation or can be and so you're juggling lots of things in your head at the same time and there's lots of stuff happening around here. Is that…

R2: So, I'm fairly experienced. I prefer it at that point, if a patient leaves and you have to pick up the pieces after, it's not so good.

R1: It creates more work, isn't it?

I: Yeah.

R2: Yeah, it's not so good. It's not…

R1: [Voices overlap 32:40].

R2: …very professional, to be honest. I prefer to say, oh, I was going to give you this, but actually I've just realised – you don't actually have to say the computers have reminded you – but you can say, I've just realised that actually we should be looking at this and this.

GP Pharmacist 1 and GP10

R2: I think…I suppose final thing is is that when it initially came, it did appear to be…I'm quite Luddite, so yeah, just bear with me on this thing. But equally I do embrace change. It did appear quite clunky and quite intrusive. It's become significantly less, so whatever the reasons, I suppose it's very hard to say, and got to the point where it strikes quite a nice balance in my opinion between giving me enough information regarding safety and patient education and cost-effectiveness. Again, it's just getting on with the work that I've got to do, i.e. I've got a set amount of work in that day, so I don't want to be spending just my time [voices overlap 36:18].

CCG Pharmacist 3

I: Yes, what they are there to do, precisely. One final question, I think 'cause we are heading towards our promised 40 minutes, do you think (the CDS) has or has it got the potential to improve care?

R: Yes. It has.

I: And the supplementary… Why?

R: Of course. It has. It's active, it's there, it works as and when required. It's giving you information and evidence based information from trusted sources on the go, which is great. The information just needs to be succinct. We also need to have a time where we can pull that information off on a monthly basis and reflect on the changes that we've made in order to make it safer.

I: Yeah.

R: Because, yeah, when you've seen 15 to 20 patients a day, you really can't remember what changes you've made.

I: Yes.

R: Yesterday, rather than a week before.

I: Yeah, precisely.

R: (the CDS) has great potential to make things safer. We should be able to use it deeper, so we can reflect on our prescribing so that future prescribing is safer.

GP4

I: Yeah, I suppose because there is the obvious educational element to the PINCER approach whereas, as you say, this is just sort of little nudges, little changes and then maybe somewhere in your head you are, oh yeah, that was a message that came through. The last thing actually, something which I perhaps didn't ask you last time and now I'm am asking other people, is has (the CDS) got the potential to improve care or is it improving care?

R: I suppose not in a global sense really but I suppose there must be individual cases where it has done, you know, where people have been prescribed something that's less likely to cause them harm. And I think, if we're looking at the economic aspects of care then, I suppose it will have, yeah. I don't know, I suppose the answer is yes but I just don't know to what degree and how strongly I would express that. I would feel positive about it.

GP9

I: If they said, right, okay, we're going to put loads more alerts… In some respects, what's going to make it so that it's something which is more embedded into your work, it's less disruptive and so on? What's going to keep it going? If you were feeding back to the CCG, because the

CCG are buying this every year… If the CCG are paying however much they pay, I think it's quite a lot per patient, and they were going to come to this practice saying, we're looking for feedback as to whether we should buy this again, would you say, oh, yes, keep going with it or do you think, actually, no, it's too disruptive, I prefer the old ways of doing things?
R: Oh, I'd probably say keep going with what we have now but equally if it came down to cost and you think, well if you made your formulary limited then you wouldn't need the (the CDS) as much because you've taken everything off, like you're not allowed to prescribe more expensive then take it off the formulary and then you wouldn't be able to choose it in the first place which would then leave (the CDS)redundant.

GP9
I: Yes, but that could be part of that actually. I'm just interested in what's going to make it sustained? What's going to keep it going? It seems that you're saying reduce some of this, that… Would it be better if it was…?
R: I suppose, A, I don't really understand what you're trying to get at in terms of what's going to keep it going because it's there so I don't… If they're going to pay for it, it's going to keep going, isn't it?
I: Yes, right.
R: If they don't, you know, it'll be something else that we look into, a different way. So it doesn't make a difference to me in terms of what I do. If they don't go with it, it's not my decision. If it suddenly gets pulled then we'll just go back to…we'll just find a different way of working and doing what we used to do before [inaudible 17:15] that. It's not my decision. I have no influence over it but I suppose it is easier to see it than to remember things because it just flashes up.

GP14
I: Yeah. Do you think it's something which should be used routinely across primary care?
R: Yeah.
I: Why?
R: Why? Because it doesn't have any negatives. From my perspective apart from annoyance when it pops up.
I: Yes.
R: Because you have that autonomy to override it if you want to, you don't have to follow what they say. There's no reason why a little pop up…as long as you can cancel it off quickly and simply and it doesn't take ages to get around it which I don't think it does, then there is no reason why little suggestions coming up from time to time can't enhance patient safety and cost effective prescribing.

GP14
I: One of the things, I think, actually we haven't really touched on is the next [inaudible 26:56] actually, is…I mean, you talked about maybe disagreeing with some of the alerts, but is…are the, by in large, do see those alerts as timely, relevant and useful or are they…?
R: Almost always. Yeah, so, if it's a patient safety thing, one assumes whoever writes the software made them pop up for good reasons. So, you have to take it seriously and have a look at it and see whether it's something that actually you need changing. I see it as very positive.
I: So, in that respect you have trust and confidence in it?
R: Yeah. Whether I should or not is probably another question because it could be any Tom, Dick or Harry, I suppose…
I: Well, I think they are…
R: …One assumes they get onto NHS systems then they must have gone through fairly rigorous testing, et cetera.

Sub theme 7iii: PINCER and (the CDS)

Software developer staff
Software developer staff 1
R: Yeah, so if we're looking at the MMT, a good way especially for reviewing the profile is I'd get them to focus…because at the moment, if they've got best practice and [look at 0:55:31] best practice, it's all just in BNF category, there is no organisation. If they use the search terms, then it will list…the search PINCER it will show them just the PINCER messages, so they can quickly go through, or just the MHRA or just the GMC, or just NHS England or just NICE or whatever. So they can look in each category. Stop-start was always…not necessarily start, but stop was also another one that people were very interested in to have a look at and make sure that they're activated. There is a thing that a lot of CCGs will go, yeah, whether it's PINCER, stop, MH…not all MHRA but most MHRA, are just automatically they'll be enabled, because they're no-brainer for safety

Software developer staff 1
R: Yeah. I mean, now with localisation of national messages, there's no reason why, if an organisation wanted to raise awareness that it was a PINCER they can add in the text that this is a PINCER indication, if GPs were aware what PINCER is. I'm sure…across in the East Midlands where I was before yeah, everyone knows what PINCER is. Go a bit further south or north, some do, some don't. I mean, that's still really quite a high-hitting message actually in a lot of…we could do that…looking at the work that (name), been doing around benchmarking, he can pull that particular message out for every CCG and see how they compare. That would be quite interesting actually to look at…you know, but PPIs and NSAIDs, again, will be a forever piece of work for CCGs, it will never end.

CCG Staff
CCG Pharmacist 2
I: We talked at the start, before we switched the tape on actually, about the distinctions between Pincer and Optimise. What do you think are the benefits of the two approaches are, because obviously that at the point of prescribing, and mopping up after the event, because in the CCG both approaches are being used, what do you think to that?

R: (the CDS) is obviously a bit more proactive, whereas the other ones are being reactive to what you found. And you're like, oh I didn't realise we had these patients who've slipped through the net. Because, I think you probably do need a bit of both. Because you're not always going to get 100 per cent somebody compliant to those messages, it might be that, oh actually, either it gets ignored, or you get interrupted and miss that message. And it can happen where they've then ended up prescribing the wrong dose, or something, or giving them something without a PPI, 'cause they're not thinking or whatever. So, it's good to have that mop up, just in case people have slipped through the net. And I think, also, this has just come to me, actually now, is that if you get patients who are discharged from hospital, on drugs and things, and so they just get blindly

added, a lot of the time it's an admin person maybe, who'll just add, these are the drugs that they're on, these are the ones that were stopped, just update the patient's records. They might come up with some messages, but they may not action those messages. So, again, somebody might slip through the net, because it's been prescribed from somewhere else, and there's been changes made, so there is that element of it as well, for mopping up. And I don't know, I'm just thinking from a clinician's perspective, I wonder whether there's more, doing it in the Pincer way, there's more of an underlying understanding that occurs, because you're actually seeing what's going on in your practice, and going, wow, we have ten patients on this, have we got a bit of an education issue, that we need to deal with in our clinicians? Or, what are the reasons, is there a pattern, is it because it's been suggested by specialist consultants or something, and we need to feed that back to the hospital, that something's not right? Or just generally, just going, oh, this patient has been going on for a while, have they had an episode of bleeding? Then you're doing a bit more of a thorough review in somebody, in those cases.

CCG Pharmacist 2

So, it's good to have that mop up, just in case people have slipped through the net. And I think, also, this has just come to me, actually now, is that if you get patients who are discharged from hospital, on drugs and things, and so they just get blindly added, a lot of the time it's an admin person maybe, who'll just add, these are the drugs that they're on, these are the ones that were stopped, just update the patient's records. They might come up with some messages, but they may not action those messages. So, again, somebody might slip through the net, because it's been prescribed from somewhere else, and there's been changes made, so there is that element of it as well, for mopping up.

CCG Pharmacist 2

I: We talked a bit about the PINCER approach in comparison to Optimise. I mean last time we were talking about how you felt it was (the CDS) maybe just avoiding the…because it's just like that change at the point of prescribing, you're not actually perhaps getting that learning that you would through PINCER. I mean is that how you see it still or is…because you're still using PINCER there, presumably, PINCER is still happening?

R: Yes, absolutely, at the minute. I don't know. Again, because I don't work in practices myself, it's hard to comment on that not knowing what the GPs in the practices think of them both. I think, yes, with the PINCER work, you've got that medicines management team, sort of, support with that and the feedback, you know… I think sometimes the figures can sometimes speak for themselves a bit more loudly. So obviously the whole thing about PINCER is trawling through and saying, how many patients have we got who are on, for example, an NSAID without gastro protection who are aged more than 65. It can be quite shocking sometimes if you then do a trawl and find that you've got like five patients or something and like, how have they slipped through the net and have they had any harm in the meantime? Yes, so there is that learning and I guess thinking about it from that perspective, (the CDS) and PINCER then go hand-in-hand in some ways because it's almost like they both doing the same sort of job, aren't they?

CCG Pharmacist 2

The thing with (the CDS) as well, I can't remember if I said this last time round, is that…and the whole thing about PINCER is it's a snapshot in time. Unless you do that search again for new patients who might have come through, those patients could slip through the net, whereas (the CDS) is there at the point of prescribing for every single prescription for that patient who… Do you know what I mean, say it could be somebody new comes along and they're like, oh, they're on an NSAID and doctor x had seen them before and hadn't considered giving them a PPI. Have they missed that alert or do they need a bit of training and educating on that front? I've picked it up now so I'm going to try and do something about it now […] So it will be a trigger all the time and as we know as well, the thing about GP systems and I know learning from incidents that have happened, is that sometimes some things just fall off the prescription new list. Sometimes it could be like something was issued as an acute and then it's never got onto the repeat or a patient stops ordering x. You know, they might be like, oh, yes, I need that. I need naproxen for my pain but I really don't like taking omeprazole because it makes me feel ill. Ever since I've started taking that I feel really hmm. No, I don't really need that but my pain's better so I'll just carry on with that, so they may not order that and it might fall off the repeat list or the [inaudible 34:03] says they don't need that, they're not ordering it. Hopefully not because they'll see the benefits of them being on a PPI and hopefully have that conversation with them but you can see how things get taken off.

CCG Pharmacist 3

R: And I thought, mm. Is it overkill? But then I think can you be too safe for a patient? And if the contraindication or interaction has already been dealt with by PINCER, it's not going to come up as a message on Optimise. So that's probably a very simplistic way of looking at it.

I: Yeah, and if it comes up on a message [inaudible 41:39] is PINCER might then deal with it at a later stage down the line, and then again, as you say, unless you stop the…

R: Yeah, and if they've accepted that message with Optimise, so for instance if it comes up with Optimise, this patient should be having a PPI, they've dealt with it. When you next run the PINCER searches, it's not going to come up because they're not [having 42:00].

I: No, precisely. Yeah.

R: So I think they complement each other. I'm sure there's a way that they could be rationalised and I mean, probably go forward, as I say, I don't know all the technical ins and outs, and I thought PINCER indicators where it didn't work embedded in Optimise.

CCG Pharmacist 3

R: Because going forward should we need PINCER separately? Because if it's embedded in (the CDS) and the whole thing with PINCER was about learned behaviour, so if it's in Optimise, do we need PINCER separately? I don't know. I haven't got enough…and I don't know whether we're at that stage yet.

CCG Pharmacist 4

R: It's going to sound like quite an altruistic answer really. My argument is that I think the best prescribing is always the most cost effective prescribing. And I think whichever way we get to that goal doesn't really matter. It's only tools that you've got. So I think it's always appropriate to prescribe drugs in this class of patients, and I think it just helps to filter the views for GPs on that where… I think we talked about Pincer and I think Pincer's probably quite a good example, and I think where (the CDS) is good is it's at the point of prescribing for GPs. So if you're going to invoke

any behavioural change, I think that's where we always struggle, by mopping work up you don't really make them think, whereas (the CDS)at least does make them think actually.

GP Staff

GP Nurse 2

I: I suppose the one other thing is that, you know, thinking from a PINCER point of view of having PINCER and going through the PINCER indicators, trawling through, finding the patients that are affected by those prescribing safety indicators. I mean in some respects you can do that when you do a medicines use review, do you think that is a better way of doing it or is it this? How does that compare and contrast with having something, you know, about to go and prescribe, boom, comes up, oh, don't do this?

R: It's better to have it before you do it, surely, really isn't it?

I: Why do you think that?

R: Well, because then you're not constantly trawling through audits and changing things and getting patients in and having to explain why you've done that, why that shouldn't be on there or them getting a letter from meds management. So, to me, it's about prevention's better than curing, just having an alert there and then, yes.

GP Pharmacist 1 and GP10

R: Yes, I mean I think because there's pros and cons to both approaches, isn't there? Both approaches are, I suppose, good in a way and the thing with the (the CDS)ones, I suppose because a lot of them are more maybe the formulary or cost things, we might not go through those in such a systematic way. So as a practice we tend to not send out batches of letters switching A to B.

R2: No.

R: We prefer to do it on a patient by patient basis so, actually, then the (the CDS) works because when the patient is sat in front of you, it pops up and tells you so then you can say to the patient, okay, actually, you're on this MR [16:14] preparation and we want you to be on the standard preparation, is that okay? That's fine, whereas when I'm working through PINCER, if there are patients that need to be actioned then we will need to call them in, arrange for a call, whatever it is.

R2: Call them in, yes.

GP Pharmacist 1 and GP10

I: I'm just thinking that connection then to the CCG, and that link through (name) to the CCG, because one of the things that actually I'm becoming interested in is the way in which (the CDS) as a system, it's okay, it's giving you all these alerts, but you've also got to have that other bit that's coming from them, haven't you?

R1: Well, it's like the PINCER. It's like the PINCER theory, isn't it? You've got the information but if you've got somebody who's going to fix it for you or highlight it to you then you're more likely to do something with it, aren't you?

I: Right. Yeah.

R1: So, (name) will pick up… I mean, obviously, she's not going to pick up individual things which PINCER or (the CDS) will pick up, your one-off things…

I: One-off.

R1: …as you're prescribing them. But if there's an area that…'cause she'll look obviously at our ePACT prescribing data, so they monitor that continuously. So, she picks up on an area, then she'll take it up with me and then I'll take it up with everybody and we'll do something about it, so that's how it works. And that works very well for us because yeah, we work well together and that works.

GP Pharmacist 1 and GP10

I: Yeah. Absolutely, yeah. And thinking of that…well, I mean, thinking within like you mentioned PINCER there, in what way is it good to have PINCER, in what way is it good to have Optimise? In what way is it good to have the alert as you're about to prescribe that drug? I know what ways is it good to have the alert…

R2: …after.

I: …afterwards, or being told afterwards you've done something wrong, not wrong ?

R1: Yeah. Well, it's good to have it at the time of prescribing because then you can do something about it in real time, and hopefully with the patient in front of you, so therefore you just deal with it…

R2: [Voices overlap 18:31].

R1: …there and then, and it's done. I suppose the PINCER stuff is useful because I suppose it's more of an audity kind of approach, isn't it, so we're looking back over the last however, six months, and then we can follow that up. I suppose with the PINCER, then you feel you're dealing with it more comprehensively. So, you know for that issue, this is the list of patients that that applies to, you can comprehensively go through and review them all and make a decision and go, right, we've dealt with that, we're now up to date on that. Whereas with Optimise, it's opportunistic, isn't it? So…

I: Yes, it's just if you're having to [break 19:05] that prescription for that particular patient at that particular time, it is, yeah.

R1: Yeah. So, both are useful in different ways.

GP Pharmacist 1 and GP10

I: Do you think we need both [PINCER and (the CDS)]?

R2: I think in a…

R1: Yeah.

R2: …system that involves humans, that anything that aids…

R1: As many things as…

R2: …aids decision-making up-front is always useful. Anything that highlights a mistake that's been made is invaluable. So…

I: So yeah.
R2: So, the answer is yes. And I think the up-front decision-making and help at the time is extremely useful but the picking up on mistakes – which is inevitable to happen, and they're not necessarily mistakes, but I use the word – is essential, absolutely essential. So yes, both are necessary, yeah.

GP Pharmacist 1 and GP10
R: So, we get PINCER at the moment biannually and the searches are done for us centrally by the CCG and it comes down as a waiting list on (GP clinical system). So, I'll get an email from (name) going, PINCER's coming in the next couple of weeks and I'll look at the waiting list and I've got a list of patients, then, that I can go through and these ones you need to look at their PPIs or the [voices overlapping 14:21] or whatever it is. So I tend to do that as an organised piece of work and I will block out some time to sit down and go through those patients systematically and review them and deal with them and action them and log all of that. Whereas obviously the (the CDS) is much more opportunistic. So, I guess, potentially I have a much…

R2: Yes, PINCER's systematic and…

R: Yes, so I've got a much better, kind of, idea…

R2: …(the CDS)is, sort of, just...

R: …of how we perform on PINCER compared to, say, how we perform on Optimise. We're looking at how many alerts we get on PINCER versus how many we get on (the CDS)and what we actually do to them. Also, I have a log so I actually have a spreadsheet of PINCER that I update each time because often the same patients will come up again. Again, on PINCER, sometimes we won't be changing them, they'll stay on that alert for whatever reason but then I'll go back and I'll know six months ago, yes, I know that patient, everything's fine, we can carry on. Whereas (the CDS)is much more opportunistic and therefore, a bit random so I couldn't tell you how many patients or, you know…

GP Pharmacist 2
I: Yes, it does, yes. So, you're looking at new indicators, but in terms of the PINCER approach, which, you know, you're well familiar with, do you think the PINCER approach...how do you think that works with Optimise? The two approaches, well actually, one is the point of prescribing, one's dealing with things that have happened, and hoping to change things. Of those two approaches, what do you think of the two, how they compare and contrast?

R: I mean, you would hope, well PINCER is a way of learning, isn't it. The idea is to see where you're at, and then, it's an ongoing thing because you get patients being put on NSAIDs all the time, but it's about teaching, isn't it, and learning. So, sharing that learning, like this is where we're at, this is what's been going on, this is what we need to do differently, let's see if we can improve. And you're never going to be 100 per cent perfect, because it's not possible really. And so, I suppose, if you learn from PINCER then that should have an impact on Optimise. Because you shouldn't be having those safety messages coming up, because that patient should have been identified as having been on Aspirin and Warfarin and no GI protection, or whatever. So, it shouldn't kick in when you use (the CDS) or when (the CDS) kicks in. So, yes, but we're having lots of different ways of trying to achieve the same goal, aren't we.

CCG Pharmacist 3
R: Thank you for adding that choice in there as well. Is there one? PINCER is nice 'cause you can go back and reflect on your prescribing?
I: Yeah.
R: And make those changes into the future. But sometimes you need to make the changes straight away – where (the CDS) is very good at doing, 'cause it gives you good information, justified information to do so.
I: Yeah.
R: Both weigh equally well to me.
I: Yeah.
R: They really do. They both have their advantages, no disadvantages really.

GP3
R: Optimise, compared to PINCER. PINCER 's interesting, because what you're doing is PINCER , you're saying this is wrong, and saying it's wrong makes you…oh, I've been doing something wrong for the last three years, and you're not comfortable with that. So if someone says this is a better way to do it, this would be what we think and the reasons why, it's much better. So I think GPs completely would just dig their heels in if someone says you've not to do something, and they'll say get more and more evidence, more and more evidence, and they'll argue black is white sometimes.

I: Yeah. In terms of the indicators, the PINCER indicators are in Optimise, so…

R: Yeah, but it's different. PINCER , for example, PINCER people that had GI bleeds that were on…and that was really very blunt. It was useful in the fact that all you do is go, well, I wouldn't do that, that's stupid, so there must be another reason, there must be a coding issue. This is what GPs do if we say there's a problem, there's a coding issue isn't there?

I: I have heard this before.

R: It's I'm a prefect prescriber, but there's a coding issue.

I: Definitely, must be coding. It's either that or it was a locum.

R: Yeah, that's another thing. I think in (the CDS) there's a slightly different way in the fact that it gives you a warning and it tells you why, and it gives you chance to correct without having been judgmental.

GP3

R: Whereas PINCER would flag up our six patients that didn't have PPI cover and things like that, and then what we'd do because we're anal about it we'd isolate the people that prescribed that and say why did you do it? So it was quite judgmental about it. Whereas if you do it as you're going along it's just like I'm changing my practice. Oh, I didn't realise. Of course that's the right thing to do. It's not a judgement […] And if you've done it wrong before the way to change is to feel as if it's comfortable to change, not that you're just a bad prescriber.

GP3

R: It pops up, and this is where I have an argument. What's the difference between 64 and 66? This is the problem about PINCER. It's much better if someone suggests would it be a good idea to have some PPI cover with this? That's a much better way, rather than say these are the people that have found…or over 65 that are nonsteroidal […] It's not the same. You say it's the same, but it's not. One offers you a bit more of a solution, the other one you have to go back and say what you did wrong.

GP4

R: I think they are complementary really. I mean, I wasn't the doctor who did the PINCER, we had one of the other doctors in the practice who took that on and (name) forwarded all the spreadsheets and he just worked through them. And he's on his second career as well, he was a chemical engineer who worked in the pharmaceutical industry and he's used to evaluating things and so he actually really enjoys it. So…and obviously now 'cause he's got a clinical skill as well, he's able to completely resolve things. So he'll go through the spreadsheets, he'll speak to the patients who need to be spoken to and it's a really good way of tidying up that group of outliers I suppose, so people who've got a significant…whether it's the number of inhalers they've been using or people who've got asthma and been prescribed betablockers…

I: Betablockers, yeah.

R: …and things like that, it's a really good way of mopping all that up and feels…if we waited just to do all that opportunistically there would be two risks, one that we wouldn't see those patients necessarily or that when we saw those patients we didn't think about it, or that when we saw those patients and the (the CDS)popped up, we wouldn't have time to deal with it.

GP4

R: So that sort of feels…this is like it's an ongoing opportunistic thing with (the CDS) whereas it feels something summative about doing the PINCER, you just get this group of patients and you catch…it's either they're identified and they're sorted in a splurge [inaudible 33:46].

I: Yeah, precisely.

R: It's a good way of dealing with that, those safety issues.

GP4

R: I think they probably both have a role but I think at an organisational level I think probably having more of the summative approach really, of having some data you can look at, like the PINCER type approach. I have always found that useful, particularly now we've got a pharmacist who can help summarise it and present it back to us in a – say, for example, the stuff about antibiotic prescribing percentages, see the drugs that we've prescribed, I think we've looked at [meodarin 22:30] prescribing and we looked at patients who were having too many inhalers and patients who were on beta blockers with aspirin, things like that. And, non-steroidal, that made a big difference to our non-steroidal prescribing was the stuff that came up through PINCER [voices overlap 22:52] as a result of that really at practice level. So, this feels like a constant formative process of just having little reminders and little nudges really almost, I think probably is quite effective but it's been difficult other in anecdotal terms to describe that really, so perhaps for that reason alone it feels like the PINCER approach has been more effective really.

GP7 and GP8

R3: So for me (the CDS) is for cost-effective prescribing in line with local guidance.

R2: That's the most helpful thing I think as well.

R3: Whereas PINCER is the safety stuff.

I: Right.

R3: So that PINCER is give a PPI or think about this. So they're two separate things.

I: This is interesting. It's really interesting because I suppose one of the things is, I've done interviewing in places where people haven't got PINCER and for them of course it's firing on all the PINCER alerts and everything else. But for you you're finding it isn't.

R3: No. You could ask [inaudible - 0:22:10] that, whether it's just been disabled because we have PINCER

[…]

R2: I think because she's got the list down to such tiny numbers that's probably part of it as well.

I: Yeah, well that's, that will be…

R2: Because every like, what, six months does she run all of our PINCER indicators and send us tasks if not more often, I don't know. So we get individual tasks about our own patients and then we

GP8

I: Actually thinking about that, since we're talked about PINCER, do you think it's better to have the sort of pop up message and make the change there and then at the point of prescribing, or is it better to have the PINCER approach where you've, if anything slips through the net the pharmacist looks at it, comes to you and says, oh you need to think about this broad range you've prescribed, or you need to think about this or these things?

R2: I think you probably need a bit of both. Because I think the (the CDS) is really good because it stops you doing it at the time or it makes you think about it at the time and really question what you're doing. But I don't think for example if somebody over-rode that and put something on a repeat prescription for 12 months that that would necessarily get picked up when the receptionists issued a medication. Because I don't think they get the Optimise, they won't get the (the CDS) box popping up when they just…

I: Well it shouldn't come up on repeats, it shouldn't.

R2: So if something has slipped through the net or if somebody prescribed something and it hadn't been on (the CDS)at that time or something like that, it's not going to be picked up potentially until the medication review and then it depends on how careful people are when they are doing medication reviews, which is variable isn't it across everybody how they're done and what you think of at that time.

GP 8 and GP9

I: Have you seen, I mean we were talking about earlier and introducing things, about the prescribing safety indicators that are measured in the PINCER, so they would be thing around the exacerbation of asthma, and things like, the beta blocker and aspirin is one of them. Things around avoidance of gastric bleeds with things like NSAIDs in the over-65s that's one of them. Or NSAIDs with a history of an ulcer; so have you seen any of those coming up through the (the CDS) system at all?

R2: I don't think so.

I: Or do you see any of that around those sorts of things?

R1: I can't remember but those patients tend to be already on a PPI…

R2: We've had PINCER alerts sent to us by our practice pharmacist because she is around PINCER things and said, can you look at this one, can you look at that one? But I don't remember seeing them on Optimise.

I: Perhaps you're doing them so well in PINCER that they aren't coming up?

R2: Well to be fair she does, she does keep on top of all the PINCER stuff doesn't she quite regularly? And she sends us all individual task things, can you look at this one? Can you look at this one?

GP9

I: What do you think of the two interventions, the two approaches to improving prescribing?

R: Well, PINCER that's just non-steroidals and not on PPI so I wouldn't use (the CDS) for that because I know we run searches on a regular basis for PINCER […] if they come up on (the CDS) then, yes, I would probably look…[voices overlapping 13:13] PPI or something like that but if they don't come up then I'd leave it to the pharmacist.

GP 11

I: So, did you find value in that PINCER work?

R: Yes, I did. It sounds bad but I think it's true, anything that forces you to review the patient safety stuff is good because actually, I think with the workload of time, and so on, I think it can be easy to not or to do a semi-job on it. So, I think something that highlights it in black and white. This is why it's a problem.

GP 11

R: Yes. So, whether it's a difference between long-term patient safety versus acute, I think that's pretty much exactly how I think of the divide in it.

I: In what sort of way?

R: So, I think lots of the things that PINCER picked up is the sort of stuff we miss because it's long-term safety. So, things like the naproxen has continued on for a stupid number of years. Let's try and sort that out, for example. And I think it's quite easy when dealing with the acute, which is keeping patients safe but in a very different way, to ignore that and defer the meds review for another time which doesn't happen because the workload of the acute is high.

I: Yes. So, you're constantly parking the meds review because you're dealing with what needs to be dealt with now and so [actually 18.15] the safe issue is this bit and that safety bit in a way. So, PINCER has that effective [period 18.21] with looking at that stuff then, doesn't it?

R: Yes, it does. And I think particularly without acute because that's what most of our patients naturally do. So, in lots of cases we are trying to chase down the long-term. And, still, patients, here in particular, will often present acutely chaotically and you do other things in a mess.

GP 11

R: Yes. So, I think complexity and the relative acute versus long-term demand and arguably, NHS wide, the supply demand mismatch. But yes, I think particularly for most of the doctors dealing with the acute it's pretty much a full-time job.

I: Yes. So, you're dealing with the here and now problem and not something that is to be dealt with. If you take the PINCER approach and the Optimise, so that you've got your safety messages at the point of prescribing, safety messages through an audit and a pharmacist, coming to doctors saying you shouldn't have done that or whatever, no not saying you shouldn't have done that, discussing that prescribing in the practice where they are based, which of those approaches, do you think, serves people better, serves clinicians better, serves patients better?

R: Yes, interesting. I wonder if (the CDS)gets used more routinely in a wider range of scenarios. And thinking of acute long-term, we're probably more specifically through that. From my memory, most of the people who are on PINCER are more long-term and [if I was 21.29], you know, pretty much PINCER would pick up everybody.

I: [As you said 21.35], it's been prescribed for two years.

R: Yes, absolutely.

I: And they've never ever had gastric protection with it.

R: Yes, definitely. So, I think that would be an advantage of (the CDS)as the everything. I think, as I've mentioned, there's the variation in how used and overridden (the CDS) is, I think. And I would guess, but I'm not sure, that there's more variation with that than with PINCER or something. Yes, I think PINCER probably pins you down to do something.

GP 11
I: Yes. Well, that's really interesting. Also within that then is, where's the learning effect with either of those approaches?

R: Yes, that's interesting. Yes, I think there certainly has been. And I can think of that with audits in general, you know, learning that still continues to change practice afterwards. Yes, I know I said prescribing is different after PINCER than it was before. So, in terms of these not these open ended 84 tablets per 28 days for the rest of your life things or at the very least do a script note of, please review after X number of months or something. So, I think that has been something that's changed with that. So, I think that's something with audits in general. For something with Optimise, it's probably sort of the change of guidance that keeps happening. I think that's really useful. So, something like Nefopam. That's something that I've changed because of that reason. So, the fact that it flashes up as Amber 2 every time I try and do it. And I still sometimes do... I think it's worth overriding that CCG rule because there isn't anything better but actually, it's useful that I understand that this is relatively cost-effective badness that I'm chucking out.

GP13
But, in comparison to the PINCER approach, that learning is different isn't it, it's a different type of learning?

R1: Yes, it is, 'cause it's a sort of…the PINCER approach is learning too late isn't it? 'Cause you've already done it wrong. It's like a Minority Report type of learning, you know, intervening just before the crime occurs, which has got to be a good thing really, as long as it's programme correctly, and like all tools, it can be used for good and used for evil, so as long as I trust the people who are programming it, and they're using it for good, and they're not being sponsored by the sertraline manufacturers, then it's a tool for that. So yes, a lot of learning and prescribing senses, but again it's the type one, type two argument again, it promotes the learning, but it might encourage complacency about the stuff it doesn't [voices overlap 00:39:44]...

GP14
I: Yeah. The other thing I was thinking just there as well is, we talked a bit about PINCER. You've done PINCER here presumably?
R: Two or three times, I think.
I: You know of PINCER pretty…well, you ought to with being in this city.
R: Yeah, exactly.
I: So, PINCER is after the event, audit, and lets us learn from our mistakes. (the CDS) is, it's preventing mistakes from happening in the first place. Do you think, which is the better approach, or is neither of them the better approach?
R: I think they're both good approaches but well, good is not as…both are important approaches, generally speaking in principle, prevention is better than cure, but that doesn't mean there isn't a place for cure. So, if you can find mistakes because they will always happen in medicine. You can't get no mistakes on the NHS because everything is so complex and there's so many, there's so much, can be millions of prescriptions, there will always be but you do everything you can to minimise that so, pro active and retrospective approaches are both entirely valid. Probably need using together to be fair, that's how you're going to get the most out of your learning and minimise harming impacts on patients.
