# Supplementary material for: The implementation, use and sustainability of a clinical decision support system for medication optimisation in primary care: A qualitative evaluation
Source: PLoS One. 2021 May 3;16(5):e0250946. doi: 10.1371/journal.pone.0250946 (PMC8092789; doi:10.1371/journal.pone.0250946)
Supplement: S1 Checklist — (DOCX) [file pone.0250946.s004.docx]

**The Implementation, Use and Sustainability of a Clinical Decision Support System for Medication Optimisation in Primary Care: A Qualitative Evaluation Using Normalisation Process Theory**

**COREQ checklist**

Note: in order to minimize the length of the manuscript, some of the details on the checklist (marked ‘*’) are not included in the manuscript.

| *Guide question* | | *Response* | *Page number in manuscript* |
| --- | --- | --- | --- |
| 1 | Interviewer/facilitator | MJ conducted the interviews. | P7 |
| 2 | Credentials | MJ holds an PhD in Pharmacy Practice | * |
| 3 | Occupation | MJ : Research Associate in medication safety; | * |
| 4 | Gender | MJ male | * |
| 5 | Experience and training | MJ has previous experience of undertaking qualitative research in healthcare related to medication safety and the use of technology at PhD and postdoctoral level | P7 |
| 6 | Relationship established | The researchers were not known to the participants prior to the study | * |
| 7 | Participant knowledge of the researcher | Participants were made aware of the reasons for doing the research via the information which was given to the participant prior to the interview | Sampling and recruitment P6; Ethics statement P9 |
| 8 | Researcher characteristics | The researchers had identified the study topic as part of larger programmes of work in their research groups, medication safety in primary care. | * |
| 9 | Methodological orientation and theory | Normalisation Process Theory. The analysis was thematic. | P7-8 |
| 10 | Sampling | The sampling frame was stakeholders involved in the implementation of the CDS system, and/or in the ongoing use of the system, working within the CCG areas. Practices and CCG areas were only included if they had installed the CDS within the previous twelve months. In addition, staff working for the software developer were also approached to take part in interviews. Stakeholders therefore included CCG managers, CCG pharmacists and pharmacy technicians, GPs, general practice nurses, general practice-based pharmacists and pharmacy technicians and software developer staff. CCG and software developer staff were approached directly by MJ or RNK via email or telephone and invited to take part in interviews. General practices where staff could be invited to take part were identified through discussions with CCG managers. A typology framework of practices in each CCG was developed based upon demographic factors to facilitate recruitment of diverse groups – including size of practice, use of different electronic health record (EHR) systems, indices of multiple deprivation for the area the practice served, CCG reported engagement with the CDS, and time since implementation of the CDS. Potential practices were approached by email or telephone by MJ and invited to take part. In addition, MJ visited CCG meetings of groups of practices. Once practices indicated that staff might be interested in taking part, MJ visited the practice to explain the study further and provided written information. Practices consented to take part in the study before individual staff were approached. Individual general practice staff were approached directly by telephone or email, or through liaising with the general practice manager. A total of 41 practices were approached, 32 either declined to take part or did not respond to approaches and nine practices consented to take part. Reasons for not taking part were usually associated with time and staffing commitments. All individual potential participants were provided with study information and given at least 24 hours to decide if they wished to take part. They were then contacted by MJ to arrange a convenient time for interview | P6 |
| 11 | Method of approach | Participants were approached by telephone or email | P6 |
| 12 | Sample size | 33 participants | Results section P8 |
| 13 | Non-participation | A number of possible participants were approached but declined to participate. This was predominantly this was for reasons of time, workload or lack of use of the system. | Discussion P27 |
| 14 | Setting of data collection | All interviews were conducted by MJ and took place at the participants’ usual place of work (general practice, CCG offices) or at university premises (two interviews). | P6-7 |
| 15 | Presence of non-participants | No non-participants were present | * |
| 16 | Description of sample | Thirty-nine interviews were conducted with 33 participants. These comprised of interviews with general practice staff (n=20:- GPs = 14, Nurse prescribers = 3, practice pharmacists = 3), CCG staff (n=10: CCG Pharmacists =7, Pharmacy Technicians = 2, data analyst =1) and software developer staff (n=3). Of the 39 interviews 11 were follow-ups. Thirty-five interviews were conducted one-to-one and four as group interviews. | Results P9 |
| 17 | Interview guide | The semi-structured interview schedule was informed by Normalisation Process Theory (NPT) and designed to illicit how people understood the value of the CDS, how it had been implemented, the work in using the system and how the intervention might be sustained. | P6-7 Si Appendix |
| 18 | Repeat interviews | 11 follow-up interviews were conducted | P8 |
| 19 | Audio/visual recording | Audio recording only, with consent from the participant | P8 |
| 20 | Field notes | None | * |
| 21 | Duration | Interviews ranged in duration from 13-68 minutes with a mean length of 36 minutes. | P8 |
| 22 | Data saturation | Data collection continued until saturation was reached and no new themes emerged from the interviews.. | * |
| 23 | Transcripts returned | No transcripts were returned to participants | * |
| 24 | Number of data coders | MJ coded the data but regular discussions codes were held with co-authors | P7 |
| 25 | Description of the coding tree | A coding tree description is not given. Coding themes were developed from the first transcripts and then developed through discussions amongst the authors | P7 |
| 26 | Derivation of themes | Emerging thematic codes were applied to the data and new themes emerged from the data. This is described in the analysis section | P7 |
| 27 | Software | QSRNvivo 12 software was utilised to manage the data | P7 |
| 28 | Participant checking | No | * |
| 29 | Quotations presented | Please see the results section of the manuscript | P9-P25 |
| 30 | Data and findings consistent |  | P9-P25 |
| 31 | Clarity of major themes |  | P9-P25 |
| 32 | Clarity of minor themes |  | P9-P25 |
