# Supplementary material for: The implementation, use and sustainability of a clinical decision support system for medication optimisation in primary care: A qualitative evaluation
Source: PLoS One. 2021 May 3;16(5):e0250946. doi: 10.1371/journal.pone.0250946 (PMC8092789; doi:10.1371/journal.pone.0250946)
Supplement: Attachment — Submitted filename: Response to Reviewers.docx [file pone.0250946.s006.docx]

PONE-D-20-35487

The Implementation, Use and Sustainability of a Clinical Decision Support System for Medication Optimisation in Primary Care: A Qualitative Evaluation Using Normalisation Process Theory

PLOS ONE

Dear Dr Borsci,

Re: ‘The Implementation, Use and Sustainability of a Clinical Decision Support System for Medication Optimisation in Primary Care: A Qualitative Evaluation Using Normalisation Process Theory’

Thank you for the further feedback on our manuscript, which we have carefully considered. We have in the light of this feedback made further revisions to the manuscript, which are detailed in the table below.

We are confident that these revisions will be to your satisfaction, but please do contact me if you require any further clarification or revisions.

Yours sincerely,

Mark Jeffries, on behalf of the co-authors

| **Reviewer comments** | **Response/Changes made** | **Location of change in tracked changed manuscript** |
| --- | --- | --- |
| TITLE: |  |  |
| The title of the manuscript is too long and it needs to be shortened such as removing ‘Using Normalisation Process Theory’. | We have taken appreciate that the title is quite long. We are therefore happy to change the title to: “The Implementation, Use and Sustainability of a Clinical Decision Support System for Medication Optimisation in Primary Care: A Qualitative Evaluation” | Title - Page 1 |
| ABSTRACT: |  |  |
| The abstracts need improvement especially the findings section by reporting the key findings as per the major themes and / or constructs of the Normalisation Process Theory, as reported in Table 3 and the main text. For example: COHERENCE (purpose of the system like Enhancing medication safety and improving cost effectiveness; Relationship of users to the technology; Engagement and communication between different stakeholders), COGNITIVE PARTICIPATION (Management of the profile of alerts) , COLLECTIVE ACTION (Prescribing in general practice; patient and population characteristics and engagement with patients; knowledge) REFLEXIVE MONITORING (Sustaining the use of the CDS through maintenance and customisation; Learning and behaviour change). | Thank you for this point. We have rewritten the results section of the abstract which now reads:  “Thirty-nine interviews were conducted either individually or in groups, with 33 stakeholders, including 11 follow-up interviews. Eight themes were interpreted in alignment with the four NPT constructs : Coherence (The purpose of the CDS: Enhancing medication safety and improving cost effectiveness; Relationship of users to the technology; Engagement and communication between different stakeholders); Cognitive Participation (Management of the profile of alerts); Collective Action (Prescribing in general practice, patient and population characteristics and engagement with patients; Knowledge);and Reflexive Monitoring (Sustaining the use of the CDS through maintenance and customisation; Learning and behaviour change. Participants saw that the CDS could have a role in enhancing medication safety and in the quality of care. Engagement through communication and support for local primary care providers and management leaders was considered important for successful implementation. Management of prescribing alert profiles for general practices was a dynamic process evolving over time. At regional management levels, work was required to adapt, and modify the system to optimise its use in practice and fulfil local priorities. Contextual factors, including patient and population characteristics, could impact upon the decision-making processes of prescribers influencing the response to alerts. The CDS could operate as a knowledge base allowing prescribers access to evidence-based information that they otherwise would not have.” | Abstract - Page 2-3 |
| The authors also mention that “there was a range of contextual factors’, which have not been reported. Could the authors the key contextual factors identified. | We have added to the abstract a phrase the outlining examples of the contextual factors. Please see the revised results section of the abstract above | Abstract - Page 2-3 |
| In addition, background and methods sections in the abstract may be shortened. | We have shortened the background and methods sections of the abstract. These now read:  “Background  The quality and safety of prescribing in general practice is important, Clinical decision support (CDS) systems can be used which present alerts to health professionals when prescribing in order to identify patients at risk of potentially hazardous prescribing. It is known that such computerised alerts may improve the safety of prescribing in hospitals but their implementation and sustainable use in general practice is less well understood. We aimed to understand the factors that influenced the successful implementation and sustained use in primary care of a CDS system.  Methods  Participants were purposively recruited from Clinical Commissioning Groups (CCGs) and general practices in the North West and East Midlands regions of England and from the CDS developers. We conducted face-to-face and telephone-based semi-structured qualitative interviews with staff stakeholders. A selection of participants was interviewed longitudinally to explore the further sustainability 1-2 years after implementation of the CDS system. The analysis, informed by Normalisation Process Theory (NPT), was thematic, iterative and conducted alongside data collection.” | Abstract – Page 2 |
| Conclusion: The conclusion only focuses on the safety and alerts but it does not reflect anything about cost saving which was the primary aim of the CCGs. This aspect needs to be reflected in the conclusion in the abstract, as reported in the conclusion section in the main text. | We have adapted the conclusion of the abstract to reflect the cost-saving aspect of the CDS which we agree was important. We have added a final sentence that reads:  “Shared understanding of the purpose of the CDS between CCGS and general practices particularly in balancing cost saving and safety messages could be beneficial” | Abstract – Page 3 |
| BACKGROUND: |  |  |
| CDC (page 4, lines 84-85): Could the authors add a citation/reference about the CSD system evaluated. In addition, the authors call the CDS sometimes as a system (page 4, line 87 and so on) and sometimes as software (page 4, lines 84-85). Is it a system or software? Please be precise and consistent. | Regretfully we are unable to provide a reference about the CDS system. This it is a commercial product and prior agreement with the developers of the CDS was that product name should remain anonymous. We feel the findings provide recommendations for the implementations of CDS in general as well as this specific system.  We agree that the use of system and software is inconsistent and imprecise. We have removed the use of ‘software’ throughout the manuscript. |  |
| CCG (page 4, line 87), Please spell out the acronym CCG here as it is mentioned for the first time, which has been spelled out later on page 5 (line 123). | Thank you. We have made this change. | Page 4  Page 5 |
| METHODS: |  |  |
| National Health Service (Pages 5-, lines 125-126): Acronym for the term ‘National Health Service’ has been given already in page 4, line 78; hence use only the acronym here (lines 125-126, page 5-6) | Thank you we have made this change. | Methods - Page 5 |
| Follow-up interviews (page 7, lines 1163-64): Follow-up interviews were conducted about 12 months after the first interviews. This is a long period. Did the follow-up interviews were conducted with the same persons / interviewees working on the same positions as in the first interviews? Were there any new people, and how many, involved in the follow-up interviews. | The interval before the follow-up interviews was designed to capture use after the initial implementation of the system. The same participants took part. We have added details of the follow up interviews in the method section. This now reads:  “Follow-up interviews were conducted approximately 12 months after the first interview with 11 participants who had taken part in first interviews., These participants were purposively selected to provide a range of different stakeholders, in order to understand changes that may have occurred and how the intervention was being sustained in everyday practice beyond the initial intervention period.” | Methods – page 7 |
| Shopping vouchers (page 7, lines 172-173): The authors paid £20 shopping vouchers to participants. Was the payment included in the ethics application? Did the authors paid/ gave vouchers to all participants or some participants and who were they. It seems unbelievable that a GP will accept/ask for £20 voucher for an interview! Most of the participants were employees of either the NHS or CCGs hence paid by them so why they were reimbursed for their time in the study? | Yes the payment was included in the ethics application and approvals. Resource consideration was a reason given for non-participation from general practices therefore the £20 voucher was useful as an incentive to take part. In our experience it is usual practice to compensate interview participants for their time. The GPs who took part in the study took time out from their working day. All CCG staff declined the vouchers. We have added this to the methods section to clarify this further.  “As approved by the ethics committee participants were offered a £20 shopping voucher per interview as reimbursement for their time. Vouchers were accepted by all staff in general practices who took time out from their working day to participate but declined by eight of the ten CCG staff.” | Methods – page 7 |
| DISCUSSION: |  |  |
| Strengths and limitations (page 27, lines 776-777): The authors have stated that This was the first qualitative study to utilise NPT to understand the complex processes that influenced the implementation and sustained use in primary care of a CDS system“. However, in the Methods section - Theoretical Framework: Normalization process theory (page 8, lines 192-205), they have reported that the NPT has been used in the evaluations of several health care interventional studies so why this study is the first to use the NPT in this study. The authors may check this claim. | We agree that this sentence is inappropriate and have removed this claim. | Discussion – Page 27 |
